# A theorized new class of polyhedral hydrocarbons of molecular formula C_n_H_n_ and their bottom-up scaffold expansions into hyperstructures

**DOI:** 10.1038/s41598-021-84562-6

**Published:** 2021-03-10

**Authors:** Camila M. B. Machado, Nathalia B. D. Lima, Sóstenes L. S. Lins, Alfredo M. Simas

**Affiliations:** 1 Departamento de Química Fundamental, CCEN, Universidade Federal de Pernambuco, Recife, Pernambuco 50740-560 Brazil; 2 Centro de Informática, CIN, Universidade Federal de Pernambuco, Recife, Pernambuco 50670-901 Brazil

**Keywords:** Chemistry, Theoretical chemistry

## Abstract

We address the use of Euler's theorem and topological algorithms to design 18 polyhedral hydrocarbons of general formula C_n_H_n_ that exist up to 28 vertexes containing four- and six-membered rings only; compounds we call “nuggets”. Subsequently, we evaluated their energies to verify the likelihood of their chemical existence. Among these compounds, 13 are novel systems, of which 3 exhibit chirality. Further, the ability of all nuggets to perform fusion reactions either through their square faces, or through their hexagonal faces was evaluated. Indeed, they are potentially able to form bottom-up derived molecular hyperstructures with great potential for several applications. By considering these fusion abilities, the growth of the nuggets into 1D, 2D, and 3D-scaffolds was studied. The results indicate that nugget_24a_ (C_24_H_24_) is predicted to be capable of carrying out fusion reactions. From nugget_24a_, we then designed 1D, 2D, and 3D-scaffolds that are predicted to be formed by favorable fusion reactions. Finally, a 3D-scaffold generated from nugget_24a_ exhibited potential to be employed as a voxel with a chemical structure remarkably similar to that of MOF ZIF-8. And, such a voxel, could in principle be employed to generate any 3D sculpture with nugget_24a_ as its level of finest granularity.

On a very thought-provoking article in New Scientist, entitled “Why think up new molecules?”, Prof. Roald Hoffman presented reasons to justify why he thinks that this is a worthwhile venture^1^. Speculative, inventive and somewhat risky predictions to either confront or make an exquisite use of a theory, are, by their very nature, scientific endeavors. As Prof. Roald Hoffman concludes, “The predictor leaves the safety of known molecules and properties for the unknown. He or she takes a risk. And, in a way, flirts—in a game of interest and synthesis—with the experimentalist.”^1^. In this article, we do indeed take this path and present a new subclass of hydrocarbons we call nuggets.

Polyhedral hydrocarbons of general formula C_n_H_n_ comprise a class of organic compounds that can exhibit unique properties, such as: tensioned bonds in rings that may be formed by three, four or more carbon atoms^2^; energy storage capability^3^; high density^3^; aromaticity or antiaromaticity^4^; magnetism^5^; and symmetry such as the ones exhibited by platonic solids and regular prisms^5^. However, due to their sometimes strongly stressed bonds, syntheses of polyhedral hydrocarbons are hardly easy. In this sense, Eaton et al.^6^ reported a synthetic strategy for the polyhedral hydrocarbon cubane (C_8_H_8_), which is a tetraprism system. Further, Katz et al., synthesized the C_6_H_6_ compound, which is a triprism system^7,8^. In particular, this compound exhibits a more tensioned structure than cubane^7,8^. In addition, the C_10_H_10_ polyhedral hydrocarbon was also synthesized^8,9^.

From a structural perspective, the bond angles of polyhedral hydrocarbons, that are either platonic or prismanes, are of smaller values (60°–90°), when compared with the most common bond angles of carbon atoms (109.5°). These small bond angles introduce a structural tension, which tends to energetically destabilize the system.

An interesting aspect of the polyhedral hydrocarbon cubane is its ability to store a large quantity of energy^10^. Based on the cubane synthesis, a set of derivatives was prepared that presented potential to be applied to materials science due to their cube fusion abilities. Examples of the cubane derivatives are the octamethylcubane^11^ and octacyclopropylcubane compounds^12^. In addition, Moran et al. evaluated the viability of carbon and hydrogen formed cages with ions, in which these systems have the potential to be applied in magnetic resonance, acting as contrast agents, with semiconductive and ferromagnetic properties^13^. Cubane derivatives can also be employed as additives, for example, in fuel, due to their tensioned structures^14^. In addition, 4-methyl-cuban-1-amine and 4-methyl-cuban-1-methylamine compounds exhibited antiviral biological activity^15^. Finally, if synthesized in larger amounts, heptanitrocubane would perhaps be one of the most effective non-nuclear explosives possible^16^.

Poater et al. studied several structural and energy aspects of a class of packed carbon nanoneedles, that were conceptualized by stacking up units of 4, 6, and 8 carbons with potential applications to nanomedicine by acting as drug carriers through nonpolar biologic media^17^. The ability of the polyhedral hydrocarbons to be structurally fused was further examined by Katin et al.^18^ The authors studied a material based on polyprismanes and concluded that these systems are similar to the carbon nanotube^18^. In addition, the interactions of the orbitals between the parallel rings of these materials seem to be the main component associated with the stability of the systems^19^.

Karpushenkava et al.^20^, studied both structural and vibrational properties of a set of polyhedral hydrocarbons of the C_n_H_n_ cage class in gas phase. The authors concluded that when the energy associated with the cage tension is either negative or slightly positive, the corresponding compounds could be synthesized. An unique exception was verified for a triprism compound with a cage energy of + 55.2 kJ mol^−1^(ref^20^).

Wang et al., reported three stable isomers of the type C_24_H_24_. In their article, G3(MP2) calculations revealed that the optimized geometries of these systems have a positive value for Δ_f_H^21^. These geometries are unstable when compared to their fullerene isomers. In addition, one of the structures formed with Si has the potential to be a semiconductor material and, by replacing the CH groups with nitrogen atoms, high-energy density materials can be prepared^21^.

On the other hand, DFT methods were also employed by Shamov et al.^22^ to predict both structural and energy properties of a set of C_n_H_n_ compounds, with n being 12, 16, 20, and 24. Both C_12_H_12_ and C_20_H_20_ compounds were synthesized, and the energetic properties indicated that C_16_H_16_ and C_24_H_24_ could be prepared. In this sense, Ohno et al., investigated both dimers and trimers of the regular prisms, with 6, 10, 12, 14, 16, 18 and 20 faces, connected by cubane-shaped bridges^23^. Their results also revealed that these compounds are able to be formed in organic reactions at low temperatures. Moreover, due to the metastable nature of the regular prismatic compounds, they could be potentially employed, for example, in energy storage^23^.

In this article, we employ Euler's theorem to deduce polyhedra containing four- and six- membered rings that exist up to 28 vertexes, that we call “nuggets”. We then evaluate their energetics in order to conjecture the likelihood of their existence. Finally, because all nuggets can be fused together in several manners, either through their square faces, or through their hexagonal faces, we investigated the fusion abilities of this set of nuggets to investigate the perspectives for their growth into 1D, 2D, and 3D-scaffolds.

## Results and discussion

### The nuggets structural possibilities from Euler's theorem

Our intention was to design hydrocarbon polyhedra that could be potentially stable. Although there are polyhedral hydrocarbons of the type C_n_H_n_ with triangular faces, such as the tetrahedron^24^ and the triprism^25,26^, as well as ones with pentagonal faces, such as the dodecahedron and the pentaprism^9,26^, we decided to restrict our work to polyhedra whose faces are polygons with an even number of vertices. Such systems can have alternating double bonds, thus potentially displaying energy stabilization due to electronic delocalization.

**Figure 1:**
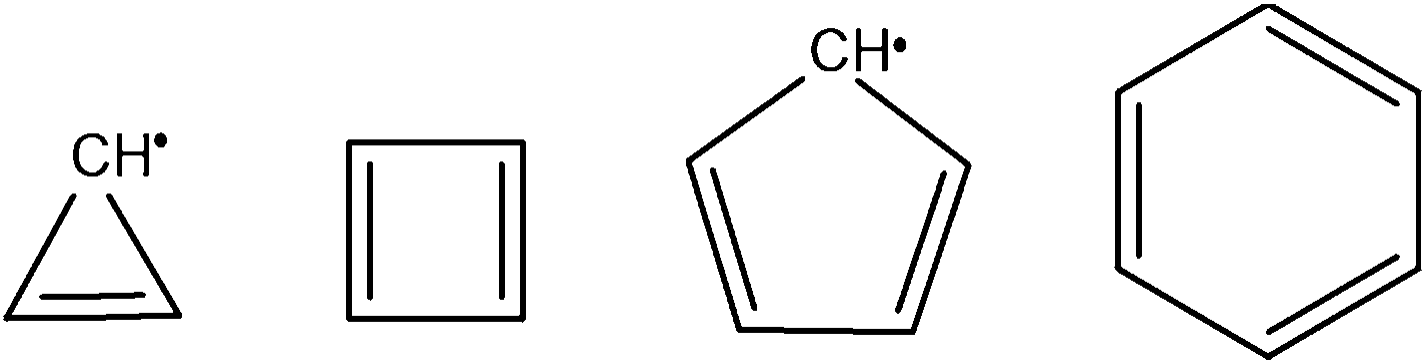
Chemical structures of cyclopropenyl radical, cyclobutadiene, cyclopentadienyl radical and benzene compounds.

Let us first consider polygonal hydrocarbons of formula C_n_H_n_. The smallest polygon with this formula is triangular C_3_H_3_. However, C_3_H_3_ is a radical system. The same happens with C_5_H_5_, as shown in Fig. 1. Actually, all neutral polygonal C_n_H_n_ hydrocarbons with n being an odd number must be radical systems.

**Figure 2:**
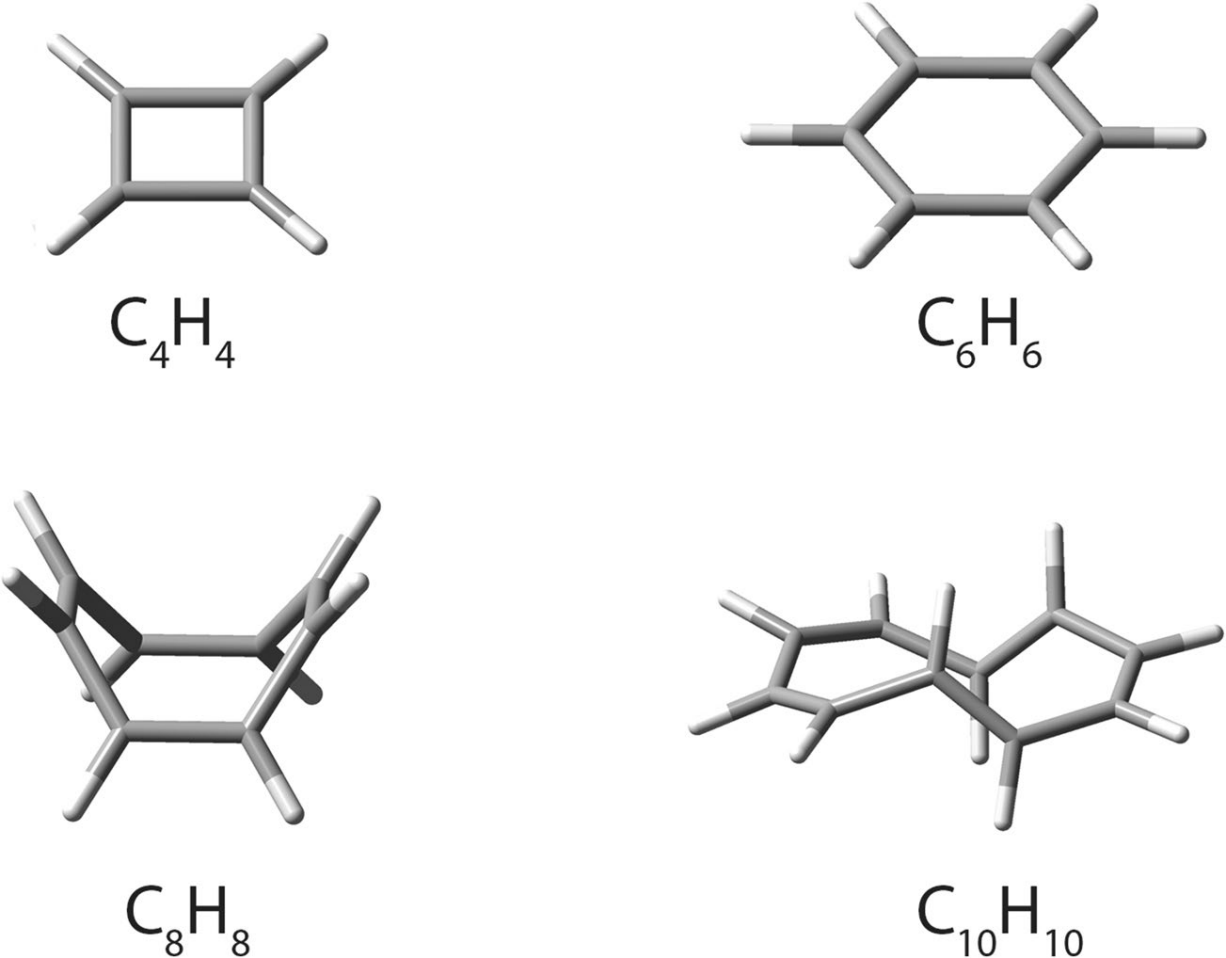
DFT ωB97XD/6-31G* optimized geometries of cyclobutadiene, C_4_H_4_, benzene, C_6_H_6_*,* cyclooctatetraene, C_8_H_8_, and cyclodecapentaene, C_10_H_10_.

On the other hand, when n is an even number with n ≥ 4, the C_n_H_n_ polygonal hydrocarbons are neutral systems, with cyclobutadiene, C_4_H_4_, and benzene, C_6_H_6_, displaying planar structures and thus being the most important members of this class. But, when n is equal to or larger than 8, the compounds become non-planar^27^. Figure 2 shows images of these polygonal compounds up to n = 10.

Because we intend to grow the polyhedra into 1D, 2D, and 3D-scaffolds by fusing together their polygonal faces, we will restrict the polyhedra in this work to those with square and hexagonal faces only, since it would be very difficult, if not impossible, to fuse together two significantly non-planar and twisted faces. In these polyhedral compounds, each carbon atom must be bound to a single hydrogen atom as well as to three other carbon atoms as well.

Euler's theorem^28^ defines a relation between the numbers of faces, edges and vertices for any simple polyhedron: the polyhedra of our interest. Simple polyhedra are topologically equivalent to a sphere, that is, these systems are polyhedra that have no central cavities as “donuts”. Therefore, if inflated, in the limit, these systems would become spheres. There are two possibilities for a hydrogen atom bonded to a carbon atom in a carbon polyhedron: either it is located inside or outside the polyhedron. If the hydrogen atoms appear in the interior of the polyhedron, steric effects would be very significant due to the congestion between other hydrogen or carbon atoms, especially for the smaller polyhedra. Moreover, if all hydrogen atoms always point inwards, at least one hydrogen atom would have an HCC angle less than 90°, which is not reasonable from the point of view of chemical bonds. Therefore, to be chemically realistic in applying Euler's theorem, we will focus on carbon polyhedra with the hydrogen atoms of the CH bonds always pointing outwards.

Euler's theorem for simple polyhedra relates the number of faces (F), edges (E), and vertices (V) by the formula:

1 $${\text{V}} - {\text{E}} + {\text{F}} = {2}$$

where V is the number of vertices, E is the number of edges and F is the number of faces.

When the polyhedron has only square and hexagonal faces, such as the nuggets, then

2 $${\text{F}} = {\text{F}}_{{4}} + {\text{F}}_{{6}}$$

where F_4_ is the number of square faces, and F_6_ is the number of hexagonal faces.

Of course, each square face of the polyhedron delimits four edges, and each hexagonal face delimits six. However, if the edges are counted from each polyhedral face, they would be counted twice, since each and every edge of the polyhedron is shared by exactly two faces. Accordingly, the relation between the number of edges, E, and the number of square and hexagonal faces of such a polyhedron is given by the following equation:

3 $${\text{2E}} = {\text{4F}}_{{4}} + {\text{6F}}_{{6}}$$

For our polyhedra, the number of vertices is represented by the union of three edges. That is, each carbon atom is chemically bonded to exactly three other carbon atoms, i.e. V = V_3_; the fourth bond being to a hydrogen atom. And each edge is bounded by two distinct end points: the vertices. Therefore, the relation between the number of edges and the number of vertices is given by:

4 $${\text{2E}} = {\text{3V}}_{{3}}$$

From Euler’s formula, Eqs. (1), and (4):

5 $$F=2+\frac{E}{3}$$

From Eqs. (2), (3), and (5), we obtain:

6 $${F}_{4}+ {F}_{6}=2+ \frac{1}{6}\left(4{F}_{4}+6{F}_{6}\right)$$

7 $${6F}_{4}+ {6F}_{6}=12+ 4{F}_{4}+6{F}_{6}$$

**Figure 3:**
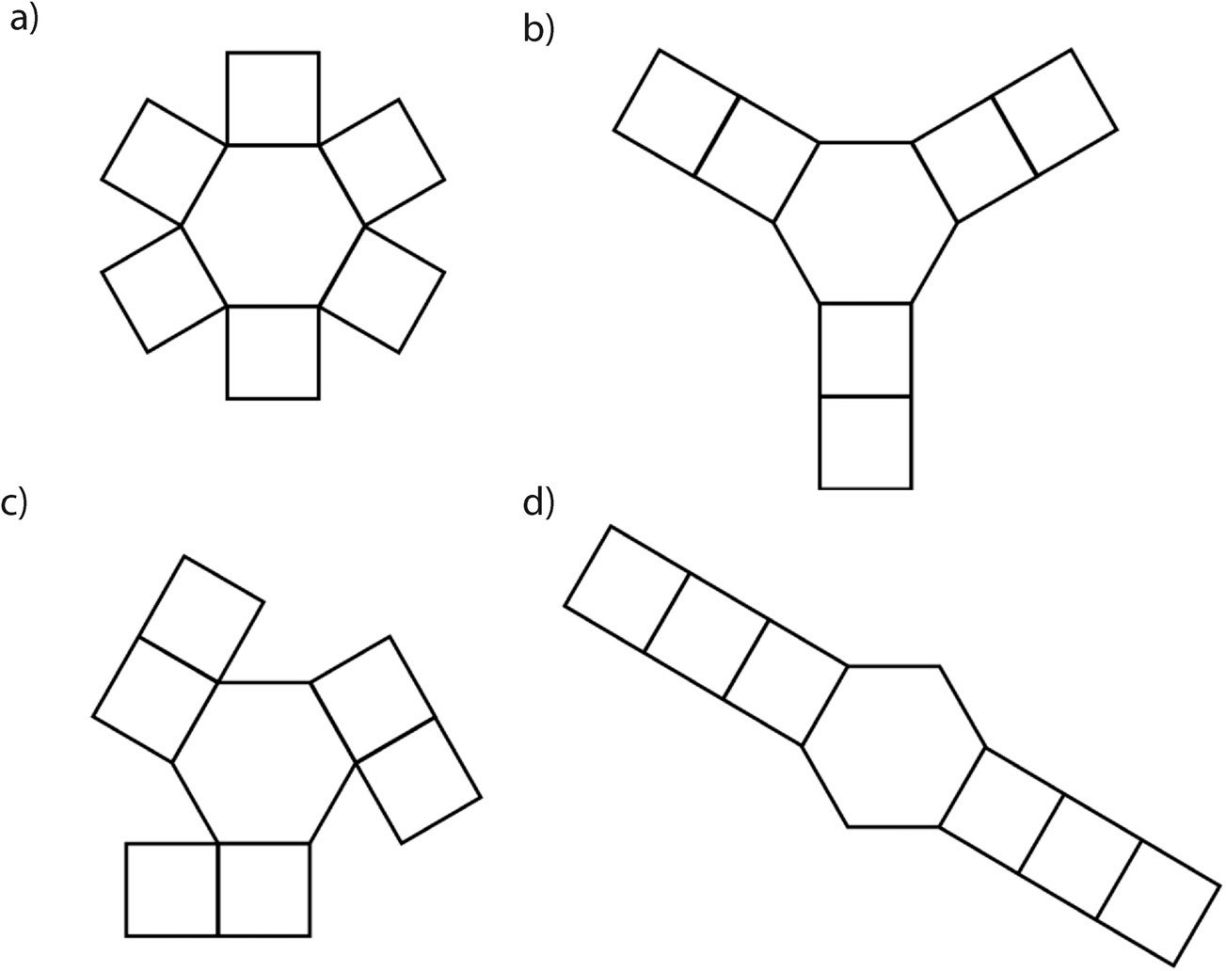
Planar configurations of one hexagon and six squares, from which one can intuit that it would be impossible for any of them, or any other one for that matter to be closed as a 3D polyhedron without creating at least a second hexagonal face.

By simplifying the term 6F_6_ on both sides of Eq. (7), we finally obtain that F_4_ = 6. This reveals that any simple polyhedron that has only square and hexagonal faces must always have 6 square faces for an arbitrary number of hexagonal faces, except one. This exception is because Euler’s formula is a necessary, but not sufficient condition for a polyhedron to exist. As can be intuited from Fig. 3 a configuration of one hexagon and six squares cannot be possibly closed into a polyhedron without forming at least a second hexagonal face. Consequently, the number of hexagonal faces must be either 0 (for the cube), or equal or greater than 2 for a constant number of six square faces.

### Design and computational details

The software Blink^29^ developed by the research group of one of us (SL) was used to generate a set of unique nuggets from the cube up to the three different solids with 28 vertexes, all with 6 square faces and up to 10 hexagonal faces. From graph encoded manifold and UNIVs data^30^ the Blink software is capable of generating several representations of graphs, only formed by faces with even numbers of vertices—squares, hexagons, octagons, etc. In this article, the Blink software was employed to map the topologically different and possible shapes of up to n = 28 vertices. Among all possibilities generated, we selected, according to chemical criteria, a subclass that we call nuggets that is composed of those that have structural forms containing six squares and an arbitrary number of hexagons, either equal to zero, or greater than or equal to two, generating a set of three-dimensional representations of the nuggets. From this class, we selected the first 18 that led to chemically different structures^29,30^.

**Figure 4:**
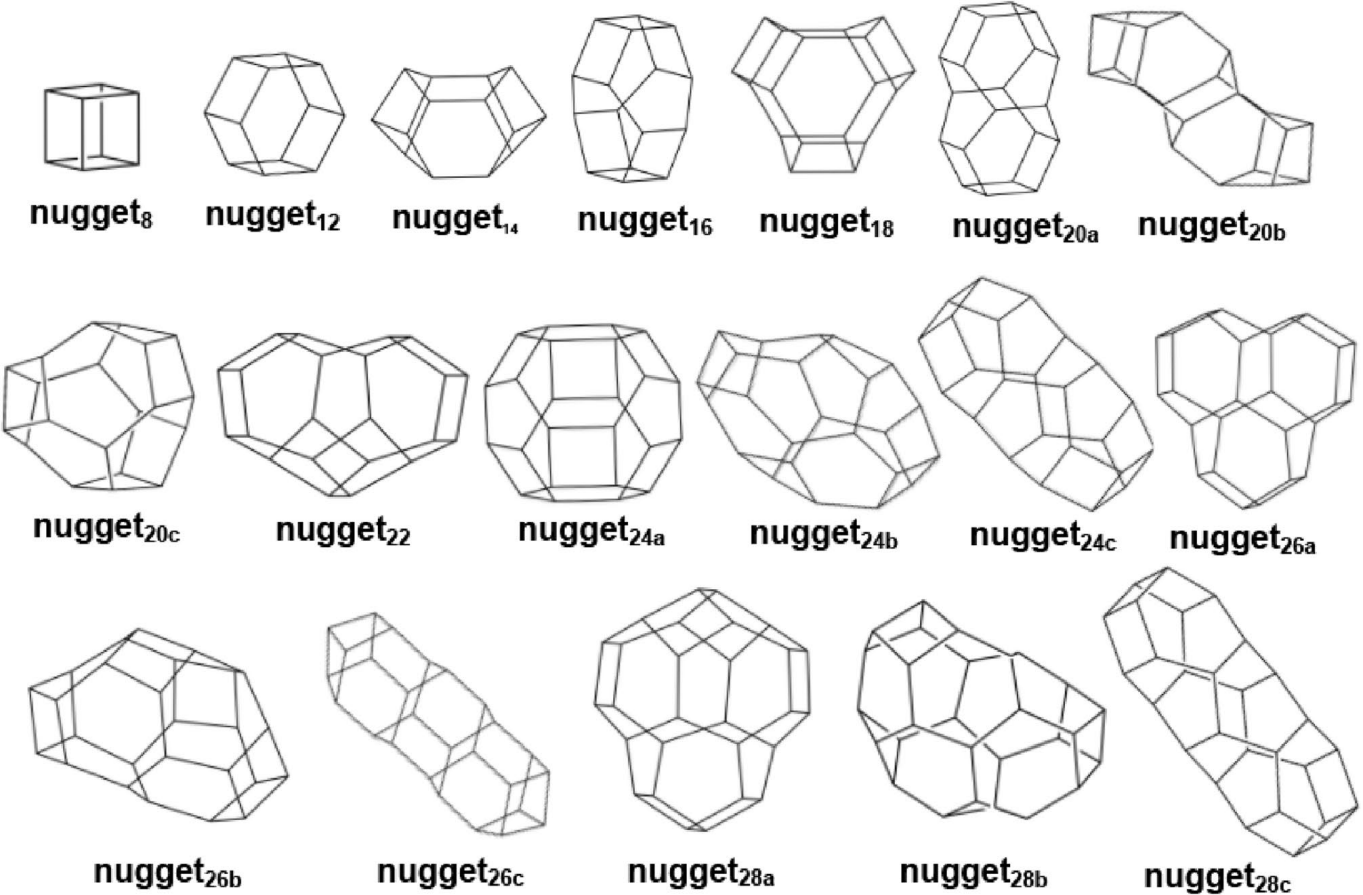
Chemical structures of the 18 nuggets generated by the Blink software.

Hence, we generated a set of all different such polyhedra, starting with the cube, C_8_H_8_, up to those containing 28 vertices, of empirical formula C_28_H_28_, a number which we found to be reasonable to explore from a chemical point of view. Figure 4 shows the chemical structures of all 18 nuggets obtained, identified by the number of vertices, that is of carbon atoms, which is identical to the number of hydrogen atoms, and an additional letter in case there are more than one such nuggets for a given number of vertices.

Being fully aware that predicting the properties of unusual molecules is risky, in order to calculate structural, vibrational and energy properties of the set of 18 nuggets, we needed to choose a quantum chemical model chemistry that would be at the same time both accurate enough and workable, given the size of the systems that we want to study, to be able to make educated inferences on the prospects of their chemical realities. We thus chose the ωB97XD functional by Chai and Head-Gordon because of its inclusion of a version of empirical Grimme’s D2 dispersion as well as long-range correction with superior results^31^, together with the 6-31G* basis set of Petersson et al.^32^, for ease of computation of the larger hyperstructures formed by the molecular building blocks. Accordingly, all geometries of the designed nuggets, as well as the more complex 1D, 2D, and 3D systems were fully optimized by ωB97XD/6-31G* calculations via both Spartan’14^33^ and Gaussian09^34^ softwares. All structures have been characterized to be minima with frequency calculations.

### Nuggets exhibiting polyhedral chirality

**Figure 5:**
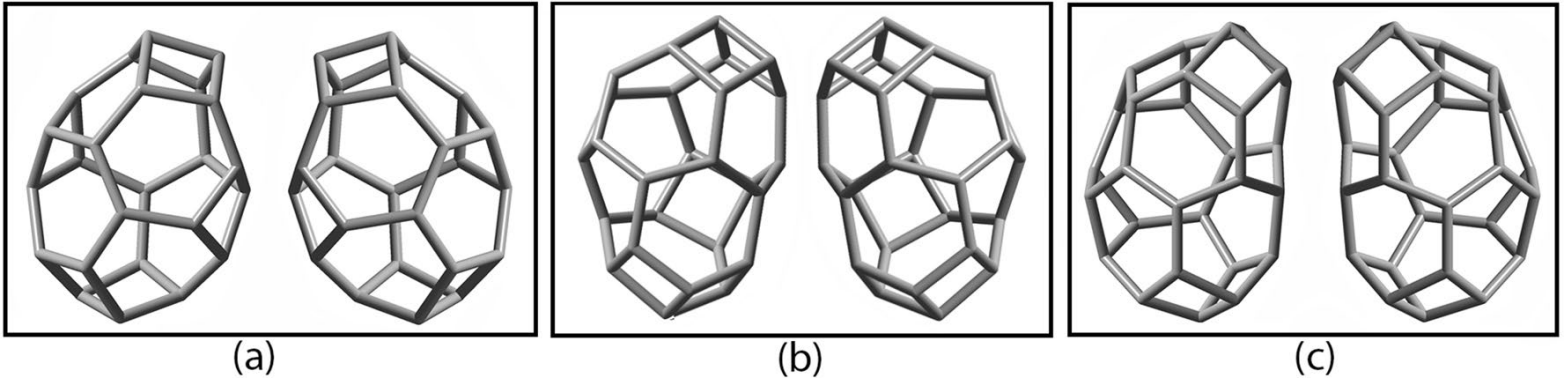
DFT ωB97XD/6-31G* optimized geometries of the following pairs of chiral nuggets: (**a**) nugget_24b_; (**b**) nugget_26b_; and (**c**) nugget_28b_.

From Fig. 4, nugget_24b_, nugget_26b_, and nugget_28b_ exhibit polyhedral chiral properties, as can be seen, in an illustrative manner, in Fig. 5, below, where we represent their respective pair of enantiomers.

### Nuggets as voxels

Voxels are the three-dimensional (3D) equivalents of pixels. Analogously to pixels, which can be used to generate any 2D images by juxtaposition, voxels can be likewise used to generate any 3D sculptures. Voxels can be virtual as in computer 3D graphics or real as in 3D printers.

For a carbon polyhedron to be able to efficiently function as a voxel, it should possess the important property of 3D space-filling. That property being satisfied, they could in principle perhaps function as solid controllable building blocks that could be used to assemble any arbitrary 3D structures by juxtaposition.

Of all nuggets that we studied, in only three of them, the carbon atoms define space-filling polyhedra that could function as chemical voxels: nugget_8_ (cubane), nugget_12_ (hexaprismane or [6]-prismane) and nugget_24a_ (a truncated octahedron hydrocarbon).

**Figure 6:**
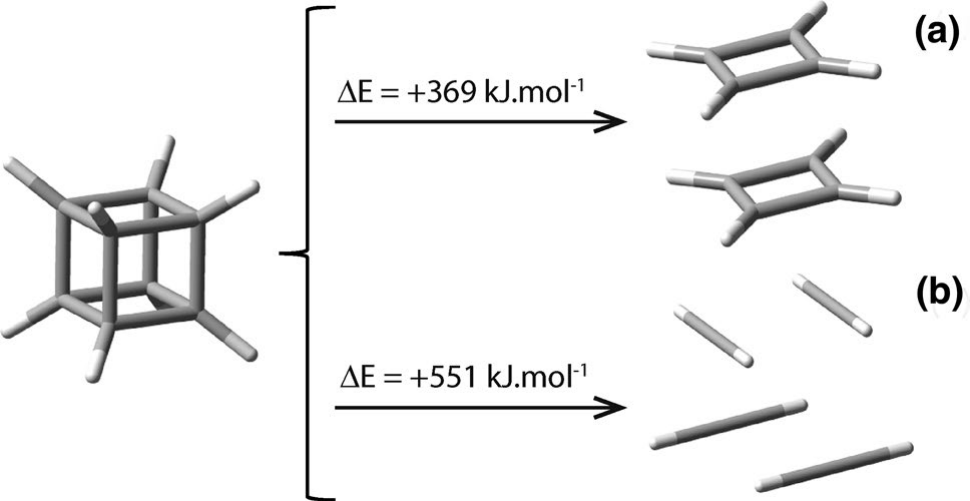
Pictorial representation of the dissociation reaction of nugget_8_ into (**a**) two cyclobutadiene compounds, C_8_H_8_ → 2C_4_H_4_, and (**b**) four ethyne molecules C_8_H_8_ → 4C_2_H_2_. The values of ΔE shown are from ωB97XD/6-31G* calculations and are given in kJ mol^−1^ units because they refer to chemical reactions involving one mole of reactant only.

Let us first consider nugget_8_ (cubane), of point group O_h_. Cubane’s chemical stability with respect to self-decomposition in the absence of any other reagents is something that can be inferred from its corresponding calculated energy change of reaction. Accordingly, let us consider the possibility of a nugget_8_, cubane, molecule dissociating into either 2 molecules of cyclobutadiene (C_8_H_8_ → 2C_4_H_4_), or into 4 molecules of ethyne (C_8_H_8_ → 4C_2_H_2_), Fig. 6.

The ΔE^ωB97XD/6-31G*^ values for these reactions are equal to + 368.8 kJ mol^−1^ and 551.2 kJ mol^−1^; large values that prevent such dissociation from occurring despite cubane’s highly tensioned cubic structure. These ΔE^ωB97XD/6-31G*^ values indicate that these entropy-favored self-decompositions, are unlikely to occur spontaneously. These findings are consistent with the fact that, as previously mentioned, cubane (nugget_8_) has already been prepared^6^. Further, cubane growth in three dimensions is predicted to be a stable allotrope of carbon. Actually, a carbon allotrope with this 3D-structure could be very well used as an energy storage compound and would probably exhibit a larger mass density when compared with all other allotropes of carbon, including diamond.

**Figure 7:**
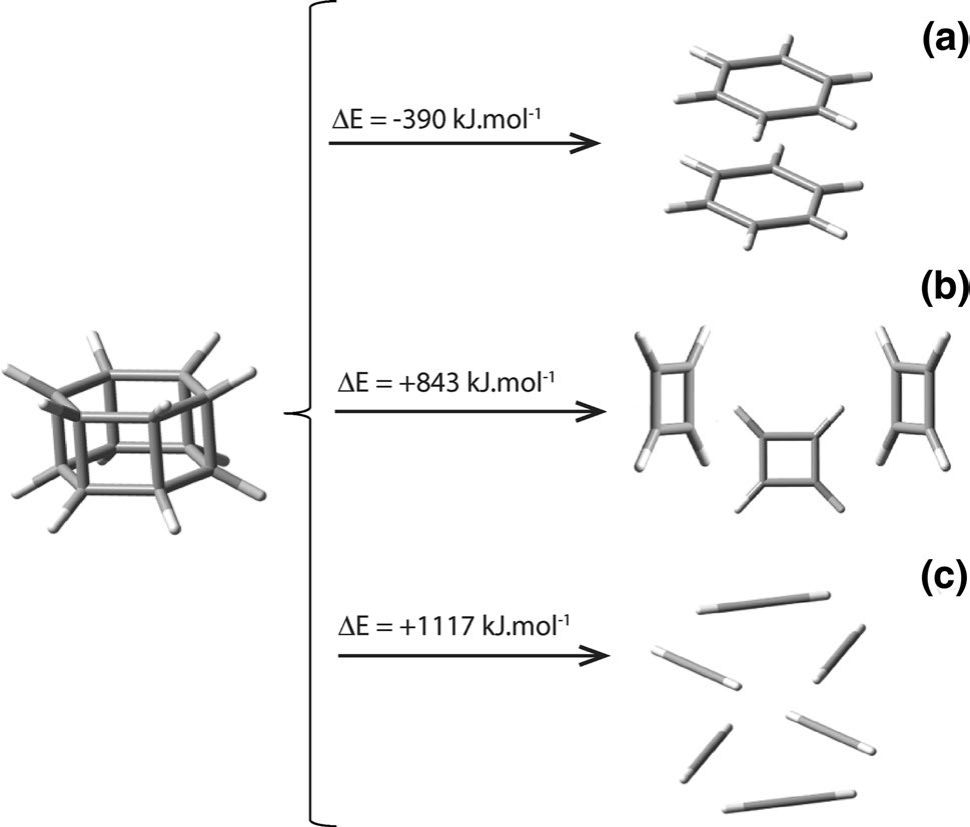
Pictorial representation of the dissociation reaction of nugget_12_ into (**a**) two benzene molecules, C_12_H_12_ → 2C_6_H_6_, (**b**) three cyclobutadiene compounds C_12_H_12_ → 3C_4_H_4_, and (**c**) six ethyne molecules C_12_H_12_ → 6C_2_H_2_. The values of ΔE shown are from ωB97XD/6-31G* calculations and are given in kJ mol^−1^ units because they refer to chemical reactions involving one mole of reactant only.

Let us now examine the case of nugget_12_, the hexaprismane, which has the structure of a prism with two parallel hexagonal faces linked through six square faces (Fig. 4). Hexaprismane can be thought of as a face-to-face dimer of benzene. The calculated energy of dissociation of nugget_12_ into two benzene molecules (C_12_H_12_ → 2C_6_H_6_) Fig. 7a, yields a ΔE^ωB97XD/6-31G*^ = **−** 389.8 kJ mol^−1^, indicating that, in this case, the spontaneous chemical self-decomposition of hexaprismane is predicted to be highly likely to occur. As a reinforcement to this affirmation, the thermal cycloaddition of two benzene molecules [6 + 6] is symmetry forbidden^35^. Indeed, so far and despite many attempts, nugget_12_, C_12_H_12_, the hexaprismane, has never been synthesized. These facts point further in the direction that the growth of nugget_12_ to three dimensions would quickly spontaneously transform such a hypothetical solid into superimposed layers of graphene, such as graphite. Recently, a vertical stacking of graphene has been evolved into materials with highly tunable electronic properties and unique functionalities: the van der Waals heterostructures (vdWHs)^36^. So, for all practical purposes, it is very unlikely that the hexaprismane hydrocarbon nugget_12_ could ever be of practical use as a chemical voxel. Nevertheless, the geometric concept of an hexaprismane polyhedron as a chemical voxel has recently been realized by the synthesis of isoreticular pillar layered metal organic frameworks exhibiting properties such as catalytic activity^37^. Two other self-dissociation reactions that could be thought of for the hexaprismane nugget_12_ would be: (i) self-dissociation into 3 cyclobutadiene molecules, that is: C_12_H_12_ → 3C_4_H_4_ with a ΔE^ωB97XD/6-31G*^ value of + 843.2 kJ, and (ii) self-dissociation into 6 ethyne molecules, C_12_H_12_ → 6C_2_H_2_, with a ΔE^ωB97XD/6-31G*^ value of + 1116.8 kJ, as can be seen in Fig. 7b,c, respectively. These two large positive calculated values reveal, as expected, that the self-decomposition of hexaprismane nugget_12_ into two benzene molecules is the one most likely to occur spontaneously.

The third and last carbon voxel is nugget_24a_, which has the geometric form of a truncated octahedron: a space-filling Archimedean solid displaying many geometric properties, nugget_24a_ is a hydrocarbon, not the C24 fullerene which presents the same carbon structure^38^, which is geometrically equivalent to both the B_12_N_12_ Fullerene reported by Matxain et al.^39^ as well as to ZIF-8, a very stable and largely researched metal–organic framework, MOF^40^.

**Figure 8:**
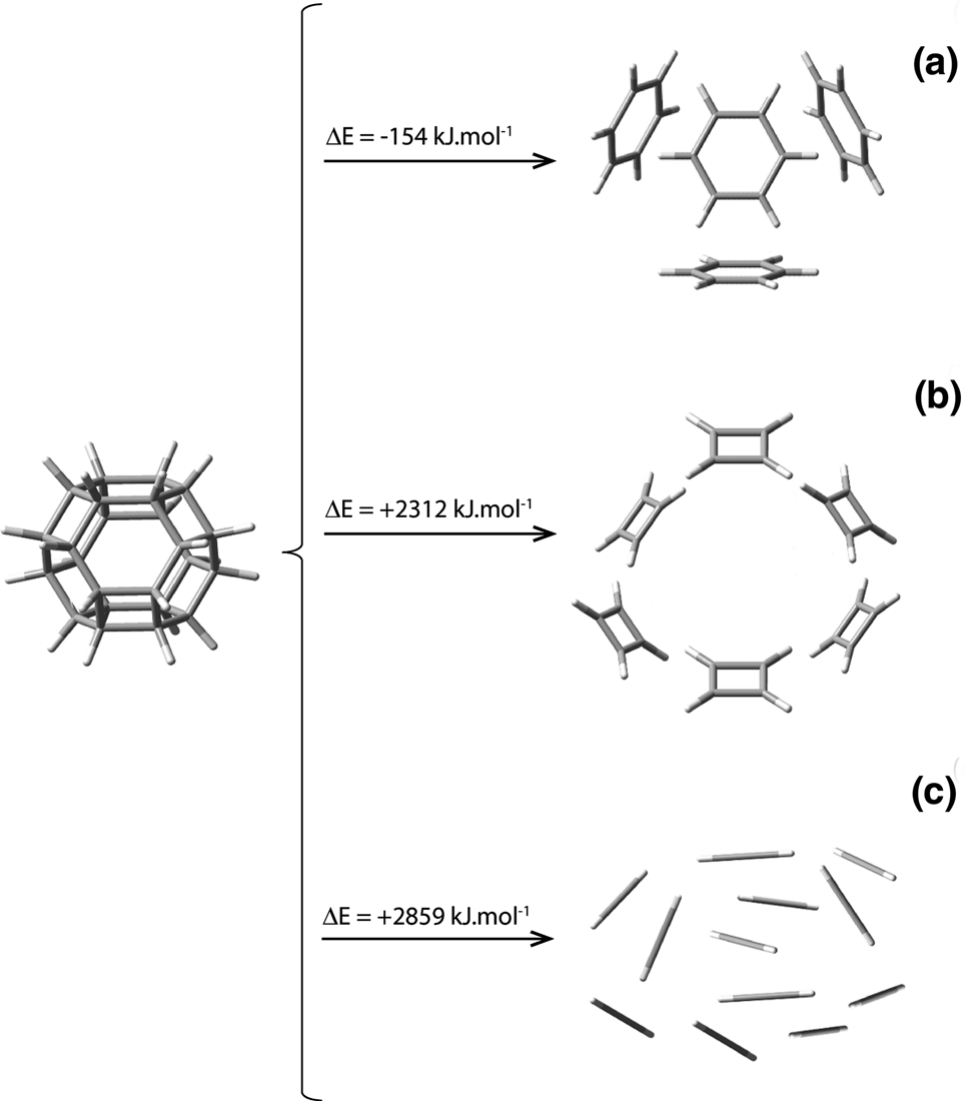
Pictorial representation of the dissociation reaction of nugget_24a_ into (**a**) four benzene molecules, C_24_H_24_ → 4C_6_H_6_, (**b**) six cyclobutadiene compounds C_24_H_24_ → 6C_4_H_4_, and (**c**) twelve ethyne molecules C_24_H_24_ → 12C_2_H_2_. The values of ΔE shown are from ωB97XD/6-31G* calculations and are given in kJ mol^−1^ units because they refer to chemical reactions involving one mole of reactant only.

Due to its high symmetry, and much less strained chemical bonds than either cubane or hexaprismane, nugget_24a_ is a possibility to be considered as a carbon voxel. Let us now proceed by first examining its three possible forms of self-decomposition of nugget_24a_: (a) into 4 benzene molecules, with a ΔE^ωB97XD/6-31G*^ value of − 154.3 kJ; (b) into 6 cyclobutadiene molecules, with a ΔE^ωB97XD/6-31G*^ value of + 2311.6 kJ; and (c) into 12 acetylene molecules, with a ΔE^ωB97XD/6-31G*^ value of + 2858.9 kJ, Fig. 8a–c, respectively.

These results indicate that nugget_24a_, although possibly unstable with respect to a self-decomposition into 4 benzene molecules, can be expanded as voxel into a 3D solid that would constitute an allotrope form of carbon. By being constituted by carbon atoms only, and noncoplanar vicinal six-membered rings, it cannot be split into benzene molecules or into graphene layers that would benefit from electron delocalization for stabilization. The geometric arrangement of the carbon-only hexagons in a such a perfectly packed 3D solid, placing each and every carbon atom in a condition of equilibrium of forces, would most certainly prevent its dismantling. Its infinite 3D expansion leads to a carbon-only solid compound which would constitute an allotrope of carbon. So much so that a sample has been found and properly characterized as a natural, super-hard, and transparent crystalline polymorph of carbon from the Popigai impact crater in Russia, formed because of a natural shockwave event^41^, and established to be consistent with such structure^42^.

### Stability of the nuggets

Now, we turn our attention to the structural stabilities of the non-voxel nuggets. Due to their molecular formula, their self-dissociation into ring compounds is a bit more complex, necessarily being at least into a mixture of benzene and cyclobutadiene, according to

$${\text{C}}_{{\text{n}}} {\text{H}}_{{\text{n}}} \to {\text{pC}}_{{6}} {\text{H}}_{{6}} + {\text{ qC}}_{{4}} {\text{H}}_{{4}}$$

where n = 6p + 4q, with n, p, and q being integers. Further, there can be multiple combinations of p and q integer numbers that solve this expression for a given integer value of n. However, due to their geometric shapes, it is not always possible for these nuggets to be disassembled into combinations of benzene and cyclobutadiene molecules according to any stoichiometrically possible pair of values of p and q. Indeed, some of these disconnections could be shape forbidden. Finally, self-dissociations could also happen into ethyne molecules according to C_n_H_n_ → (n/2)C_2_H_2_, a reaction that would always be possible since n is necessarily an even number and there are no geometric restrictions for any edges to be detached from the polyhedra. Table 1 shows ωB97XD/6-31G* calculated energies of reaction for all possible shape-allowed self-dissociations of all studied nuggets.

**Table 1:** Energy values for the shape allowed dissociation reactions of the studied nuggets into either C_6_H_6_ and/or C_4_H_4_ compounds, or into C_2_H_2_. All values were calculated by the model chemistry ωB97XD/6-31G*. The energy values are given in kJ mol^−1^ units because they refer to chemical reactions involving one mole of reactant only.

Compound	Reaction	ΔEω^B97XD/6-31G^* (kJ mol^−1^)
Nugget_8_	C_8_H_8_ → 2C_4_H_4_	+ 368.8
	C_8_H_8_ → 4C_2_H_2_	+551.2
Nugget_12_	C_12_H_12_ → 2C_6_H_6_	− 389.8
	C_12_H_12_ → 3C_4_H_4_	+843.2
	C_12_H_12_ → 6C_2_H_2_	+1116.8
Nugget_14_	C_14_H_14_ → C_6_H_6_ + 2C_4_H_4_	+ 563.0
	C_14_H_14_ → 7C_2_H_2_	+1498.7
Nugget_16_	C_16_H_16_ → 4C_4_H_4_	+ 1412.6
	C_16_H_16_ → 2C_6_H_6_ + C_4_H_4_	+ 179.6
	C_16_H_16_ → 8 C_2_H_2_	+1777.5
Nugget_18_	C_18_H_18_ → 3C_6_H_6_	− 198.9
	C_18_H_18_ → C_6_H_6_ + 3C_4_H_4_	+ 1034.1
	C_18_H_18_ → 9C_2_H_2_	+2061.1
Nugget_20a_	C_20_H_20_ → 2C_6_H_6_ + 2C_4_H_4_	+ 650.5
	C_20_H_20_ → 10C_2_H_2_	+2339.5
Nugget_20b_	C_20_H_20_ → 2C_6_H_6_ + 2C_4_H_4_	+ 684.2
	C_20_H_20_ → 10C_2_H_2_	+2373.3
Nugget_20c_	C_20_H_20_ → 2C_6_H_6_ + 2C_4_H_4_	+ 621.0
	C_20_H_20_ → 10C_2_H_2_	+2310.0
Nugget_22_	C_22_H_22_ → 3C_6_H_6_ + C_4_H_4_	+ 260.4
	C_22_H_22_ → C_6_H_6_ + 4C_4_H_4_	+ 1493.4
	C_22_H_22_ → 11C_2_H_2_	+2611.5
Nugget_24a_	C_24_H_24_ → 4C_6_H_6_	− 154.3
	C_24_H_24_ → 6C_4_H_4_	+ 2311.6
	C_24_H_24_ → 12C_2_H_2_	+2858.9
Nugget_24b_	C_24_H_24_ → 4C_6_H_6_	− 186.6
	C_24_H_24_ → 2C_6_H_6_ + 3C_4_H_4_	+ 1046.3
	C_24_H_24_ → 12C_2_H_2_	+2826.6
Nugget_24c_	C_24_H_24_ → 4C_6_H_6_	− 97.2
	C_24_H_24_ → 2C_6_H_6_ + 3C_4_H_4_	+ 1135.8
	C_24_H_24_ → 12C_2_H_2_	+2916.1
Nugget_26a_	C_26_H_26_ → 3C_6_H_6_ + 2C_4_H_4_	+ 651.9
	C_26_H_26_ → 13C_2_H_2_	+3094.3
Nugget_26b_	C_26_H_26_ → 3C_6_H_6_ + 2C_4_H_4_	+ 694.8
	C_26_H_26_ → 13C_2_H_2_	+3137.2
Nugget_26c_	C_26_H_26_ → 3C_6_H_6_ + 2C_4_H_4_	+ 783.2
	C_26_H_26_ → C_6_H_6_ + 5C_4_H_4_	+ 2016,2
	C_26_H_26_ → 13C_2_H_2_	+3225.6
Nugget_28a_	C_28_H_28_ → 4C_6_H_6_ + C_4_H_4_	+ 244.6
	C_28_H_28_ → 2C_6_H_6_ + 4C_4_H_4_	+ 1477.6
	C_28_H_28_ → 14C_2_H_2_	+3349.1
Nugget_28b_	C_28_H_28_ → 4C_6_H_6_ + C_4_H_4_	+ 245.1
	C_28_H_28_ → 2C_6_H_6_ + 4C_4_H_4_	+ 1478.1
	C_28_H_28_ → 14C_2_H_2_	+3349.6
Nugget_28c_	C_28_H_28_ → 4C_6_H_6_ + C_4_H_4_	+ 394.8
	C_28_H_28_ → 2C_6_H_6_ + 4C_4_H_4_	+ 1627.8
	C_28_H_28_ → 14C_2_H_2_	+3499.3

From Table 1, complete dissociations into ethyne molecules are unlikely to happen for all nuggets, the same happening for self-dissociations producing any number of cyclobutadiene molecules. Thus, we can divide the nuggets into two groups, according to their energies of self-dissociation reaction ΔE^ωB97XD/6-31G*^.

The first group of nuggets is comprised by the ones with at least one of the calculated ΔE values being negative: nugget_12_ (hexaprismane), nugget_18_, and all nuggets_24_ (including the truncated octahedron, nugget_24a_). These are the nuggets that may perhaps be less stable.

The second group of potentially more stable nuggets comprises nuggets 8, 14, 16, 20 (a,b,c), 22, 26 (a,b,c) and 28 (a,b,c). This group includes nugget_28b_ which exhibits polyhedral chirality. As far as we know, so far, none of them have been reported in the literature, not even as a theoretical possibility. These results reveal that most of the designed nuggets are seemingly energetically stable and, probably, not easily capable of self- dissociation into simpler organic compounds.

On the other hand, the nuggets of formula C_20_H_20_, C_24_H_24_, C_26_H_26_, and C_28_H_28_ possess structural isomers. Table 2 shows the energy of isomerization for all energetically favorable possibilities between these isomers. From Table 2, the most stable isomers for each of the molecular formulas are nugget_20c_, nugget_24b_, nugget_26a_, and nugget_28a_. However, transformation of one of the isomers into the other, involves fracturing a relatively rigid polyhedron through rearrangements of chemical bonds, thus rendering this type of transformation not likely.

**Table 2:** Isomerization energies between structural isomers for each of the following molecular formulas: C_20_H_20_, C_24_H_24_, C_26_H_26_, and C_28_H_28_. All values were calculated by the model chemistry ωB97XD/6-31G*. The energy values are given in kJ mol^−1^ because they refer to chemical reactions involving one mole of reactant only.

Molecular formula	Reaction	Isomerization energies (kJ mol^−1^)
C_20_H_20_	Nugget_20a_ → Nugget_20b_	− 33.7
	Nugget_20c_ → Nugget_20a_	− 29.5
	Nugget_20c_ → Nugget_20b_	− 63.2
C_24_H_24_	Nugget_24b_ → Nugget_24a_	− 32.3
	Nugget_24a_ → Nugget_24c_	− 57.1
	Nugget_24b_ → Nugget_24c_	− 89.4
C_26_H_26_	Nugget_26a_ → Nugget_26b_	− 42.9
	Nugget_26a_ → Nugget_26c_	− 131.3
	Nugget_26b_ → Nugget_26c_	− 88.4
C_28_H_28_	Nugget_28a_ → Nugget_28b_	− 0.5
	Nugget_28a_ → Nugget_28c_	− 150.2
	Nugget_28b_ → Nugget_28c_	− 149.7

### Vibrational frequencies

We now turn to examine the rigidity of the carbon scaffolds of the nuggets, that is, how they would vary from being hard and inflexible to soft and malleable as the number of vertices (carbon atoms) increases. We regard rigidity as a desirable property in a constrained geometry polyhedral compound, contributing to its structural stability and to other properties such as less susceptibility to thermal relaxation of excited states. Accordingly, in this work, we use the lowest calculated vibrational frequency of each nugget as a measure of its rigidity, the larger this frequency, the more rigid the compound. Indeed, the lowest frequency vibration, generally corresponds to a collective movement of all atoms of the molecule, fluttering in a synchronized manner along the corresponding normal coordinate.

Table 3 shows frequency values for the lowest vibrational modes for each of the 18 nuggets, after geometry optimization, from ωB97XD/6-31G* density functional theory, DFT, calculations.

**Table 3:** DFT ωB97XD/6-31G* frequency values of the first vibrational mode of the 18 nuggets studied and a few other compounds for comparison purposes.

Compound	νω^B97XD/6-31G^* (cm^−1^)
Nugget_8_	628
Nugget_12_	394
Nugget_14_	328
Nugget_16_	335
Nugget_18_	355
Nugget_20a_	227
Nugget_20b_	242
Nugget_20c_	301
Nugget_22_	305
Nugget_24a_	372
Nugget_24b_	255
Nugget_24c_	172
Nugget_26a_	257
Nugget_26b_	264
Nugget_26c_	177
Nugget_28a_	263
Nugget_28b_	245
Nugget_28c_	143
Cyclobutadiene, C_4_H_4_	547
Cyclobutane, C_4_H_8_	175
n-Butane, C_4_H_10_	123
Benzene, C_6_H_6_	414
Cyclohexane, C_6_H_12_	232
n-Hexane, C_6_H_14_	74
n-Octacosane, C_28_H_58_	7

For comparison purposes, Table 3 also shows the lowest vibrational frequency of other compounds, where one can see that, as expected, cyclic compounds are generally more rigid than linear ones. Further, the presence of double bonds certainly increases rigidity in otherwise similar compounds.

Let us first consider the case of nugget_8_ (cubane, C_8_H_8_), which can be regarded as having been formed by two piled up cyclobutadienes. Cubane (ν^ωB97XD/6-31G*^ = 628 cm^−1^) is more rigid than a cyclobutadiene (ν^ωB97XD/6-31G*^ = 547 cm^−1^), indicating a sturdier structure. On the contrary, nugget_12_ (ν^ωB97XD/6-31G*^ = 394 cm^−1^), the [6]-prismane, which can be regarded as having been formed by two piled up benzene molecules, is actually more flexible than benzene, which has a ν^ωB97XD/6-31G*^ value of 414 cm^−1^. In general, it can be argued that the sturdier the structure, the more difficult it is for it to get disassembled. Accordingly, as previously discussed, nugget_12_ would probably easily self-dismantle into two benzene molecules.

If we consider all other nuggets, from nugget_14_ to nugget_28c_, one of them, nugget_24a_ stands out as being the most rigid, having a very large lowest ν^ωB97XD/6-31G*^ of 372 cm^−1^. Nugget_24a_ is certainly special, displaying a very symmetric structure. This points to a molecular structure with much more balanced forces in each atom than those of the other nuggets. This reinforces the possibility of its 3D expansion, as discussed above, as likely being a very stable carbon allotrope that will probably be found to exhibit unique physical properties.

All other nuggets display rigidities that are seemingly large enough to guarantee their structural stabilities. As one would expect, the more prolate ones (the “c” ones) are less rigid than the more spherical ones (the “a” ones).

Naturally, as the number of carbon atoms in their structures increases, the nuggets tend to become less and less rigid. Nevertheless, their rigidities are, of course, still larger by a large difference than those displayed by the n-alkanes, and even by the cyclic alkanes with the same number of carbon atoms. All of this points to the direction that they could all be synthesized, as the synthetically challenging cubane indeed has been^6^.

As rigid as they are, the nuggets can then be fused together to form even larger structures, generating an assortment of shapes and forms that can bring about regular and irregular solids, porous structures, etc., with many potential applications to materials science. To examine such possibilities, let us now turn to their energetic properties of fusing.

### Energetics of nugget-nugget face-fusion reactions

To be able to design novel 1D, 2D, and 3D-scaffolds from the set of nuggets considered in this article, let us now study the ability of these systems to perform face-fusion reactions. Because the nuggets present both square and hexagonal faces, their growths must occur via the fusion reactions of either two square or two hexagonal faces. However, not all these face-fusions may take place because some of the faces of these nuggets, mostly the hexagonal faces, are not exactly flat surfaces, but slightly skew polygons, whose vertices are not all coplanar. In such cases, for a fusion to occur, a requirement of spatial complementarity may not always be possible because the hexagonal faces tend to be all concave. On the other hand, square faces in these polyhedra are almost all invariably planar. Therefore, face-fusion reactions are generally predicted to occur more frequently through square faces, rather than via the usually more skewed hexagonal faces.

Let us first consider the most probable face-fusion reactions between two identical nuggets only. Of course, between two square faces, the fusions may occur in up to 4 different relative orientations of one face with respect to the other. Likewise, with hexagonal faces, the fusions may occur in up to 6 such different relative orientations, all leading to a huge number of possibilities. Table 4 shows the energies of reactions, one for each type of fusion (whenever possible) that displayed the least ωB97XD/6-31G* energy values of reaction for each pair of identical nuggets. Results on Table 4 indicate that while there are 18 square face fusions, the number of hexagonal face fusions possible is only 5. The values of energy of hexagonal face-fusion reactions range from − 185.5 kJ for nugget_24a_ to 638.8 kJ to nugget_12_, with the same numbers for square face fusion reactions ranging from − 80.2 kJ, for nugget_26b_, to + 427.4 kJ for nugget_8_, cubane. Although the larger the nugget, the more likely it is to display negative face-fusion energies of reaction, we notice an exception to this rule: among the 18 nuggets designed in this article, two identical molecules of the carbon voxel nugget_24a_ are predicted to perform hexagonal face-fusion reactions with the largest negative value of ΔE^ωB97XD/6-31G*^ = − 185.0 kJ. Therefore, of all nuggets studied, nugget_24a_ is predicted to exhibit the largest aptitude to be applied to growth as 1D, 2D, and 3D-scaffolds, especially when one considers its voxel characteristics.

**Table 4:** Energy values of the most stable fusion reactions between two identical nuggets, either via square faces releasing C_4_H_8_, or, whenever possible, via planar hexagonal faces releasing C_6_H_12_. All values were calculated by employing the level of calculation ωB97XD/6-31G*.

Type of nugget	Fusion reaction	ΔEω^B97XD^ (kJ)
Nugget_8_	2 C_8_H_8_ → C_12_H_8_ + C_4_H_8_	+ 427.4
Nugget_12_	2 C_12_H_12_ → C_20_H_16_ + C_4_H_8_	− 15.1
Nugget_12_	2 C_12_H_12_ → C_18_H_12_ + C_6_H_12_	+ 638.8
Nugget_14_	2 C_14_H_14_ → C_24_H_20_ + C_4_H_8_	+ 101.1
Nugget_16_	2 C_16_H_16_ → C_28_H_24_ + C_4_H_8_	− 21.3
Nugget_18_	2 C_18_H_18_ → C_32_H_28_ + C_4_H_8_	− 69.7
Nugget_18_	2 C_18_H_18_ → C_30_H_24_ + C_6_H_12_	− 128.4
Nugget_20a_	2 C_20_H_20_ → C_36_H_32_ + C_4_H_8_	− 34.3
Nugget_20b_	2 C_20_H_20_ → C_36_H_32_ + C_4_H_8_	+ 169.0
Nugget_20c_	2 C_20_H_20_—> C_36_H_32_ + C_4_H_8_	− 77.1
Nugget_22_	2 C_22_H_22_ → C_40_H_36_ + C_4_H_8_	− 71.3
Nugget_24a_	2 C_24_H_24_ → C_44_H_40_ + C_4_H_8_	+ 176.6
Nugget_24a_	2 C_24_H_24_ → C_42_H_36_ + C_6_H_12_	− 185.0
Nugget_24b_	2 C_24_H_24_ → C_44_H_40_ + C_4_H_8_	− 76.0
Nugget_24c_	2C_24_H_24_ → C_44_H_40_ + C_4_H_8_	− 35.3
Nugget_26a_	2 C_26_H_26_ → C_48_H_44_ + C_4_H_8_	− 47.8
Nugget_26b_	2 C_26_H_26_ → C_48_H_44_ + C_4_H_8_	− 80.2
Nugget_26c_	2 C_26_H_26_ → C_48_H_44_ + C_4_H_8_	+ 100.3
Nugget_28a_	2 C_28_H_28_ → C_52_H_48_ + C_4_H_8_	− 73.3
Nugget_28a_	2 C_28_H_28_ → C_50_H_44_ + C_6_H_12_	+ 115.2
Nugget_28b_	2 C_28_H_28_ → C_52_H_48_ + C_4_H_8_	− 10.2
Nugget_28b_	2 C_28_H_28_ → C_50_H_44_ + C_6_H_12_	− 43.6
Nugget_28c_	2 C_28_H_28_ → C_52_H_48_ + C_4_H_8_	− 38.3

### Growth of nuggets into patterns

Upon face-fusion reactions, nuggets can grow into either regular or irregular structures. Let us first consider possible fused compounds displaying structures with regular patterns.

**Figure 9:**
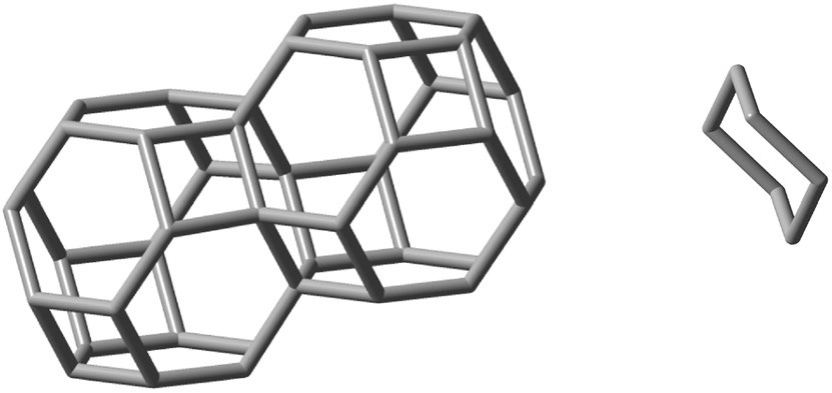
Left: Optimized geometry of the 1D-scaffold generator C_42_H_36_ obtained from the linear hexagonal face-fusion of nugget_24a_. Right: the released cyclohexane molecule. C_6_H_12_.

The simplest of these patterns are tessellations: covering of the space with nuggets, without overlaps or gaps. Tessellations can occur in one, two or three dimensions, and are the result of face-fusion reactions of a nugget, or of a combination of nuggets, made up by their translations, rotations or reflections. The carbon voxels, nugget_8_, nugget_12_ and nugget_24a_ would be natural candidates. However, as explained above, only nugget_24a_ would make such a chemically feasible tile for this purpose. Let us therefore turn to consider the growth of nugget_24a_ in 1 dimension. The idealized self-fusion reaction of two of them via one of its all-equivalent hexagonal faces, 2C_24_H_24_ → C_42_H_36_ + C_6_H_12_, ΔE^ωB97XD/6-31G*^ is − 185.0 kJ, where C_6_H_12_ refers to cyclohexane leads to a generator of the simplest 1D scaffold extension. Figure 9 shows its optimized geometry together with the released cyclohexane for easier visualization.

**Figure 10:**
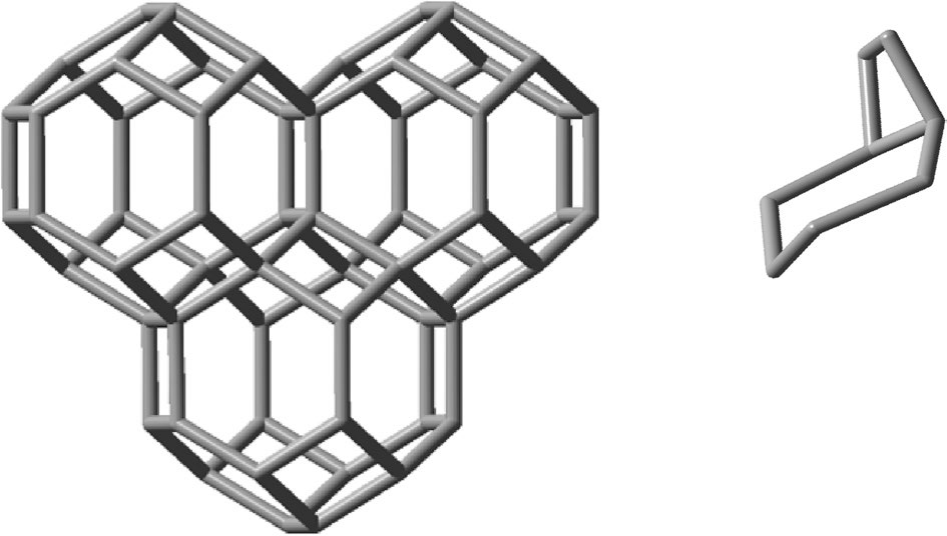
Left: optimized geometry of the C_58_H_46_ 2D-scaffold generator obtained by fusing three nugget_24a_ molecules. Right: the released (1R,6S)-bicyclo[4.2.0]octane molecule, C_8_H_14_, which is the product of the idealized second fusion reaction.

Next, to evaluate the ability of nugget_24a_ in generating 2D-scaffolds, the following idealized fusion reaction was now considered: C_24_H_24_ + C_42_H_36_ → C_58_H_46_ + C_8_H_14_, see Fig. 10 (left), where C_8_H_14_ is (1R,6S)-bicyclo[4.2.0]octane, Fig. 10 (right) and whose predicted energy of reaction is − 85.7 kJ. Due to its 2D-structural arrangement its stability is substantially more accentuated when compared with the formation of the essentially linear C_60_H_48_ 1D compound obtained by fusing together the 1d-generator compound in Fig. 9 with another nugget_24a_. This is because now a larger quantity of viable fusion reactions was carried out.

**Figure 11:**
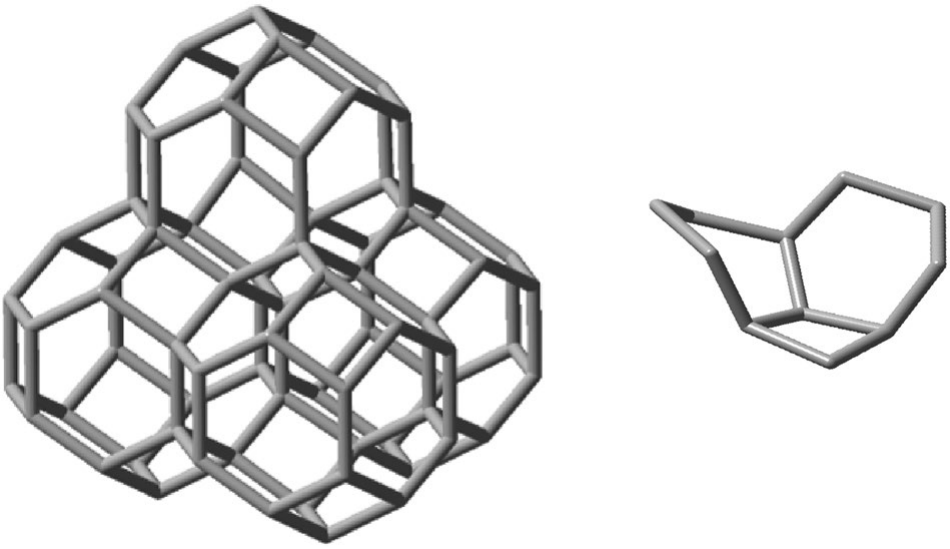
Left: optimized geometry of the C_71_H_52_ 3D-scaffold generator obtained from the growth of nugget_24a_. Right: the released (1s,1aS,4ar,7aR)-nonahydro-1H-cyclobuta[de]naphthalene molecule, C_11_H_18_, which is the product of the idealized third fusion reaction.

Finally, let us evaluate the ability of nugget_24a_ in generating 3D-scaffolds. The following idealized fusion reaction was considered: C_58_H_46_ + C_24_H_24_ → C_71_H_52_ + C_11_H_18_, see Fig. 11, where C_11_H_18_ stands for (1s,1aS,4ar,7aR)-nonahydro-1H-cyclobuta[de]naphthalene.

**Figure 12:**
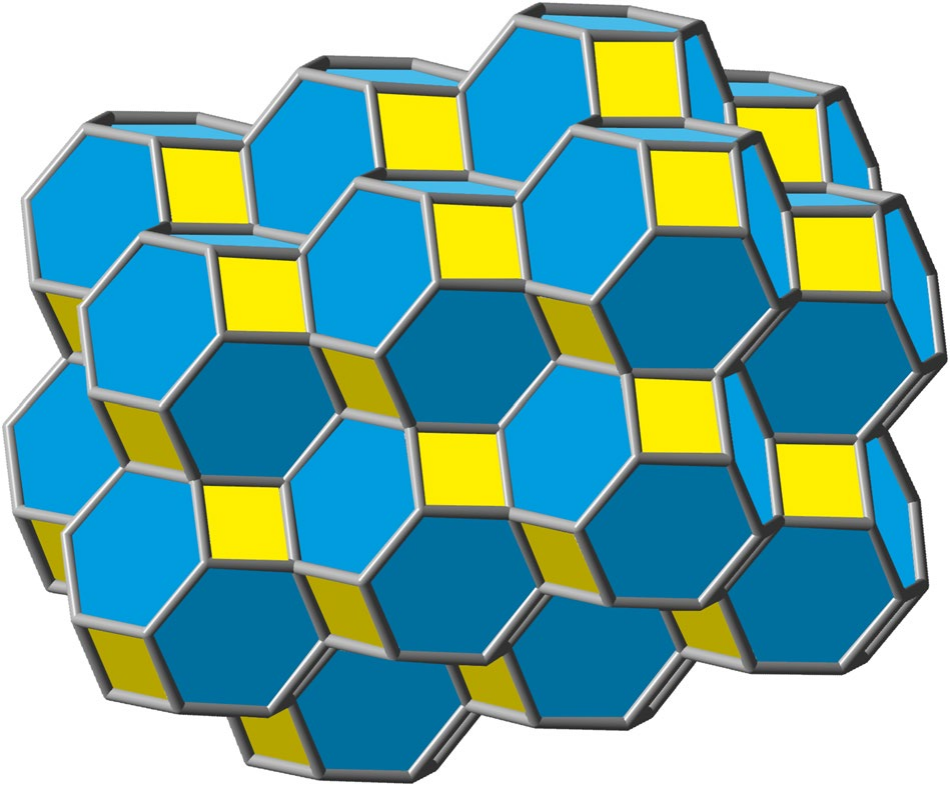
A solid view of the 3D carbon allotrope formed by fusions of several space filling carbon voxel nuggets_24a_ containing 252 carbon atoms.

The infinite 3D expansion of this polyhedron will lead to a carbon-only compound that would constitute an allotrope of carbon^42^. A solid model image of a piece of this allotrope can be seen in Fig. 12 below. It is noteworthy that, by acting as a space filling carbon voxel in this manner, at least in principle, nugget_24a_ could be employed to generate any 3D sculpture with itself as its finest granularity level.

**Figure 13:**
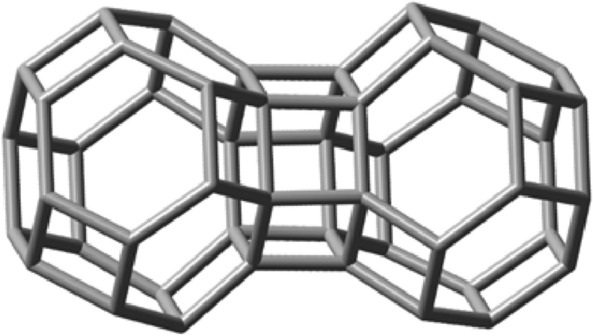
Compound C_48_H_42_ obtained by fusing together two nugget_24a_ compounds via a hexagonal prism.

Another seemingly rigid allotrope of carbon can also be made from nugget_24a_ in the form of a regular skew apeirohedron. Similarly, but not exactly like the one advanced by Zhou et al.^43^, this will be formed by joining the carbon voxels nugget_24a_ through hexagonal pyramidal bridges linking hexagonal faces of one to square faces of others, in a manner so that each external square face of the hexagonal prismatic bridge shares an edge with a square face of one of the polyhedra while its opposite edge is shared with a hexagonal face of the other. Figure 13 exemplifies such a hexagonal prismatic bridge between two nuggets_24a_. In this case, the idealized chemical reaction would be: 2C_24_H_24_ → C_48_H_36_ + 6H_2_. Indeed, according to our calculations (Table 4), these bridged connections of hexagonal faces are more energetically favorable than connections via square faces.

**Figure 14:**
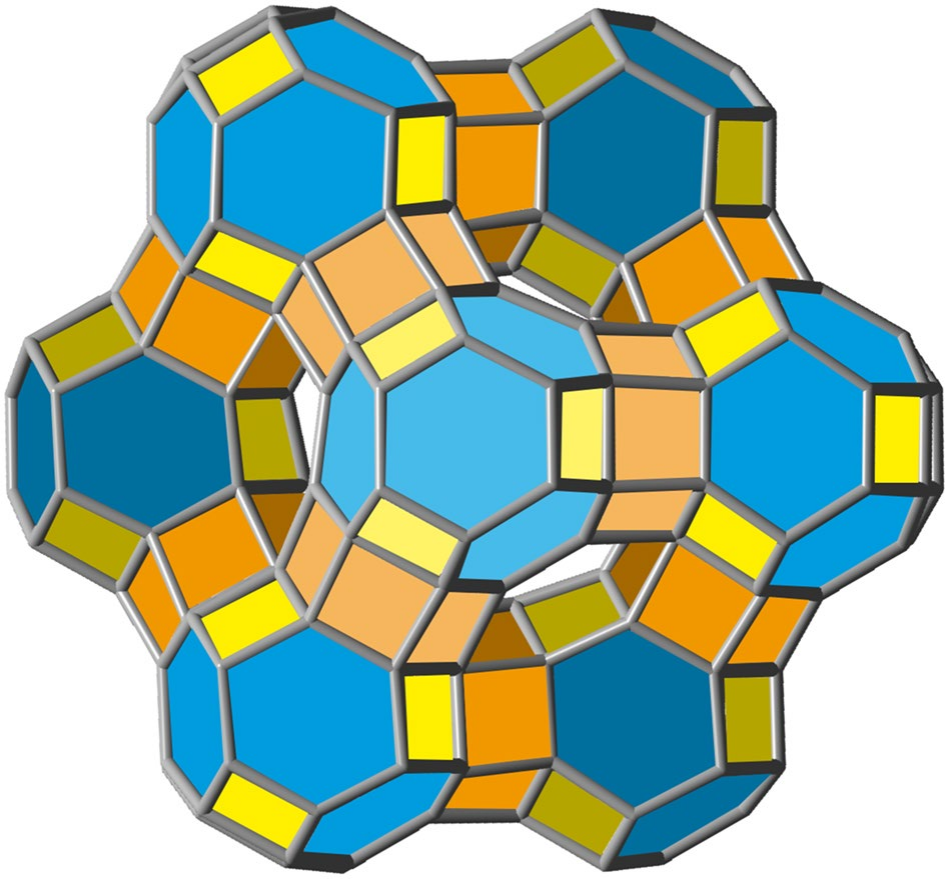
Solid view perspective of a section of the regular skew apeirohedron allotrope of carbon formed by fusions of nuggets_24a_ through their hexagonal faces via hexagonal prisms. In this figure, there are 10 fused nuggets_24a_ with 240 carbon atoms.

Therefore, the regular skew apeirohedron can then be formed by linking together*,* in this manner, each nugget_24a_ by 4 of its 8 hexagonal faces according to Fig. 14 below^44^. This putative allotrope of carbon, adding to previous exotic carbon allotropes^45^, would be very stable and rigid. Its density, however, would be evidently smaller than that of the space filling allotrope shown in Fig. 12. The presence of zeolite-like nanoporous cavities inside its structure could be a singular feature, that could perhaps prove to be the origin of many emerging and interesting properties.

**Figure 15:**
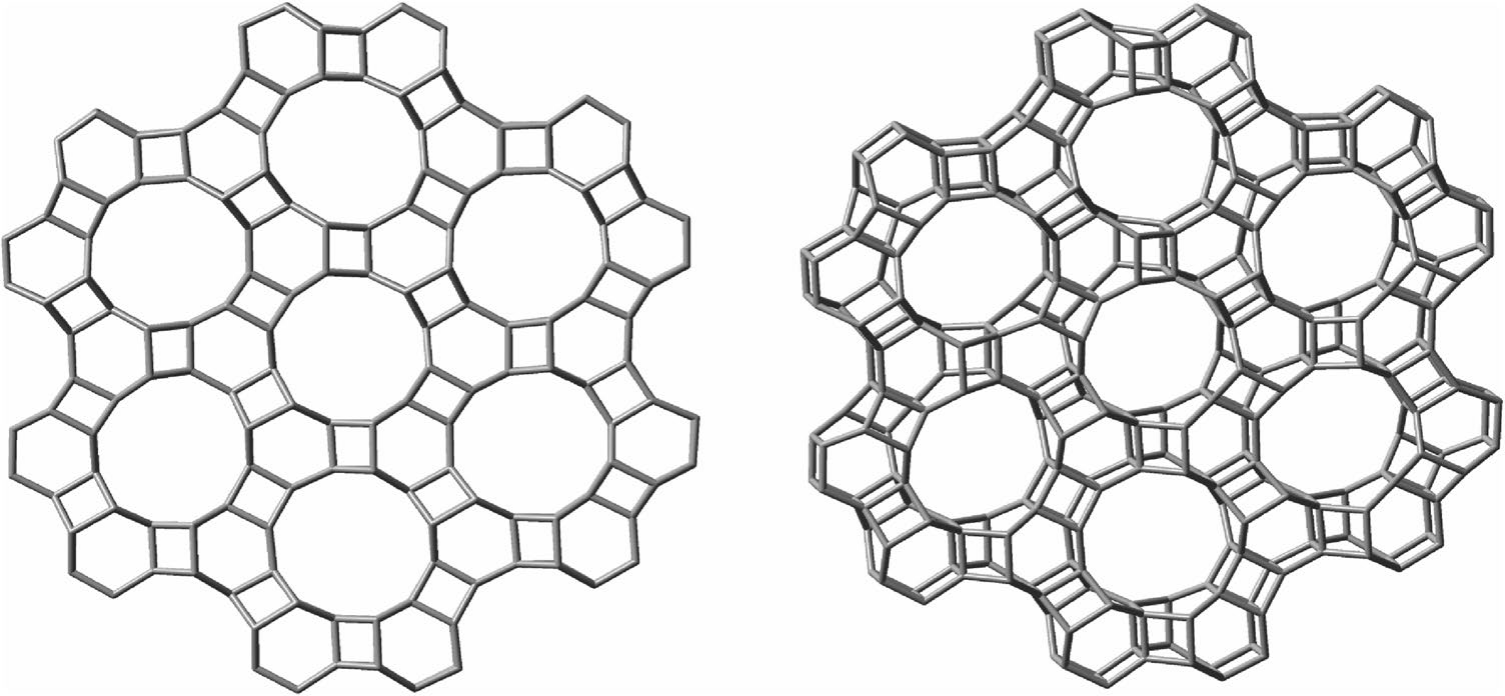
Two perspectives of compound C_288_H_144_ obtained by square face-fusions of 24 units of nugget_16_.

Other types of polyhedral solids, with larger cavities, can also be conceptualized, such as the one made, this time by nugget_16_, via square face-fusions, and whose projection in one plane reveals a semiregular or Archimedean tessellation, that can be grown indefinitely Fig. 15. Such a compound, if ever obtained, would also likely behave as a load resisting skeleton due to its symmetric nature. Furthermore, this structure could also be grown in 3D leading to lengthy tubular cavities that could prove eventually useful. Structures such as these, with large cavities in the middle, suggest applications to materials science as catalysts, porous powders, etc.

**Figure 16:**
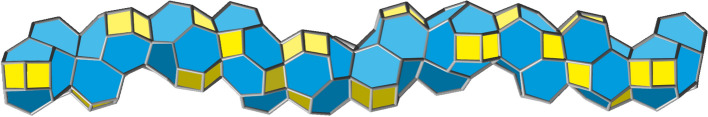
Perspective of a helix made by fusion of nugget_28b_ via its hexagonal face, of formula C_226_H_172_.

Many more combinations can be conceptualized by connecting the nuggets. Figure 16 shows a helix compound made by fusion of nugget_28b_ via two of its quasi-planar hexagonal faces. Such a compound, whose form resembles a twisted rope, would exhibit helicity, a form of chirality.

Besides, these regular and aesthetically appealing structures, several other large structures can be conceived by binding together several of the nuggets, leading to a myriad of hydrocarbon structures that would extend far beyond what is being here presented. The geometric possibilities of molecular structures that could in principle be formed based on these nuggets are truly vast: “symmetries, spirals, trees, waves, foams, tessellations, meanders, cracks, and stripes with fractal dimensions”^46^.

## Conclusions

Euler's theorem and topological strategies were employed in order to theoretically design a set of 18 hydrocarbon nuggets of general formula C_n_H_n_ containing four- and six-membered rings, that exist up to 28 vertexes. From Euler’s theorem we demonstrated that all such polyhedra must contain exactly six four-membered rings, for an arbitrary number of six-membered rings equal or greater than two. Among these 18 nuggets, 13 are novel systems, with 3 of them exhibiting polyhedral chirality.

We also showed that, with the exception of hexaprismane, which is predicted to easily self-dissociate into two benzene molecules, and therefore unlikely to be synthesizable; and also with the exception of nugget_18_, which is presumably expected to dissociate into three benzene molecules, all other nuggets are likely to be relatively stable and not self-dissociate or degrade.

Subsequently, vibrational properties revealed that the designed nuggets are sufficiently rigid. In this sense, the nuggets with 28 carbons are predicted to exhibit a structural rigidity, in average about 100 times greater than that of the linear alkane n-octacosane C_28_H_58_.

We also explored the expansions of these nuggets into larger structures by face-fusion reactions involving mainly hexagonal and sometimes square faces.

Nugget_24a_, the carbon voxel, resembles the most a fullerene (6 and 5-membered rings, however) in terms of the spherical shape, and possesses a chemical structure similar to the MOF ZIF-8. Due to its energetically favorable face-fusion reactions, Nugget_24a_ is deemed to be the most suitable one to have a large potential to be applied to growth as 1D, 2D, and 3D-scaffolds. Accordingly, any 3D sculpture could be generated with nugget_24a_ at its finest granularity level if sufficient synthetic control is one day discovered; or perhaps by carving from the innovative carbon allotrope presented in Fig. 11.

In conclusion, as mentioned in the previous section, the nuggets could be in principle expanded into all sorts of forms: “symmetries, spirals, trees, waves, foams, tessellations, meanders, cracks, and stripes of fractal dimensions”^46^. Their scaffolds may be decorated with strategically placed substituents as quantized perturbations, to promote attractive forces between them for a potential use in molecular tectonics. Perhaps they can form designer hyperstructures made layer by layer in a precisely chosen sequence where electronic or even exotic phenomena, typically requiring exceptionally low temperatures, can be explored. In summary, these are structures that should be considered as possibilities and of interest to researchers from all areas of carbonaceous nanomaterials (e.g., fullerene, nanotube, graphene, etc.). Finally, we also present the perspective of novel carbon allotropes, both space filled, as well as with cavities, hinting at interesting properties if synthesized or found as it appears to be the case with the natural, super-hard, and transparent crystalline polymorph of carbon from the Popigai impact crater in Russia, formed because of a natural shockwave event^41,42^.

## Supplementary information

Supplementary Information.
