# Supplementary material for: A theorized new class of polyhedral hydrocarbons of molecular formula CnHn and their bottom-up scaffold expansions into hyperstructures
Source: Sci Rep. 2021 Mar 10;11:5576. doi: 10.1038/s41598-021-84562-6 (PMC7946909; doi:10.1038/s41598-021-84562-6)
Supplement: Supplementary file 1 — Supplementary Information. [file 41598_2021_84562_MOESM1_ESM.pdf]

Supplementary Information

A theorized new class of polyhedral hydrocarbons of molecular formula C_nH_n and their bottom-up scaffold expansions into hyperstructures

Camila M. B. Machado[†], Nathalia B. D. Lima[†], Sóstenes L. S. Lins[§], and Alfredo M. Simas*[†]

[†]Departamento de Química Fundamental, CCEN, Universidade Federal de Pernambuco, 50740-560, Recife, Pernambuco, Brazil

[§]Centro de Informática, CIN, Universidade Federal de Pernambuco, 50670-901, Recife, Pernambuco, Brazil.

*simas@ufpe.br

List of Figures

Figure S1. Chemical structure of nugget$_8$ generated by the Blink software. Cartesian coordinates of its atoms; the first line contains the total charge and multiplicity; the following lines contain the atomic numbers, followed by the x, y, and z coordinates in Å for each one of the atoms. Next, atomic coordinates in Tripos Mol2 file format (.mol2) with the distances also in Å. 6

Figure S2. Chemical structure of nugget$_{12}$ generated by the Blink software. Cartesian coordinates of its atoms; the first line contains the total charge and multiplicity; the following lines contain the atomic numbers, followed by the x, y, and z coordinates in Å for each one of the atoms. Next, atomic coordinates in Tripos Mol2 file format (.mol2) with the distances also in Å. 9

Figure S3. Chemical structure of nugget$_{14}$ generated by the Blink software. Cartesian coordinates of its atoms; the first line contains the total charge and multiplicity; the following lines contain the atomic numbers, followed by the x, y, and z coordinates in Å for each one of the atoms. Next, atomic coordinates in Tripos Mol2 file format (.mol2) with the distances also in Å. 12

Figure S4. Chemical structure of nugget$_{16}$ generated by the Blink software. Cartesian coordinates of its atoms; the first line contains the total charge and multiplicity; the following lines contain the atomic numbers, followed by the x, y, and z coordinates in Å for each one of the atoms. Next, atomic coordinates in Tripos Mol2 file format (.mol2) with the distances also in Å. 15

Figure S5. Chemical structure of nugget$_{18}$ generated by the Blink software. Cartesian coordinates of its atoms; the first line contains the total charge and multiplicity; the following lines contain the atomic numbers, followed by the x, y, and z coordinates in Å for each one of the atoms. Next, atomic coordinates in Tripos Mol2 file format (.mol2) with the distances also in Å. 18

Figure S6. Chemical structure of nugget$_{20a}$ generated by the Blink software. Cartesian coordinates of its atoms; the first line contains the total charge and multiplicity; the following lines contain the atomic numbers, followed by the x, y, and z coordinates in Å for each one of the atoms. Next, atomic coordinates in Tripos Mol2 file format (.mol2) with the distances also in Å. 22

Figure S7. Chemical structure of nugget$_{20b}$ generated by the Blink software. Cartesian coordinates of its atoms; the first line contains the total charge and multiplicity; the following lines contain the atomic numbers, followed by the x, y, and z coordinates in Å for each one of the atoms. Next, atomic coordinates in Tripos Mol2 file format (.mol2) with the distances also in Å. 26

Figure S8. Chemical structure of nugget$_{20c}$ generated by the Blink software. Cartesian coordinates of its atoms; the first line contains the total charge and multiplicity; the following lines contain the atomic numbers, followed by the x, y, and z coordinates in Å for each one of the atoms. Next, atomic coordinates in Tripos Mol2 file format (.mol2) with the distances also in Å. 30

Figure S9. Chemical structure of nugget$_{22}$ generated by the Blink software. Cartesian coordinates of its atoms; the first line contains the total charge and multiplicity; the following lines contain the atomic numbers, followed by the x, y, and z coordinates in Å for each one of the atoms. Next, atomic coordinates in Tripos Mol2 file format (.mol2) with the distances also in Å. 34

Figure S10. Chemical structure of $nugget_{24a}$ generated by the Blink software. Cartesian coordinates of its atoms; the first line contains the total charge and multiplicity; the following lines contain the atomic numbers, followed by the x, y, and z coordinates in Å for each one of the atoms. Next, atomic coordinates in Tripos Mol2 file format (.mol2) with the distances also in Å. 38

Figure S11. Chemical structure of $nugget_{24b}$ generated by the Blink software. Cartesian coordinates of its atoms; the first line contains the total charge and multiplicity; the following lines contain the atomic numbers, followed by the x, y, and z coordinates in Å for each one of the atoms. Next, atomic coordinates in Tripos Mol2 file format (.mol2) with the distances also in Å. 42

Figure S12. Chemical structure of $nugget_{24c}$ generated by the Blink software. Cartesian coordinates of its atoms; the first line contains the total charge and multiplicity; the following lines contain the atomic numbers, followed by the x, y, and z coordinates in Å for each one of the atoms. Next, atomic coordinates in Tripos Mol2 file format (.mol2) with the distances also in Å. 46

Figure S13. Chemical structure of $nugget_{26a}$ generated by the Blink software. Cartesian coordinates of its atoms; the first line contains the total charge and multiplicity; the following lines contain the atomic numbers, followed by the x, y, and z coordinates in Å for each one of the atoms. Next, atomic coordinates in Tripos Mol2 file format (.mol2) with the distances also in Å. 50

Figure S14. Chemical structure of $nugget_{26b}$ generated by the Blink software. Cartesian coordinates of its atoms; the first line contains the total charge and multiplicity; the following lines contain the atomic numbers, followed by the x, y, and z coordinates in Å for each one of the atoms. Next, atomic coordinates in Tripos Mol2 file format (.mol2) with the distances also in Å. 54

Figure S15. Chemical structure of $nugget_{26c}$ generated by the Blink software. Cartesian coordinates of its atoms; the first line contains the total charge and multiplicity; the following lines contain the atomic numbers, followed by the x, y, and z coordinates in Å for each one of the atoms. Next, atomic coordinates in Tripos Mol2 file format (.mol2) with the distances also in Å. 58

Figure S16. Chemical structure of $nugget_{28a}$ generated by the Blink software. Cartesian coordinates of its atoms; the first line contains the total charge and multiplicity; the following lines contain the atomic numbers, followed by the x, y, and z coordinates in Å for each one of the atoms. Next, atomic coordinates in Tripos Mol2 file format (.mol2) with the distances also in Å. 63

Figure S17. Chemical structure of $nugget_{28b}$ generated by the Blink software. Cartesian coordinates of its atoms; the first line contains the total charge and multiplicity; the following lines contain the atomic numbers, followed by the x, y, and z coordinates in Å for each one of the atoms. Next, atomic coordinates in Tripos Mol2 file format (.mol2) with the distances also in Å. 68

Figure S18. Chemical structure of $nugget_{28c}$ generated by the Blink software. Cartesian coordinates of its atoms; the first line contains the total charge and multiplicity; the following lines contain the atomic numbers, followed by the x, y, and z coordinates in Å for each one of the atoms. Next, atomic coordinates in Tripos Mol2 file format (.mol2) with the distances also in Å. 73

Figure S19. DFT ωB97XD/6-31G* optimized geometries of the following pairs of chiral nuggets: (a) nugget$_{24b}$ ($C_{24}H_{24}$); (b) nugget$_{26b}$ ($C_{26}H_{26}$); and (c) nugget$_{28b}$ ($C_{28}H_{28}$). Cartesian coordinates of its atoms; the first line contains the total charge and multiplicity; the following lines contain the atomic numbers, followed by the x, y, and z coordinates in Å for each one of the atoms. Next, atomic coordinates in Tripos Mol2 file format (.mol2) with the distances also in Å. 97

Figure S20. Left: Optimized geometry of the 1D-scaffold generator $C_{42}H_{36}$ obtained from the linear hexagonal face-fusion of nugget$_{24a}$. Right: the released cyclohexane molecule. Cartesian coordinates of its atoms; the first line contains the total charge and multiplicity; the following lines contain the atomic numbers, followed by the x, y, and z coordinates in Å for each one of the atoms. Next, atomic coordinates in Tripos Mol2 file format (.mol2) with the distances also in Å. 104

Figure S21. Left: optimized geometry of the $C_{58}H_{46}$ 2D-scaffold generator obtained by fusing three nugget$_{24a}$ molecules. Right: the released (1R,6S)-bicyclo[4.2.0]octane molecule, C_8H_{14}. Cartesian coordinates of its atoms; the first line contains the total charge and multiplicity; the following lines contain the atomic numbers, followed by the x, y, and z coordinates in Å for each one of the atoms. Next, atomic coordinates in Tripos Mol2 file format (.mol2) with the distances also in Å. 112

Figure S22. Left: optimized geometry of the $C_{71}H_{52}$ 3D-scaffold generator obtained from the growth of nugget$_{24a}$. Right: the released (1s,1aS,4ar,7aR)-nonahydro-1H-cyclobuta[de]naphthalene molecule, $C_{11}H_{18}$, which is the product of the idealized third fusion reaction. Cartesian coordinates of its atoms; the first line contains the total charge and multiplicity; the following lines contain the atomic numbers, followed by the x, y, and z coordinates in Å for each one of the atoms. Next, atomic coordinates in Tripos Mol2 file format (.mol2) with the distances also in Å. 122

Figure S23. A solid view of the 3D carbon allotrope formed by fusions of several space filling carbon voxel nugget$_{24a}$s containing 252 carbon atoms. Cartesian coordinates of its atoms; the first line contains the total charge and multiplicity; the following lines contain the atomic numbers, followed by the x, y, and z coordinates in Å for each one of the atoms. Next, atomic coordinates in Tripos Mol2 file format (.mol2) with the distances also in Å. 151

Figure S24. Compound $C_{48}H_{42}$ obtained by fusing together two nugget$_{24a}$ compounds via a hexagonal prism. Cartesian coordinates of its atoms; the first line contains the total charge and multiplicity; the following lines contain the atomic numbers, followed by the x, y, and z coordinates in Å for each one of the atoms. Next, atomic coordinates in Tripos Mol2 file format (.mol2) with the distances also in Å. 158

Figure S25. Solid view perspective of a section of the regular skew apeirohedron allotrope of carbon formed by fusions of nugget$_{24a}$s through their hexagonal faces via hexagonal prisms. In this figure, there are 10 fused nuggets$_{24a}$ with 240 carbon atoms. Cartesian coordinates of its atoms; the first line contains the total charge and multiplicity; the following lines contain the atomic numbers, followed by the x, y, and z coordinates in Å for each one of the atoms. Next, atomic coordinates in Tripos Mol2 file format (.mol2) with the distances also in Å. 184

Figure S26. Two perspectives of compound $C_{288}H_{144}$ obtained by square face-fusions of 24 units of nugget$_{16}$. Cartesian coordinates of its atoms; the first line contains the total charge and multiplicity; the following lines contain the atomic numbers, followed by the

x, y, and z coordinates in Å for each one of the atoms. Next, atomic coordinates in Tripos Mol2 file format (.mol2) with the distances also in Å... 216

Figure S27. Perspective of a helix made by fusion of $nuggets_{28b}$ via its hexagonal face, of formula $C_{226}H_{172}$. Cartesian coordinates of its atoms; the first line contains the total charge and multiplicity; the following lines contain the atomic numbers, followed by the x, y, and z coordinates in Å for each one of the atoms. Next, atomic coordinates in Tripos Mol2 file format (.mol2) with the distances also in Å... 244

Nugget$_8$ (C_8H_8)

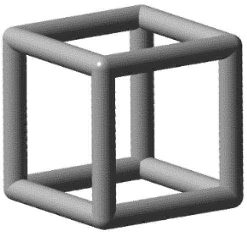

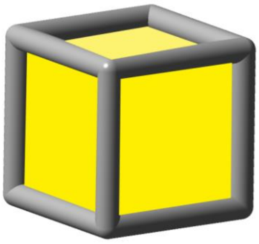

Cartesian Coordinates (Å)

0 1

6	-1.244	-0.533	0.040
6	-0.408	-0.289	-1.258
6	-0.854	0.890	0.558
6	-0.018	1.133	-0.740
6	0.018	-1.133	0.740
6	0.408	0.289	1.258
6	0.854	-0.890	-0.559
6	1.244	0.532	-0.040
1	-2.246	-0.962	0.073
1	-0.737	-0.521	-2.271
1	-1.542	1.607	1.008
1	-0.032	2.046	-1.336
1	0.033	-2.046	1.336
1	0.737	0.522	2.271
1	1.542	-1.606	-1.008
1	2.246	0.962	-0.073

.mol2 file

@<TRIPOS>MOLECULE
Molecule Name
16 20 1 0 0
SMALL
NO_CHARGES
****
Generated from the CSD
@<TRIPOS>ATOM

1	C1	-1.2440	-0.5326	0.0404	C.3	1 1	0.0000
2	C2	-0.4082	-0.2888	-1.2578	C.3	1 1	0.0000
3	C3	-0.8541	0.8898	0.5583	C.3	1 1	0.0000
4	C4	-0.0183	1.1333	-0.7398	C.3	1 1	0.0000
5	C5	0.0184	-1.1333	0.7398	C.3	1 1	0.0000
6	C6	0.4082	0.2888	1.2579	C.3	1 1	0.0000
7	C7	0.8539	-0.8898	-0.5585	C.3	1 1	0.0000
8	C8	1.2441	0.5325	-0.0403	C.3	1 1	0.0000
9	H9	-2.2457	-0.9624	0.0728	H	1 1	0.0000
10	H10	-0.7373	-0.5210	-2.2712	H	1 1	0.0000
11	H11	-1.5421	1.6065	1.0076	H	1 1	0.0000

```
   12 H12     -0.0323   2.0462  -1.3360   H         1 1      0.0000
   13 H13      0.0328  -2.0461   1.3362   H         1 1      0.0000
   14 H14      0.7369   0.5216   2.2713   H         1 1      0.0000
   15 H15      1.5420  -1.6064  -1.0078   H         1 1      0.0000
   16 H16      2.2457   0.9622  -0.0726   H         1 1      0.0000
@<TRIPOS>BOND
    1    1    2    1
    2    1    3    1
    3    1    5    1
    4    1    9    1
    5    2    4    1
    6    2    7    1
    7    2   10    1
    8    3    4    1
    9    3    6    1
   10    3   11    1
   11    4    8    1
   12    4   12    1
   13    5    6    1
   14    5    7    1
   15    5   13    1
   16    6    8    1
   17    6   14    1
   18    7    8    1
   19    7   15    1
   20    8   16    1
@<TRIPOS>SUBSTRUCTURE
    1 RES1       1 GROUP             0 ****  ****    0
```

Figure S1. Chemical structure of nugget$_8$ generated by the Blink software. Cartesian coordinates of its atoms; the first line contains the total charge and multiplicity; the following lines contain the atomic numbers, followed by the x, y, and z coordinates in Å for each one of the atoms. Next, atomic coordinates in Tripos Mol2 file format (.mol2) with the distances also in Å.

Nugget$_{12}$ ($C_{12}H_{12}$)

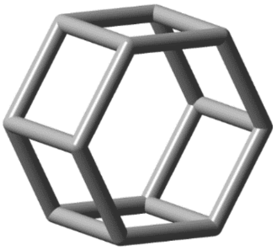
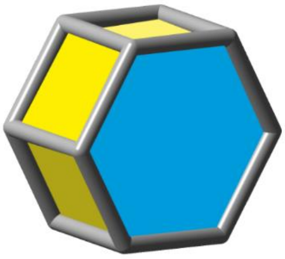

Cartesian Coordinates (Å)

0 1

6	-3.867	0.774	0.780
6	-3.867	0.774	-0.780
6	-2.480	1.459	0.779
6	-2.480	1.459	-0.779
1	-2.541	2.480	-1.171
1	-4.652	1.253	1.369
1	-2.541	2.480	1.171
1	-4.652	1.253	-1.369
6	-3.867	-0.774	-0.780
1	-4.652	-1.253	-1.369
6	-3.867	-0.774	0.780
1	-4.652	-1.253	1.369
6	-2.480	-1.459	-0.779
1	-2.541	-2.480	-1.171
6	-2.480	-1.459	0.779
1	-2.541	-2.480	1.171
6	-1.271	-0.780	1.420
1	-1.252	-1.082	2.475
6	-1.271	0.780	1.420
1	-1.252	1.082	2.475
6	-1.271	0.780	-1.420
1	-1.252	1.082	-2.475
6	-1.271	-0.780	-1.420
1	-1.252	-1.082	-2.475
6	0.000	-1.381	-0.787
1	0.000	-2.440	-1.082
6	0.000	-1.381	0.787
1	0.000	-2.440	1.082
6	0.000	1.381	0.787
1	0.000	2.440	1.082
6	0.000	1.381	-0.787
1	0.000	2.440	-1.082
6	1.271	-0.780	1.420
1	1.252	-1.082	2.475
6	1.271	0.780	1.420
1	1.252	1.082	2.475
6	1.271	0.780	-1.420
1	1.252	1.082	-2.475

6	1.271	-0.780	-1.420
1	1.252	-1.082	-2.475
6	2.480	1.459	0.779
1	2.541	2.480	1.171
6	2.480	1.459	-0.779
1	2.541	2.480	-1.171
6	2.480	-1.459	-0.779
1	2.541	-2.480	-1.171
6	2.480	-1.459	0.779
1	2.541	-2.480	1.171
6	3.867	0.774	0.780
1	4.652	1.253	1.369
6	3.867	-0.774	0.780
1	4.652	-1.253	1.369
6	3.867	-0.774	-0.780
1	4.652	-1.253	-1.369
6	3.867	0.774	-0.780
1	4.652	1.253	-1.369

.mol2 file

```
@<TRIPOS>MOLECULE
Molecule Name
24   30    1    0    0
SMALL
NO_CHARGES
****
Generated from the CSD

@<TRIPOS>ATOM
1 H1       2.4390  -0.4753  -1.3507   H        1 1      0.0000
2 C2       1.5252  -0.2973  -0.7794   C.3      1 1      0.0000
3 C3      -0.5049   1.4687  -0.7793   C.3      1 1      0.0000
4 C4      -1.0199  -1.1717  -0.7793   C.3      1 1      0.0000
5 C5      -1.5252   0.2973  -0.7794   C.3      1 1      0.0000
6 C6       0.5049  -1.4687  -0.7793   C.3      1 1      0.0000
7 C7       1.0199   1.1716  -0.7793   C.3      1 1      0.0000
8 H8      -2.4390   0.4753  -1.3506   H        1 1      0.0000
9 H9       0.8079  -2.3496  -1.3498   H        1 1      0.0000
10 H10     1.6312   1.8746  -1.3497   H        1 1      0.0000
11 H11    -0.8078   2.3496  -1.3499   H        1 1      0.0000
12 H12    -1.6312  -1.8745  -1.3498   H        1 1      0.0000
13 C13     1.5252  -0.2973   0.7794   C.3      1 1      0.0000
14 H14     2.4390  -0.4753   1.3506   H        1 1      0.0000
15 C15     1.0199   1.1716   0.7793   C.3      1 1      0.0000
16 H16     1.6312   1.8746   1.3497   H        1 1      0.0000
17 C17    -0.5049   1.4687   0.7793   C.3      1 1      0.0000
18 H18    -0.8079   2.3495   1.3499   H        1 1      0.0000
19 C19    -1.5252   0.2973   0.7793   C.3      1 1      0.0000
20 H20    -2.4390   0.4754   1.3506   H        1 1      0.0000
```

```
21 C21     -1.0199  -1.1716   0.7793   C.3       1 1      0.0000
22 H22     -1.6312  -1.8746   1.3498   H         1 1      0.0000
23 C23      0.5049  -1.4687   0.7793   C.3       1 1      0.0000
24 H24      0.8078  -2.3495   1.3499   H         1 1      0.0000
@<TRIPOS>BOND
1    1    2    1
2    2    6    1
3    2    7    1
4    2   13    1
5    3    5    1
6    3    7    1
7    3   11    1
8    3   17    1
9    4    5    1
10    4    6    1
11    4   12    1
12    4   21    1
13    5    8    1
14    5   19    1
15    6    9    1
16    6   23    1
17    7   10    1
18    7   15    1
19   13   14    1
20   13   15    1
21   13   23    1
22   15   16    1
23   15   17    1
24   17   18    1
25   17   19    1
26   19   20    1
27   19   21    1
28   21   22    1
29   21   23    1
30   23   24    1
@<TRIPOS>SUBSTRUCTURE
1 RES1       1 GROUP            0 ****  ****    0
```

Figure S2. Chemical structure of nugget$_{12}$ generated by the Blink software. Cartesian coordinates of its atoms; the first line contains the total charge and multiplicity; the following lines contain the atomic numbers, followed by the x, y, and z coordinates in Å for each one of the atoms. Next, atomic coordinates in Tripos Mol2 file format (.mol2) with the distances also in Å.

Nugget$_{14}$ ($C_{14}H_{14}$)

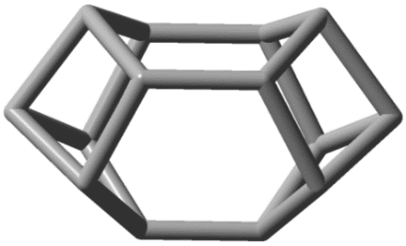

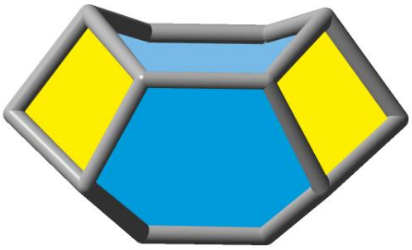

Cartesian Coordinates (Å)

0 1

6	2.391	0.000	0.000
6	1.471	-1.254	0.112
6	1.471	0.724	1.030
6	0.767	-0.622	1.340
1	1.176	-1.050	2.260
1	3.480	-0.000	0.000
1	1.820	1.319	1.877
1	1.820	-2.285	0.203
6	1.471	0.530	-1.142
1	1.820	0.966	-2.081
6	0.767	-0.849	-1.209
1	1.177	-1.432	-2.039
6	0.767	1.471	-0.131
1	1.176	2.482	-0.221
6	-0.767	-0.622	1.340
1	-1.176	-1.050	2.260
6	-1.471	-1.254	0.112
1	-1.820	-2.285	0.203
6	-0.767	-0.849	-1.209
1	-1.177	-1.432	-2.039
6	-0.767	1.471	-0.131
1	-1.176	2.482	-0.221
6	-1.471	0.724	1.030
1	-1.820	1.319	1.877
6	-2.391	0.000	0.000
1	-3.480	-0.000	0.000
6	-1.471	0.530	-1.142
1	-1.820	0.966	-2.081

.mol2 file

@<TRIPOS>MOLECULE
Molecule Name
28 35 1 0 0
SMALL
NO_CHARGES
****
Generated from the CSD

@<TRIPOS>ATOM
1 C1 2.3910 0.0000 0.0000 C.3 1 1 0.0000
2 C2 1.4709 -1.2543 0.1117 C.3 1 1 0.0000
3 C3 1.4710 0.7238 1.0303 C.3 1 1 0.0000
4 C4 0.7668 -0.6223 1.3397 C.3 1 1 0.0000
5 H5 1.1765 -1.0495 2.2601 H 1 1 0.0000
6 H6 3.4800 -0.0001 0.0000 H 1 1 0.0000
7 H7 1.8203 1.3188 1.8774 H 1 1 0.0000
8 H8 1.8201 -2.2855 0.2031 H 1 1 0.0000
9 C9 1.4711 0.5305 -1.1420 C.3 1 1 0.0000
10 H10 1.8204 0.9663 -2.0811 H 1 1 0.0000
11 C11 0.7668 -0.8490 -1.2087 C.3 1 1 0.0000
12 H12 1.1769 -1.4324 -2.0389 H 1 1 0.0000
13 C13 0.7668 1.4714 -0.1310 C.3 1 1 0.0000
14 H14 1.1761 2.4824 -0.2205 H 1 1 0.0000
15 C15 -0.7668 -0.6223 1.3397 C.3 1 1 0.0000
16 H16 -1.1765 -1.0495 2.2601 H 1 1 0.0000
17 C17 -1.4709 -1.2543 0.1117 C.3 1 1 0.0000
18 H18 -1.8201 -2.2855 0.2031 H 1 1 0.0000
19 C19 -0.7668 -0.8490 -1.2087 C.3 1 1 0.0000
20 H20 -1.1769 -1.4324 -2.0389 H 1 1 0.0000
21 C21 -0.7668 1.4714 -0.1310 C.3 1 1 0.0000
22 H22 -1.1761 2.4824 -0.2205 H 1 1 0.0000
23 C23 -1.4710 0.7238 1.0303 C.3 1 1 0.0000
24 H24 -1.8203 1.3188 1.8774 H 1 1 0.0000
25 C25 -2.3910 0.0000 0.0000 C.3 1 1 0.0000
26 H26 -3.4800 -0.0001 0.0000 H 1 1 0.0000
27 C27 -1.4711 0.5305 -1.1420 C.3 1 1 0.0000
28 H28 -1.8203 0.9663 -2.0811 H 1 1 0.0000
@<TRIPOS>BOND
1 1 2 1
2 1 3 1
3 1 6 1
4 1 9 1
5 2 4 1
6 2 8 1
7 2 11 1
8 3 4 1
9 3 7 1
10 3 13 1
11 4 5 1
12 4 15 1
13 9 10 1
14 9 11 1
15 9 13 1
16 11 12 1
17 11 19 1
18 13 14 1
19 13 21 1

```
20    15    16    1
21    15    17    1
22    15    23    1
23    17    18    1
24    17    19    1
25    17    25    1
26    19    20    1
27    19    27    1
28    21    22    1
29    21    23    1
30    21    27    1
31    23    24    1
32    23    25    1
33    25    26    1
34    25    27    1
35    27    28    1
@<TRIPOS>SUBSTRUCTURE
1 RES1       1 GROUP            0 ****  ****    0
```

Figure S3. Chemical structure of $nugget_{14}$ generated by the Blink software. Cartesian coordinates of its atoms; the first line contains the total charge and multiplicity; the following lines contain the atomic numbers, followed by the x, y, and z coordinates in Å for each one of the atoms. Next, atomic coordinates in Tripos Mol2 file format (.mol2) with the distances also in Å.

Nugget$_{16}$ ($C_{16}H_{16}$)

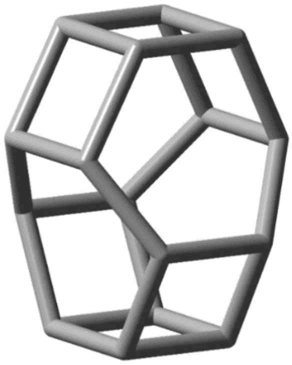

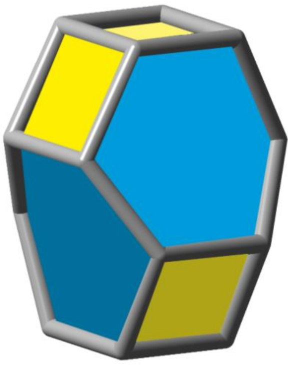

Cartesian Coordinates (Å)

0 1

6	1.956	1.076	0.228
6	1.956	0.225	-1.077
6	1.956	-0.225	1.077
6	1.956	-1.076	-0.228
1	2.747	1.791	0.464
1	2.749	0.297	-1.825
6	0.574	0.816	-1.467
1	0.655	1.467	-2.343
6	0.574	1.671	-0.158
1	0.656	2.735	-0.403
6	0.574	-0.816	1.467
6	0.574	-1.671	0.158
6	-0.574	1.467	0.816
1	-0.656	2.343	1.467
6	-0.574	0.158	1.671
1	-0.656	0.403	2.735
6	-0.574	-1.467	-0.816
1	-0.655	-2.343	-1.467
6	-0.574	-0.158	-1.671
1	-0.656	-0.403	-2.735
6	-1.956	0.228	-1.076
1	-2.747	0.464	-1.791
6	-1.956	1.077	0.225
1	-2.749	1.825	0.297
6	-1.956	-1.077	-0.225
1	-2.749	-1.825	-0.297
6	-1.956	-0.228	1.076
1	-2.747	-0.464	1.791
1	2.749	-0.297	1.825
1	2.747	-1.791	-0.464
1	0.656	-1.467	2.343
1	0.656	-2.735	0.403

.mol2 file

```
@<TRIPOS>MOLECULE
Molecule Name
   32    40     1     0     0
SMALL
NO_CHARGES
****
Generated from the CSD

@<TRIPOS>ATOM
     1 C1       1.9558   1.0763   0.2275   C.3       1 1      0.0000
     2 C2       1.9562   0.2251  -1.0765   C.3       1 1      0.0000
     3 C3       1.9562  -0.2251   1.0765   C.3       1 1      0.0000
     4 C4       1.9558  -1.0763  -0.2276   C.3       1 1      0.0000
     5 H5       2.7473   1.7913   0.4640   H         1 1      0.0000
     6 H6       2.7487   0.2967  -1.8252   H         1 1      0.0000
     7 C7       0.5740   0.8157  -1.4670   C.3       1 1      0.0000
     8 H8       0.6555   1.4666  -2.3431   H         1 1      0.0000
     9 C9       0.5737   1.6709  -0.1582   C.3       1 1      0.0000
    10 H10      0.6558   2.7346  -0.4027   H         1 1      0.0000
    11 C11      0.5740  -0.8157   1.4670   C.3       1 1      0.0000
    12 C12      0.5738  -1.6709   0.1582   C.3       1 1      0.0000
    13 C13     -0.5740   1.4671   0.8157   C.3       1 1      0.0000
    14 H14     -0.6556   2.3431   1.4666   H         1 1      0.0000
    15 C15     -0.5737   0.1582   1.6709   C.3       1 1      0.0000
    16 H16     -0.6557   0.4027   2.7346   H         1 1      0.0000
    17 C17     -0.5740  -1.4671  -0.8157   C.3       1 1      0.0000
    18 H18     -0.6555  -2.3431  -1.4666   H         1 1      0.0000
    19 C19     -0.5738  -0.1582  -1.6709   C.3       1 1      0.0000
    20 H20     -0.6558  -0.4027  -2.7345   H         1 1      0.0000
    21 C21     -1.9558   0.2275  -1.0763   C.3       1 1      0.0000
    22 H22     -2.7473   0.4639  -1.7913   H         1 1      0.0000
    23 C23     -1.9562   1.0765   0.2251   C.3       1 1      0.0000
    24 H24     -2.7487   1.8252   0.2966   H         1 1      0.0000
    25 C25     -1.9562  -1.0766  -0.2251   C.3       1 1      0.0000
    26 H26     -2.7487  -1.8252  -0.2966   H         1 1      0.0000
    27 C27     -1.9558  -0.2275   1.0764   C.3       1 1      0.0000
    28 H28     -2.7473  -0.4640   1.7913   H         1 1      0.0000
    29 H29      2.7488  -0.2967   1.8251   H         1 1      0.0000
    30 H30      2.7473  -1.7913  -0.4640   H         1 1      0.0000
    31 H31      0.6556  -1.4666   2.3431   H         1 1      0.0000
    32 H32      0.6558  -2.7346   0.4027   H         1 1      0.0000
@<TRIPOS>BOND
     1     1     2    1
     2     1     3    1
     3     1     5    1
     4     1     9    1
     5     2     4    1
     6     2     6    1
     7     2     7    1
```

```
    8     3     4    1
    9     3    11    1
   10     3    29    1
   11     4    12    1
   12     4    30    1
   13     7     8    1
   14     7     9    1
   15     7    19    1
   16     9    10    1
   17     9    13    1
   18    11    12    1
   19    11    15    1
   20    11    31    1
   21    12    17    1
   22    12    32    1
   23    13    14    1
   24    13    15    1
   25    13    23    1
   26    15    16    1
   27    15    27    1
   28    17    18    1
   29    17    19    1
   30    17    25    1
   31    19    20    1
   32    19    21    1
   33    21    22    1
   34    21    23    1
   35    21    25    1
   36    23    24    1
   37    23    27    1
   38    25    26    1
   39    25    27    1
   40    27    28    1
@<TRIPOS>SUBSTRUCTURE
     1 RES1       1 GROUP            0 ****  ****    0
```

Figure S4. Chemical structure of $nugget_{16}$ generated by the Blink software. Cartesian coordinates of its atoms; the first line contains the total charge and multiplicity; the following lines contain the atomic numbers, followed by the x, y, and z coordinates in Å for each one of the atoms. Next, atomic coordinates in Tripos Mol2 file format (.mol2) with the distances also in Å.

Nugget18 ($C_{18}H_{18}$)

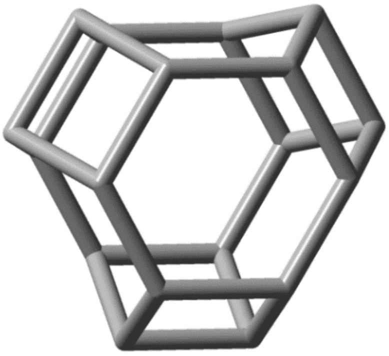

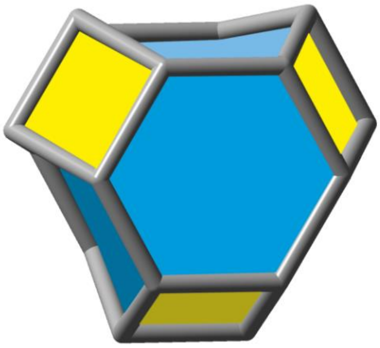

Cartesian Coordinates (Å)

0 1

6	-1.002	1.182	-1.305
6	0.546	1.450	-1.305
6	-1.136	2.003	0.000
6	0.397	2.268	0.000
1	0.840	3.266	0.000
1	-1.457	1.728	-2.136
1	-1.888	2.795	0.000
1	0.792	2.118	-2.136
6	0.546	1.450	1.305
1	0.792	2.118	2.136
6	-1.002	1.182	1.305
1	-1.457	1.728	2.137
6	-1.529	-0.252	-1.305
1	-2.230	-0.373	-2.136
6	-2.162	-0.790	0.000
1	-3.249	-0.906	0.000
6	-1.529	-0.252	1.305
1	-2.230	-0.373	2.136
6	1.525	0.277	1.305
1	2.226	0.398	2.136
6	2.302	-0.017	0.000
1	3.364	0.238	0.000
6	1.525	0.277	-1.305
1	2.226	0.398	-2.136
6	0.983	-1.198	-1.305
1	1.438	-1.744	-2.137
6	-0.523	-1.459	-1.305
1	-0.768	-2.127	-2.136
6	-0.523	-1.459	1.305
1	-0.768	-2.127	2.136
6	0.983	-1.198	1.305
1	1.438	-1.744	2.137
6	-1.167	-1.985	0.000
1	-1.476	-3.032	0.000
6	1.766	-1.478	0.000

1 2.409 -2.360 0.000

.mol2 file

```
@<TRIPOS>MOLECULE
Molecule Name
36   45   1   0   0
SMALL
NO_CHARGES
****
Generated from the CSD

@<TRIPOS>ATOM
1 C1     -1.0020   1.1823  -1.3053   C.3     1 1    0.0000
2 C2      0.5463   1.4504  -1.3051   C.3     1 1    0.0000
3 C3     -1.1360   2.0026   0.0000   C.3     1 1    0.0000
4 C4      0.3965   2.2678   0.0000   C.3     1 1    0.0000
5 H5      0.8399   3.2664   0.0000   H       1 1    0.0000
6 H6     -1.4568   1.7280  -2.1365   H       1 1    0.0000
7 H7     -1.8884   2.7947   0.0000   H       1 1    0.0000
8 H8      0.7921   2.1179  -2.1356   H       1 1    0.0000
9 C9      0.5463   1.4504   1.3051   C.3     1 1    0.0000
10 H10    0.7921   2.1179   2.1355   H       1 1    0.0000
11 C11   -1.0020   1.1823   1.3053   C.3     1 1    0.0000
12 H12   -1.4568   1.7280   2.1366   H       1 1    0.0000
13 C13   -1.5291  -0.2522  -1.3053   C.3     1 1    0.0000
14 H14   -2.2302  -0.3731  -2.1357   H       1 1    0.0000
15 C15   -2.1622  -0.7899   0.0000   C.3     1 1    0.0000
16 H16   -3.2486  -0.9058   0.0000   H       1 1    0.0000
17 C17   -1.5291  -0.2522   1.3053   C.3     1 1    0.0000
18 H18   -2.2302  -0.3731   2.1357   H       1 1    0.0000
19 C19    1.5250   0.2765   1.3051   C.3     1 1    0.0000
20 H20    2.2258   0.3979   2.1357   H       1 1    0.0000
21 C21    2.3020  -0.0174   0.0000   C.3     1 1    0.0000
22 H22    3.3644   0.2378   0.0000   H       1 1    0.0000
23 C23    1.5250   0.2765  -1.3051   C.3     1 1    0.0000
24 H24    2.2258   0.3979  -2.1357   H       1 1    0.0000
25 C25    0.9830  -1.1983  -1.3052   C.3     1 1    0.0000
26 H26    1.4376  -1.7441  -2.1366   H       1 1    0.0000
27 C27   -0.5230  -1.4591  -1.3053   C.3     1 1    0.0000
28 H28   -0.7682  -2.1269  -2.1357   H       1 1    0.0000
29 C29   -0.5230  -1.4591   1.3053   C.3     1 1    0.0000
30 H30   -0.7682  -2.1269   2.1357   H       1 1    0.0000
31 C31    0.9830  -1.1983   1.3052   C.3     1 1    0.0000
32 H32    1.4376  -1.7441   2.1366   H       1 1    0.0000
33 C33   -1.1665  -1.9847   0.0000   C.3     1 1    0.0000
34 H34   -1.4765  -3.0324   0.0000   H       1 1    0.0000
35 C35    1.7657  -1.4775   0.0000   C.3     1 1    0.0000
36 H36    2.4094  -2.3603   0.0000   H       1 1    0.0000
@<TRIPOS>BOND
1   1   2   1
2   1   3   1
```

3	1	6	1
4	1	13	1
5	2	4	1
6	2	8	1
7	2	23	1
8	3	4	1
9	3	7	1
10	3	11	1
11	4	5	1
12	4	9	1
13	9	10	1
14	9	11	1
15	9	19	1
16	11	12	1
17	11	17	1
18	13	14	1
19	13	15	1
20	13	27	1
21	15	16	1
22	15	17	1
23	15	33	1
24	17	18	1
25	17	29	1
26	19	20	1
27	19	21	1
28	19	31	1
29	21	22	1
30	21	23	1
31	21	35	1
32	23	24	1
33	23	25	1
34	25	26	1
35	25	27	1
36	25	35	1
37	27	28	1
38	27	33	1
39	29	30	1
40	29	31	1
41	29	33	1
42	31	32	1
43	31	35	1
44	33	34	1
45	35	36	1

@<TRIPOS>SUBSTRUCTURE
1 RES1 1 GROUP 0 **** **** 0

Figure S5. Chemical structure of nugget$_{18}$ generated by the Blink software. Cartesian coordinates of its atoms; the first line contains the total charge and multiplicity; the following lines contain the atomic numbers, followed by the x, y, and z coordinates in Å for each one of the atoms. Next, atomic coordinates in Tripos Mol2 file format (.mol2) with the distances also in Å.

Nugget$_{20a}$ ($C_{20}H_{20}$)

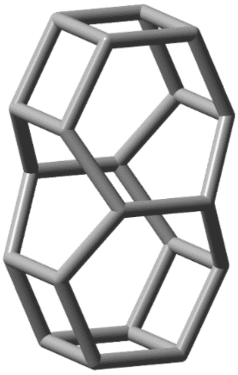

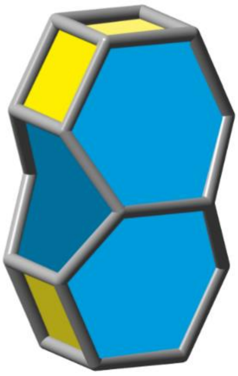

Cartesian Coordinates (Å)

0 1

6	2.602	-0.774	-0.780
6	2.602	-0.774	0.780
6	2.602	0.774	-0.780
6	2.602	0.774	0.780
1	3.386	1.254	1.369
1	3.386	-1.254	-1.369
1	3.386	1.254	-1.369
1	3.386	-1.254	1.369
6	1.213	-1.450	0.783
1	1.275	-2.475	1.166
6	1.213	-1.450	-0.783
1	1.275	-2.475	-1.166
6	1.213	1.450	-0.783
1	1.275	2.475	-1.166
6	1.213	1.450	0.783
1	1.275	2.475	1.166
6	0.000	0.781	1.447
1	0.000	1.079	2.501
6	0.000	-0.781	1.447
1	0.000	-1.079	2.501
6	0.000	-0.781	-1.447
1	0.000	-1.079	-2.501
6	0.000	0.781	-1.447
1	0.000	1.079	-2.501
6	-1.213	-1.450	0.782
1	-1.275	-2.475	1.166
6	-1.213	-1.450	-0.783
1	-1.275	-2.475	-1.166
6	-1.213	1.450	0.783
1	-1.275	2.475	1.166
6	-2.602	-0.774	0.780
1	-3.386	-1.254	1.369
6	-2.602	0.774	0.780
1	-3.386	1.254	1.369
6	-2.602	-0.774	-0.780

```
1   -3.386   -1.254   -1.369
6   -2.602    0.774   -0.780
1   -3.386    1.254   -1.369
6   -1.213    1.450   -0.782
1   -1.275    2.475   -1.166
```

.mol2 file

```
@<TRIPOS>MOLECULE
Molecule Name
40   50   1   0   0
SMALL
NO_CHARGES
****
Generated from the CSD

@<TRIPOS>ATOM
1 C1      2.6022  -0.7737  -0.7796   C.3     1 1     0.0000
2 C2      2.6023  -0.7737   0.7796   C.3     1 1     0.0000
3 C3      2.6022   0.7737  -0.7796   C.3     1 1     0.0000
4 C4      2.6022   0.7737   0.7796   C.3     1 1     0.0000
5 H5      3.3864   1.2542   1.3691   H       1 1     0.0000
6 H6      3.3864  -1.2541  -1.3692   H       1 1     0.0000
7 H7      3.3864   1.2542  -1.3690   H       1 1     0.0000
8 H8      3.3864  -1.2542   1.3691   H       1 1     0.0000
9 C9      1.2133  -1.4504   0.7826   C.3     1 1     0.0000
10 H10    1.2753  -2.4748   1.1655   H       1 1     0.0000
11 C11    1.2133  -1.4504  -0.7826   C.3     1 1     0.0000
12 H12    1.2753  -2.4748  -1.1655   H       1 1     0.0000
13 C13    1.2133   1.4504  -0.7826   C.3     1 1     0.0000
14 H14    1.2752   2.4749  -1.1655   H       1 1     0.0000
15 C15    1.2132   1.4504   0.7826   C.3     1 1     0.0000
16 H16    1.2753   2.4748   1.1656   H       1 1     0.0000
17 C17    0.0000   0.7811   1.4466   C.3     1 1     0.0000
18 H18    0.0000   1.0789   2.5011   H       1 1     0.0000
19 C19    0.0000  -0.7811   1.4466   C.3     1 1     0.0000
20 H20    0.0000  -1.0790   2.5011   H       1 1     0.0000
21 C21    0.0000  -0.7811  -1.4466   C.3     1 1     0.0000
22 H22    0.0000  -1.0789  -2.5011   H       1 1     0.0000
23 C23    0.0000   0.7811  -1.4466   C.3     1 1     0.0000
24 H24    0.0000   1.0790  -2.5011   H       1 1     0.0000
25 C25   -1.2133  -1.4504   0.7825   C.3     1 1     0.0000
26 H26   -1.2753  -2.4748   1.1655   H       1 1     0.0000
27 C27   -1.2133  -1.4504  -0.7826   C.3     1 1     0.0000
28 H28   -1.2753  -2.4748  -1.1656   H       1 1     0.0000
29 C29   -1.2133   1.4504   0.7826   C.3     1 1     0.0000
30 H30   -1.2753   2.4748   1.1656   H       1 1     0.0000
31 C31   -2.6022  -0.7737   0.7796   C.3     1 1     0.0000
32 H32   -3.3864  -1.2542   1.3691   H       1 1     0.0000
33 C33   -2.6022   0.7737   0.7796   C.3     1 1     0.0000
34 H34   -3.3864   1.2542   1.3691   H       1 1     0.0000
```

35 C35 -2.6022 -0.7737 -0.7796 C.3 1 1 0.0000
36 H36 -3.3864 -1.2542 -1.3691 H 1 1 0.0000
37 C37 -2.6022 0.7737 -0.7796 C.3 1 1 0.0000
38 H38 -3.3864 1.2542 -1.3691 H 1 1 0.0000
39 C39 -1.2133 1.4504 -0.7825 C.3 1 1 0.0000
40 H40 -1.2753 2.4748 -1.1655 H 1 1 0.0000
@<TRIPOS>BOND
1 1 2 1
2 1 3 1
3 1 6 1
4 1 11 1
5 2 4 1
6 2 8 1
7 2 9 1
8 3 4 1
9 3 7 1
10 3 13 1
11 4 5 1
12 4 15 1
13 9 10 1
14 9 11 1
15 9 19 1
16 11 12 1
17 11 21 1
18 13 14 1
19 13 15 1
20 13 23 1
21 15 16 1
22 15 17 1
23 17 18 1
24 17 19 1
25 17 29 1
26 19 20 1
27 19 25 1
28 21 22 1
29 21 23 1
30 21 27 1
31 23 24 1
32 23 39 1
33 25 26 1
34 25 27 1
35 25 31 1
36 27 28 1
37 27 35 1
38 29 30 1
39 29 33 1
40 29 39 1
41 31 32 1
42 31 33 1
43 31 35 1

```
44    33    34    1
45    33    37    1
46    35    36    1
47    35    37    1
48    37    38    1
49    37    39    1
50    39    40    1
@<TRIPOS>SUBSTRUCTURE
1 RES1       1 GROUP            0 ****  ****    0
```

Figure S6. Chemical structure of nugget$_{20a}$ generated by the Blink software. Cartesian coordinates of its atoms; the first line contains the total charge and multiplicity; the following lines contain the atomic numbers, followed by the x, y, and z coordinates in Å for each one of the atoms. Next, atomic coordinates in Tripos Mol2 file format (.mol2) with the distances also in Å.

Nugget$_{20b}$ ($C_{20}H_{20}$)

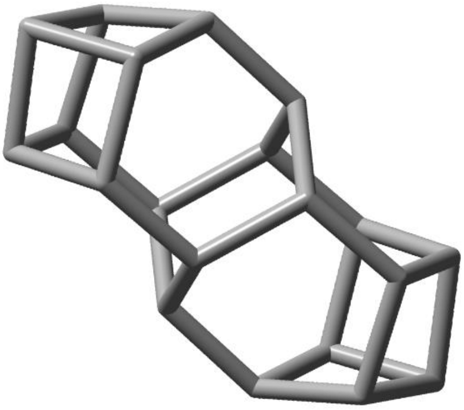

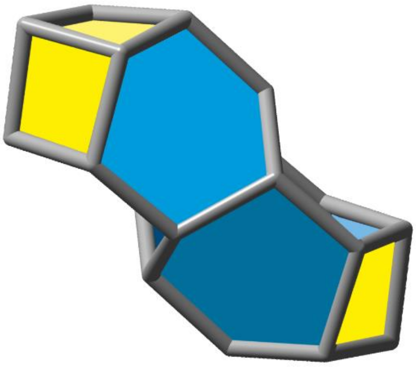

Cartesian Coordinates (Å)

0 1

6	2.528	-0.925	0.853
6	3.448	-0.001	0.000
6	1.813	-1.400	-0.437
6	2.529	-0.276	-1.227
1	2.882	-0.502	-2.236
1	2.881	-1.685	1.554
1	2.201	-2.378	-0.741
1	4.537	-0.001	0.000
6	2.529	1.201	0.375
1	2.882	2.188	0.682
6	1.813	0.322	1.431
1	2.201	0.546	2.430
6	1.813	1.079	-0.994
1	2.200	1.831	-1.688
6	0.269	-1.382	-0.432
1	-0.114	-2.364	-0.738
6	-0.268	-0.317	-1.412
1	0.115	-0.542	-2.415
6	0.268	1.065	-0.981
1	-0.114	1.821	-1.678
6	-0.269	1.382	0.432
6	0.268	0.317	1.412
1	-0.115	0.542	2.415
6	-0.268	-1.065	0.981
1	0.114	-1.821	1.678
1	0.114	2.364	0.738
6	-1.813	-0.322	-1.431
1	-2.200	-0.546	-2.430
6	-1.813	-1.079	0.994
1	-2.200	-1.831	1.688
6	-2.529	0.276	1.227
1	-2.882	0.502	2.236
6	-2.528	0.925	-0.853
1	-2.881	1.685	-1.554

```
6    -3.448    0.001   -0.000
1    -4.537    0.001   -0.000
6    -2.529   -1.201   -0.375
1    -2.882   -2.188   -0.682
6    -1.813    1.400    0.437
1    -2.201    2.378    0.741
```

.mol2 file

```
@<TRIPOS>MOLECULE
Molecule Name
   40    50     1     0     0
SMALL
NO_CHARGES
****
Generated from the CSD

@<TRIPOS>ATOM
     1 C1       2.5281  -0.9246   0.8530   C.3       1 1      0.0000
     2 C2       3.4478  -0.0006   0.0001   C.3       1 1      0.0000
     3 C3       1.8131  -1.4004  -0.4370   C.3       1 1      0.0000
     4 C4       2.5287  -0.2759  -1.2272   C.3       1 1      0.0000
     5 H5       2.8816  -0.5023  -2.2362   H         1 1      0.0000
     6 H6       2.8806  -1.6845   1.5543   H         1 1      0.0000
     7 H7       2.2009  -2.3777  -0.7411   H         1 1      0.0000
     8 H8       4.5367  -0.0012   0.0001   H         1 1      0.0000
     9 C9       2.5291   1.2010   0.3745   C.3       1 1      0.0000
    10 H10      2.8822   2.1881   0.6821   H         1 1      0.0000
    11 C11      1.8128   0.3223   1.4312   C.3       1 1      0.0000
    12 H12      2.2006   0.5464   2.4299   H         1 1      0.0000
    13 C13      1.8131   1.0788  -0.9941   C.3       1 1      0.0000
    14 H14      2.2003   1.8312  -1.6883   H         1 1      0.0000
    15 C15      0.2688  -1.3821  -0.4317   C.3       1 1      0.0000
    16 H16     -0.1137  -2.3636  -0.7378   H         1 1      0.0000
    17 C17     -0.2683  -0.3174  -1.4123   C.3       1 1      0.0000
    18 H18      0.1153  -0.5420  -2.4152   H         1 1      0.0000
    19 C19      0.2684   1.0649  -0.9807   C.3       1 1      0.0000
    20 H20     -0.1144   1.8207  -1.6775   H         1 1      0.0000
    21 C21     -0.2688   1.3821   0.4317   C.3       1 1      0.0000
    22 C22      0.2683   0.3173   1.4123   C.3       1 1      0.0000
    23 H23     -0.1152   0.5420   2.4152   H         1 1      0.0000
    24 C24     -0.2684  -1.0649   0.9807   C.3       1 1      0.0000
    25 H25      0.1144  -1.8207   1.6775   H         1 1      0.0000
    26 H26      0.1137   2.3636   0.7378   H         1 1      0.0000
    27 C27     -1.8128  -0.3223  -1.4312   C.3       1 1      0.0000
    28 H28     -2.2005  -0.5464  -2.4299   H         1 1      0.0000
    29 C29     -1.8131  -1.0788   0.9941   C.3       1 1      0.0000
    30 H30     -2.2003  -1.8312   1.6883   H         1 1      0.0000
    31 C31     -2.5287   0.2759   1.2272   C.3       1 1      0.0000
```

```
   32 H32      -2.8816   0.5023   2.2362   H         1 1      0.0000
   33 C33      -2.5281   0.9246  -0.8530   C.3       1 1      0.0000
   34 H34      -2.8806   1.6845  -1.5543   H         1 1      0.0000
   35 C35      -3.4478   0.0006  -0.0001   C.3       1 1      0.0000
   36 H36      -4.5367   0.0012  -0.0001   H         1 1      0.0000
   37 C37      -2.5291  -1.2010  -0.3745   C.3       1 1      0.0000
   38 H38      -2.8822  -2.1881  -0.6821   H         1 1      0.0000
   39 C39      -1.8132   1.4004   0.4370   C.3       1 1      0.0000
   40 H40      -2.2009   2.3777   0.7410   H         1 1      0.0000
@<TRIPOS>BOND
    1    1    2    1
    2    1    3    1
    3    1    6    1
    4    1   11    1
    5    2    4    1
    6    2    8    1
    7    2    9    1
    8    3    4    1
    9    3    7    1
   10    3   15    1
   11    4    5    1
   12    4   13    1
   13    9   10    1
   14    9   11    1
   15    9   13    1
   16   11   12    1
   17   11   22    1
   18   13   14    1
   19   13   19    1
   20   15   16    1
   21   15   17    1
   22   15   24    1
   23   17   18    1
   24   17   19    1
   25   17   27    1
   26   19   20    1
   27   19   21    1
   28   21   22    1
   29   21   26    1
   30   21   39    1
   31   22   23    1
   32   22   24    1
   33   24   25    1
   34   24   29    1
   35   27   28    1
   36   27   33    1
   37   27   37    1
   38   29   30    1
   39   29   31    1
   40   29   37    1
```

```
  41   31   32   1
  42   31   35   1
  43   31   39   1
  44   33   34   1
  45   33   35   1
  46   33   39   1
  47   35   36   1
  48   35   37   1
  49   37   38   1
  50   39   40   1
@<TRIPOS>SUBSTRUCTURE
     1 RES1        1 GROUP             0 ****  ****    0
```

Figure S7. Chemical structure of $nugget_{20b}$ generated by the Blink software. Cartesian coordinates of its atoms; the first line contains the total charge and multiplicity; the following lines contain the atomic numbers, followed by the x, y, and z coordinates in Å for each one of the atoms. Next, atomic coordinates in Tripos Mol2 file format (.mol2) with the distances also in Å.

Nugget$_{20c}$ ($C_{20}H_{20}$)

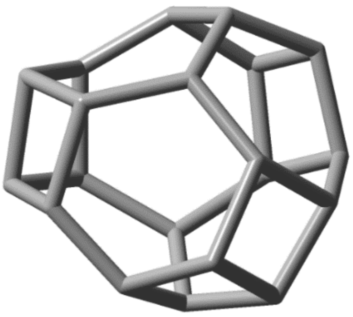

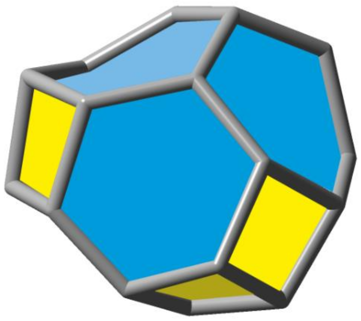

Cartesian Coordinates (Å)

0 1

6	2.455	0.053	-0.584
1	3.401	0.195	-1.111
6	2.261	-0.957	0.584
1	3.087	-1.440	1.111
6	1.511	0.153	1.388
1	2.046	0.364	2.317
6	1.939	1.189	0.299
1	2.771	1.785	0.688
6	1.347	-0.700	-1.388
1	1.766	-1.094	-2.317
6	1.362	-1.822	-0.299
1	1.914	-2.684	-0.689
6	0.060	-2.273	0.300
6	0.000	0.001	1.761
1	0.001	0.001	2.853
6	-0.624	-1.384	1.388
1	-0.709	-1.952	2.318
6	0.000	-0.000	-1.761
1	0.000	-0.000	-2.853
6	0.896	2.090	-0.300
1	1.366	2.999	-0.690
6	-0.068	1.516	-1.388
1	0.064	2.075	-2.318
6	-0.887	1.232	1.388
1	-1.337	1.590	2.317
6	-0.301	2.436	0.584
1	-0.296	3.394	1.110
6	-1.960	-1.479	0.583
1	-2.790	-1.954	1.111
6	-2.258	-0.269	-0.300
6	-1.999	1.085	0.300
1	-2.931	1.506	0.690
6	-1.181	-2.152	-0.583
1	-1.531	-3.043	-1.110
6	-1.280	-0.817	-1.388
6	-1.274	2.099	-0.584

```
1   -1.870   2.847  -1.111
1   -1.830  -0.983  -2.317
1   -3.281  -0.316  -0.690
1    0.160  -3.292   0.690
```

.mol2 file

```
@<TRIPOS>MOLECULE
Molecule Name
40   50    1    0    0
SMALL
NO_CHARGES
****
Generated from the CSD

@<TRIPOS>ATOM
1 C1      2.4547   0.0530  -0.5836   C.3      1 1     0.0000
2 H2      3.4013   0.1953  -1.1107   H        1 1     0.0000
3 C3      2.2607  -0.9572   0.5837   C.3      1 1     0.0000
4 H4      3.0874  -1.4395   1.1111   H        1 1     0.0000
5 C5      1.5105   0.1527   1.3876   C.3      1 1     0.0000
6 H6      2.0459   0.3640   2.3165   H        1 1     0.0000
7 C7      1.9390   1.1889   0.2991   C.3      1 1     0.0000
8 H8      2.7711   1.7853   0.6883   H        1 1     0.0000
9 C9      1.3470  -0.7002  -1.3878   C.3      1 1     0.0000
10 H10    1.7661  -1.0939  -2.3170   H        1 1     0.0000
11 C11    1.3616  -1.8217  -0.2994   C.3      1 1     0.0000
12 H12    1.9141  -2.6838  -0.6885   H        1 1     0.0000
13 C13    0.0598  -2.2734   0.3002   C.3      1 1     0.0000
14 C14    0.0004   0.0005   1.7609   C.3      1 1     0.0000
15 H15    0.0008   0.0006   2.8534   H        1 1     0.0000
16 C16   -0.6235  -1.3835   1.3882   C.3      1 1     0.0000
17 H17   -0.7087  -1.9516   2.3177   H        1 1     0.0000
18 C18    0.0001  -0.0003  -1.7608   C.3      1 1     0.0000
19 H19    0.0002  -0.0001  -2.8533   H        1 1     0.0000
20 C20    0.8963   2.0900  -0.3001   C.3      1 1     0.0000
21 H21    1.3659   2.9994  -0.6900   H        1 1     0.0000
22 C22   -0.0675   1.5161  -1.3880   C.3      1 1     0.0000
23 H23    0.0637   2.0753  -2.3176   H        1 1     0.0000
24 C24   -0.8868   1.2318   1.3880   C.3      1 1     0.0000
25 H25   -1.3366   1.5896   2.3174   H        1 1     0.0000
26 C26   -0.3014   2.4364   0.5835   C.3      1 1     0.0000
27 H27   -0.2965   3.3937   1.1104   H        1 1     0.0000
28 C28   -1.9595  -1.4790   0.5834   C.3      1 1     0.0000
29 H29   -2.7904  -1.9539   1.1110   H        1 1     0.0000
30 C30   -2.2584  -0.2685  -0.3001   C.3      1 1     0.0000
31 C31   -1.9989   1.0847   0.2998   C.3      1 1     0.0000
32 H32   -2.9314   1.5063   0.6900   H        1 1     0.0000
33 C33   -1.1809  -2.1522  -0.5833   C.3      1 1     0.0000
```

34 H34 -1.5308 -3.0435 -1.1099 H 1 1 0.0000
35 C35 -1.2796 -0.8168 -1.3881 C.3 1 1 0.0000
36 C36 -1.2735 2.0988 -0.5836 C.3 1 1 0.0000
37 H37 -1.8702 2.8474 -1.1108 H 1 1 0.0000
38 H38 -1.8302 -0.9832 -2.3171 H 1 1 0.0000
39 H39 -3.2808 -0.3158 -0.6901 H 1 1 0.0000
40 H40 0.1602 -3.2921 0.6900 H 1 1 0.0000
@<TRIPOS>BOND
1 1 2 1
2 1 3 1
3 1 7 1
4 1 9 1
5 3 4 1
6 3 5 1
7 3 11 1
8 5 6 1
9 5 7 1
10 5 14 1
11 7 8 1
12 7 20 1
13 9 10 1
14 9 11 1
15 9 18 1
16 11 12 1
17 11 13 1
18 13 16 1
19 13 33 1
20 13 40 1
21 14 15 1
22 14 16 1
23 14 24 1
24 16 17 1
25 16 28 1
26 18 19 1
27 18 22 1
28 18 35 1
29 20 21 1
30 20 22 1
31 20 26 1
32 22 23 1
33 22 36 1
34 24 25 1
35 24 26 1
36 24 31 1
37 26 27 1
38 26 36 1
39 28 29 1
40 28 30 1
41 28 33 1
42 30 31 1

43	30	35	1
44	30	39	1
45	31	32	1
46	31	36	1
47	33	34	1
48	33	35	1
49	35	38	1
50	36	37	1

@<TRIPOS>SUBSTRUCTURE
1 RES1 1 GROUP 0 **** **** 0

Figure S8. Chemical structure of nugget$_{20c}$ generated by the Blink software. Cartesian coordinates of its atoms; the first line contains the total charge and multiplicity; the following lines contain the atomic numbers, followed by the x, y, and z coordinates in Å for each one of the atoms. Next, atomic coordinates in Tripos Mol2 file format (.mol2) with the distances also in Å.

Nugget$_{22}$ ($C_{22}H_{22}$)

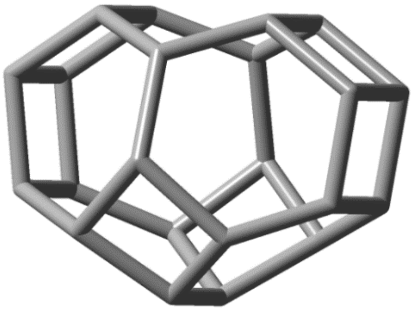

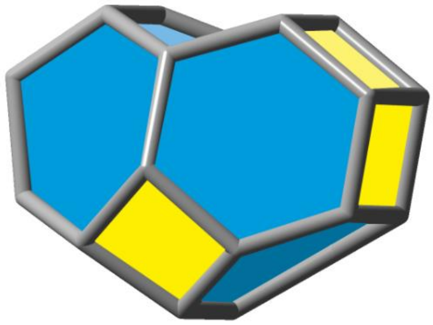

Cartesian Coordinates (Å)

0 1

6	1.251	-1.677	0.783
6	2.536	-0.820	0.781
6	1.251	-1.677	-0.783
6	2.536	-0.820	-0.781
1	3.369	-1.186	-1.385
1	1.483	-2.692	1.123
1	1.483	-2.692	-1.123
1	3.369	-1.186	1.385
6	2.303	0.709	0.781
1	3.196	1.228	1.139
6	2.303	0.709	-0.781
1	3.196	1.228	-1.139
6	0.000	-1.216	1.556
1	0.000	-1.796	2.484
6	-1.251	-1.677	0.783
1	-1.483	-2.692	1.123
6	-1.251	-1.677	-0.783
1	-1.483	-2.692	-1.123
6	0.000	-1.216	-1.556
1	0.000	-1.796	-2.484
6	0.000	0.256	2.035
1	0.000	0.217	3.128
6	1.088	1.242	1.525
1	1.436	1.833	2.378
6	0.000	0.256	-2.035
1	0.000	0.217	-3.128
6	1.088	1.242	-1.525
1	1.436	1.833	-2.378
6	-2.536	-0.820	0.781
1	-3.369	-1.186	1.385
6	-2.536	-0.820	-0.781
1	-3.369	-1.186	-1.385
6	-2.303	0.709	0.781
1	-3.196	1.228	1.139

```
6   -2.303   0.709   -0.781
6   -1.088   1.242    1.525
1   -1.436   1.833    2.378
6    0.000   2.068   -0.768
6    0.000   2.068    0.768
1    0.000   3.113    1.089
1   -3.196   1.228   -1.139
1    0.000   3.113   -1.089
6   -1.088   1.242   -1.525
1   -1.436   1.833   -2.378
```

.mol2 file

```
@<TRIPOS>MOLECULE
Molecule Name
44   55    1    0    0
SMALL
NO_CHARGES
****
Generated from the CSD

@<TRIPOS>ATOM
1 C1      1.2514  -1.6770   0.7829   C.3      1 1     0.0000
2 C2      2.5359  -0.8196   0.7806   C.3      1 1     0.0000
3 C3      1.2514  -1.6770  -0.7829   C.3      1 1     0.0000
4 C4      2.5359  -0.8197  -0.7806   C.3      1 1     0.0000
5 H5      3.3688  -1.1864  -1.3854   H        1 1     0.0000
6 H6      1.4828  -2.6915   1.1227   H        1 1     0.0000
7 H7      1.4827  -2.6915  -1.1228   H        1 1     0.0000
8 H8      3.3688  -1.1864   1.3854   H        1 1     0.0000
9 C9      2.3027   0.7093   0.7807   C.3      1 1     0.0000
10 H10     3.1963   1.2282   1.1391   H        1 1     0.0000
11 C11     2.3026   0.7093  -0.7807   C.3      1 1     0.0000
12 H12     3.1963   1.2281  -1.1392   H        1 1     0.0000
13 C13     0.0000  -1.2155   1.5558   C.3      1 1     0.0000
14 H14     0.0000  -1.7964   2.4836   H        1 1     0.0000
15 C15    -1.2514  -1.6770   0.7829   C.3      1 1     0.0000
16 H16    -1.4827  -2.6915   1.1227   H        1 1     0.0000
17 C17    -1.2514  -1.6770  -0.7829   C.3      1 1     0.0000
18 H18    -1.4827  -2.6915  -1.1228   H        1 1     0.0000
19 C19     0.0000  -1.2155  -1.5558   C.3      1 1     0.0000
20 H20     0.0000  -1.7964  -2.4836   H        1 1     0.0000
21 C21     0.0000   0.2557   2.0351   C.3      1 1     0.0000
22 H22     0.0000   0.2170   3.1280   H        1 1     0.0000
23 C23     1.0880   1.2417   1.5248   C.3      1 1     0.0000
24 H24     1.4357   1.8326   2.3777   H        1 1     0.0000
25 C25     0.0000   0.2557  -2.0351   C.3      1 1     0.0000
26 H26     0.0000   0.2170  -3.1280   H        1 1     0.0000
27 C27     1.0880   1.2417  -1.5248   C.3      1 1     0.0000
28 H28     1.4357   1.8326  -2.3777   H        1 1     0.0000
```

```
29 C29     -2.5359  -0.8197   0.7806   C.3       1 1      0.0000
30 H30     -3.3688  -1.1864   1.3854   H         1 1      0.0000
31 C31     -2.5359  -0.8197  -0.7807   C.3       1 1      0.0000
32 H32     -3.3688  -1.1864  -1.3854   H         1 1      0.0000
33 C33     -2.3026   0.7093   0.7807   C.3       1 1      0.0000
34 H34     -3.1963   1.2282   1.1392   H         1 1      0.0000
35 C35     -2.3026   0.7093  -0.7807   C.3       1 1      0.0000
36 C36     -1.0880   1.2417   1.5248   C.3       1 1      0.0000
37 H37     -1.4357   1.8326   2.3777   H         1 1      0.0000
38 C38      0.0000   2.0678  -0.7676   C.3       1 1      0.0000
39 C39      0.0000   2.0678   0.7676   C.3       1 1      0.0000
40 H40      0.0000   3.1134   1.0891   H         1 1      0.0000
41 H41     -3.1963   1.2282  -1.1392   H         1 1      0.0000
42 H42      0.0000   3.1134  -1.0890   H         1 1      0.0000
43 C43     -1.0880   1.2417  -1.5248   C.3       1 1      0.0000
44 H44     -1.4357   1.8326  -2.3777   H         1 1      0.0000
@<TRIPOS>BOND
1    1    2    1
2    1    3    1
3    1    6    1
4    1    13   1
5    2    4    1
6    2    8    1
7    2    9    1
8    3    4    1
9    3    7    1
10   3    19   1
11   4    5    1
12   4    11   1
13   9    10   1
14   9    11   1
15   9    23   1
16   11   12   1
17   11   27   1
18   13   14   1
19   13   15   1
20   13   21   1
21   15   16   1
22   15   17   1
23   15   29   1
24   17   18   1
25   17   19   1
26   17   31   1
27   19   20   1
28   19   25   1
29   21   22   1
30   21   23   1
31   21   36   1
32   23   24   1
33   23   39   1
```

```
34    25    26    1
35    25    27    1
36    25    43    1
37    27    28    1
38    27    38    1
39    29    30    1
40    29    31    1
41    29    33    1
42    31    32    1
43    31    35    1
44    33    34    1
45    33    35    1
46    33    36    1
47    35    41    1
48    35    43    1
49    36    37    1
50    36    39    1
51    38    39    1
52    38    42    1
53    38    43    1
54    39    40    1
55    43    44    1
@<TRIPOS>SUBSTRUCTURE
1 RES1       1 GROUP             0 ****  ****    0
```

Figure S9. Chemical structure of $nugget_{22}$ generated by the Blink software. Cartesian coordinates of its atoms; the first line contains the total charge and multiplicity; the following lines contain the atomic numbers, followed by the x, y, and z coordinates in Å for each one of the atoms. Next, atomic coordinates in Tripos Mol2 file format (.mol2) with the distances also in Å.

Nugget$_{24a}$ ($C_{24}H_{24}$)

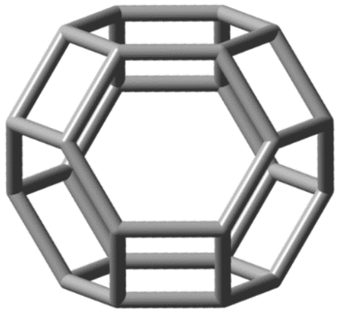

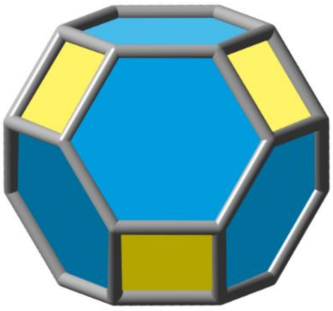

Cartesian Coordinates (Å)

0 1

6	1.303	1.859	-0.908
6	2.281	0.704	-0.532
6	1.040	2.129	0.605
6	2.018	0.974	0.982
1	2.919	1.397	1.434
1	1.873	2.692	-1.329
1	1.489	3.086	0.884
1	3.304	1.003	-0.779
6	2.068	-0.695	-1.107
1	3.000	-0.990	-1.597
6	-0.086	0.164	-2.438
1	-0.112	0.223	-3.530
6	0.132	1.592	-1.851
1	0.207	2.311	-2.671
6	1.585	-1.853	-0.180
1	2.295	-2.683	-0.243
6	1.327	-1.589	1.301
1	1.927	-2.307	1.868
6	1.546	-0.161	1.888
1	2.246	-0.219	2.726
6	-0.389	2.126	1.144
1	-0.546	3.082	1.651
6	-1.327	1.589	-1.301
1	-1.927	2.307	-1.868
6	-1.585	1.853	0.180
1	-2.295	2.683	0.243
6	0.389	-2.126	-1.144
1	0.546	-3.082	-1.651
6	-1.546	0.161	-1.888
1	-2.246	0.219	-2.726
6	-0.872	0.967	2.070
1	-1.251	1.388	3.005
6	0.086	-0.164	2.438
1	0.112	-0.223	3.530
6	-0.132	-1.592	1.851
1	-0.207	-2.311	2.671

6	-2.068	0.695	1.107
1	-3.000	0.990	1.597
6	-2.281	-0.704	0.532
1	-3.304	-1.003	0.779
6	-1.303	-1.859	0.908
1	-1.873	-2.692	1.329
6	-1.040	-2.129	-0.605
1	-1.489	-3.086	-0.884
6	-2.018	-0.974	-0.982
1	-2.919	-1.397	-1.434
6	0.872	-0.967	-2.070
1	1.251	-1.388	-3.005

.mol2 file

```
@<TRIPOS>MOLECULE
Molecule Name
48   60    1    0    0
SMALL
NO_CHARGES
****
Generated from the CSD

@<TRIPOS>ATOM
1 C1      1.3032   1.8592  -0.9080   C.3      1 1      0.0000
2 C2      2.2813   0.7038  -0.5319   C.3      1 1      0.0000
3 C3      1.0398   2.1290   0.6052   C.3      1 1      0.0000
4 C4      2.0178   0.9735   0.9815   C.3      1 1      0.0000
5 H5      2.9186   1.3968   1.4343   H        1 1      0.0000
6 H6      1.8734   2.6921  -1.3287   H        1 1      0.0000
7 H7      1.4894   3.0861   0.8838   H        1 1      0.0000
8 H8      3.3037   1.0032  -0.7786   H        1 1      0.0000
9 C9      2.0676  -0.6948  -1.1065   C.3      1 1      0.0000
10 H10     2.9997  -0.9896  -1.5965   H        1 1      0.0000
11 C11    -0.0858   0.1637  -2.4381   C.3      1 1      0.0000
12 H12    -0.1122   0.2234  -3.5296   H        1 1      0.0000
13 C13     0.1323   1.5920  -1.8509   C.3      1 1      0.0000
14 H14     0.2068   2.3110  -2.6714   H        1 1      0.0000
15 C15     1.5854  -1.8535  -0.1803   C.3      1 1      0.0000
16 H16     2.2949  -2.6832  -0.2426   H        1 1      0.0000
17 C17     1.3275  -1.5892   1.3012   C.3      1 1      0.0000
18 H18     1.9266  -2.3070   1.8682   H        1 1      0.0000
19 C19     1.5455  -0.1607   1.8882   C.3      1 1      0.0000
20 H20     2.2460  -0.2187   2.7258   H        1 1      0.0000
21 C21    -0.3892   2.1260   1.1435   C.3      1 1      0.0000
22 H22    -0.5460   3.0817   1.6512   H        1 1      0.0000
23 C23    -1.3275   1.5892  -1.3012   C.3      1 1      0.0000
24 H24    -1.9266   2.3070  -1.8682   H        1 1      0.0000
25 C25    -1.5854   1.8535   0.1803   C.3      1 1      0.0000
26 H26    -2.2949   2.6832   0.2426   H        1 1      0.0000
```

27 C27 0.3892 -2.1260 -1.1435 C.3 1 1 0.0000
28 H28 0.5460 -3.0817 -1.6512 H 1 1 0.0000
29 C29 -1.5455 0.1607 -1.8882 C.3 1 1 0.0000
30 H30 -2.2460 0.2187 -2.7258 H 1 1 0.0000
31 C31 -0.8715 0.9674 2.0697 C.3 1 1 0.0000
32 H32 -1.2514 1.3883 3.0047 H 1 1 0.0000
33 C33 0.0858 -0.1637 2.4381 C.3 1 1 0.0000
34 H34 0.1122 -0.2234 3.5296 H 1 1 0.0000
35 C35 -0.1323 -1.5920 1.8509 C.3 1 1 0.0000
36 H36 -0.2068 -2.3110 2.6714 H 1 1 0.0000
37 C37 -2.0676 0.6948 1.1065 C.3 1 1 0.0000
38 H38 -2.9997 0.9896 1.5965 H 1 1 0.0000
39 C39 -2.2813 -0.7038 0.5319 C.3 1 1 0.0000
40 H40 -3.3037 -1.0032 0.7786 H 1 1 0.0000
41 C41 -1.3032 -1.8592 0.9080 C.3 1 1 0.0000
42 H42 -1.8734 -2.6921 1.3287 H 1 1 0.0000
43 C43 -1.0398 -2.1290 -0.6052 C.3 1 1 0.0000
44 H44 -1.4894 -3.0861 -0.8838 H 1 1 0.0000
45 C45 -2.0178 -0.9735 -0.9815 C.3 1 1 0.0000
46 H46 -2.9186 -1.3968 -1.4343 H 1 1 0.0000
47 C47 0.8715 -0.9674 -2.0697 C.3 1 1 0.0000
48 H48 1.2514 -1.3883 -3.0047 H 1 1 0.0000
@<TRIPOS>BOND
1 1 2 1
2 1 3 1
3 1 6 1
4 1 13 1
5 2 4 1
6 2 8 1
7 2 9 1
8 3 4 1
9 3 7 1
10 3 21 1
11 4 5 1
12 4 19 1
13 9 10 1
14 9 15 1
15 9 47 1
16 11 12 1
17 11 13 1
18 11 29 1
19 11 47 1
20 13 14 1
21 13 23 1
22 15 16 1
23 15 17 1
24 15 27 1
25 17 18 1
26 17 19 1
27 17 35 1

```
28   19   20   1
29   19   33   1
30   21   22   1
31   21   25   1
32   21   31   1
33   23   24   1
34   23   25   1
35   23   29   1
36   25   26   1
37   25   37   1
38   27   28   1
39   27   43   1
40   27   47   1
41   29   30   1
42   29   45   1
43   31   32   1
44   31   33   1
45   31   37   1
46   33   34   1
47   33   35   1
48   35   36   1
49   35   41   1
50   37   38   1
51   37   39   1
52   39   40   1
53   39   41   1
54   39   45   1
55   41   42   1
56   41   43   1
57   43   44   1
58   43   45   1
59   45   46   1
60   47   48   1
@<TRIPOS>SUBSTRUCTURE
1 RES1       1 GROUP            0 ****  ****    0
```

Figure S10. Chemical structure of nugget$_{24a}$ generated by the Blink software. Cartesian coordinates of its atoms; the first line contains the total charge and multiplicity; the following lines contain the atomic numbers, followed by the x, y, and z coordinates in Å for each one of the atoms. Next, atomic coordinates in Tripos Mol2 file format (.mol2) with the distances also in Å.

Nugget$_{24b}$ ($C_{24}H_{24}$)

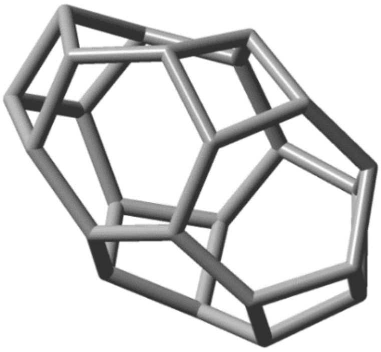

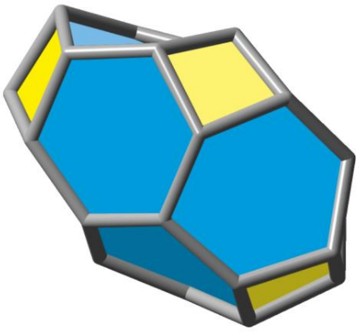

Cartesian Coordinates (Å)

0 1

6	-0.357	1.918	-1.030
6	1.005	2.176	-0.352
6	-1.005	2.176	0.352
6	0.357	1.918	1.030
1	0.636	2.683	1.762
1	-0.636	2.683	-1.762
1	-1.234	3.246	0.416
1	1.234	3.246	-0.416
6	2.211	1.369	-0.746
1	2.908	1.982	-1.325
6	-0.577	0.551	-1.726
6	2.014	-0.003	1.474
1	2.528	-0.031	2.438
6	0.577	0.551	1.726
1	0.580	0.837	2.781
1	-0.580	0.837	-2.781
6	0.577	-0.551	-1.726
1	0.580	-0.837	-2.781
6	2.014	0.003	-1.474
1	2.528	0.031	-2.438
6	-2.014	-0.003	-1.474
1	-2.528	-0.031	-2.438
6	-2.941	0.664	-0.402
1	-3.846	1.133	-0.796
6	-2.211	1.369	0.746
1	-2.908	1.982	1.325
6	-0.577	-0.551	1.726
1	-0.580	-0.837	2.781
6	0.357	-1.918	-1.030
1	0.636	-2.683	-1.762
6	-1.005	-2.176	-0.352
1	-1.234	-3.246	-0.416
6	-2.211	-1.369	-0.746
1	-2.908	-1.982	-1.325
6	-2.941	-0.664	0.402
1	-3.846	-1.133	0.796

6	2.211	-1.369	0.746
1	2.908	-1.982	1.325
6	2.941	-0.664	-0.402
1	3.846	-1.133	-0.796
6	2.941	0.664	0.402
1	3.846	1.133	0.796
6	-2.014	0.003	1.474
1	-2.528	0.031	2.438
6	1.005	-2.176	0.352
1	1.234	-3.246	0.416
6	-0.357	-1.918	1.030
1	-0.635	-2.683	1.762

.mol2 file

```
@<TRIPOS>MOLECULE
Molecule Name
48    60     1     0     0
SMALL
NO_CHARGES
****
Generated from the CSD

@<TRIPOS>ATOM
1 C1      -0.3568   1.9182  -1.0304   C.3       1 1      0.0000
2 C2       1.0053   2.1763  -0.3518   C.3       1 1      0.0000
3 C3      -1.0053   2.1763   0.3519   C.3       1 1      0.0000
4 C4       0.3568   1.9182   1.0304   C.3       1 1      0.0000
5 H5       0.6356   2.6833   1.7622   H         1 1      0.0000
6 H6      -0.6356   2.6834  -1.7621   H         1 1      0.0000
7 H7      -1.2341   3.2457   0.4162   H         1 1      0.0000
8 H8       1.2342   3.2457  -0.4161   H         1 1      0.0000
9 C9       2.2105   1.3689  -0.7457   C.3       1 1      0.0000
10 H10     2.9079   1.9821  -1.3254   H         1 1      0.0000
11 C11    -0.5767   0.5507  -1.7264   C.3       1 1      0.0000
12 C12     2.0138  -0.0025   1.4736   C.3       1 1      0.0000
13 H13     2.5283  -0.0307   2.4376   H         1 1      0.0000
14 C14     0.5767   0.5507   1.7264   C.3       1 1      0.0000
15 H15     0.5801   0.8367   2.7812   H         1 1      0.0000
16 H16    -0.5801   0.8368  -2.7812   H         1 1      0.0000
17 C17     0.5767  -0.5507  -1.7264   C.3       1 1      0.0000
18 H18     0.5801  -0.8367  -2.7812   H         1 1      0.0000
19 C19     2.0139   0.0025  -1.4736   C.3       1 1      0.0000
20 H20     2.5283   0.0307  -2.4377   H         1 1      0.0000
21 C21    -2.0138  -0.0025  -1.4736   C.3       1 1      0.0000
22 H22    -2.5283  -0.0307  -2.4377   H         1 1      0.0000
23 C23    -2.9410   0.6645  -0.4023   C.3       1 1      0.0000
24 H24    -3.8459   1.1333  -0.7965   H         1 1      0.0000
25 C25    -2.2105   1.3689   0.7456   C.3       1 1      0.0000
26 H26    -2.9079   1.9821   1.3254   H         1 1      0.0000
```

```
27 C27     -0.5767  -0.5507   1.7264   C.3       1 1      0.0000
28 H28     -0.5801  -0.8368   2.7812   H         1 1      0.0000
29 C29      0.3568  -1.9182  -1.0304   C.3       1 1      0.0000
30 H30      0.6356  -2.6833  -1.7622   H         1 1      0.0000
31 C31     -1.0053  -2.1763  -0.3519   C.3       1 1      0.0000
32 H32     -1.2341  -3.2457  -0.4162   H         1 1      0.0000
33 C33     -2.2105  -1.3689  -0.7457   C.3       1 1      0.0000
34 H34     -2.9079  -1.9821  -1.3254   H         1 1      0.0000
35 C35     -2.9410  -0.6645   0.4023   C.3       1 1      0.0000
36 H36     -3.8459  -1.1333   0.7965   H         1 1      0.0000
37 C37      2.2105  -1.3689   0.7457   C.3       1 1      0.0000
38 H38      2.9079  -1.9821   1.3254   H         1 1      0.0000
39 C39      2.9410  -0.6645  -0.4022   C.3       1 1      0.0000
40 H40      3.8459  -1.1332  -0.7965   H         1 1      0.0000
41 C41      2.9410   0.6645   0.4022   C.3       1 1      0.0000
42 H42      3.8459   1.1333   0.7965   H         1 1      0.0000
43 C43     -2.0138   0.0025   1.4736   C.3       1 1      0.0000
44 H44     -2.5283   0.0307   2.4377   H         1 1      0.0000
45 C45      1.0053  -2.1763   0.3518   C.3       1 1      0.0000
46 H46      1.2341  -3.2457   0.4161   H         1 1      0.0000
47 C47     -0.3568  -1.9182   1.0304   C.3       1 1      0.0000
48 H48     -0.6355  -2.6834   1.7621   H         1 1      0.0000
@<TRIPOS>BOND
1     1     2    1
2     1     3    1
3     1     6    1
4     1    11    1
5     2     4    1
6     2     8    1
7     2     9    1
8     3     4    1
9     3     7    1
10     3    25    1
11     4     5    1
12     4    14    1
13     9    10    1
14     9    19    1
15     9    41    1
16    11    16    1
17    11    17    1
18    11    21    1
19    12    13    1
20    12    14    1
21    12    37    1
22    12    41    1
23    14    15    1
24    14    27    1
25    17    18    1
26    17    19    1
27    17    29    1
```

28	19	20	1
29	19	39	1
30	21	22	1
31	21	23	1
32	21	33	1
33	23	24	1
34	23	25	1
35	23	35	1
36	25	26	1
37	25	43	1
38	27	28	1
39	27	43	1
40	27	47	1
41	29	30	1
42	29	31	1
43	29	45	1
44	31	32	1
45	31	33	1
46	31	47	1
47	33	34	1
48	33	35	1
49	35	36	1
50	35	43	1
51	37	38	1
52	37	39	1
53	37	45	1
54	39	40	1
55	39	41	1
56	41	42	1
57	43	44	1
58	45	46	1
59	45	47	1
60	47	48	1

@<TRIPOS>SUBSTRUCTURE
1 RES1 1 GROUP 0 **** **** 0

Figure S11. Chemical structure of nugget$_{24b}$ generated by the Blink software. Cartesian coordinates of its atoms; the first line contains the total charge and multiplicity; the following lines contain the atomic numbers, followed by the x, y, and z coordinates in Å for each one of the atoms. Next, atomic coordinates in Tripos Mol2 file format (.mol2) with the distances also in Å.

Nugget$_{24c}$ ($C_{24}H_{24}$)

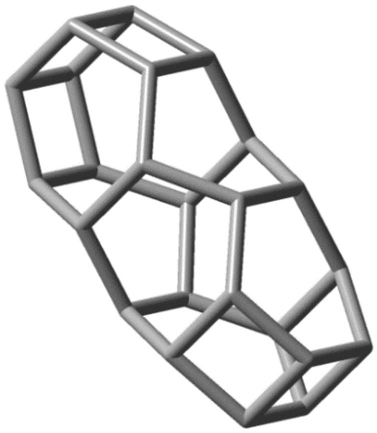

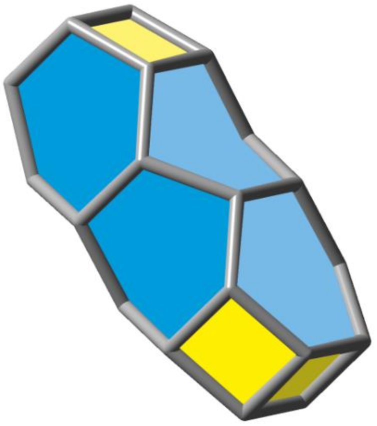

Cartesian Coordinates (Å)

0 1

6	-3.236	-1.069	0.253
6	-3.236	-0.245	-1.071
6	-3.236	0.245	1.071
6	-3.236	1.069	-0.253
1	-4.021	1.787	-0.501
1	-4.021	-1.787	0.501
1	-4.021	0.340	1.825
1	-4.021	-0.340	-1.825
6	-1.850	-0.828	-1.432
1	-1.910	-1.488	-2.305
6	-1.850	-1.651	-0.110
1	-1.911	-2.725	-0.315
6	-1.850	0.828	1.432
1	-1.910	1.488	2.305
6	-1.850	1.651	0.110
1	-1.911	2.725	0.315
6	-0.635	-1.412	0.786
1	-0.619	-2.221	1.526
6	-0.635	-0.081	1.614
1	-0.618	-0.387	2.668
6	-0.635	1.412	-0.786
1	-0.619	2.221	-1.526
6	-0.635	0.081	-1.614
1	-0.618	0.387	-2.668
6	0.635	-0.786	-1.412
1	0.619	-1.526	-2.221
6	0.635	-1.614	-0.081
1	0.618	-2.668	-0.387
6	0.635	0.786	1.412
1	0.619	1.526	2.221
6	0.635	1.614	0.081
1	0.618	2.668	0.387
6	1.850	1.432	-0.828

1	1.910	2.305	-1.488
6	1.850	0.110	-1.650
1	1.911	0.315	-2.725
6	1.850	-1.432	0.828
1	1.910	-2.305	1.488
6	1.850	-0.110	1.651
1	1.911	-0.315	2.725
6	3.236	-0.253	-1.069
1	4.021	-0.502	-1.787
6	3.236	-1.071	0.245
1	4.021	-1.825	0.340
6	3.236	0.253	1.069
1	4.021	0.501	1.787
6	3.236	1.071	-0.245
1	4.021	1.825	-0.340

.mol2 file

```
@<TRIPOS>MOLECULE
Molecule Name
48   60    1    0    0
SMALL
NO_CHARGES
****
Generated from the CSD

@<TRIPOS>ATOM
1 C1      -3.2358  -1.0691   0.2532   C.3      1 1     0.0000
2 C2      -3.2360  -0.2454  -1.0713   C.3      1 1     0.0000
3 C3      -3.2360   0.2453   1.0712   C.3      1 1     0.0000
4 C4      -3.2358   1.0691  -0.2532   C.3      1 1     0.0000
5 H5      -4.0208   1.7870  -0.5015   H        1 1     0.0000
6 H6      -4.0208  -1.7871   0.5015   H        1 1     0.0000
7 H7      -4.0214   0.3398   1.8247   H        1 1     0.0000
8 H8      -4.0214  -0.3399  -1.8247   H        1 1     0.0000
9 C9      -1.8497  -0.8281  -1.4322   C.3      1 1     0.0000
10 H10    -1.9096  -1.4880  -2.3046   H        1 1     0.0000
11 C11    -1.8497  -1.6505  -0.1099   C.3      1 1     0.0000
12 H12    -1.9109  -2.7248  -0.3152   H        1 1     0.0000
13 C13    -1.8497   0.8281   1.4322   C.3      1 1     0.0000
14 H14    -1.9097   1.4880   2.3045   H        1 1     0.0000
15 C15    -1.8497   1.6505   0.1099   C.3      1 1     0.0000
16 H16    -1.9109   2.7248   0.3153   H        1 1     0.0000
17 C17    -0.6354  -1.4116   0.7859   C.3      1 1     0.0000
18 H18    -0.6187  -2.2211   1.5262   H        1 1     0.0000
19 C19    -0.6353  -0.0811   1.6138   C.3      1 1     0.0000
20 H20    -0.6180  -0.3867   2.6675   H        1 1     0.0000
21 C21    -0.6354   1.4116  -0.7859   C.3      1 1     0.0000
22 H22    -0.6187   2.2211  -1.5262   H        1 1     0.0000
23 C23    -0.6354   0.0811  -1.6138   C.3      1 1     0.0000
```

```
24 H24     -0.6182   0.3868  -2.6675   H         1 1      0.0000
25 C25      0.6354  -0.7859  -1.4115   C.3       1 1      0.0000
26 H26      0.6187  -1.5261  -2.2211   H         1 1      0.0000
27 C27      0.6353  -1.6138  -0.0811   C.3       1 1      0.0000
28 H28      0.6182  -2.6675  -0.3867   H         1 1      0.0000
29 C29      0.6354   0.7860   1.4115   C.3       1 1      0.0000
30 H30      0.6186   1.5262   2.2210   H         1 1      0.0000
31 C31      0.6354   1.6139   0.0810   C.3       1 1      0.0000
32 H32      0.6182   2.6676   0.3867   H         1 1      0.0000
33 C33      1.8497   1.4322  -0.8281   C.3       1 1      0.0000
34 H34      1.9097   2.3045  -1.4881   H         1 1      0.0000
35 C35      1.8497   0.1098  -1.6504   C.3       1 1      0.0000
36 H36      1.9109   0.3151  -2.7247   H         1 1      0.0000
37 C37      1.8496  -1.4322   0.8282   C.3       1 1      0.0000
38 H38      1.9096  -2.3045   1.4882   H         1 1      0.0000
39 C39      1.8497  -0.1098   1.6505   C.3       1 1      0.0000
40 H40      1.9109  -0.3151   2.7248   H         1 1      0.0000
41 C41      3.2358  -0.2533  -1.0690   C.3       1 1      0.0000
42 H42      4.0207  -0.5017  -1.7870   H         1 1      0.0000
43 C43      3.2359  -1.0713   0.2454   C.3       1 1      0.0000
44 H44      4.0213  -1.8248   0.3399   H         1 1      0.0000
45 C45      3.2358   0.2532   1.0690   C.3       1 1      0.0000
46 H46      4.0208   0.5015   1.7870   H         1 1      0.0000
47 C47      3.2361   1.0712  -0.2454   C.3       1 1      0.0000
48 H48      4.0215   1.8246  -0.3400   H         1 1      0.0000
@<TRIPOS>BOND
1    1    2    1
2    1    3    1
3    1    6    1
4    1    11   1
5    2    4    1
6    2    8    1
7    2    9    1
8    3    4    1
9    3    7    1
10   3    13   1
11   4    5    1
12   4    15   1
13   9    10   1
14   9    11   1
15   9    23   1
16   11   12   1
17   11   17   1
18   13   14   1
19   13   15   1
20   13   19   1
21   15   16   1
22   15   21   1
23   17   18   1
24   17   19   1
```

25	17	27	1
26	19	20	1
27	19	29	1
28	21	22	1
29	21	23	1
30	21	31	1
31	23	24	1
32	23	25	1
33	25	26	1
34	25	27	1
35	25	35	1
36	27	28	1
37	27	37	1
38	29	30	1
39	29	31	1
40	29	39	1
41	31	32	1
42	31	33	1
43	33	34	1
44	33	35	1
45	33	47	1
46	35	36	1
47	35	41	1
48	37	38	1
49	37	39	1
50	37	43	1
51	39	40	1
52	39	45	1
53	41	42	1
54	41	43	1
55	41	47	1
56	43	44	1
57	43	45	1
58	45	46	1
59	45	47	1
60	47	48	1

@<TRIPOS>SUBSTRUCTURE

1 RES1 1 GROUP 0 **** **** 0

Figure S12. Chemical structure of nugget$_{24c}$ generated by the Blink software. Cartesian coordinates of its atoms; the first line contains the total charge and multiplicity; the following lines contain the atomic numbers, followed by the x, y, and z coordinates in Å for each one of the atoms. Next, atomic coordinates in Tripos Mol2 file format (.mol2) with the distances also in Å.

Nugget$_{26a}$ ($C_{26}H_{26}$)

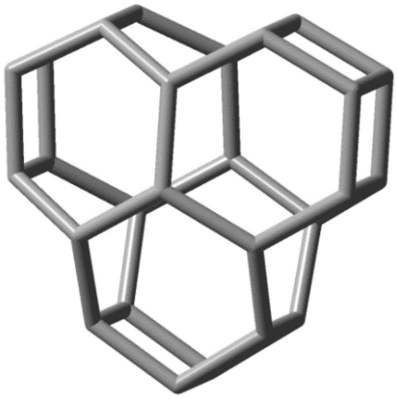

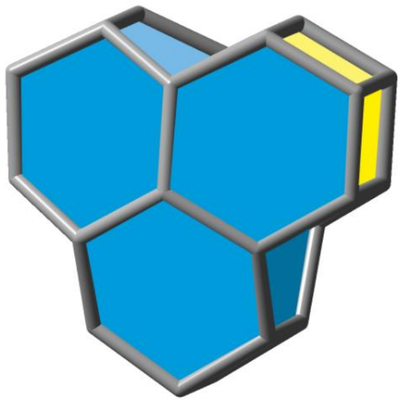

Cartesian Coordinates (Å)

0 1

6	-1.477	0.487	1.497
6	1.161	1.035	1.497
6	-0.606	2.915	0.784
6	0.821	2.349	0.780
6	-1.689	1.828	0.780
6	0.000	-0.000	1.772
1	1.487	1.327	2.501
1	-0.790	3.803	1.394
1	-1.893	0.625	2.501
1	1.537	3.097	1.140
1	-2.644	2.228	1.140
1	0.000	-0.001	2.861
6	0.317	-1.523	1.495
1	0.406	-1.951	2.500
6	-2.446	-0.463	0.780
1	-3.451	-0.218	1.141
6	-2.222	-1.982	0.784
1	-2.899	-2.586	1.393
6	-0.739	-2.377	0.780
1	-0.610	-3.404	1.140
6	1.625	-1.886	0.780
1	1.916	-2.879	1.140
6	2.828	-0.933	0.784
1	3.689	-1.217	1.394
6	2.428	0.549	0.780
1	3.252	1.175	1.140
6	2.428	0.549	-0.780
1	3.252	1.175	-1.140
6	1.161	1.035	-1.497
1	1.487	1.327	-2.501
6	-0.606	2.915	-0.784
1	-0.790	3.803	-1.394
6	-1.689	1.828	-0.780
1	-2.644	2.228	-1.140

6	-2.446	-0.463	-0.780
1	-3.451	-0.218	-1.141
6	-1.477	0.487	-1.497
1	-1.893	0.625	-2.501
6	2.828	-0.933	-0.784
1	3.689	-1.217	-1.394
6	-2.222	-1.982	-0.784
1	-2.899	-2.586	-1.393
6	-0.739	-2.377	-0.780
1	-0.610	-3.404	-1.140
6	1.625	-1.886	-0.780
1	1.916	-2.879	-1.140
6	0.317	-1.523	-1.495
1	0.406	-1.951	-2.500
6	0.000	-0.000	-1.772
1	0.000	-0.001	-2.861
6	0.821	2.349	-0.780
1	1.537	3.097	-1.140

.mol2 file

@<TRIPOS>MOLECULE
Molecule Name
52 65 1 0 0
SMALL
NO_CHARGES
****
Generated from the CSD

@<TRIPOS>ATOM
1 C1 -1.4772 0.4874 1.4966 C.3 1 1 0.0000
2 C2 1.1607 1.0354 1.4966 C.3 1 1 0.0000
3 C3 -0.6058 2.9153 0.7840 C.3 1 1 0.0000
4 C4 0.8213 2.3494 0.7803 C.3 1 1 0.0000
5 C5 -1.6893 1.8277 0.7803 C.3 1 1 0.0000
6 C6 0.0002 -0.0001 1.7716 C.3 1 1 0.0000
7 H7 1.4873 1.3270 2.5012 H 1 1 0.0000
8 H8 -0.7904 3.8028 1.3937 H 1 1 0.0000
9 H9 -1.8929 0.6250 2.5011 H 1 1 0.0000
10 H10 1.5370 3.0971 1.1400 H 1 1 0.0000
11 H11 -2.6435 2.2285 1.1401 H 1 1 0.0000
12 H12 0.0004 -0.0007 2.8612 H 1 1 0.0000
13 C13 0.3165 -1.5231 1.4954 C.3 1 1 0.0000
14 H14 0.4056 -1.9508 2.5004 H 1 1 0.0000
15 C15 -2.4455 -0.4632 0.7805 C.3 1 1 0.0000
16 H16 -3.4507 -0.2176 1.1405 H 1 1 0.0000
17 C17 -2.2223 -1.9820 0.7840 C.3 1 1 0.0000
18 H18 -2.8990 -2.5856 1.3933 H 1 1 0.0000
19 C19 -0.7390 -2.3770 0.7803 C.3 1 1 0.0000
20 H20 -0.6102 -3.4037 1.1401 H 1 1 0.0000
21 C21 1.6247 -1.8861 0.7802 C.3 1 1 0.0000
22 H22 1.9157 -2.8792 1.1400 H 1 1 0.0000
23 C23 2.8280 -0.9330 0.7839 C.3 1 1 0.0000

24 H24 3.6890 -1.2169 1.3936 H 1 1 0.0000
25 C25 2.4276 0.5491 0.7804 C.3 1 1 0.0000
26 H26 3.2515 1.1751 1.1403 H 1 1 0.0000
27 C27 2.4276 0.5491 -0.7804 C.3 1 1 0.0000
28 H28 3.2515 1.1751 -1.1403 H 1 1 0.0000
29 C29 1.1607 1.0354 -1.4966 C.3 1 1 0.0000
30 H30 1.4873 1.3270 -2.5012 H 1 1 0.0000
31 C31 -0.6058 2.9153 -0.7840 C.3 1 1 0.0000
32 H32 -0.7904 3.8028 -1.3937 H 1 1 0.0000
33 C33 -1.6893 1.8277 -0.7803 C.3 1 1 0.0000
34 H34 -2.6435 2.2285 -1.1401 H 1 1 0.0000
35 C35 -2.4455 -0.4632 -0.7805 C.3 1 1 0.0000
36 H36 -3.4507 -0.2176 -1.1405 H 1 1 0.0000
37 C37 -1.4772 0.4874 -1.4966 C.3 1 1 0.0000
38 H38 -1.8929 0.6250 -2.5011 H 1 1 0.0000
39 C39 2.8280 -0.9330 -0.7839 C.3 1 1 0.0000
40 H40 3.6890 -1.2169 -1.3936 H 1 1 0.0000
41 C41 -2.2223 -1.9820 -0.7840 C.3 1 1 0.0000
42 H42 -2.8990 -2.5856 -1.3933 H 1 1 0.0000
43 C43 -0.7390 -2.3770 -0.7803 C.3 1 1 0.0000
44 H44 -0.6102 -3.4038 -1.1401 H 1 1 0.0000
45 C45 1.6247 -1.8861 -0.7802 C.3 1 1 0.0000
46 H46 1.9157 -2.8792 -1.1401 H 1 1 0.0000
47 C47 0.3166 -1.5231 -1.4954 C.3 1 1 0.0000
48 H48 0.4056 -1.9508 -2.5004 H 1 1 0.0000
49 C49 0.0002 -0.0001 -1.7716 C.3 1 1 0.0000
50 H50 0.0004 -0.0007 -2.8612 H 1 1 0.0000
51 C51 0.8213 2.3494 -0.7803 C.3 1 1 0.0000
52 H52 1.5370 3.0971 -1.1400 H 1 1 0.0000
@<TRIPOS>BOND
1 1 5 1
2 1 6 1
3 1 9 1
4 1 15 1
5 2 4 1
6 2 6 1
7 2 7 1
8 2 25 1
9 3 4 1
10 3 5 1
11 3 8 1
12 3 31 1
13 4 10 1
14 4 51 1
15 5 11 1
16 5 33 1
17 6 12 1
18 6 13 1
19 13 14 1
20 13 19 1
21 13 21 1
22 15 16 1
23 15 17 1
24 15 35 1
25 17 18 1

26 17 19 1
27 17 41 1
28 19 20 1
29 19 43 1
30 21 22 1
31 21 23 1
32 21 45 1
33 23 24 1
34 23 25 1
35 23 39 1
36 25 26 1
37 25 27 1
38 27 28 1
39 27 29 1
40 27 39 1
41 29 30 1
42 29 49 1
43 29 51 1
44 31 32 1
45 31 33 1
46 31 51 1
47 33 34 1
48 33 37 1
49 35 36 1
50 35 37 1
51 35 41 1
52 37 38 1
53 37 49 1
54 39 40 1
55 39 45 1
56 41 42 1
57 41 43 1
58 43 44 1
59 43 47 1
60 45 46 1
61 45 47 1
62 47 48 1
63 47 49 1
64 49 50 1
65 51 52 1
@<TRIPOS>SUBSTRUCTURE
1 RES1 1 GROUP 0 **** **** 0

Figure S13. Chemical structure of nugget$_{26a}$ generated by the Blink software. Cartesian coordinates of its atoms; the first line contains the total charge and multiplicity; the following lines contain the atomic numbers, followed by the x, y, and z coordinates in Å for each one of the atoms. Next, atomic coordinates in Tripos Mol2 file format (.mol2) with the distances also in Å.

Nugget$_{26b}$ ($C_{26}H_{26}$)

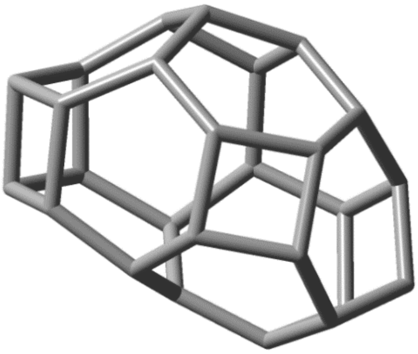

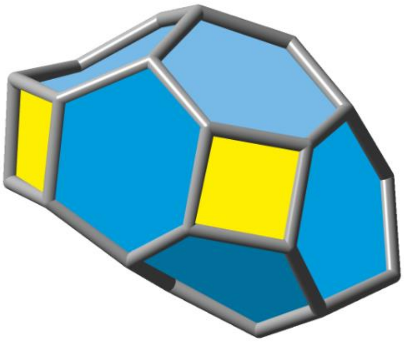

Cartesian Coordinates (Å)

0 1

6	-0.283	2.248	0.457
6	-0.302	0.714	-1.734
6	-2.566	1.266	-0.403
6	-1.843	0.721	-1.677
6	-1.747	1.827	0.746
6	0.326	1.854	-0.892
1	-0.043	1.037	-2.750
1	-3.305	2.012	-0.712
1	-0.123	3.328	0.558
1	-2.215	1.260	-2.553
1	-2.297	2.655	1.203
1	0.155	2.733	-1.527
6	0.168	1.497	1.735
1	0.271	2.243	2.529
6	-1.252	0.870	1.870
1	-1.698	1.154	2.827
6	-3.245	-0.101	-0.202
1	-4.322	-0.099	-0.017
6	-2.417	-1.128	0.610
1	-3.082	-1.875	1.052
6	-1.462	-0.656	1.736
1	-1.979	-0.926	2.661
6	-0.168	-1.497	1.735
1	-0.271	-2.243	2.529
6	1.252	-0.870	1.870
1	1.698	-1.154	2.827
6	1.462	0.656	1.736
1	1.979	0.926	2.661
6	1.843	1.710	-0.725
1	2.305	2.688	-0.896
6	-1.843	-1.710	-0.725
1	-2.305	-2.688	-0.896
6	-0.326	-1.854	-0.892
1	-0.155	-2.733	-1.527
6	0.283	-2.248	0.457

1	0.123	-3.328	0.558
6	-2.597	-0.627	-1.520
1	-3.175	-0.958	-2.386
6	1.747	-1.827	0.746
1	2.297	-2.655	1.203
6	2.597	0.627	-1.520
1	3.175	0.958	-2.386
6	1.843	-0.721	-1.677
1	2.215	-1.260	-2.553
6	0.302	-0.714	-1.734
1	0.043	-1.037	-2.750
6	3.245	0.101	-0.202
1	4.322	0.099	-0.017
6	2.566	-1.266	-0.403
1	3.305	-2.012	-0.712
6	2.417	1.128	0.610
1	3.082	1.875	1.052

.mol2 file

```
@<TRIPOS>MOLECULE
Molecule Name
52    65     1     0     0
SMALL
NO_CHARGES
****
Generated from the CSD

@<TRIPOS>ATOM
1 C1      -0.2831   2.2482   0.4574   C.3      1 1     0.0000
2 C2      -0.3022   0.7141  -1.7344   C.3      1 1     0.0000
3 C3      -2.5658   1.2662  -0.4032   C.3      1 1     0.0000
4 C4      -1.8426   0.7207  -1.6772   C.3      1 1     0.0000
5 C5      -1.7467   1.8267   0.7461   C.3      1 1     0.0000
6 C6       0.3262   1.8539  -0.8922   C.3      1 1     0.0000
7 H7      -0.0429   1.0373  -2.7499   H        1 1     0.0000
8 H8      -3.3048   2.0118  -0.7124   H        1 1     0.0000
9 H9      -0.1226   3.3278   0.5576   H        1 1     0.0000
10 H10    -2.2147   1.2596  -2.5534   H        1 1     0.0000
11 H11    -2.2971   2.6549   1.2032   H        1 1     0.0000
12 H12     0.1548   2.7331  -1.5267   H        1 1     0.0000
13 C13     0.1678   1.4967   1.7352   C.3      1 1     0.0000
14 H14     0.2705   2.2433   2.5291   H        1 1     0.0000
15 C15    -1.2521   0.8698   1.8703   C.3      1 1     0.0000
16 H16    -1.6982   1.1539   2.8274   H        1 1     0.0000
17 C17    -3.2453  -0.1013  -0.2015   C.3      1 1     0.0000
18 H18    -4.3222  -0.0992  -0.0169   H        1 1     0.0000
19 C19    -2.4165  -1.1283   0.6104   C.3      1 1     0.0000
20 H20    -3.0824  -1.8754   1.0522   H        1 1     0.0000
21 C21    -1.4625  -0.6560   1.7358   C.3      1 1     0.0000
22 H22    -1.9788  -0.9264   2.6611   H        1 1     0.0000
23 C23    -0.1678  -1.4967   1.7352   C.3      1 1     0.0000
24 H24    -0.2705  -2.2432   2.5291   H        1 1     0.0000
```

```
25 C25      1.2521  -0.8698   1.8703   C.3      1 1      0.0000
26 H26      1.6982  -1.1539   2.8274   H        1 1      0.0000
27 C27      1.4625   0.6560   1.7358   C.3      1 1      0.0000
28 H28      1.9788   0.9264   2.6611   H        1 1      0.0000
29 C29      1.8426   1.7100  -0.7254   C.3      1 1      0.0000
30 H30      2.3049   2.6877  -0.8959   H        1 1      0.0000
31 C31     -1.8426  -1.7100  -0.7254   C.3      1 1      0.0000
32 H32     -2.3049  -2.6878  -0.8958   H        1 1      0.0000
33 C33     -0.3261  -1.8539  -0.8922   C.3      1 1      0.0000
34 H34     -0.1548  -2.7331  -1.5266   H        1 1      0.0000
35 C35      0.2831  -2.2482   0.4574   C.3      1 1      0.0000
36 H36      0.1226  -3.3278   0.5576   H        1 1      0.0000
37 C37     -2.5967  -0.6269  -1.5197   C.3      1 1      0.0000
38 H38     -3.1754  -0.9576  -2.3858   H        1 1      0.0000
39 C39      1.7467  -1.8267   0.7461   C.3      1 1      0.0000
40 H40      2.2972  -2.6548   1.2033   H        1 1      0.0000
41 C41      2.5967   0.6269  -1.5197   C.3      1 1      0.0000
42 H42      3.1754   0.9575  -2.3858   H        1 1      0.0000
43 C43      1.8426  -0.7207  -1.6772   C.3      1 1      0.0000
44 H44      2.2147  -1.2596  -2.5534   H        1 1      0.0000
45 C45      0.3022  -0.7141  -1.7344   C.3      1 1      0.0000
46 H46      0.0429  -1.0374  -2.7499   H        1 1      0.0000
47 C47      3.2453   0.1013  -0.2015   C.3      1 1      0.0000
48 H48      4.3223   0.0992  -0.0169   H        1 1      0.0000
49 C49      2.5658  -1.2663  -0.4032   C.3      1 1      0.0000
50 H50      3.3048  -2.0118  -0.7123   H        1 1      0.0000
51 C51      2.4165   1.1283   0.6104   C.3      1 1      0.0000
52 H52      3.0824   1.8754   1.0522   H        1 1      0.0000
@<TRIPOS>BOND
1     1     5    1
2     1     6    1
3     1     9    1
4     1    13    1
5     2     4    1
6     2     6    1
7     2     7    1
8     2    45    1
9     3     4    1
10    3     5    1
11    3     8    1
12    3    17    1
13    4    10    1
14    4    37    1
15    5    11    1
16    5    15    1
17    6    12    1
18    6    29    1
19   13    14    1
20   13    15    1
21   13    27    1
22   15    16    1
23   15    21    1
24   17    18    1
25   17    19    1
26   17    37    1
```

```
27   19   20   1
28   19   21   1
29   19   31   1
30   21   22   1
31   21   23   1
32   23   24   1
33   23   25   1
34   23   35   1
35   25   26   1
36   25   27   1
37   25   39   1
38   27   28   1
39   27   51   1
40   29   30   1
41   29   41   1
42   29   51   1
43   31   32   1
44   31   33   1
45   31   37   1
46   33   34   1
47   33   35   1
48   33   45   1
49   35   36   1
50   35   39   1
51   37   38   1
52   39   40   1
53   39   49   1
54   41   42   1
55   41   43   1
56   41   47   1
57   43   44   1
58   43   45   1
59   43   49   1
60   45   46   1
61   47   48   1
62   47   49   1
63   47   51   1
64   49   50   1
65   51   52   1
@<TRIPOS>SUBSTRUCTURE
1 RES1       1 GROUP            0 ****  ****    0
```

Figure S14. Chemical structure of nugget$_{26b}$ generated by the Blink software. Cartesian coordinates of its atoms; the first line contains the total charge and multiplicity; the following lines contain the atomic numbers, followed by the x, y, and z coordinates in Å for each one of the atoms. Next, atomic coordinates in Tripos Mol2 file format (.mol2) with the distances also in Å.

Nugget$_{26c}$ ($C_{26}H_{26}$)

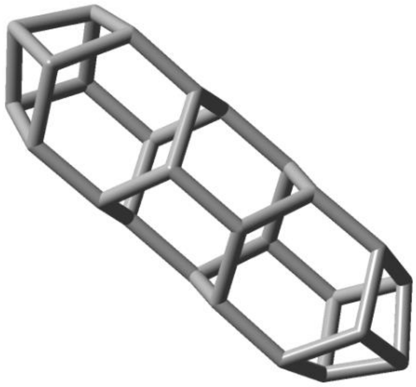
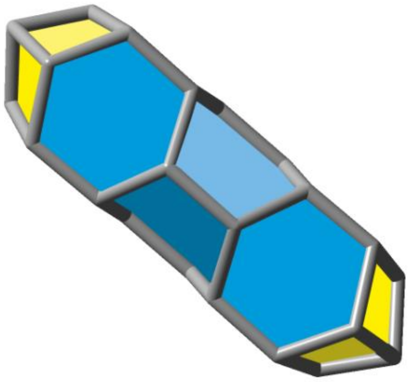

Cartesian Coordinates (Å)

0 1

6	3.606	0.996	0.767
6	4.527	-0.000	0.001
6	2.889	-0.193	1.449
6	3.606	-1.162	0.480
1	3.956	-2.119	0.874
1	3.956	1.817	1.398
1	3.272	-0.329	2.465
1	5.616	-0.000	0.001
6	3.607	0.166	-1.246
1	3.957	0.303	-2.272
6	2.889	1.351	-0.557
1	3.273	2.299	-0.948
6	2.889	-1.158	-0.891
1	3.272	-1.971	-1.517
6	1.346	1.333	-0.550
1	0.997	2.291	-0.945
6	0.784	1.139	0.876
1	1.139	1.961	1.509
6	1.345	-0.190	1.429
1	0.996	-0.328	2.456
6	0.785	0.190	-1.425
1	1.140	0.327	-2.453
6	1.346	-1.143	-0.880
1	0.997	-1.964	-1.512
6	0.784	-1.329	0.548
1	1.140	-2.288	0.943
6	-0.784	1.139	0.876
1	-1.139	1.961	1.509
6	-0.784	0.190	-1.425
1	-1.140	0.327	-2.453
6	-0.784	-1.329	0.548
1	-1.140	-2.288	0.943
6	-1.345	-0.190	1.429
1	-0.996	-0.328	2.456

6	-1.346	-1.143	-0.880
1	-0.997	-1.964	-1.512
6	-2.889	-0.193	1.449
1	-3.272	-0.329	2.465
6	-3.606	-1.162	0.480
1	-3.956	-2.119	0.874
6	-2.889	-1.158	-0.891
1	-3.272	-1.971	-1.517
6	-1.346	1.333	-0.550
1	-0.997	2.291	-0.945
6	-2.889	1.351	-0.557
1	-3.273	2.299	-0.948
6	-3.607	0.166	-1.246
1	-3.957	0.303	-2.272
6	-3.606	0.996	0.767
1	-3.956	1.817	1.398
6	-4.527	-0.000	0.001
1	-5.616	-0.000	0.001

.mol2 file

```
@<TRIPOS>MOLECULE
Molecule Name
52    65    1    0    0
SMALL
NO_CHARGES
****
Generated from the CSD

@<TRIPOS>ATOM
1 C1      3.6063   0.9962   0.7670   C.3     1 1     0.0000
2 C2      4.5273  -0.0001   0.0005   C.3     1 1     0.0000
3 C3      2.8888  -0.1928   1.4490   C.3     1 1     0.0000
4 C4      3.6062  -1.1620   0.4796   C.3     1 1     0.0000
5 H5      3.9562  -2.1188   0.8743   H       1 1     0.0000
6 H6      3.9561   1.8166   1.3982   H       1 1     0.0000
7 H7      3.2718  -0.3286   2.4652   H       1 1     0.0000
8 H8      5.6163  -0.0003   0.0007   H       1 1     0.0000
9 C9      3.6066   0.1659  -1.2457   C.3     1 1     0.0000
10 H10     3.9572   0.3027  -2.2715   H       1 1     0.0000
11 C11     2.8894   1.3514  -0.5572   C.3     1 1     0.0000
12 H12     3.2731   2.2992  -0.9475   H       1 1     0.0000
13 C13     2.8894  -1.1584  -0.8915   C.3     1 1     0.0000
14 H14     3.2724  -1.9711  -1.5165   H       1 1     0.0000
15 C15     1.3458   1.3335  -0.5503   C.3     1 1     0.0000
16 H16     0.9966   2.2913  -0.9451   H       1 1     0.0000
17 C17     0.7844   1.1387   0.8759   C.3     1 1     0.0000
18 H18     1.1391   1.9614   1.5089   H       1 1     0.0000
19 C19     1.3452  -0.1904   1.4293   C.3     1 1     0.0000
20 H20     0.9958  -0.3276   2.4561   H       1 1     0.0000
21 C21     0.7846   0.1896  -1.4245   C.3     1 1     0.0000
22 H22     1.1398   0.3271  -2.4532   H       1 1     0.0000
23 C23     1.3458  -1.1430  -0.8800   C.3     1 1     0.0000
```

```
24 H24      0.9969  -1.9642  -1.5116   H        1 1      0.0000
25 C25      0.7845  -1.3287   0.5476   C.3      1 1      0.0000
26 H26      1.1396  -2.2882   0.9430   H        1 1      0.0000
27 C27     -0.7844   1.1387   0.8759   C.3      1 1      0.0000
28 H28     -1.1391   1.9614   1.5089   H        1 1      0.0000
29 C29     -0.7845   0.1896  -1.4245   C.3      1 1      0.0000
30 H30     -1.1398   0.3271  -2.4532   H        1 1      0.0000
31 C31     -0.7845  -1.3287   0.5476   C.3      1 1      0.0000
32 H32     -1.1396  -2.2882   0.9430   H        1 1      0.0000
33 C33     -1.3452  -0.1904   1.4293   C.3      1 1      0.0000
34 H34     -0.9958  -0.3276   2.4561   H        1 1      0.0000
35 C35     -1.3458  -1.1430  -0.8800   C.3      1 1      0.0000
36 H36     -0.9969  -1.9642  -1.5116   H        1 1      0.0000
37 C37     -2.8888  -0.1928   1.4490   C.3      1 1      0.0000
38 H38     -3.2719  -0.3286   2.4652   H        1 1      0.0000
39 C39     -3.6062  -1.1620   0.4796   C.3      1 1      0.0000
40 H40     -3.9562  -2.1188   0.8743   H        1 1      0.0000
41 C41     -2.8894  -1.1584  -0.8915   C.3      1 1      0.0000
42 H42     -3.2724  -1.9711  -1.5165   H        1 1      0.0000
43 C43     -1.3458   1.3335  -0.5503   C.3      1 1      0.0000
44 H44     -0.9966   2.2913  -0.9451   H        1 1      0.0000
45 C45     -2.8894   1.3514  -0.5572   C.3      1 1      0.0000
46 H46     -3.2731   2.2992  -0.9475   H        1 1      0.0000
47 C47     -3.6066   0.1659  -1.2457   C.3      1 1      0.0000
48 H48     -3.9572   0.3027  -2.2715   H        1 1      0.0000
49 C49     -3.6063   0.9962   0.7670   C.3      1 1      0.0000
50 H50     -3.9561   1.8166   1.3982   H        1 1      0.0000
51 C51     -4.5273  -0.0001   0.0005   C.3      1 1      0.0000
52 H52     -5.6163  -0.0003   0.0007   H        1 1      0.0000
@<TRIPOS>BOND
1     1     2    1
2     1     3    1
3     1     6    1
4     1    11    1
5     2     4    1
6     2     8    1
7     2     9    1
8     3     4    1
9     3     7    1
10    3    19    1
11    4     5    1
12    4    13    1
13    9    10    1
14    9    11    1
15    9    13    1
16   11    12    1
17   11    15    1
18   13    14    1
19   13    23    1
20   15    16    1
21   15    17    1
22   15    21    1
23   17    18    1
24   17    19    1
25   17    27    1
```

```
26    19    20    1
27    19    25    1
28    21    22    1
29    21    23    1
30    21    29    1
31    23    24    1
32    23    25    1
33    25    26    1
34    25    31    1
35    27    28    1
36    27    33    1
37    27    43    1
38    29    30    1
39    29    35    1
40    29    43    1
41    31    32    1
42    31    33    1
43    31    35    1
44    33    34    1
45    33    37    1
46    35    36    1
47    35    41    1
48    37    38    1
49    37    39    1
50    37    49    1
51    39    40    1
52    39    41    1
53    39    51    1
54    41    42    1
55    41    47    1
56    43    44    1
57    43    45    1
58    45    46    1
59    45    47    1
60    45    49    1
61    47    48    1
62    47    51    1
63    49    50    1
64    49    51    1
65    51    52    1
@<TRIPOS>SUBSTRUCTURE
1 RES1        1 GROUP             0 ****  ****    0
```

Figure S15. Chemical structure of nugget$_{26c}$ generated by the Blink software. Cartesian coordinates of its atoms; the first line contains the total charge and multiplicity; the following lines contain the atomic numbers, followed by the x, y, and z coordinates in Å for each one of the atoms. Next, atomic coordinates in Tripos Mol2 file format (.mol2) with the distances also in Å.

Nugget$_{28a}$ ($C_{28}H_{28}$)

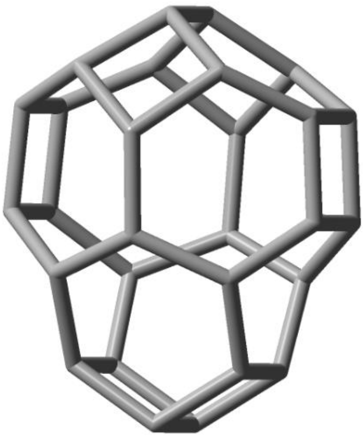

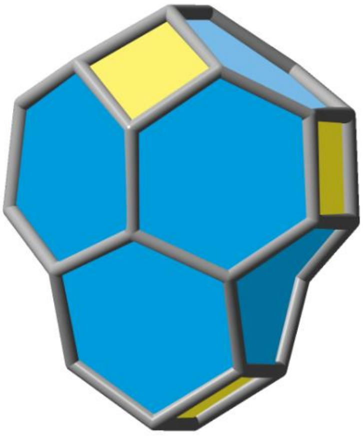

Cartesian Coordinates (Å)

0 1

6	0.426	0.000	-2.018
6	-2.017	1.093	-1.538
6	0.152	2.372	-0.774
6	-1.393	2.255	-0.781
6	1.029	1.363	-1.529
6	-1.117	0.000	-2.195
1	-2.663	1.512	-2.314
1	0.463	3.359	-1.134
1	0.787	0.000	-3.048
1	-1.847	3.181	-1.146
1	1.247	1.867	-2.476
1	-1.294	0.000	-3.274
6	1.029	-1.363	-1.529
1	1.247	-1.867	-2.476
6	-2.017	-1.093	-1.538
1	-2.663	-1.512	-2.314
6	0.152	-2.372	-0.774
1	0.463	-3.359	-1.134
6	2.370	1.295	-0.783
1	2.978	2.138	-1.128
6	3.205	0.000	-0.783
1	4.115	0.000	-1.387
6	2.370	-1.295	-0.783
1	2.978	-2.138	-1.128
6	-2.816	0.000	-0.766
1	-3.862	0.000	-1.084
6	2.370	-1.295	0.783
1	2.978	-2.138	1.128
6	1.029	-1.363	1.529
1	1.247	-1.867	2.476
6	0.152	-2.372	0.774
1	0.463	-3.359	1.134
6	-2.816	0.000	0.766

1	-3.862	0.000	1.084
6	-2.017	1.093	1.538
1	-2.663	1.512	2.314
6	-1.393	2.255	0.781
1	-1.847	3.181	1.146
6	0.152	2.372	0.774
1	0.463	3.359	1.134
6	2.370	1.295	0.783
1	2.978	2.138	1.128
6	1.029	1.363	1.529
1	1.247	1.867	2.476
6	3.205	0.000	0.783
1	4.115	0.000	1.387
6	-1.393	-2.255	0.781
1	-1.847	-3.181	1.146
6	-2.017	-1.093	1.538
1	-2.663	-1.512	2.314
6	-1.393	-2.255	-0.781
1	-1.847	-3.181	-1.146
6	0.426	0.000	2.018
1	0.787	0.000	3.048
6	-1.117	0.000	2.195
1	-1.294	0.000	3.274

.mol2 file

```
@<TRIPOS>MOLECULE
Molecule Name
   56    70     1     0     0
SMALL
NO_CHARGES
****
Generated from the CSD

@<TRIPOS>ATOM
     1 C1      0.4265   0.0000  -2.0182   C.3      1 1      0.0000
     2 C2     -2.0167   1.0927  -1.5376   C.3      1 1      0.0000
     3 C3      0.1522   2.3723  -0.7741   C.3      1 1      0.0000
     4 C4     -1.3931   2.2554  -0.7806   C.3      1 1      0.0000
     5 C5      1.0294   1.3634  -1.5286   C.3      1 1      0.0000
     6 C6     -1.1170   0.0000  -2.1949   C.3      1 1      0.0000
     7 H7     -2.6626   1.5120  -2.3145   H        1 1      0.0000
     8 H8      0.4631   3.3591  -1.1343   H        1 1      0.0000
     9 H9      0.7874   0.0000  -3.0482   H        1 1      0.0000
    10 H10    -1.8467   3.1811  -1.1459   H        1 1      0.0000
    11 H11     1.2469   1.8675  -2.4763   H        1 1      0.0000
    12 H12    -1.2941   0.0000  -3.2742   H        1 1      0.0000
    13 C13     1.0294  -1.3634  -1.5286   C.3      1 1      0.0000
    14 H14     1.2468  -1.8675  -2.4763   H        1 1      0.0000
    15 C15    -2.0167  -1.0927  -1.5376   C.3      1 1      0.0000
```

```
16 H16     -2.6626  -1.5120  -2.3145   H         1 1      0.0000
17 C17      0.1522  -2.3723  -0.7740   C.3       1 1      0.0000
18 H18      0.4630  -3.3591  -1.1343   H         1 1      0.0000
19 C19      2.3703   1.2951  -0.7827   C.3       1 1      0.0000
20 H20      2.9785   2.1377  -1.1280   H         1 1      0.0000
21 C21      3.2046   0.0000  -0.7826   C.3       1 1      0.0000
22 H22      4.1152   0.0000  -1.3869   H         1 1      0.0000
23 C23      2.3703  -1.2951  -0.7827   C.3       1 1      0.0000
24 H24      2.9785  -2.1376  -1.1280   H         1 1      0.0000
25 C25     -2.8158   0.0000  -0.7663   C.3       1 1      0.0000
26 H26     -3.8624   0.0000  -1.0837   H         1 1      0.0000
27 C27      2.3703  -1.2951   0.7827   C.3       1 1      0.0000
28 H28      2.9785  -2.1377   1.1280   H         1 1      0.0000
29 C29      1.0294  -1.3634   1.5286   C.3       1 1      0.0000
30 H30      1.2468  -1.8675   2.4763   H         1 1      0.0000
31 C31      0.1522  -2.3723   0.7740   C.3       1 1      0.0000
32 H32      0.4631  -3.3591   1.1343   H         1 1      0.0000
33 C33     -2.8158   0.0000   0.7663   C.3       1 1      0.0000
34 H34     -3.8624   0.0000   1.0837   H         1 1      0.0000
35 C35     -2.0166   1.0927   1.5376   C.3       1 1      0.0000
36 H36     -2.6626   1.5120   2.3144   H         1 1      0.0000
37 C37     -1.3930   2.2554   0.7806   C.3       1 1      0.0000
38 H38     -1.8467   3.1811   1.1459   H         1 1      0.0000
39 C39      0.1522   2.3723   0.7740   C.3       1 1      0.0000
40 H40      0.4631   3.3591   1.1342   H         1 1      0.0000
41 C41      2.3703   1.2951   0.7827   C.3       1 1      0.0000
42 H42      2.9785   2.1376   1.1280   H         1 1      0.0000
43 C43      1.0294   1.3634   1.5286   C.3       1 1      0.0000
44 H44      1.2468   1.8675   2.4763   H         1 1      0.0000
45 C45      3.2046   0.0000   0.7826   C.3       1 1      0.0000
46 H46      4.1152   0.0000   1.3869   H         1 1      0.0000
47 C47     -1.3931  -2.2554   0.7806   C.3       1 1      0.0000
48 H48     -1.8468  -3.1811   1.1460   H         1 1      0.0000
49 C49     -2.0167  -1.0927   1.5376   C.3       1 1      0.0000
50 H50     -2.6626  -1.5120   2.3144   H         1 1      0.0000
51 C51     -1.3931  -2.2553  -0.7806   C.3       1 1      0.0000
52 H52     -1.8468  -3.1811  -1.1459   H         1 1      0.0000
53 C53      0.4265   0.0000   2.0182   C.3       1 1      0.0000
54 H54      0.7874   0.0000   3.0482   H         1 1      0.0000
55 C55     -1.1170   0.0000   2.1949   C.3       1 1      0.0000
56 H56     -1.2940   0.0000   3.2742   H         1 1      0.0000
@<TRIPOS>BOND
  1    1    5    1
  2    1    6    1
  3    1    9    1
  4    1   13    1
  5    2    4    1
  6    2    6    1
  7    2    7    1
  8    2   25    1
```

9	3	4	1
10	3	5	1
11	3	8	1
12	3	39	1
13	4	10	1
14	4	37	1
15	5	11	1
16	5	19	1
17	6	12	1
18	6	15	1
19	13	14	1
20	13	17	1
21	13	23	1
22	15	16	1
23	15	25	1
24	15	51	1
25	17	18	1
26	17	31	1
27	17	51	1
28	19	20	1
29	19	21	1
30	19	41	1
31	21	22	1
32	21	23	1
33	21	45	1
34	23	24	1
35	23	27	1
36	25	26	1
37	25	33	1
38	27	28	1
39	27	29	1
40	27	45	1
41	29	30	1
42	29	31	1
43	29	53	1
44	31	32	1
45	31	47	1
46	33	34	1
47	33	35	1
48	33	49	1
49	35	36	1
50	35	37	1
51	35	55	1
52	37	38	1
53	37	39	1
54	39	40	1
55	39	43	1
56	41	42	1
57	41	43	1
58	41	45	1

```
  59   43   44   1
  60   43   53   1
  61   45   46   1
  62   47   48   1
  63   47   49   1
  64   47   51   1
  65   49   50   1
  66   49   55   1
  67   51   52   1
  68   53   54   1
  69   53   55   1
  70   55   56   1
@<TRIPOS>SUBSTRUCTURE
     1 RES1        1 GROUP             0 ****  ****    0
```

Figure S16. Chemical structure of $nugget_{28a}$ generated by the Blink software. Cartesian coordinates of its atoms; the first line contains the total charge and multiplicity; the following lines contain the atomic numbers, followed by the x, y, and z coordinates in Å for each one of the atoms. Next, atomic coordinates in Tripos Mol2 file format (.mol2) with the distances also in Å.

Nugget$_{28b}$ ($C_{28}H_{28}$)

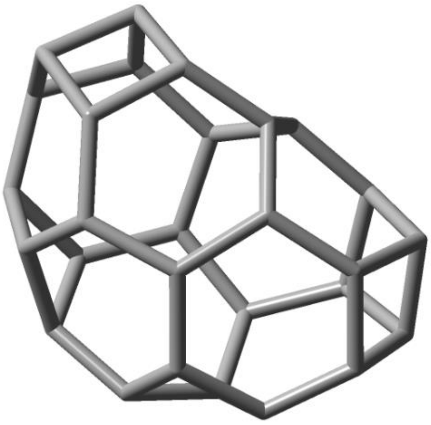

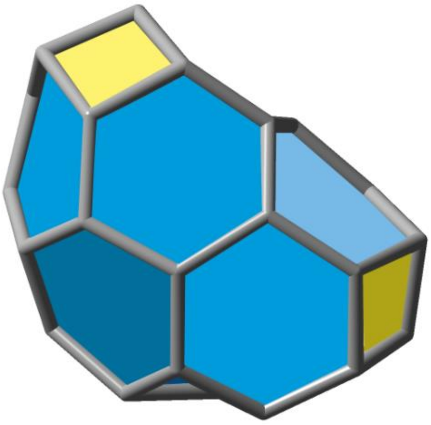

Cartesian Coordinates (Å)

0 1

6	0.031	3.182	0.061
6	-0.676	2.490	-1.138
6	1.386	3.074	-0.657
6	0.672	2.525	-1.925
1	0.649	3.054	-2.881
1	-0.288	4.228	0.116
1	2.024	3.961	-0.659
1	-1.392	3.182	-1.591
6	1.502	1.235	-1.845
1	2.286	1.239	-2.610
6	2.129	1.711	-0.496
1	3.202	1.864	-0.643
6	-0.059	2.536	1.417
1	-0.141	3.308	2.190
6	1.954	0.862	0.793
1	2.936	0.887	1.273
6	-1.124	1.448	1.710
1	-1.951	1.816	2.326
6	-0.043	0.748	2.568
1	-0.143	1.121	3.593
6	1.767	-0.692	0.517
1	2.799	-1.050	0.561
6	1.124	-1.448	1.710
1	1.951	-1.816	2.326
6	0.043	-0.748	2.568
1	0.143	-1.121	3.593
6	-1.386	1.122	-0.953
1	-2.374	1.256	-1.409
6	-1.954	-0.862	0.793
1	-2.936	-0.887	1.273
6	-1.063	-1.562	1.860
1	-1.753	-2.040	2.562

6	-1.767	0.692	0.517
1	-2.799	1.050	0.561
6	1.063	1.562	1.860
1	1.753	2.040	2.562
6	0.059	-2.536	1.417
1	0.141	-3.308	2.190
6	0.759	-0.092	-1.900
1	0.950	-0.526	-2.892
6	1.386	-1.122	-0.953
1	2.374	-1.256	-1.409
6	0.676	-2.490	-1.138
1	1.392	-3.182	-1.591
6	-0.031	-3.182	0.061
1	0.288	-4.228	0.116
6	-1.386	-3.074	-0.657
1	-2.024	-3.961	-0.659
6	-2.129	-1.711	-0.496
1	-3.202	-1.864	-0.643
6	-0.672	-2.525	-1.925
1	-0.649	-3.054	-2.881
6	-1.502	-1.235	-1.845
1	-2.286	-1.239	-2.610
6	-0.759	0.092	-1.900
1	-0.950	0.526	-2.892

.mol2 file

```
@<TRIPOS>MOLECULE
Molecule Name
   56    70     1     0     0
SMALL
NO_CHARGES
****
Generated from the CSD

@<TRIPOS>ATOM
     1 C1        0.0307    3.1822    0.0610    C.3        1 1       0.0000
     2 C2       -0.6761    2.4904   -1.1377    C.3        1 1       0.0000
     3 C3        1.3861    3.0741   -0.6573    C.3        1 1       0.0000
     4 C4        0.6718    2.5249   -1.9253    C.3        1 1       0.0000
     5 H5        0.6491    3.0536   -2.8813    H          1 1       0.0000
     6 H6       -0.2883    4.2279    0.1157    H          1 1       0.0000
     7 H7        2.0242    3.9613   -0.6590    H          1 1       0.0000
     8 H8       -1.3916    3.1817   -1.5915    H          1 1       0.0000
     9 C9        1.5024    1.2352   -1.8453    C.3        1 1       0.0000
    10 H10       2.2860    1.2387   -2.6101    H          1 1       0.0000
    11 C11       2.1291    1.7112   -0.4958    C.3        1 1       0.0000
    12 H12       3.2018    1.8644   -0.6435    H          1 1       0.0000
    13 C13      -0.0585    2.5363    1.4170    C.3        1 1       0.0000
    14 H14      -0.1411    3.3082    2.1898    H          1 1       0.0000
```

```
  15 C15      1.9535   0.8621   0.7935   C.3       1 1      0.0000
  16 H16      2.9356   0.8869   1.2726   H         1 1      0.0000
  17 C17     -1.1239   1.4479   1.7097   C.3       1 1      0.0000
  18 H18     -1.9508   1.8161   2.3260   H         1 1      0.0000
  19 C19     -0.0427   0.7480   2.5678   C.3       1 1      0.0000
  20 H20     -0.1428   1.1210   3.5926   H         1 1      0.0000
  21 C21      1.7672  -0.6918   0.5167   C.3       1 1      0.0000
  22 H22      2.7987  -1.0496   0.5613   H         1 1      0.0000
  23 C23      1.1239  -1.4479   1.7097   C.3       1 1      0.0000
  24 H24      1.9508  -1.8161   2.3260   H         1 1      0.0000
  25 C25      0.0427  -0.7480   2.5678   C.3       1 1      0.0000
  26 H26      0.1428  -1.1210   3.5926   H         1 1      0.0000
  27 C27     -1.3861   1.1218  -0.9531   C.3       1 1      0.0000
  28 H28     -2.3743   1.2564  -1.4088   H         1 1      0.0000
  29 C29     -1.9535  -0.8621   0.7935   C.3       1 1      0.0000
  30 H30     -2.9356  -0.8869   1.2726   H         1 1      0.0000
  31 C31     -1.0626  -1.5622   1.8599   C.3       1 1      0.0000
  32 H32     -1.7531  -2.0404   2.5618   H         1 1      0.0000
  33 C33     -1.7672   0.6918   0.5167   C.3       1 1      0.0000
  34 H34     -2.7987   1.0496   0.5613   H         1 1      0.0000
  35 C35      1.0626   1.5622   1.8599   C.3       1 1      0.0000
  36 H36      1.7531   2.0404   2.5618   H         1 1      0.0000
  37 C37      0.0585  -2.5363   1.4170   C.3       1 1      0.0000
  38 H38      0.1411  -3.3082   2.1898   H         1 1      0.0000
  39 C39      0.7586  -0.0924  -1.9001   C.3       1 1      0.0000
  40 H40      0.9500  -0.5265  -2.8920   H         1 1      0.0000
  41 C41      1.3861  -1.1218  -0.9531   C.3       1 1      0.0000
  42 H42      2.3743  -1.2564  -1.4088   H         1 1      0.0000
  43 C43      0.6761  -2.4904  -1.1377   C.3       1 1      0.0000
  44 H44      1.3916  -3.1817  -1.5915   H         1 1      0.0000
  45 C45     -0.0307  -3.1822   0.0610   C.3       1 1      0.0000
  46 H46      0.2883  -4.2279   0.1157   H         1 1      0.0000
  47 C47     -1.3861  -3.0741  -0.6573   C.3       1 1      0.0000
  48 H48     -2.0242  -3.9613  -0.6590   H         1 1      0.0000
  49 C49     -2.1291  -1.7112  -0.4958   C.3       1 1      0.0000
  50 H50     -3.2018  -1.8644  -0.6435   H         1 1      0.0000
  51 C51     -0.6718  -2.5249  -1.9253   C.3       1 1      0.0000
  52 H52     -0.6491  -3.0536  -2.8813   H         1 1      0.0000
  53 C53     -1.5024  -1.2352  -1.8453   C.3       1 1      0.0000
  54 H54     -2.2860  -1.2387  -2.6101   H         1 1      0.0000
  55 C55     -0.7586   0.0924  -1.9001   C.3       1 1      0.0000
  56 H56     -0.9500   0.5265  -2.8920   H         1 1      0.0000
@<TRIPOS>BOND
     1     1     2    1
     2     1     3    1
     3     1     6    1
     4     1    13    1
     5     2     4    1
     6     2     8    1
     7     2    27    1
```

8	3	4	1
9	3	7	1
10	3	11	1
11	4	5	1
12	4	9	1
13	9	10	1
14	9	11	1
15	9	39	1
16	11	12	1
17	11	15	1
18	13	14	1
19	13	17	1
20	13	35	1
21	15	16	1
22	15	21	1
23	15	35	1
24	17	18	1
25	17	19	1
26	17	33	1
27	19	20	1
28	19	25	1
29	19	35	1
30	21	22	1
31	21	23	1
32	21	41	1
33	23	24	1
34	23	25	1
35	23	37	1
36	25	26	1
37	25	31	1
38	27	28	1
39	27	33	1
40	27	55	1
41	29	30	1
42	29	31	1
43	29	33	1
44	29	49	1
45	31	32	1
46	31	37	1
47	33	34	1
48	35	36	1
49	37	38	1
50	37	45	1
51	39	40	1
52	39	41	1
53	39	55	1
54	41	42	1
55	41	43	1
56	43	44	1
57	43	45	1

```
  58   43   51   1
  59   45   46   1
  60   45   47   1
  61   47   48   1
  62   47   49   1
  63   47   51   1
  64   49   50   1
  65   49   53   1
  66   51   52   1
  67   51   53   1
  68   53   54   1
  69   53   55   1
  70   55   56   1
@<TRIPOS>SUBSTRUCTURE
     1 RES1        1 GROUP             0 ****  ****    0
```

Figure S17. Chemical structure of $nugget_{28b}$ generated by the Blink software. Cartesian coordinates of its atoms; the first line contains the total charge and multiplicity; the following lines contain the atomic numbers, followed by the x, y, and z coordinates in Å for each one of the atoms. Next, atomic coordinates in Tripos Mol2 file format (.mol2) with the distances also in Å.

Nugget$_{28c}$ ($C_{28}H_{28}$)

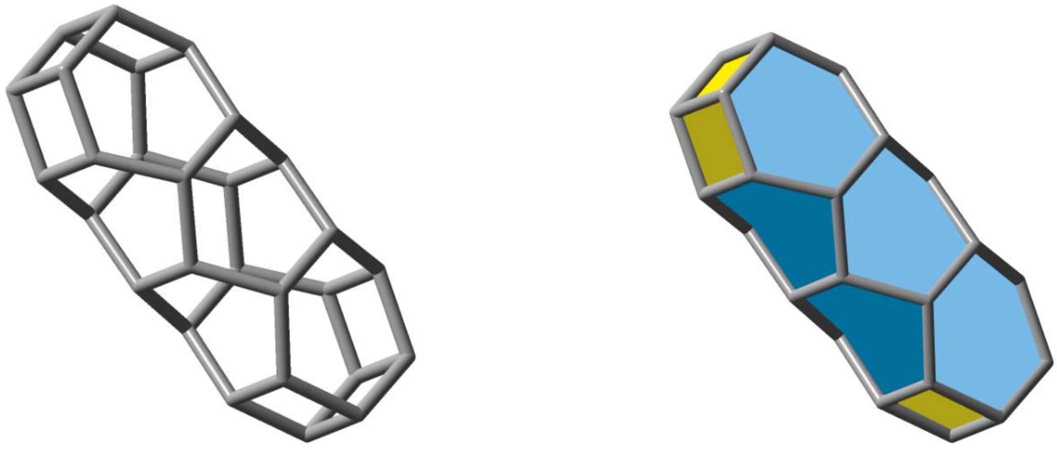

Cartesian Coordinates (Å)

0 1

6	-3.867	0.774	0.780
6	-3.867	0.774	-0.780
6	-2.480	1.459	0.779
6	-2.480	1.459	-0.779
1	-2.541	2.480	-1.171
1	-4.652	1.253	1.369
1	-2.541	2.480	1.171
1	-4.652	1.253	-1.369
6	-3.867	-0.774	-0.780
1	-4.652	-1.253	-1.369
6	-3.867	-0.774	0.780
1	-4.652	-1.253	1.369
6	-2.480	-1.459	-0.779
1	-2.541	-2.480	-1.171
6	-2.480	-1.459	0.779
1	-2.541	-2.480	1.171
6	-1.271	-0.780	1.420
1	-1.252	-1.082	2.475
6	-1.271	0.780	1.420
1	-1.252	1.082	2.475
6	-1.271	0.780	-1.420
1	-1.252	1.082	-2.475
6	-1.271	-0.780	-1.420
1	-1.252	-1.082	-2.475
6	0.000	-1.381	-0.787
1	0.000	-2.440	-1.082
6	0.000	-1.381	0.787
1	0.000	-2.440	1.082
6	0.000	1.381	0.787
1	0.000	2.440	1.082

6	0.000	1.381	-0.787
1	0.000	2.440	-1.082
6	1.271	-0.780	1.420
1	1.252	-1.082	2.475
6	1.271	0.780	1.420
1	1.252	1.082	2.475
6	1.271	0.780	-1.420
1	1.252	1.082	-2.475
6	1.271	-0.780	-1.420
1	1.252	-1.082	-2.475
6	2.480	1.459	0.779
1	2.541	2.480	1.171
6	2.480	1.459	-0.779
1	2.541	2.480	-1.171
6	2.480	-1.459	-0.779
1	2.541	-2.480	-1.171
6	2.480	-1.459	0.779
1	2.541	-2.480	1.171
6	3.867	0.774	0.780
1	4.652	1.253	1.369
6	3.867	-0.774	0.780
1	4.652	-1.253	1.369
6	3.867	-0.774	-0.780
1	4.652	-1.253	-1.369
6	3.867	0.774	-0.780
1	4.652	1.253	-1.369

.mol2 file

```
@<TRIPOS>MOLECULE
Molecule Name
   56    70     1     0     0
SMALL
NO_CHARGES
****
Generated from the CSD

@<TRIPOS>ATOM
     1 C1      -3.8667   0.7742   0.7797   C.3       1 1      0.0000
     2 C2      -3.8667   0.7742  -0.7797   C.3       1 1      0.0000
     3 C3      -2.4802   1.4593   0.7789   C.3       1 1      0.0000
     4 C4      -2.4802   1.4593  -0.7789   C.3       1 1      0.0000
     5 H5      -2.5412   2.4804  -1.1711   H         1 1      0.0000
     6 H6      -4.6524   1.2528   1.3689   H         1 1      0.0000
     7 H7      -2.5412   2.4804   1.1711   H         1 1      0.0000
     8 H8      -4.6524   1.2528  -1.3689   H         1 1      0.0000
     9 C9      -3.8667  -0.7742  -0.7797   C.3       1 1      0.0000
    10 H10     -4.6524  -1.2528  -1.3689   H         1 1      0.0000
    11 C11     -3.8667  -0.7742   0.7797   C.3       1 1      0.0000
    12 H12     -4.6524  -1.2528   1.3689   H         1 1      0.0000
```

```
 13 C13     -2.4802  -1.4593  -0.7789   C.3       1 1      0.0000
 14 H14     -2.5412  -2.4804  -1.1711   H         1 1      0.0000
 15 C15     -2.4802  -1.4593   0.7789   C.3       1 1      0.0000
 16 H16     -2.5412  -2.4804   1.1711   H         1 1      0.0000
 17 C17     -1.2707  -0.7799   1.4201   C.3       1 1      0.0000
 18 H18     -1.2524  -1.0823   2.4749   H         1 1      0.0000
 19 C19     -1.2707   0.7799   1.4201   C.3       1 1      0.0000
 20 H20     -1.2524   1.0823   2.4749   H         1 1      0.0000
 21 C21     -1.2707   0.7799  -1.4201   C.3       1 1      0.0000
 22 H22     -1.2524   1.0823  -2.4749   H         1 1      0.0000
 23 C23     -1.2707  -0.7799  -1.4201   C.3       1 1      0.0000
 24 H24     -1.2524  -1.0823  -2.4749   H         1 1      0.0000
 25 C25      0.0000  -1.3814  -0.7867   C.3       1 1      0.0000
 26 H26      0.0000  -2.4396  -1.0823   H         1 1      0.0000
 27 C27      0.0000  -1.3814   0.7867   C.3       1 1      0.0000
 28 H28      0.0000  -2.4396   1.0823   H         1 1      0.0000
 29 C29      0.0000   1.3814   0.7867   C.3       1 1      0.0000
 30 H30      0.0000   2.4396   1.0823   H         1 1      0.0000
 31 C31      0.0000   1.3814  -0.7867   C.3       1 1      0.0000
 32 H32      0.0000   2.4396  -1.0823   H         1 1      0.0000
 33 C33      1.2707  -0.7799   1.4201   C.3       1 1      0.0000
 34 H34      1.2524  -1.0823   2.4749   H         1 1      0.0000
 35 C35      1.2707   0.7799   1.4201   C.3       1 1      0.0000
 36 H36      1.2524   1.0823   2.4749   H         1 1      0.0000
 37 C37      1.2707   0.7799  -1.4201   C.3       1 1      0.0000
 38 H38      1.2524   1.0823  -2.4749   H         1 1      0.0000
 39 C39      1.2707  -0.7799  -1.4201   C.3       1 1      0.0000
 40 H40      1.2524  -1.0823  -2.4749   H         1 1      0.0000
 41 C41      2.4802   1.4593   0.7789   C.3       1 1      0.0000
 42 H42      2.5412   2.4804   1.1711   H         1 1      0.0000
 43 C43      2.4802   1.4593  -0.7789   C.3       1 1      0.0000
 44 H44      2.5412   2.4804  -1.1711   H         1 1      0.0000
 45 C45      2.4802  -1.4593  -0.7789   C.3       1 1      0.0000
 46 H46      2.5412  -2.4804  -1.1711   H         1 1      0.0000
 47 C47      2.4802  -1.4593   0.7789   C.3       1 1      0.0000
 48 H48      2.5412  -2.4804   1.1711   H         1 1      0.0000
 49 C49      3.8667   0.7742   0.7797   C.3       1 1      0.0000
 50 H50      4.6524   1.2528   1.3689   H         1 1      0.0000
 51 C51      3.8667  -0.7742   0.7797   C.3       1 1      0.0000
 52 H52      4.6524  -1.2528   1.3689   H         1 1      0.0000
 53 C53      3.8667  -0.7742  -0.7797   C.3       1 1      0.0000
 54 H54      4.6524  -1.2528  -1.3689   H         1 1      0.0000
 55 C55      3.8667   0.7742  -0.7797   C.3       1 1      0.0000
 56 H56      4.6524   1.2528  -1.3689   H         1 1      0.0000
@<TRIPOS>BOND
     1     1     2    1
     2     1     3    1
     3     1     6    1
     4     1    11    1
     5     2     4    1
```

6	2	8	1
7	2	9	1
8	3	4	1
9	3	7	1
10	3	19	1
11	4	5	1
12	4	21	1
13	9	10	1
14	9	11	1
15	9	13	1
16	11	12	1
17	11	15	1
18	13	14	1
19	13	15	1
20	13	23	1
21	15	16	1
22	15	17	1
23	17	18	1
24	17	19	1
25	17	27	1
26	19	20	1
27	19	29	1
28	21	22	1
29	21	23	1
30	21	31	1
31	23	24	1
32	23	25	1
33	25	26	1
34	25	27	1
35	25	39	1
36	27	28	1
37	27	33	1
38	29	30	1
39	29	31	1
40	29	35	1
41	31	32	1
42	31	37	1
43	33	34	1
44	33	35	1
45	33	47	1
46	35	36	1
47	35	41	1
48	37	38	1
49	37	39	1
50	37	43	1
51	39	40	1
52	39	45	1
53	41	42	1
54	41	43	1
55	41	49	1

```
  56   43   44   1
  57   43   55   1
  58   45   46   1
  59   45   47   1
  60   45   53   1
  61   47   48   1
  62   47   51   1
  63   49   50   1
  64   49   51   1
  65   49   55   1
  66   51   52   1
  67   51   53   1
  68   53   54   1
  69   53   55   1
  70   55   56   1
@<TRIPOS>SUBSTRUCTURE
   1 RES1       1 GROUP         0 ****  ****    0
```

Figure S18. Chemical structure of nugget$_{28c}$ generated by the Blink software. Cartesian coordinates of its atoms; the first line contains the total charge and multiplicity; the following lines contain the atomic numbers, followed by the x, y, and z coordinates in Å for each one of the atoms. Next, atomic coordinates in Tripos Mol2 file format (.mol2) with the distances also in Å.

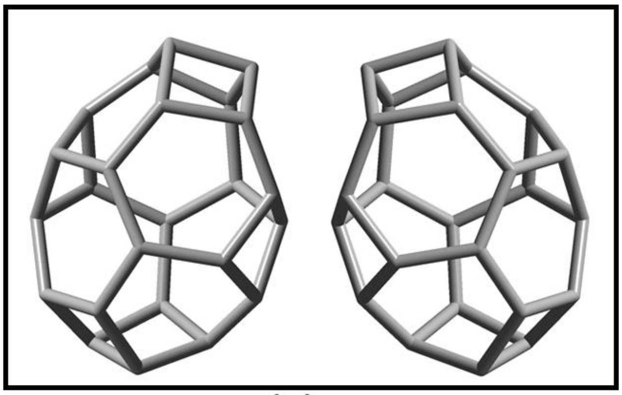

(a)

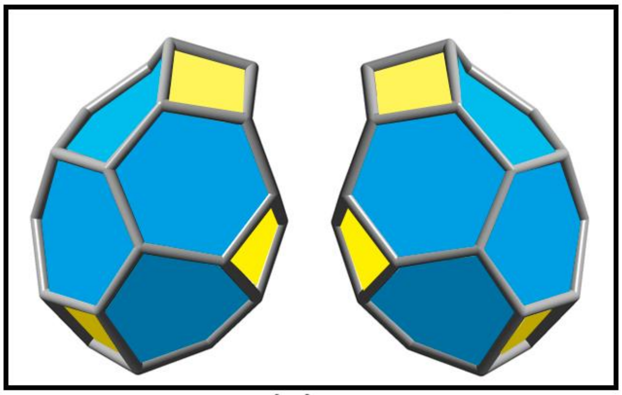

(a)

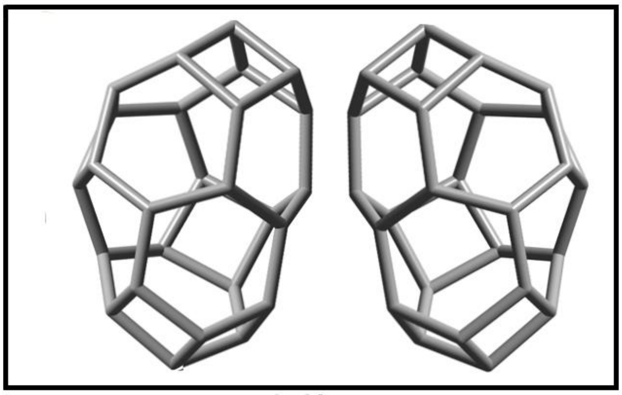

(b)

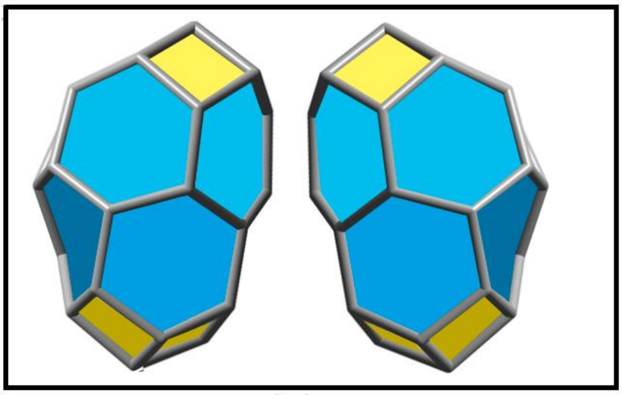

(b)

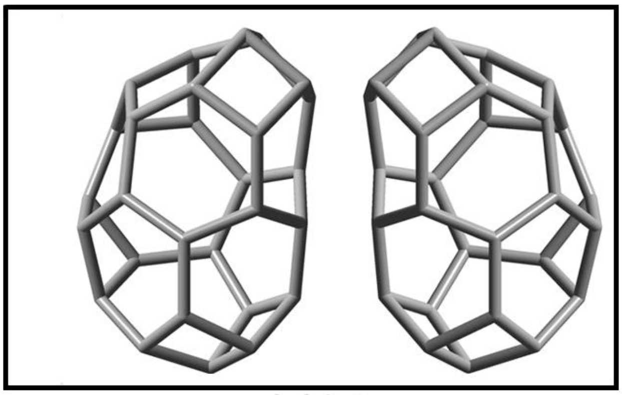

(c)

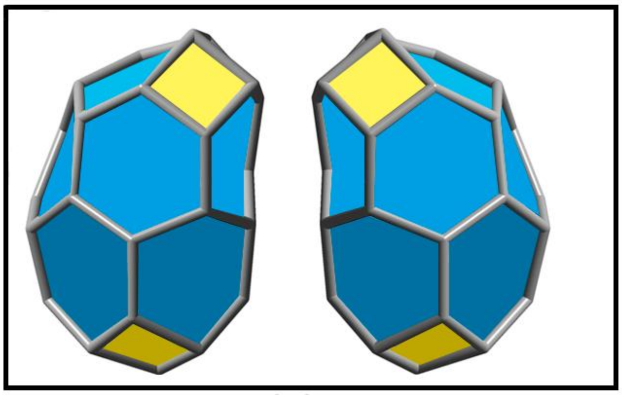

(c)

(a) Cartesian Coordinates to the left chiral compound (Å)

0 1			
6	-0.357	1.918	-1.030
6	1.005	2.176	-0.352
6	-1.005	2.176	0.352
6	0.357	1.918	1.030
1	0.636	2.683	1.762
1	-0.636	2.683	-1.762
1	-1.234	3.246	0.416
1	1.234	3.246	-0.416
6	2.211	1.369	-0.746
1	2.908	1.982	-1.325
6	-0.577	0.551	-1.726
6	2.014	-0.003	1.474
1	2.528	-0.031	2.438
6	0.577	0.551	1.726
1	0.580	0.837	2.781
1	-0.580	0.837	-2.781
6	0.577	-0.551	-1.726
1	0.580	-0.837	-2.781
6	2.014	0.003	-1.474
1	2.528	0.031	-2.438
6	-2.014	-0.003	-1.474
1	-2.528	-0.031	-2.438
6	-2.941	0.664	-0.402
1	-3.846	1.133	-0.796
6	-2.211	1.369	0.746
1	-2.908	1.982	1.325
6	-0.577	-0.551	1.726
1	-0.580	-0.837	2.781
6	0.357	-1.918	-1.030
1	0.636	-2.683	-1.762
6	-1.005	-2.176	-0.352
1	-1.234	-3.246	-0.416
6	-2.211	-1.369	-0.746
1	-2.908	-1.982	-1.325
6	-2.941	-0.664	0.402
1	-3.846	-1.133	0.796
6	2.211	-1.369	0.746
1	2.908	-1.982	1.325
6	2.941	-0.664	-0.402
1	3.846	-1.133	-0.796
6	2.941	0.664	0.402
1	3.846	1.133	0.796
6	-2.014	0.003	1.474
1	-2.528	0.031	2.438
6	1.005	-2.176	0.352
1	1.234	-3.246	0.416
6	-0.357	-1.918	1.030

1 -0.635 -2.683 1.762

.mol2 file

@<TRIPOS>MOLECULE
Molecule Name
48 60
SMALL
NO_CHARGES

@<TRIPOS>ATOM
1 C1 -0.3568 1.9182 -1.0304 C
2 C2 1.0053 2.1763 -0.3518 C
3 C3 -1.0053 2.1763 0.3519 C
4 C4 0.3568 1.9182 1.0304 C
5 H5 0.6356 2.6833 1.7622 H
6 H6 -0.6356 2.6834 -1.7621 H
7 H7 -1.2341 3.2457 0.4162 H
8 H8 1.2342 3.2457 -0.4161 H
9 C9 2.2105 1.3689 -0.7457 C
10 H10 2.9079 1.9821 -1.3254 H
11 C11 -0.5767 0.5507 -1.7264 C
12 C12 2.0138 -0.0025 1.4736 C
13 H13 2.5283 -0.0307 2.4376 H
14 C14 0.5767 0.5507 1.7264 C
15 H15 0.5801 0.8367 2.7812 H
16 H16 -0.5801 0.8368 -2.7812 H
17 C17 0.5767 -0.5507 -1.7264 C
18 H18 0.5801 -0.8367 -2.7812 H
19 C19 2.0139 0.0025 -1.4736 C
20 H20 2.5283 0.0307 -2.4377 H
21 C21 -2.0138 -0.0025 -1.4736 C
22 H22 -2.5283 -0.0307 -2.4377 H
23 C23 -2.9410 0.6645 -0.4023 C
24 H24 -3.8459 1.1333 -0.7965 H
25 C25 -2.2105 1.3689 0.7456 C
26 H26 -2.9079 1.9821 1.3254 H
27 C27 -0.5767 -0.5507 1.7264 C
28 H28 -0.5801 -0.8368 2.7812 H
29 C29 0.3568 -1.9182 -1.0304 C
30 H30 0.6356 -2.6833 -1.7622 H
31 C31 -1.0053 -2.1763 -0.3519 C
32 H32 -1.2341 -3.2457 -0.4162 H
33 C33 -2.2105 -1.3689 -0.7457 C
34 H34 -2.9079 -1.9821 -1.3254 H
35 C35 -2.9410 -0.6645 0.4023 C
36 H36 -3.8459 -1.1333 0.7965 H
37 C37 2.2105 -1.3689 0.7457 C
38 H38 2.9079 -1.9821 1.3254 H
39 C39 2.9410 -0.6645 -0.4022 C

40 H40 3.8459 -1.1332 -0.7965 H
41 C41 2.9410 0.6645 0.4022 C
42 H42 3.8459 1.1333 0.7965 H
43 C43 -2.0138 0.0025 1.4736 C
44 H44 -2.5283 0.0307 2.4377 H
45 C45 1.0053 -2.1763 0.3518 C
46 H46 1.2341 -3.2457 0.4161 H
47 C47 -0.3568 -1.9182 1.0304 C
48 H48 -0.6355 -2.6834 1.7621 H
@<TRIPOS>BOND
1 1 2 1
2 1 3 1
3 1 6 1
4 1 11 1
5 2 4 1
6 2 8 1
7 2 9 1
8 3 4 1
9 3 7 1
10 3 25 1
11 4 5 1
12 4 14 1
13 9 10 1
14 9 19 1
15 9 41 1
16 11 16 1
17 11 17 1
18 11 21 1
19 12 13 1
20 12 14 1
21 12 37 1
22 12 41 1
23 14 15 1
24 14 27 1
25 17 18 1
26 17 19 1
27 17 29 1
28 19 20 1
29 19 39 1
30 21 22 1
31 21 23 1
32 21 33 1
33 23 24 1
34 23 25 1
35 23 35 1
36 25 26 1
37 25 43 1
38 27 28 1
39 27 43 1
40 27 47 1

41 29 30 1
42 29 31 1
43 29 45 1
44 31 32 1
45 31 33 1
46 31 47 1
47 33 34 1
48 33 35 1
49 35 36 1
50 35 43 1
51 37 38 1
52 37 39 1
53 37 45 1
54 39 40 1
55 39 41 1
56 41 42 1
57 43 44 1
58 45 46 1
59 45 47 1
60 47 48 1

(a) Cartesian Coordinates to the right chiral compound (Å)

0 1

1	0.291	-2.164	-3.392
6	0.350	-1.681	-2.415
6	0.990	-2.484	-1.277
6	-0.956	-1.565	-1.592
6	-0.367	-2.578	-0.604
1	-0.825	-3.567	-0.744
1	-1.763	-2.067	-2.155
1	1.503	-3.409	-1.549
6	1.387	-0.592	-2.207
1	2.174	-0.626	-2.973
6	1.865	-1.260	-0.912
1	2.925	-1.543	-1.044
6	-0.316	-2.211	0.851
1	-0.427	-3.111	1.477
6	0.902	-1.375	1.312
1	1.580	-2.045	1.871
6	1.811	-0.548	0.427
1	2.806	-0.677	0.886
6	-1.228	-1.072	1.330
1	-2.050	-1.486	1.941
6	-0.071	-0.695	2.286
1	-0.154	-1.251	3.233
6	-1.905	-0.156	0.333
1	-2.960	-0.477	0.360
6	-1.555	-0.248	-1.133
1	-2.535	-0.164	-1.632
6	-0.731	0.844	-1.783

1	-1.275	1.098	-2.710
6	0.777	0.777	-2.122
1	0.994	1.349	-3.039
6	1.648	0.952	0.373
6	1.057	1.603	-0.859
1	1.745	2.433	-1.102
6	0.126	0.769	2.557
1	0.474	0.935	3.586
6	0.959	1.590	1.565
1	1.766	2.090	2.130
6	-1.868	1.235	0.939
1	-2.928	1.519	1.070
6	-1.146	2.401	0.254
1	-1.824	3.258	0.131
6	-0.391	2.128	-1.015
1	-0.413	3.013	-1.671
6	-0.231	2.572	1.453
1	-0.103	3.572	1.872
6	-1.109	1.593	2.239
1	-1.690	2.001	3.068
1	2.689	1.318	0.385

.mol2 file

```
@<TRIPOS>MOLECULE
Molecule Name
48 60
SMALL
NO_CHARGES


@<TRIPOS>ATOM
1 H1     0.2910    -2.1640    -3.3920 H
2 C2     0.3500    -1.6810    -2.4150 C
3 C3     0.9900    -2.4840    -1.2770 C
4 C4    -0.9560    -1.5650    -1.5920 C
5 C5    -0.3670    -2.5780    -0.6040 C
6 H6    -0.8250    -3.5670    -0.7440 H
7 H7    -1.7630    -2.0670    -2.1550 H
8 H8     1.5030    -3.4090    -1.5490 H
9 C9     1.3870    -0.5920    -2.2070 C
10 H10     2.1740    -0.6260    -2.9730 H
11 C11     1.8650    -1.2600    -0.9120 C
12 H12     2.9250    -1.5430    -1.0440 H
13 C13    -0.3160    -2.2110     0.8510 C
14 H14    -0.4270    -3.1110     1.4770 H
15 C15     0.9020    -1.3750     1.3120 C
16 H16     1.5800    -2.0450     1.8710 H
17 C17     1.8110    -0.5480     0.4270 C
18 H18     2.8060    -0.6770     0.8860 H
19 C19    -1.2280    -1.0720     1.3300 C
```

```
20 H20    -2.0500    -1.4860     1.9410 H
21 C21    -0.0710    -0.6950     2.2860 C
22 H22    -0.1540    -1.2510     3.2330 H
23 C23    -1.9050    -0.1560     0.3330 C
24 H24    -2.9600    -0.4770     0.3600 H
25 C25    -1.5550    -0.2480    -1.1330 C
26 H26    -2.5350    -0.1640    -1.6320 H
27 C27    -0.7310     0.8440    -1.7830 C
28 H28    -1.2750     1.0980    -2.7100 H
29 C29     0.7770     0.7770    -2.1220 C
30 H30     0.9940     1.3490    -3.0390 H
31 C31     1.6480     0.9520     0.3730 C
32 C32     1.0570     1.6030    -0.8590 C
33 H33     1.7450     2.4330    -1.1020 H
34 C34     0.1260     0.7690     2.5570 C
35 H35     0.4740     0.9350     3.5860 H
36 C36     0.9590     1.5900     1.5650 C
37 H37     1.7660     2.0900     2.1300 H
38 C38    -1.8680     1.2350     0.9390 C
39 H39    -2.9280     1.5190     1.0700 H
40 C40    -1.1460     2.4010     0.2540 C
41 H41    -1.8240     3.2580     0.1310 H
42 C42    -0.3910     2.1280    -1.0150 C
43 H43    -0.4130     3.0130    -1.6710 H
44 C44    -0.2310     2.5720     1.4530 C
45 H45    -0.1030     3.5720     1.8720 H
46 C46    -1.1090     1.5930     2.2390 C
47 H47    -1.6900     2.0010     3.0680 H
48 H48     2.6890     1.3180     0.3850 H
@<TRIPOS>BOND
1 1 2 1
2 2 3 1
3 2 4 1
4 2 9 1
5 3 8 1
6 3 5 1
7 3 11 1
8 4 7 1
9 4 5 1
10 4 25 1
11 5 6 1
12 5 13 1
13 9 10 1
14 9 11 1
15 9 29 1
16 11 12 1
17 11 17 1
18 13 14 1
19 13 15 1
20 13 19 1
```

21 15 16 1
22 15 17 1
23 15 21 1
24 17 18 1
25 17 31 1
26 19 20 1
27 19 21 1
28 19 23 1
29 21 22 1
30 21 34 1
31 23 24 1
32 23 25 1
33 23 38 1
34 25 26 1
35 25 27 1
36 27 28 1
37 27 29 1
38 27 42 1
39 29 30 1
40 29 32 1
41 31 32 1
42 31 48 1
43 31 36 1
44 32 33 1
45 32 42 1
46 34 35 1
47 34 36 1
48 34 46 1
49 36 37 1
50 36 44 1
51 38 39 1
52 38 40 1
53 38 46 1
54 40 41 1
55 40 42 1
56 40 44 1
57 42 43 1
58 44 45 1
59 44 46 1
60 46 47 1

(b) Cartesian Coordinates to the left chiral compound (Å)

0 1

6	-0.283	2.248	0.457
6	-0.302	0.714	-1.734
6	-2.566	1.266	-0.403
6	-1.843	0.721	-1.677
6	-1.747	1.827	0.746
6	0.326	1.854	-0.892

1	-0.043	1.037	-2.750
1	-3.305	2.012	-0.712
1	-0.123	3.328	0.558
1	-2.215	1.260	-2.553
1	-2.297	2.655	1.203
1	0.155	2.733	-1.527
6	0.168	1.497	1.735
1	0.271	2.243	2.529
6	-1.252	0.870	1.870
1	-1.698	1.154	2.827
6	-3.245	-0.101	-0.202
1	-4.322	-0.099	-0.017
6	-2.417	-1.128	0.610
1	-3.082	-1.875	1.052
6	-1.462	-0.656	1.736
1	-1.979	-0.926	2.661
6	-0.168	-1.497	1.735
1	-0.271	-2.243	2.529
6	1.252	-0.870	1.870
1	1.698	-1.154	2.827
6	1.462	0.656	1.736
1	1.979	0.926	2.661
6	1.843	1.710	-0.725
1	2.305	2.688	-0.896
6	-1.843	-1.710	-0.725
1	-2.305	-2.688	-0.896
6	-0.326	-1.854	-0.892
1	-0.155	-2.733	-1.527
6	0.283	-2.248	0.457
1	0.123	-3.328	0.558
6	-2.597	-0.627	-1.520
1	-3.175	-0.958	-2.386
6	1.747	-1.827	0.746
1	2.297	-2.655	1.203
6	2.597	0.627	-1.520
1	3.175	0.958	-2.386
6	1.843	-0.721	-1.677
1	2.215	-1.260	-2.553
6	0.302	-0.714	-1.734
1	0.043	-1.037	-2.750
6	3.245	0.101	-0.202
1	4.322	0.099	-0.017
6	2.566	-1.266	-0.403
1	3.305	-2.012	-0.712
6	2.417	1.128	0.610
1	3.082	1.875	1.052

.mol2 file

```
# size26_gem3
@<TRIPOS>MOLECULE
Molecule Name
52 65
SMALL
NO_CHARGES


@<TRIPOS>ATOM
1 C1    -0.2831    2.2482    0.4574 C
2 C2    -0.3022    0.7141   -1.7344 C
3 C3    -2.5658    1.2662   -0.4032 C
4 C4    -1.8426    0.7207   -1.6772 C
5 C5    -1.7467    1.8267    0.7461 C
6 C6     0.3262    1.8539   -0.8922 C
7 H7    -0.0429    1.0373   -2.7499 H
8 H8    -3.3048    2.0118   -0.7124 H
9 H9    -0.1226    3.3278    0.5576 H
10 H10    -2.2147    1.2596   -2.5534 H
11 H11    -2.2971    2.6549    1.2032 H
12 H12     0.1548    2.7331   -1.5267 H
13 C13     0.1678    1.4967    1.7352 C
14 H14     0.2705    2.2433    2.5291 H
15 C15    -1.2521    0.8698    1.8703 C
16 H16    -1.6982    1.1539    2.8274 H
17 C17    -3.2453   -0.1013   -0.2015 C
18 H18    -4.3222   -0.0992   -0.0169 H
19 C19    -2.4165   -1.1283    0.6104 C
20 H20    -3.0824   -1.8754    1.0522 H
21 C21    -1.4625   -0.6560    1.7358 C
22 H22    -1.9788   -0.9264    2.6611 H
23 C23    -0.1678   -1.4967    1.7352 C
24 H24    -0.2705   -2.2432    2.5291 H
25 C25     1.2521   -0.8698    1.8703 C
26 H26     1.6982   -1.1539    2.8274 H
27 C27     1.4625    0.6560    1.7358 C
28 H28     1.9788    0.9264    2.6611 H
29 C29     1.8426    1.7100   -0.7254 C
30 H30     2.3049    2.6877   -0.8959 H
31 C31    -1.8426   -1.7100   -0.7254 C
32 H32    -2.3049   -2.6878   -0.8958 H
33 C33    -0.3261   -1.8539   -0.8922 C
34 H34    -0.1548   -2.7331   -1.5266 H
35 C35     0.2831   -2.2482    0.4574 C
36 H36     0.1226   -3.3278    0.5576 H
37 C37    -2.5967   -0.6269   -1.5197 C
38 H38    -3.1754   -0.9576   -2.3858 H
39 C39     1.7467   -1.8267    0.7461 C
40 H40     2.2972   -2.6548    1.2033 H
```

41 C41 2.5967 0.6269 -1.5197 C
42 H42 3.1754 0.9575 -2.3858 H
43 C43 1.8426 -0.7207 -1.6772 C
44 H44 2.2147 -1.2596 -2.5534 H
45 C45 0.3022 -0.7141 -1.7344 C
46 H46 0.0429 -1.0374 -2.7499 H
47 C47 3.2453 0.1013 -0.2015 C
48 H48 4.3223 0.0992 -0.0169 H
49 C49 2.5658 -1.2663 -0.4032 C
50 H50 3.3048 -2.0118 -0.7123 H
51 C51 2.4165 1.1283 0.6104 C
52 H52 3.0824 1.8754 1.0522 H
@<TRIPOS>BOND
1 1 5 1
2 1 6 1
3 1 9 1
4 1 13 1
5 2 4 1
6 2 6 1
7 2 7 1
8 2 45 1
9 3 4 1
10 3 5 1
11 3 8 1
12 3 17 1
13 4 10 1
14 4 37 1
15 5 11 1
16 5 15 1
17 6 12 1
18 6 29 1
19 13 14 1
20 13 15 1
21 13 27 1
22 15 16 1
23 15 21 1
24 17 18 1
25 17 19 1
26 17 37 1
27 19 20 1
28 19 21 1
29 19 31 1
30 21 22 1
31 21 23 1
32 23 24 1
33 23 25 1
34 23 35 1
35 25 26 1
36 25 27 1
37 25 39 1

38 27 28 1
39 27 51 1
40 29 30 1
41 29 41 1
42 29 51 1
43 31 32 1
44 31 33 1
45 31 37 1
46 33 34 1
47 33 35 1
48 33 45 1
49 35 36 1
50 35 39 1
51 37 38 1
52 39 40 1
53 39 49 1
54 41 42 1
55 41 43 1
56 41 47 1
57 43 44 1
58 43 45 1
59 43 49 1
60 45 46 1
61 47 48 1
62 47 49 1
63 47 51 1
64 49 50 1
65 51 52 1

(b) Cartesian Coordinates to the right chiral compound (Å)

0 1

6	-0.773	-0.219	0.456
1	0.155	0.297	0.759
6	-0.766	-0.255	-1.069
1	0.269	0.022	-1.345
6	-0.847	-1.658	-1.665
1	0.197	-1.998	-1.524
6	-1.605	-2.769	-0.978
1	-1.020	-3.683	-1.172
6	-1.761	-2.617	0.522
1	-1.562	-3.609	0.965
6	-0.894	-1.556	1.205
1	-0.071	-1.877	1.845
6	-2.146	-1.019	1.825
6	-1.946	0.366	1.277
1	-1.584	1.076	2.034
6	-3.052	-2.032	1.172
1	-3.445	-2.755	1.906
6	-1.539	0.896	-1.665
1	-0.875	1.770	-1.577
6	-2.838	1.276	-1.000

1	-2.952	2.360	-1.181
6	-3.085	0.979	0.484
1	-3.512	1.846	1.009
6	-4.191	-0.037	0.095
1	-5.119	0.311	0.590
6	-4.241	-1.522	0.385
1	-5.162	-1.740	0.943
6	-4.184	0.580	-1.333
1	-4.958	1.372	-1.354
6	-4.430	-0.175	-2.610
1	-5.399	0.110	-3.042
6	-4.425	-1.650	-2.326
1	-5.433	-1.946	-2.678
6	-4.243	-2.280	-0.917
1	-5.052	-3.024	-0.778
6	-3.010	-3.053	-1.453
1	-3.167	-4.132	-1.276
6	-3.443	-2.697	-2.883
1	-3.995	-3.525	-3.354
6	-2.350	-2.208	-3.805
1	-2.121	-2.968	-4.566
6	-1.054	-1.682	-3.172
1	-0.201	-2.233	-3.604
6	-1.252	-0.362	-3.932
1	-0.508	-0.082	-4.680
6	-1.928	0.742	-3.119
1	-1.764	1.719	-3.606
6	-2.615	-0.852	-4.434
1	-2.795	-0.814	-5.510
6	-3.317	0.192	-3.559
1	-3.768	0.952	-4.223
1	-2.462	-0.925	2.864

.mol2 file

```
@<TRIPOS>MOLECULE
Molecule Name
52 65
SMALL
NO_CHARGES


@<TRIPOS>ATOM
1 C1    -0.7733    -0.2193     0.4561 C
2 H2     0.1549     0.2966     0.7594 H
3 C3    -0.7659    -0.2553    -1.0695 C
4 H4     0.2694     0.0218    -1.3449 H
5 C5    -0.8465    -1.6576    -1.6648 C
6 H6     0.1968    -1.9983    -1.5244 H
7 C7    -1.6054    -2.7695    -0.9780 C
```

8 H8 -1.0203 -3.6825 -1.1716 H
9 C9 -1.7612 -2.6174 0.5218 C
10 H10 -1.5616 -3.6093 0.9650 H
11 C11 -0.8937 -1.5561 1.2050 C
12 H12 -0.0715 -1.8773 1.8447 H
13 C13 -2.1461 -1.0186 1.8252 C
14 C14 -1.9463 0.3664 1.2767 C
15 H15 -1.5842 1.0756 2.0343 H
16 C16 -3.0517 -2.0319 1.1725 C
17 H17 -3.4450 -2.7547 1.9060 H
18 C18 -1.5395 0.8959 -1.6647 C
19 H19 -0.8746 1.7696 -1.5774 H
20 C20 -2.8380 1.2763 -0.9995 C
21 H21 -2.9517 2.3604 -1.1810 H
22 C22 -3.0848 0.9787 0.4837 C
23 H23 -3.5116 1.8457 1.0094 H
24 C24 -4.1909 -0.0373 0.0951 C
25 H25 -5.1190 0.3112 0.5896 H
26 C26 -4.2407 -1.5216 0.3853 C
27 H27 -5.1621 -1.7395 0.9431 H
28 C28 -4.1841 0.5796 -1.3329 C
29 H29 -4.9580 1.3724 -1.3541 H
30 C30 -4.4300 -0.1751 -2.6104 C
31 H31 -5.3990 0.1095 -3.0421 H
32 C32 -4.4254 -1.6499 -2.3263 C
33 H33 -5.4332 -1.9464 -2.6778 H
34 C34 -4.2427 -2.2799 -0.9169 C
35 H35 -5.0517 -3.0241 -0.7779 H
36 C36 -3.0095 -3.0530 -1.4529 C
37 H37 -3.1671 -4.1322 -1.2760 H
38 C38 -3.4435 -2.6971 -2.8834 C
39 H39 -3.9950 -3.5253 -3.3544 H
40 C40 -2.3502 -2.2080 -3.8046 C
41 H41 -2.1210 -2.9676 -4.5665 H
42 C42 -1.0535 -1.6819 -3.1716 C
43 H43 -0.2010 -2.2328 -3.6040 H
44 C44 -1.2516 -0.3625 -3.9322 C
45 H45 -0.5085 -0.0821 -4.6801 H
46 C46 -1.9282 0.7417 -3.1195 C
47 H47 -1.7642 1.7186 -3.6059 H
48 C48 -2.6154 -0.8523 -4.4341 C
49 H49 -2.7947 -0.8138 -5.5101 H
50 C50 -3.3169 0.1915 -3.5587 C
51 H51 -3.7680 0.9517 -4.2231 H
52 H52 -2.4621 -0.9247 2.8641 H
@<TRIPOS>BOND
1 1 2 1
2 1 3 1
3 1 11 1
4 1 14 1

5 3 4 1
6 3 5 1
7 3 18 1
8 5 6 1
9 5 7 1
10 5 42 1
11 7 8 1
12 7 9 1
13 7 36 1
14 9 10 1
15 9 11 1
16 9 16 1
17 11 12 1
18 11 13 1
19 13 14 1
20 13 16 1
21 13 52 1
22 14 15 1
23 14 22 1
24 16 17 1
25 16 26 1
26 18 19 1
27 18 20 1
28 18 46 1
29 20 21 1
30 20 22 1
31 20 28 1
32 22 23 1
33 22 24 1
34 24 25 1
35 24 26 1
36 24 28 1
37 26 27 1
38 26 34 1
39 28 29 1
40 28 30 1
41 30 31 1
42 30 32 1
43 30 50 1
44 32 33 1
45 32 34 1
46 32 38 1
47 34 35 1
48 34 36 1
49 36 37 1
50 36 38 1
51 38 39 1
52 38 40 1
53 40 41 1
54 40 42 1

55 40 48 1
56 42 43 1
57 42 44 1
58 44 45 1
59 44 46 1
60 44 48 1
61 46 47 1
62 46 50 1
63 48 49 1
64 48 50 1
65 50 51 1

(c) Cartesian Coordinates to the left chiral compound (Å)

0 1

6	0.031	3.182	0.061
6	-0.676	2.490	-1.138
6	1.386	3.074	-0.657
6	0.672	2.525	-1.925
1	0.649	3.054	-2.881
1	-0.288	4.228	0.116
1	2.024	3.961	-0.659
1	-1.392	3.182	-1.591
6	1.502	1.235	-1.845
1	2.286	1.239	-2.610
6	2.129	1.711	-0.496
1	3.202	1.864	-0.643
6	-0.059	2.536	1.417
1	-0.141	3.308	2.190
6	1.954	0.862	0.793
1	2.936	0.887	1.273
6	-1.124	1.448	1.710
1	-1.951	1.816	2.326
6	-0.043	0.748	2.568
1	-0.143	1.121	3.593
6	1.767	-0.692	0.517
1	2.799	-1.050	0.561
6	1.124	-1.448	1.710
1	1.951	-1.816	2.326
6	0.043	-0.748	2.568
1	0.143	-1.121	3.593
6	-1.386	1.122	-0.953
1	-2.374	1.256	-1.409
6	-1.954	-0.862	0.793
1	-2.936	-0.887	1.273
6	-1.063	-1.562	1.860
1	-1.753	-2.040	2.562
6	-1.767	0.692	0.517
1	-2.799	1.050	0.561
6	1.063	1.562	1.860
1	1.753	2.040	2.562

6	0.059	-2.536	1.417
1	0.141	-3.308	2.190
6	0.759	-0.092	-1.900
1	0.950	-0.526	-2.892
6	1.386	-1.122	-0.953
1	2.374	-1.256	-1.409
6	0.676	-2.490	-1.138
1	1.392	-3.182	-1.591
6	-0.031	-3.182	0.061
1	0.288	-4.228	0.116
6	-1.386	-3.074	-0.657
1	-2.024	-3.961	-0.659
6	-2.129	-1.711	-0.496
1	-3.202	-1.864	-0.643
6	-0.672	-2.525	-1.925
1	-0.649	-3.054	-2.881
6	-1.502	-1.235	-1.845
1	-2.286	-1.239	-2.610
6	-0.759	0.092	-1.900
1	-0.950	0.526	-2.892

.mol2 file

@<TRIPOS>MOLECULE
Molecule Name
56 70
SMALL
NO_CHARGES

@<TRIPOS>ATOM
1 C1 0.0307 3.1822 0.0610 C
2 C2 -0.6761 2.4904 -1.1377 C
3 C3 1.3861 3.0741 -0.6573 C
4 C4 0.6718 2.5249 -1.9253 C
5 H5 0.6491 3.0536 -2.8813 H
6 H6 -0.2883 4.2279 0.1157 H
7 H7 2.0242 3.9613 -0.6590 H
8 H8 -1.3916 3.1817 -1.5915 H
9 C9 1.5024 1.2352 -1.8453 C
10 H10 2.2860 1.2387 -2.6101 H
11 C11 2.1291 1.7112 -0.4958 C
12 H12 3.2018 1.8644 -0.6435 H
13 C13 -0.0585 2.5363 1.4170 C
14 H14 -0.1411 3.3082 2.1898 H
15 C15 1.9535 0.8621 0.7935 C
16 H16 2.9356 0.8869 1.2726 H
17 C17 -1.1239 1.4479 1.7097 C
18 H18 -1.9508 1.8161 2.3260 H
19 C19 -0.0427 0.7480 2.5678 C
20 H20 -0.1428 1.1210 3.5926 H

21 C21 1.7672 -0.6918 0.5167 C
22 H22 2.7987 -1.0496 0.5613 H
23 C23 1.1239 -1.4479 1.7097 C
24 H24 1.9508 -1.8161 2.3260 H
25 C25 0.0427 -0.7480 2.5678 C
26 H26 0.1428 -1.1210 3.5926 H
27 C27 -1.3861 1.1218 -0.9531 C
28 H28 -2.3743 1.2564 -1.4088 H
29 C29 -1.9535 -0.8621 0.7935 C
30 H30 -2.9356 -0.8869 1.2726 H
31 C31 -1.0626 -1.5622 1.8599 C
32 H32 -1.7531 -2.0404 2.5618 H
33 C33 -1.7672 0.6918 0.5167 C
34 H34 -2.7987 1.0496 0.5613 H
35 C35 1.0626 1.5622 1.8599 C
36 H36 1.7531 2.0404 2.5618 H
37 C37 0.0585 -2.5363 1.4170 C
38 H38 0.1411 -3.3082 2.1898 H
39 C39 0.7586 -0.0924 -1.9001 C
40 H40 0.9500 -0.5265 -2.8920 H
41 C41 1.3861 -1.1218 -0.9531 C
42 H42 2.3743 -1.2564 -1.4088 H
43 C43 0.6761 -2.4904 -1.1377 C
44 H44 1.3916 -3.1817 -1.5915 H
45 C45 -0.0307 -3.1822 0.0610 C
46 H46 0.2883 -4.2279 0.1157 H
47 C47 -1.3861 -3.0741 -0.6573 C
48 H48 -2.0242 -3.9613 -0.6590 H
49 C49 -2.1291 -1.7112 -0.4958 C
50 H50 -3.2018 -1.8644 -0.6435 H
51 C51 -0.6718 -2.5249 -1.9253 C
52 H52 -0.6491 -3.0536 -2.8813 H
53 C53 -1.5024 -1.2352 -1.8453 C
54 H54 -2.2860 -1.2387 -2.6101 H
55 C55 -0.7586 0.0924 -1.9001 C
56 H56 -0.9500 0.5265 -2.8920 H
@<TRIPOS>BOND
1 1 2 1
2 1 3 1
3 1 6 1
4 1 13 1
5 2 4 1
6 2 8 1
7 2 27 1
8 3 4 1
9 3 7 1
10 3 11 1
11 4 5 1
12 4 9 1
13 9 10 1

14 9 11 1
15 9 39 1
16 11 12 1
17 11 15 1
18 13 14 1
19 13 17 1
20 13 35 1
21 15 16 1
22 15 21 1
23 15 35 1
24 17 18 1
25 17 19 1
26 17 33 1
27 19 20 1
28 19 25 1
29 19 35 1
30 21 22 1
31 21 23 1
32 21 41 1
33 23 24 1
34 23 25 1
35 23 37 1
36 25 26 1
37 25 31 1
38 27 28 1
39 27 33 1
40 27 55 1
41 29 30 1
42 29 31 1
43 29 33 1
44 29 49 1
45 31 32 1
46 31 37 1
47 33 34 1
48 35 36 1
49 37 38 1
50 37 45 1
51 39 40 1
52 39 41 1
53 39 55 1
54 41 42 1
55 41 43 1
56 43 44 1
57 43 45 1
58 43 51 1
59 45 46 1
60 45 47 1
61 47 48 1
62 47 49 1
63 47 51 1

64 49 50 1
65 49 53 1
66 51 52 1
67 51 53 1
68 53 54 1
69 53 55 1
70 55 56 1

(c) Cartesian Coordinates to the right chiral compound (Å)

0 1

6	0.058	-0.913	-0.271
1	-0.456	-1.216	-1.185
6	0.548	-2.000	0.668
1	0.077	-2.969	0.448
6	-0.135	-1.328	1.859
1	-0.893	-2.015	2.269
6	0.863	0.401	-0.307
1	0.682	0.898	-1.277
6	-0.116	0.999	0.733
1	-0.780	1.725	0.237
6	0.614	-0.739	3.051
1	0.095	-1.163	3.931
6	0.433	1.634	1.997
1	-0.252	2.468	2.231
6	0.313	0.740	3.218
1	-0.767	0.772	3.447
6	-0.807	-0.358	0.861
1	-1.890	-0.411	0.743
6	1.006	1.304	4.445
6	1.608	2.705	4.340
6	2.290	3.009	2.988
6	1.767	2.309	1.737
6	2.932	2.185	4.899
6	3.630	2.688	3.648
6	2.386	0.744	4.868
6	3.016	-0.393	4.078
6	2.723	-1.926	2.074
6	2.038	-2.124	0.711
6	2.855	-0.929	0.163
6	3.865	-1.273	1.275
6	2.363	0.491	-0.075
6	2.817	1.591	0.886
6	4.160	1.286	1.536
1	4.920	1.712	0.858
6	4.590	-0.140	1.923

1	5.678	-0.265	1.791
6	4.245	0.182	3.389
1	5.085	-0.133	4.036
6	4.491	1.657	2.991
1	5.556	1.918	3.110
1	2.305	0.374	5.905
1	3.254	2.581	5.864
1	3.424	-1.098	4.822
1	4.585	-2.035	0.933
1	3.076	-2.899	2.459
1	3.275	-1.233	-0.813
1	2.356	-3.063	0.230
1	2.809	0.776	-1.042
1	3.084	2.408	0.189
1	1.459	3.139	1.073
1	2.242	4.093	2.789
1	1.124	3.527	4.869
1	0.309	1.245	5.297
1	4.197	3.610	3.844
6	2.047	-1.240	3.252
1	1.853	-2.093	3.930

.mol2 file

```
@<TRIPOS>MOLECULE
Molecule Name
56 70
SMALL
NO_CHARGES


@<TRIPOS>ATOM
1 C1     0.0579    -0.9135    -0.2708 C
2 H2    -0.4555    -1.2159    -1.1854 H
3 C3     0.5482    -2.0003     0.6685 C
4 H4     0.0775    -2.9693     0.4478 H
5 C5    -0.1347    -1.3276     1.8588 C
6 H6    -0.8935    -2.0153     2.2691 H
7 C7     0.8627     0.4007    -0.3074 C
8 H8     0.6824     0.8982    -1.2765 H
9 C9    -0.1159     0.9987     0.7325 C
10 H10    -0.7799     1.7252     0.2366 H
11 C11     0.6142    -0.7390     3.0508 C
12 H12     0.0948    -1.1629     3.9307 H
13 C13     0.4325     1.6336     1.9972 C
14 H14    -0.2524     2.4681     2.2308 H
15 C15     0.3132     0.7397     3.2184 C
```

16 H16 -0.7670 0.7719 3.4468 H
17 C17 -0.8074 -0.3585 0.8613 C
18 H18 -1.8905 -0.4113 0.7426 H
19 C19 1.0058 1.3038 4.4453 C
20 C20 1.6076 2.7050 4.3400 C
21 C21 2.2901 3.0087 2.9878 C
22 C22 1.7670 2.3092 1.7367 C
23 C23 2.9324 2.1846 4.8988 C
24 C24 3.6304 2.6879 3.6484 C
25 C25 2.3857 0.7436 4.8679 C
26 C26 3.0164 -0.3927 4.0776 C
27 C27 2.7234 -1.9261 2.0735 C
28 C28 2.0379 -2.1238 0.7106 C
29 C29 2.8548 -0.9293 0.1629 C
30 C30 3.8647 -1.2725 1.2748 C
31 C31 2.3632 0.4906 -0.0750 C
32 C32 2.8172 1.5909 0.8857 C
33 C33 4.1597 1.2857 1.5356 C
34 H34 4.9204 1.7117 0.8582 H
35 C35 4.5904 -0.1398 1.9227 C
36 H36 5.6778 -0.2653 1.7911 H
37 C37 4.2452 0.1824 3.3894 C
38 H38 5.0848 -0.1325 4.0359 H
39 C39 4.4914 1.6571 2.9911 C
40 H40 5.5556 1.9177 3.1098 H
41 H41 2.3051 0.3743 5.9053 H
42 H42 3.2541 2.5811 5.8636 H
43 H43 3.4238 -1.0976 4.8216 H
44 H44 4.5845 -2.0345 0.9333 H
45 H45 3.0756 -2.8987 2.4595 H
46 H46 3.2750 -1.2330 -0.8134 H
47 H47 2.3560 -3.0632 0.2300 H
48 H48 2.8090 0.7765 -1.0424 H
49 H49 3.0838 2.4079 0.1887 H
50 H50 1.4594 3.1392 1.0732 H
51 H51 2.2418 4.0926 2.7887 H
52 H52 1.1237 3.5273 4.8689 H
53 H53 0.3092 1.2453 5.2973 H
54 H54 4.1971 3.6097 3.8440 H
55 C55 2.0466 -1.2398 3.2519 C
56 H56 1.8531 -2.0927 3.9298 H
@<TRIPOS>BOND
1 1 2 1
2 1 3 1
3 1 7 1
4 1 17 1
5 3 4 1

6 3 5 1
7 3 28 1
8 5 6 1
9 5 11 1
10 5 17 1
11 7 8 1
12 7 9 1
13 7 31 1
14 9 10 1
15 9 13 1
16 9 17 1
17 11 12 1
18 11 15 1
19 11 55 1
20 13 14 1
21 13 15 1
22 13 22 1
23 15 16 1
24 15 19 1
25 17 18 1
26 19 20 1
27 19 25 1
28 19 53 1
29 20 21 1
30 20 23 1
31 20 52 1
32 21 22 1
33 21 24 1
34 21 51 1
35 22 32 1
36 22 50 1
37 23 24 1
38 23 25 1
39 23 42 1
40 24 39 1
41 24 54 1
42 25 26 1
43 25 41 1
44 26 37 1
45 26 43 1
46 26 55 1
47 27 28 1
48 27 30 1
49 27 45 1
50 27 55 1
51 28 29 1
52 28 47 1

53 29 30 1
54 29 31 1
55 29 46 1
56 30 35 1
57 30 44 1
58 31 32 1
59 31 48 1
60 32 33 1
61 32 49 1
62 33 34 1
63 33 35 1
64 33 39 1
65 35 36 1
66 35 37 1
67 37 38 1
68 37 39 1
69 39 40 1
70 55 56 1

Figure S19. DFT ωB97XD/6-31G* optimized geometries of the following pairs of chiral nuggets: (a) $nugget_{24b}$ ($C_{24}H_{24}$); (b) $nugget_{26b}$ ($C_{26}H_{26}$); and (c) $nugget_{28b}$ ($C_{28}H_{28}$). Cartesian coordinates of its atoms; the first line contains the total charge and multiplicity; the following lines contain the atomic numbers, followed by the x, y, and z coordinates in Å for each one of the atoms. Next, atomic coordinates in Tripos Mol2 file format (.mol2) with the distances also in Å.

1D-scaffold generator $C_{42}H_{36}$

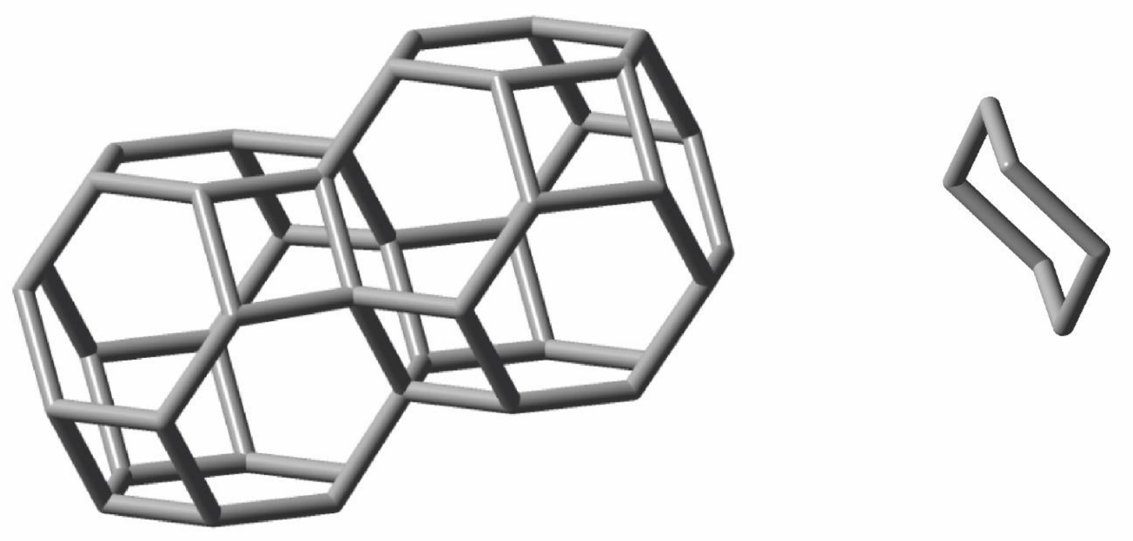

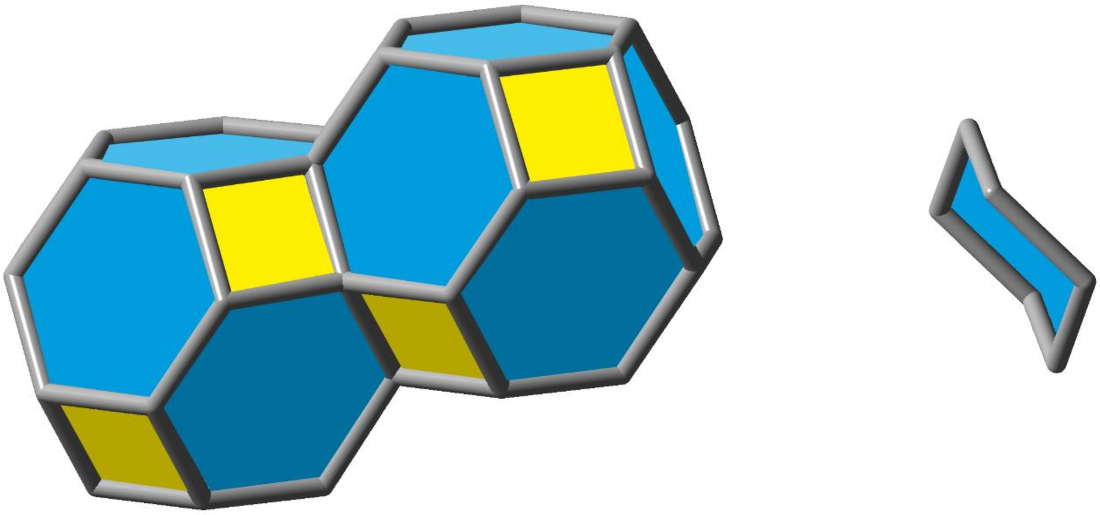

Cartesian Coordinates (Å)

0 1

6	-1.250	-0.693	-2.271
6	0.000	-0.718	-1.361
6	-1.250	0.865	-2.211
6	0.000	0.820	-1.302
1	-0.969	-1.017	-3.279
1	-0.968	1.265	-3.191
6	-0.000	-1.538	-0.059
6	-2.494	-2.310	-0.530
1	-2.761	-3.345	-0.759
6	-2.493	-1.477	-1.853
1	-2.761	-2.129	-2.688

6	-0.000	-0.820	1.302
6	-0.000	0.718	1.361
6	0.000	1.538	0.059
6	-2.493	1.615	-1.735
1	-2.761	2.330	-2.518
6	-3.764	-0.712	-1.370
1	-4.618	-1.011	-1.984
6	-3.764	0.815	-1.311
1	-4.619	1.160	-1.899
6	-1.251	-1.621	1.735
1	-0.969	-2.331	2.521
6	-3.765	-1.543	-0.050
1	-4.619	-2.225	-0.055
6	-2.493	2.344	-0.352
1	-2.761	3.394	-0.500
6	-1.250	2.314	0.536
1	-0.968	3.349	0.759
6	-1.250	1.483	1.854
1	-0.968	2.130	2.692
6	-3.764	1.543	0.068
1	-4.619	2.223	0.116
6	-3.765	0.728	1.361
1	-4.619	1.066	1.954
6	-2.493	0.695	2.265
1	-2.760	1.015	3.276
6	-2.493	-0.867	2.205
1	-2.761	-1.263	3.188
6	-3.765	-0.830	1.301
1	-4.619	-1.212	1.867
6	-1.251	-2.348	0.357
1	-0.969	-3.397	0.500
6	2.493	-0.695	-2.265
6	3.765	-0.728	-1.361
6	2.493	0.867	-2.205
6	3.765	0.830	-1.301
1	4.619	1.212	-1.867
1	2.760	-1.015	-3.276
1	2.761	1.263	-3.188
1	4.619	-1.066	-1.954
6	3.764	-1.543	-0.068
1	4.619	-2.223	-0.116
6	1.250	-2.314	-0.536
1	0.968	-3.349	-0.759
6	1.250	-1.483	-1.854
1	0.968	-2.130	-2.692
6	3.764	-0.815	1.311
1	4.619	-1.160	1.899
6	3.764	0.712	1.370
1	4.618	1.011	1.984
6	3.765	1.543	0.050

1	4.619	2.225	0.055
6	1.251	1.621	-1.735
1	0.969	2.331	-2.521
6	2.493	-1.615	1.735
1	2.761	-2.330	2.518
6	1.251	2.348	-0.357
1	0.969	3.397	-0.500
6	2.494	2.310	0.530
1	2.761	3.345	0.759
6	2.493	1.477	1.853
1	2.761	2.129	2.688
6	1.250	0.693	2.271
1	0.969	1.017	3.279
6	1.250	-0.865	2.211
1	0.968	-1.265	3.191
6	2.493	-2.344	0.352
1	2.761	-3.394	0.500

.mol2 file

```
@<TRIPOS>MOLECULE
Molecule Name
78 102
SMALL
NO_CHARGES


@<TRIPOS>ATOM
1 C1    -1.2504    -0.6925    -2.2712 C
2 C2     0.0001    -0.7176    -1.3610 C
3 C3    -1.2504     0.8649    -2.2112 C
4 C4     0.0003     0.8200    -1.3018 C
5 H5    -0.9689    -1.0171    -3.2786 H
6 H6    -0.9683     1.2652    -3.1909 H
7 C7    -0.0003    -1.5379    -0.0592 C
8 C8    -2.4935    -2.3100    -0.5305 C
9 H9    -2.7613    -3.3451    -0.7593 H
10 C10    -2.4931    -1.4766    -1.8533 C
11 H11    -2.7607    -2.1294    -2.6885 H
12 C12    -0.0003    -0.8200     1.3018 C
13 C13    -0.0001     0.7176     1.3610 C
14 C14     0.0003     1.5379     0.0592 C
15 C15    -2.4930     1.6149    -1.7348 C
16 H16    -2.7608     2.3296    -2.5177 H
17 C17    -3.7643    -0.7118    -1.3699 C
18 H18    -4.6183    -1.0110    -1.9836 H
19 C19    -3.7642     0.8151    -1.3110 C
20 H20    -4.6187     1.1604    -1.8994 H
21 C21    -1.2507    -1.6212     1.7354 C
22 H22    -0.9688    -2.3309     2.5206 H
23 C23    -3.7647    -1.5431    -0.0504 C
```

24	H24	-4.6191	-2.2253	-0.0554	H
25	C25	-2.4931	2.3441	-0.3519	C
26	H26	-2.7608	3.3938	-0.5000	H
27	C27	-1.2502	2.3139	0.5359	C
28	H28	-0.9682	3.3485	0.7589	H
29	C29	-1.2505	1.4832	1.8544	C
30	H30	-0.9683	2.1304	2.6916	H
31	C31	-3.7642	1.5426	0.0684	C
32	H32	-4.6185	2.2233	0.1158	H
33	C33	-3.7646	0.7283	1.3612	C
34	H34	-4.6188	1.0656	1.9545	H
35	C35	-2.4933	0.6953	2.2653	C
36	H36	-2.7603	1.0154	3.2760	H
37	C37	-2.4934	-0.8671	2.2050	C
38	H38	-2.7612	-1.2633	3.1882	H
39	C39	-3.7647	-0.8302	1.3011	C
40	H40	-4.6190	-1.2117	1.8670	H
41	C41	-1.2508	-2.3481	0.3570	C
42	H42	-0.9689	-3.3969	0.4996	H
43	C43	2.4933	-0.6953	-2.2653	C
44	C44	3.7646	-0.7283	-1.3612	C
45	C45	2.4934	0.8671	-2.2050	C
46	C46	3.7647	0.8302	-1.3011	C
47	H47	4.6190	1.2117	-1.8670	H
48	H48	2.7603	-1.0154	-3.2760	H
49	H49	2.7612	1.2633	-3.1882	H
50	H50	4.6188	-1.0656	-1.9545	H
51	C51	3.7642	-1.5426	-0.0684	C
52	H52	4.6185	-2.2233	-0.1158	H
53	C53	1.2502	-2.3139	-0.5359	C
54	H54	0.9682	-3.3485	-0.7589	H
55	C55	1.2505	-1.4832	-1.8544	C
56	H56	0.9683	-2.1304	-2.6916	H
57	C57	3.7642	-0.8151	1.3110	C
58	H58	4.6187	-1.1604	1.8994	H
59	C59	3.7643	0.7118	1.3699	C
60	H60	4.6183	1.0110	1.9836	H
61	C61	3.7647	1.5431	0.0504	C
62	H62	4.6191	2.2253	0.0554	H
63	C63	1.2507	1.6212	-1.7354	C
64	H64	0.9688	2.3309	-2.5206	H
65	C65	2.4930	-1.6149	1.7348	C
66	H66	2.7608	-2.3296	2.5177	H
67	C67	1.2508	2.3481	-0.3570	C
68	H68	0.9689	3.3969	-0.4996	H
69	C69	2.4935	2.3100	0.5305	C
70	H70	2.7613	3.3451	0.7593	H
71	C71	2.4931	1.4767	1.8533	C
72	H72	2.7607	2.1294	2.6885	H
73	C73	1.2504	0.6925	2.2712	C

74 H74 0.9689 1.0171 3.2786 H
75 C75 1.2504 -0.8649 2.2112 C
76 H76 0.9683 -1.2652 3.1909 H
77 C77 2.4931 -2.3441 0.3519 C
78 H78 2.7608 -3.3938 0.5000 H
@<TRIPOS>BOND
1 1 2 1
2 1 3 1
3 1 5 1
4 1 10 1
5 2 4 1
6 2 7 1
7 2 55 1
8 3 4 1
9 3 6 1
10 3 15 1
11 4 14 1
12 4 63 1
13 7 12 1
14 7 41 1
15 7 53 1
16 8 9 1
17 8 10 1
18 8 23 1
19 8 41 1
20 10 11 1
21 10 17 1
22 12 13 1
23 12 21 1
24 12 75 1
25 13 14 1
26 13 29 1
27 13 73 1
28 14 27 1
29 14 67 1
30 15 16 1
31 15 19 1
32 15 25 1
33 17 18 1
34 17 19 1
35 17 23 1
36 19 20 1
37 19 31 1
38 21 22 1
39 21 37 1
40 21 41 1
41 23 24 1
42 23 39 1
43 25 26 1
44 25 27 1

45 25 31 1
46 27 28 1
47 27 29 1
48 29 30 1
49 29 35 1
50 31 32 1
51 31 33 1
52 33 34 1
53 33 35 1
54 33 39 1
55 35 36 1
56 35 37 1
57 37 38 1
58 37 39 1
59 39 40 1
60 41 42 1
61 43 44 1
62 43 45 1
63 43 48 1
64 43 55 1
65 44 46 1
66 44 50 1
67 44 51 1
68 45 46 1
69 45 49 1
70 45 63 1
71 46 47 1
72 46 61 1
73 51 52 1
74 51 57 1
75 51 77 1
76 53 54 1
77 53 55 1
78 53 77 1
79 55 56 1
80 57 58 1
81 57 59 1
82 57 65 1
83 59 60 1
84 59 61 1
85 59 71 1
86 61 62 1
87 61 69 1
88 63 64 1
89 63 67 1
90 65 66 1
91 65 75 1
92 65 77 1
93 67 68 1
94 67 69 1

95 69 70 1
96 69 71 1
97 71 72 1
98 71 73 1
99 73 74 1
100 73 75 1
101 75 76 1
102 77 78 1

Figure S20. Left: Optimized geometry of the 1D-scaffold generator $C_{42}H_{36}$ obtained from the linear hexagonal face-fusion of nugget$_{24a}$. Right: the released cyclohexane molecule. Cartesian coordinates of its atoms; the first line contains the total charge and multiplicity; the following lines contain the atomic numbers, followed by the x, y, and z coordinates in Å for each one of the atoms. Next, atomic coordinates in Tripos Mol2 file format (.mol2) with the distances also in Å.

$C_{58}H_{46}$ 2D-scaffold

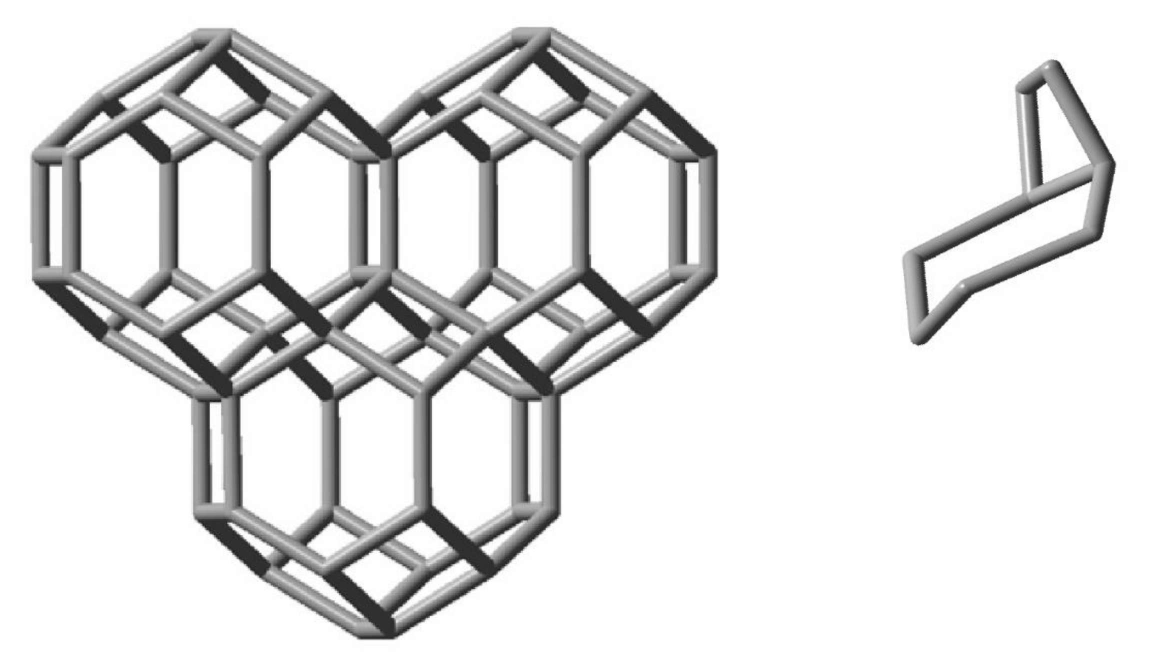

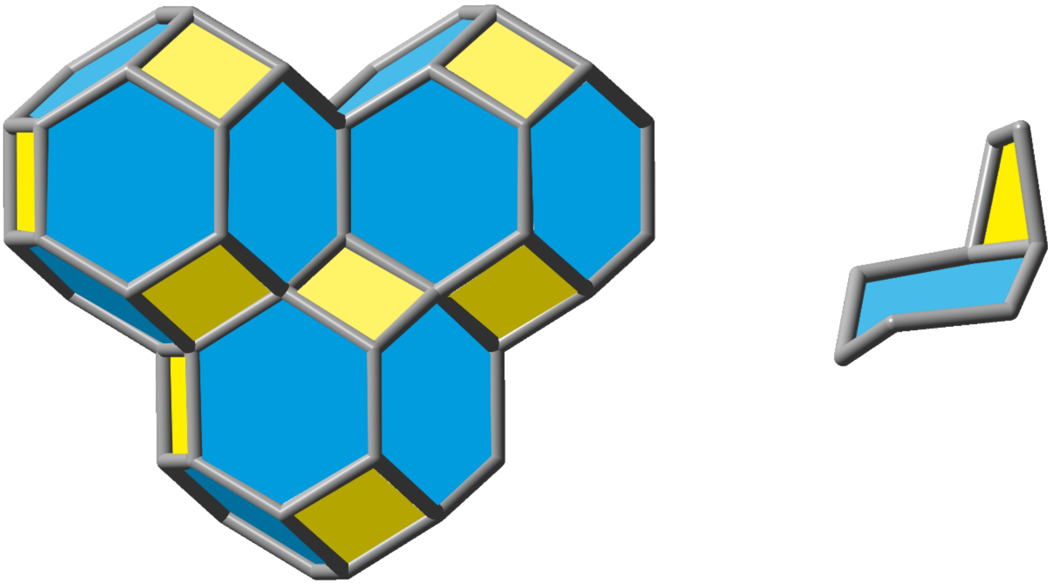

Cartesian Coordinates (Å)

0 1

6	2.219	-0.199	2.328
6	1.094	0.500	1.527
6	3.291	0.617	1.554
6	2.164	1.309	0.764
1	2.183	0.134	3.370
1	3.752	1.344	2.232
6	0.000	-0.317	0.772
6	1.239	-2.541	1.511

1	0.843	-3.273	2.212
6	2.290	-1.714	2.311
1	2.282	-2.050	3.352
6	0.000	-0.317	-0.772
6	1.094	0.500	-1.527
6	2.164	1.309	-0.765
6	4.410	-0.080	0.783
1	5.357	0.333	1.141
6	3.443	-2.441	1.548
1	3.979	-3.097	2.239
6	4.488	-1.635	0.781
1	5.471	-1.951	1.141
6	-0.000	-1.898	-0.807
6	2.387	-3.263	0.757
1	2.423	-4.307	1.081
6	4.410	-0.080	-0.783
1	5.357	0.333	-1.142
6	3.291	0.617	-1.554
1	3.752	1.343	-2.232
6	2.219	-0.200	-2.328
1	2.183	0.134	-3.370
6	4.488	-1.635	-0.781
1	5.471	-1.951	-1.141
6	3.443	-2.441	-1.548
1	3.979	-3.097	-2.239
6	2.290	-1.715	-2.311
1	2.281	-2.050	-3.352
6	1.238	-2.542	-1.511
1	0.843	-3.273	-2.212
6	2.387	-3.263	-0.756
1	2.422	-4.307	-1.080
6	-0.000	-1.899	0.808
6	0.000	2.795	2.325
6	-1.097	3.598	1.552
6	1.098	3.598	1.552
6	0.000	4.377	0.767
1	0.000	5.424	1.085
1	0.000	3.104	3.373
1	1.599	4.278	2.246
1	-1.599	4.278	2.246
6	-2.172	2.854	0.778
1	-3.149	3.197	1.137
6	-1.094	0.500	1.527
6	0.000	1.257	2.311
1	0.000	0.906	3.352
6	-2.173	2.854	-0.777
1	-3.149	3.197	-1.137
6	-1.097	3.598	-1.552
1	-1.599	4.278	-2.246
6	0.000	4.378	-0.767

1	0.000	5.424	-1.084
6	2.173	2.854	0.778
1	3.149	3.197	1.137
6	-2.164	1.310	-0.764
6	2.173	2.854	-0.777
1	3.149	3.197	-1.137
6	1.098	3.598	-1.552
1	1.600	4.278	-2.246
6	0.000	2.795	-2.325
1	-0.000	3.104	-3.373
6	-0.000	1.257	-2.311
1	0.000	0.906	-3.351
6	-1.094	0.500	-1.527
6	-2.164	1.309	0.764
6	-2.219	-0.200	2.328
6	-3.291	0.617	1.554
1	-2.183	0.134	3.370
1	-3.751	1.344	2.232
6	-4.410	-0.079	0.783
1	-5.357	0.333	1.142
6	-3.443	-2.441	1.548
1	-3.980	-3.097	2.239
6	-2.290	-1.714	2.311
1	-2.282	-2.050	3.352
6	-4.410	-0.079	-0.783
1	-5.357	0.333	-1.142
6	-3.291	0.618	-1.554
1	-3.751	1.344	-2.232
6	-1.239	-2.542	1.511
1	-0.843	-3.273	2.212
6	-4.488	-1.635	-0.781
1	-5.471	-1.951	-1.141
6	-2.388	-3.263	0.756
1	-2.423	-4.307	1.081
6	-2.219	-0.199	-2.328
1	-2.183	0.134	-3.370
6	-1.239	-2.542	-1.511
1	-0.843	-3.273	-2.211
6	-2.289	-1.714	-2.311
1	-2.281	-2.050	-3.352
6	-3.443	-2.441	-1.548
1	-3.979	-3.097	-2.239
6	-2.388	-3.263	-0.756
1	-2.423	-4.307	-1.081
6	-4.488	-1.635	0.780
1	-5.471	-1.951	1.141

.mol2 file

```
@<TRIPOS>MOLECULE
Molecule Name
104 139
SMALL
NO_CHARGES


@<TRIPOS>ATOM
1 C1     2.2194    -0.1994     2.3275 C
2 C2     1.0942     0.5001     1.5267 C
3 C3     3.2910     0.6174     1.5537 C
4 C4     2.1640     1.3093     0.7645 C
5 H5     2.1828     0.1342     3.3703 H
6 H6     3.7515     1.3436     2.2316 H
7 C7     0.0000    -0.3174     0.7719 C
8 C8     1.2387    -2.5414     1.5112 C
9 H9     0.8431    -3.2730     2.2119 H
10 C10     2.2899    -1.7145     2.3113 C
11 H11     2.2817    -2.0503     3.3521 H
12 C12     0.0001    -0.3174    -0.7719 C
13 C13     1.0941     0.5001    -1.5267 C
14 C14     2.1640     1.3094    -0.7647 C
15 C15     4.4101    -0.0795     0.7826 C
16 H16     5.3572     0.3327     1.1414 H
17 C17     3.4428    -2.4408     1.5481 C
18 H18     3.9792    -3.0972     2.2387 H
19 C19     4.4882    -1.6349     0.7806 C
20 H20     5.4708    -1.9512     1.1411 H
21 C21    -0.0001    -1.8985    -0.8074 C
22 C22     2.3872    -3.2633     0.7566 C
23 H23     2.4226    -4.3073     1.0805 H
24 C24     4.4101    -0.0795    -0.7828 C
25 H25     5.3571     0.3327    -1.1417 H
26 C26     3.2909     0.6174    -1.5538 C
27 H27     3.7515     1.3435    -2.2319 H
28 C28     2.2192    -0.1996    -2.3276 C
29 H29     2.1825     0.1341    -3.3704 H
30 C30     4.4881    -1.6349    -0.7807 C
31 H31     5.4708    -1.9513    -1.1413 H
32 C32     3.4427    -2.4409    -1.5481 C
33 H33     3.9790    -3.0974    -2.2387 H
34 C34     2.2897    -1.7146    -2.3113 C
35 H35     2.2815    -2.0504    -3.3521 H
36 C36     1.2385    -2.5415    -1.5110 C
37 H37     0.8427    -3.2731    -2.2116 H
38 C38     2.3871    -3.2633    -0.7564 C
39 H39     2.4224    -4.3073    -1.0804 H
40 C40    -0.0002    -1.8986     0.8075 C
41 C41     0.0001     2.7949     2.3249 C
42 C42    -1.0975     3.5980     1.5523 C
43 C43     1.0977     3.5979     1.5524 C
44 C44     0.0002     4.3774     0.7675 C
45 H45     0.0002     5.4239     1.0846 H
```

46	H46	-0.0000	3.1042	3.3735	H
47	H47	1.5995	4.2781	2.2459	H
48	H48	-1.5991	4.2784	2.2457	H
49	C49	-2.1725	2.8542	0.7777	C
50	H50	-3.1486	3.1970	1.1371	H
51	C51	-1.0940	0.5000	1.5270	C
52	C52	0.0000	1.2574	2.3113	C
53	H53	0.0003	0.9064	3.3515	H
54	C54	-2.1727	2.8542	-0.7774	C
55	H55	-3.1486	3.1974	-1.1368	H
56	C56	-1.0975	3.5980	-1.5521	C
57	H57	-1.5992	4.2783	-2.2457	H
58	C58	0.0002	4.3775	-0.7675	C
59	H59	0.0001	5.4240	-1.0844	H
60	C60	2.1726	2.8541	0.7776	C
61	H61	3.1488	3.1970	1.1366	H
62	C62	-2.1638	1.3095	-0.7644	C
63	C63	2.1726	2.8542	-0.7775	C
64	H64	3.1487	3.1971	-1.1365	H
65	C65	1.0977	3.5980	-1.5524	C
66	H66	1.5996	4.2782	-2.2460	H
67	C67	0.0000	2.7950	-2.3248	C
68	H68	-0.0002	3.1042	-3.3735	H
69	C69	-0.0001	1.2574	-2.3111	C
70	H70	0.0001	0.9065	-3.3514	H
71	C71	-1.0941	0.5001	-1.5267	C
72	C72	-2.1636	1.3092	0.7644	C
73	C73	-2.2193	-0.1995	2.3277	C
74	C74	-3.2908	0.6174	1.5535	C
75	H75	-2.1831	0.1342	3.3704	H
76	H76	-3.7510	1.3437	2.2316	H
77	C77	-4.4103	-0.0792	0.7827	C
78	H78	-5.3572	0.3330	1.1419	H
79	C79	-3.4431	-2.4405	1.5482	C
80	H80	-3.9796	-3.0967	2.2390	H
81	C81	-2.2898	-1.7145	2.3113	C
82	H82	-2.2817	-2.0504	3.3521	H
83	C83	-4.4103	-0.0790	-0.7828	C
84	H84	-5.3572	0.3332	-1.1418	H
85	C85	-3.2908	0.6175	-1.5537	C
86	H86	-3.7509	1.3437	-2.2319	H
87	C87	-1.2388	-2.5416	1.5110	C
88	H88	-0.8433	-3.2735	2.2115	H
89	C89	-4.4881	-1.6345	-0.7809	C
90	H90	-5.4709	-1.9508	-1.1412	H
91	C91	-2.3877	-3.2632	0.7565	C
92	H92	-2.4232	-4.3072	1.0805	H
93	C93	-2.2192	-0.1994	-2.3276	C
94	H94	-2.1829	0.1343	-3.3703	H
95	C95	-1.2387	-2.5415	-1.5108	C
96	H96	-0.8431	-3.2734	-2.2112	H
97	C97	-2.2895	-1.7144	-2.3114	C
98	H98	-2.2813	-2.0503	-3.3522	H
99	C99	-3.4429	-2.4405	-1.5484	C
100	H100	-3.9793	-3.0968	-2.2391	H

```
101 C101    -2.3876    -3.2630    -0.7565 C
102 H102    -2.4231    -4.3071    -1.0805 H
103 C103    -4.4882    -1.6346     0.7805 C
104 H104    -5.4710    -1.9510     1.1407 H
@<TRIPOS>BOND
1 1 2 1
2 1 3 1
3 1 5 1
4 1 10 1
5 2 4 1
6 2 7 1
7 2 52 1
8 3 4 1
9 3 6 1
10 3 15 1
11 4 14 1
12 4 60 1
13 7 12 1
14 7 40 1
15 7 51 1
16 8 9 1
17 8 10 1
18 8 22 1
19 8 40 1
20 10 11 1
21 10 17 1
22 12 13 1
23 12 21 1
24 12 71 1
25 13 14 1
26 13 28 1
27 13 69 1
28 14 26 1
29 14 63 1
30 15 16 1
31 15 19 1
32 15 24 1
33 17 18 1
34 17 19 1
35 17 22 1
36 19 20 1
37 19 30 1
38 21 36 1
39 21 40 1
40 21 95 1
41 22 23 1
42 22 38 1
43 24 25 1
44 24 26 1
45 24 30 1
46 26 27 1
47 26 28 1
48 28 29 1
49 28 34 1
50 30 31 1
```

51 30 32 1
52 32 33 1
53 32 34 1
54 32 38 1
55 34 35 1
56 34 36 1
57 36 37 1
58 36 38 1
59 38 39 1
60 40 87 1
61 41 42 1
62 41 43 1
63 41 46 1
64 41 52 1
65 42 44 1
66 42 48 1
67 42 49 1
68 43 44 1
69 43 47 1
70 43 60 1
71 44 45 1
72 44 58 1
73 49 50 1
74 49 54 1
75 49 72 1
76 51 52 1
77 51 72 1
78 51 73 1
79 52 53 1
80 54 55 1
81 54 56 1
82 54 62 1
83 56 57 1
84 56 58 1
85 56 67 1
86 58 59 1
87 58 65 1
88 60 61 1
89 60 63 1
90 62 71 1
91 62 72 1
92 62 85 1
93 63 64 1
94 63 65 1
95 65 66 1
96 65 67 1
97 67 68 1
98 67 69 1
99 69 70 1
100 69 71 1
101 71 93 1
102 72 74 1
103 73 74 1
104 73 75 1
105 73 81 1

106 74 76 1
107 74 77 1
108 77 78 1
109 77 83 1
110 77 103 1
111 79 80 1
112 79 81 1
113 79 91 1
114 79 103 1
115 81 82 1
116 81 87 1
117 83 84 1
118 83 85 1
119 83 89 1
120 85 86 1
121 85 93 1
122 87 88 1
123 87 91 1
124 89 90 1
125 89 99 1
126 89 103 1
127 91 92 1
128 91 101 1
129 93 94 1
130 93 97 1
131 95 96 1
132 95 97 1
133 95 101 1
134 97 98 1
135 97 99 1
136 99 100 1
137 99 101 1
138 101 102 1
139 103 104 1

Figure S21. Left: optimized geometry of the $C_{58}H_{46}$ 2D-scaffold generator obtained by fusing three nugget$_{24a}$ molecules. Right: the released (1R,6S)-bicyclo[4.2.0]octane molecule, C_8H_{14}. Cartesian coordinates of its atoms; the first line contains the total charge and multiplicity; the following lines contain the atomic numbers, followed by the x, y, and z coordinates in Å for each one of the atoms. Next, atomic coordinates in Tripos Mol2 file format (.mol2) with the distances also in Å.

$C_{71}H_{52}$ 3D-scaffold

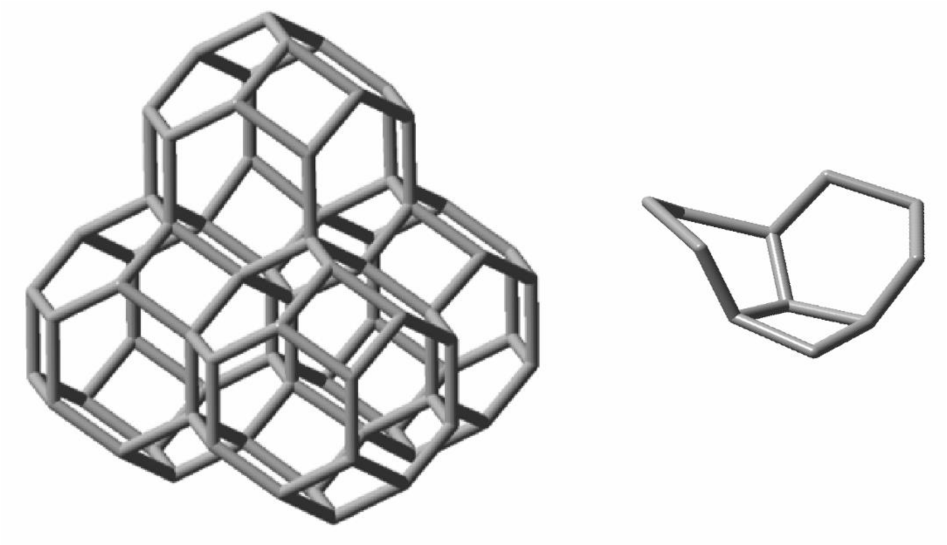

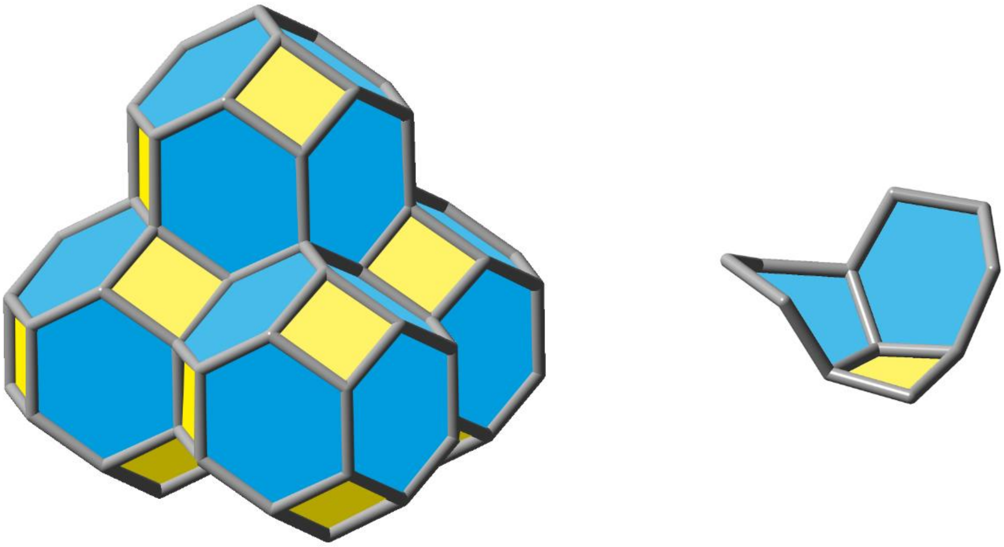

Cartesian Coordinates (Å)

0 1

6	8.836	-0.421	1.571
6	10.299	-0.930	1.694
6	8.571	-0.829	3.046
6	10.033	-1.337	3.169
6	10.821	-1.933	0.728
6	8.503	-1.985	-0.522
6	7.958	-0.938	0.487
6	11.101	-3.388	1.194
1	12.168	-3.649	0.954
6	10.846	-3.778	2.606
1	11.797	-4.218	3.015
6	10.301	-2.731	3.615
1	10.995	-2.679	4.498
6	7.438	-1.736	3.375

6	6.775	-1.884	0.831
6	6.521	-2.274	2.243
6	10.184	-3.927	0.062
1	10.820	-4.441	-0.709
6	7.321	-2.932	-0.178
1	6.627	-2.983	-1.061
6	7.718	-3.191	3.841
1	7.214	-3.360	4.831
6	9.118	-3.677	3.958
1	9.257	-4.070	5.003
6	9.664	-4.724	2.949
1	10.059	-5.609	3.519
6	6.801	-3.729	2.709
1	5.865	-4.152	3.168
6	7.323	-4.732	1.743
1	6.628	-5.615	1.758
6	8.786	-5.241	1.866
1	8.777	-6.362	1.938
6	9.051	-4.833	0.391
1	9.168	-5.763	-0.230
6	7.589	-4.325	0.268
1	7.018	-5.016	-0.410
6	9.904	-2.472	-0.404
6	6.573	2.554	1.988
6	8.036	2.046	2.111
6	6.308	2.147	3.463
6	7.770	1.639	3.586
1	6.581	3.676	1.916
1	6.191	3.077	4.084
6	8.558	1.043	1.145
6	6.240	0.991	-0.105
1	6.102	1.384	-1.149
6	5.695	2.038	0.904
1	5.300	2.923	0.335
6	8.038	0.245	4.032
6	5.175	1.240	3.792
1	4.539	1.755	4.563
6	4.512	1.092	1.248
1	3.562	1.532	0.839
6	4.258	0.702	2.660
1	3.191	0.962	2.900
6	5.058	0.044	0.239
1	4.364	-0.007	-0.644
6	5.455	-0.215	4.258
1	4.951	-0.384	5.248
6	6.856	-0.701	4.375
1	6.994	-1.094	5.420
6	4.538	-0.754	3.126
1	3.603	-1.176	3.585
6	5.060	-1.756	2.160

1	4.365	-2.639	2.175
6	5.326	-1.349	0.685
1	4.755	-2.040	0.007
6	7.641	0.504	0.013
6	10.091	3.503	2.901
6	11.553	2.994	3.024
6	9.825	3.095	4.376
6	11.288	2.587	4.499
1	11.859	3.278	5.177
1	10.099	4.625	2.828
1	9.709	4.025	4.997
1	12.249	3.877	3.009
6	12.075	1.992	2.058
1	13.011	2.414	1.599
6	9.758	1.940	0.808
6	9.213	2.987	1.817
1	8.818	3.872	1.248
6	12.355	0.537	2.524
1	13.422	0.276	2.284
6	12.101	0.146	3.936
1	13.052	-0.294	4.345
6	11.556	1.194	4.945
1	12.250	1.246	5.828
6	8.693	2.189	4.705
1	8.057	2.703	5.476
6	11.438	-0.002	1.392
6	8.973	0.734	5.171
1	8.468	0.565	6.161
6	10.373	0.247	5.288
1	10.511	-0.146	6.333
6	10.919	-0.800	4.279
1	11.313	-1.685	4.849
6	11.158	1.453	0.926
6	10.062	2.335	-0.722
6	11.525	1.827	-0.599
1	10.071	3.457	-0.794
1	12.220	2.710	-0.614
6	12.047	0.824	-1.565
1	12.983	1.246	-2.023
6	9.730	0.772	-2.814
1	9.591	1.165	-3.858
6	9.184	1.819	-1.805
1	8.790	2.704	-2.375
6	12.327	-0.631	-1.099
1	13.394	-0.892	-1.339
6	12.073	-1.021	0.313
1	13.023	-1.461	0.722
6	8.002	0.872	-1.462
1	7.051	1.312	-1.871
6	11.410	-1.170	-2.231

```
1    12.046   -1.684   -3.002
6     8.547   -0.175   -2.471
1     7.853   -0.227   -3.354
6    10.278   -2.076   -1.902
1    10.394   -3.007   -2.522
6     8.815   -1.568   -2.025
1     8.244   -2.260   -2.703
6    11.130    0.285   -2.697
1    11.634    0.454   -3.687
```

.mol2 file

```
@<TRIPOS>MOLECULE
Molecule Name
123 168
SMALL
NO_CHARGES


@<TRIPOS>ATOM
1 C1     8.8359    -0.4215     1.5710 C
2 C2    10.2986    -0.9299     1.6935 C
3 C3     8.5706    -0.8291     3.0462 C
4 C4    10.0331    -1.3373     3.1687 C
5 C5    10.8207    -1.9327     0.7276 C
6 C6     8.5034    -1.9848    -0.5217 C
7 C7     7.9579    -0.9375     0.4873 C
8 C8    11.1006    -3.3877     1.1937 C
9 H9    12.1675    -3.6488     0.9540 H
10 C10    10.8464    -3.7779     2.6060 C
11 H11    11.7968    -4.2179     3.0148 H
12 C12    10.3011    -2.7305     3.6150 C
13 H13    10.9950    -2.6786     4.4980 H
14 C14     7.4384    -1.7356     3.3749 C
15 C15     6.7753    -1.8841     0.8306 C
16 C16     6.5211    -2.2744     2.2430 C
17 C17    10.1835    -3.9266     0.0620 C
18 H18    10.8195    -4.4408    -0.7094 H
19 C19     7.3209    -2.9315    -0.1785 C
20 H20     6.6268    -2.9834    -1.0614 H
21 C21     7.7183    -3.1906     3.8408 C
22 H22     7.2137    -3.3598     4.8309 H
23 C23     9.1185    -3.6771     3.9583 C
24 H24     9.2567    -4.0699     5.0025 H
25 C25     9.6639    -4.7244     2.9494 C
26 H26    10.0585    -5.6092     3.5193 H
27 C27     6.8010    -3.7295     2.7090 C
28 H28     5.8655    -4.1521     3.1675 H
29 C29     7.3233    -4.7322     1.7429 C
30 H30     6.6276    -5.6151     1.7579 H
```

31	C31	8.7859	-5.2406	1.8657	C
32	H32	8.7773	-6.3624	1.9385	H
33	C33	9.0514	-4.8330	0.3907	C
34	H34	9.1675	-5.7632	-0.2296	H
35	C35	7.5889	-4.3247	0.2677	C
36	H36	7.0177	-5.0160	-0.4103	H
37	C37	9.9037	-2.4717	-0.4042	C
38	C38	6.5729	2.5544	1.9882	C
39	C39	8.0356	2.0460	2.1107	C
40	C40	6.3076	2.1468	3.4634	C
41	C41	7.7701	1.6386	3.5859	C
42	H42	6.5814	3.6762	1.9155	H
43	H43	6.1914	3.0770	4.0837	H
44	C44	8.5577	1.0432	1.1448	C
45	C45	6.2404	0.9911	-0.1045	C
46	H46	6.1020	1.3840	-1.1486	H
47	C47	5.6949	2.0384	0.9045	C
48	H48	5.3002	2.9233	0.3346	H
49	C49	8.0381	0.2454	4.0322	C
50	C50	5.1754	1.2403	3.7921	C
51	H51	4.5392	1.7547	4.5633	H
52	C52	4.5123	1.0918	1.2478	C
53	H53	3.5618	1.5318	0.8389	H
54	C54	4.2581	0.7015	2.6602	C
55	H55	3.1911	0.9624	2.8997	H
56	C56	5.0579	0.0444	0.2387	C
57	H57	4.3638	-0.0075	-0.6442	H
58	C58	5.4553	-0.2147	4.2580	C
59	H59	4.9507	-0.3839	5.2481	H
60	C60	6.8555	-0.7012	4.3755	C
61	H61	6.9937	-1.0940	5.4197	H
62	C62	4.5380	-0.7536	3.1262	C
63	H63	3.6025	-1.1762	3.5847	H
64	C64	5.0603	-1.7563	2.1601	C
65	H65	4.3646	-2.6392	2.1751	H
66	C66	5.3259	-1.3488	0.6849	C
67	H67	4.7547	-2.0401	0.0069	H
68	C68	7.6407	0.5042	0.0130	C
69	C69	10.0906	3.5028	2.9011	C
70	C70	11.5533	2.9944	3.0236	C
71	C71	9.8253	3.0952	4.3763	C
72	C72	11.2878	2.5870	4.4988	C
73	H73	11.8589	3.2783	5.1768	H
74	H74	10.0991	4.6246	2.8284	H
75	H75	9.7091	4.0254	4.9966	H
76	H76	12.2488	3.8775	3.0086	H
77	C77	12.0754	1.9916	2.0577	C
78	H78	13.0109	2.4142	1.5993	H
79	C79	9.7581	1.9395	0.8084	C
80	C80	9.2126	2.9868	1.8174	C

81 H81 8.8179 3.8717 1.2475 H
82 C82 12.3553 0.5366 2.5238 C
83 H83 13.4222 0.2755 2.2841 H
84 C84 12.1011 0.1464 3.9361 C
85 H85 13.0515 -0.2936 4.3449 H
86 C86 11.5558 1.1938 4.9451 C
87 H87 12.2497 1.2457 5.8281 H
88 C88 8.6931 2.1887 4.7050 C
89 H89 8.0569 2.7031 5.4762 H
90 C90 11.4382 -0.0023 1.3921 C
91 C91 8.9730 0.7337 5.1709 C
92 H92 8.4684 0.5645 6.1610 H
93 C93 10.3732 0.2472 5.2884 C
94 H94 10.5114 -0.1456 6.3326 H
95 C95 10.9186 -0.8001 4.2795 C
96 H96 11.3132 -1.6849 4.8494 H
97 C97 11.1584 1.4526 0.9259 C
98 C98 10.0623 2.3350 -0.7216 C
99 C99 11.5250 1.8266 -0.5991 C
100 H100 10.0708 3.4568 -0.7943 H
101 H101 12.2205 2.7097 -0.6141 H
102 C102 12.0471 0.8238 -1.5650 C
103 H103 12.9826 1.2464 -2.0234 H
104 C104 9.7298 0.7717 -2.8143 C
105 H105 9.5914 1.1646 -3.8584 H
106 C106 9.1843 1.8190 -1.8053 C
107 H107 8.7896 2.7039 -2.3752 H
108 C108 12.3270 -0.6312 -1.0989 C
109 H109 13.3939 -0.8923 -1.3386 H
110 C110 12.0728 -1.0214 0.3134 C
111 H111 13.0232 -1.4614 0.7222 H
112 C112 8.0017 0.8724 -1.4620 C
113 H113 7.0512 1.3124 -1.8709 H
114 C114 11.4099 -1.1701 -2.2306 C
115 H115 12.0459 -1.6843 -3.0020 H
116 C116 8.5473 -0.1750 -2.4711 C
117 H117 7.8532 -0.2269 -3.3540 H
118 C118 10.2778 -2.0765 -1.9019 C
119 H119 10.3939 -3.0067 -2.5222 H
120 C120 8.8153 -1.5682 -2.0249 C
121 H121 8.2441 -2.2595 -2.7029 H
122 C122 11.1301 0.2848 -2.6968 C
123 H123 11.6345 0.4543 -3.6870 H
@<TRIPOS>BOND
1 1 2 1
2 1 3 1
3 1 7 1
4 1 44 1
5 2 4 1
6 2 5 1

7 2 90 1
8 3 4 1
9 3 14 1
10 3 49 1
11 4 12 1
12 4 95 1
13 5 8 1
14 5 37 1
15 5 110 1
16 6 7 1
17 6 19 1
18 6 37 1
19 6 120 1
20 7 15 1
21 7 68 1
22 8 9 1
23 8 10 1
24 8 17 1
25 10 11 1
26 10 12 1
27 10 25 1
28 12 13 1
29 12 23 1
30 14 16 1
31 14 21 1
32 14 60 1
33 15 16 1
34 15 19 1
35 15 66 1
36 16 27 1
37 16 64 1
38 17 18 1
39 17 33 1
40 17 37 1
41 19 20 1
42 19 35 1
43 21 22 1
44 21 23 1
45 21 27 1
46 23 24 1
47 23 25 1
48 25 26 1
49 25 31 1
50 27 28 1
51 27 29 1
52 29 30 1
53 29 31 1
54 29 35 1
55 31 32 1
56 31 33 1

57 33 34 1
58 33 35 1
59 35 36 1
60 37 118 1
61 38 42 1
62 38 39 1
63 38 40 1
64 38 47 1
65 39 41 1
66 39 44 1
67 39 80 1
68 40 43 1
69 40 41 1
70 40 50 1
71 41 49 1
72 41 88 1
73 44 68 1
74 44 79 1
75 45 46 1
76 45 47 1
77 45 56 1
78 45 68 1
79 47 48 1
80 47 52 1
81 49 60 1
82 49 91 1
83 50 51 1
84 50 54 1
85 50 58 1
86 52 53 1
87 52 54 1
88 52 56 1
89 54 55 1
90 54 62 1
91 56 57 1
92 56 66 1
93 58 59 1
94 58 60 1
95 58 62 1
96 60 61 1
97 62 63 1
98 62 64 1
99 64 65 1
100 64 66 1
101 66 67 1
102 68 112 1
103 69 74 1
104 69 70 1
105 69 71 1
106 69 80 1

107 70 76 1
108 70 72 1
109 70 77 1
110 71 75 1
111 71 72 1
112 71 88 1
113 72 73 1
114 72 86 1
115 77 78 1
116 77 82 1
117 77 97 1
118 79 80 1
119 79 97 1
120 79 98 1
121 80 81 1
122 82 83 1
123 82 84 1
124 82 90 1
125 84 85 1
126 84 86 1
127 84 95 1
128 86 87 1
129 86 93 1
130 88 89 1
131 88 91 1
132 90 97 1
133 90 110 1
134 91 92 1
135 91 93 1
136 93 94 1
137 93 95 1
138 95 96 1
139 97 99 1
140 98 100 1
141 98 99 1
142 98 106 1
143 99 101 1
144 99 102 1
145 102 103 1
146 102 108 1
147 102 122 1
148 104 105 1
149 104 106 1
150 104 116 1
151 104 122 1
152 106 107 1
153 106 112 1
154 108 109 1
155 108 110 1
156 108 114 1

157 110 111 1
158 112 113 1
159 112 116 1
160 114 115 1
161 114 118 1
162 114 122 1
163 116 117 1
164 116 120 1
165 118 119 1
166 118 120 1
167 120 121 1
168 122 123 1

Figure S22. Left: optimized geometry of the $C_{71}H_{52}$ 3D-scaffold generator obtained from the growth of nugget$_{24a}$. Right: the released (1s,1aS,4ar,7aR)-nonahydro-1H-cyclobuta[de]naphthalene molecule, $C_{11}H_{18}$, which is the product of the idealized third fusion reaction. Cartesian coordinates of its atoms; the first line contains the total charge and multiplicity; the following lines contain the atomic numbers, followed by the x, y, and z coordinates in Å for each one of the atoms. Next, atomic coordinates in Tripos Mol2 file format (.mol2) with the distances also in Å.

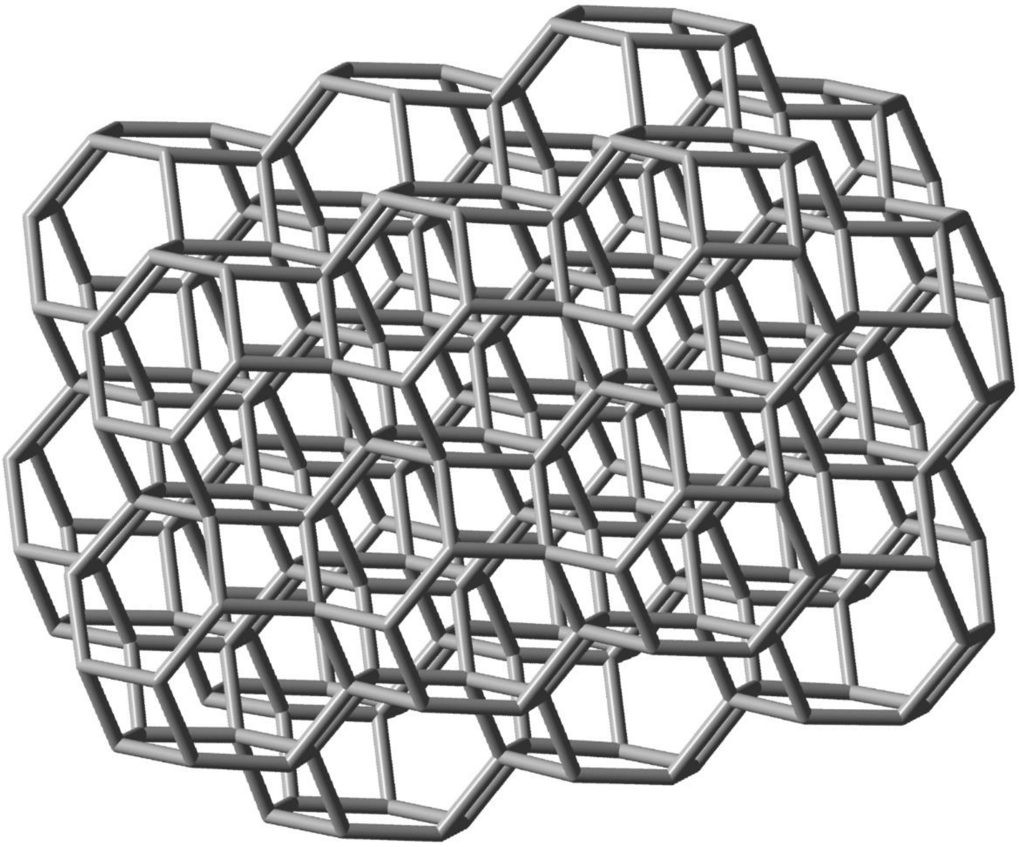

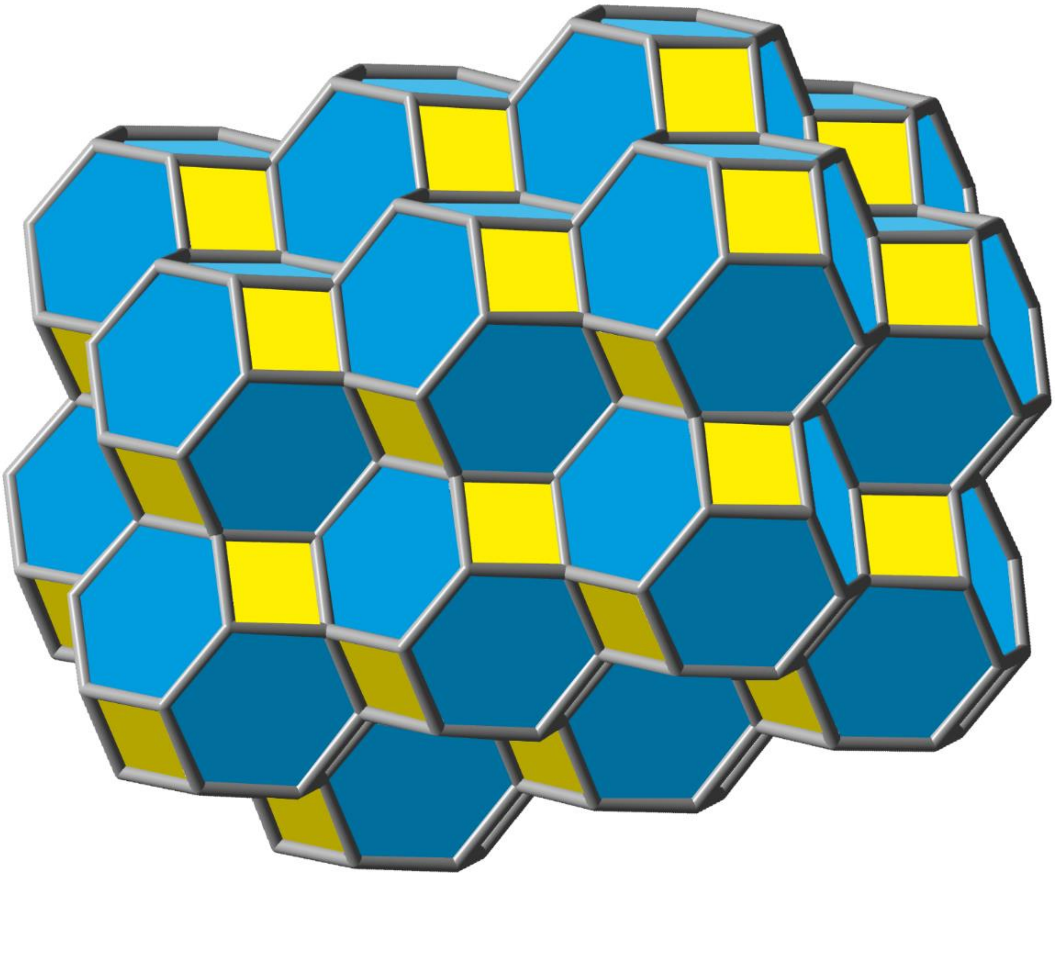

Cartesian Coordinates (Å)

0 1

6	-0.036	0.044	-0.111
6	1.522	0.030	-0.072
6	0.009	1.601	-0.058
6	1.546	1.568	-0.055
6	2.274	-0.764	1.009
6	-0.043	-1.515	2.087
6	-0.808	-0.708	0.988
6	3.051	-0.022	2.110
6	3.078	1.516	2.125
6	2.325	2.311	1.044
6	-0.746	2.373	1.036
6	-1.577	0.077	2.099
6	-1.577	1.615	2.115
6	2.268	-0.785	3.190
6	-0.813	-0.730	3.198
6	0.033	3.112	2.135
6	1.570	3.084	2.137
6	2.322	2.290	3.218
6	-0.751	2.350	3.214
6	-0.000	1.557	4.294
6	1.536	1.525	4.296
6	1.511	-0.012	4.282
6	-0.047	-0.001	4.315
6	1.494	-1.573	2.090
6	3.031	-0.004	-2.281
6	4.590	-0.007	-2.232
6	5.341	-0.801	-1.152
6	3.021	-1.582	-0.088
6	2.253	-0.771	-1.192
6	6.117	-0.059	-0.050
6	6.145	1.481	-0.034
6	5.338	-0.834	1.026
6	5.394	2.257	1.059
6	4.614	1.485	2.135
6	4.587	-0.054	2.118
6	4.563	-1.622	-0.079
6	6.117	-0.028	-4.428
6	7.683	-0.055	-4.421
6	8.436	-0.851	-3.338
6	6.096	-1.608	-2.250
6	5.331	-0.799	-3.351
6	9.193	-0.081	-2.214
6	9.223	1.456	-2.195
6	8.409	-0.849	-1.139
6	8.465	2.225	-1.101
6	7.682	1.456	-0.025
6	7.653	-0.082	-0.042

6	7.636	-1.633	-2.242
6	3.102	3.033	4.316
6	2.315	2.267	5.392
6	1.581	4.574	6.498
6	1.553	3.038	6.482
6	6.173	3.000	2.157
6	5.394	2.229	3.233
6	4.639	3.005	4.323
6	9.245	2.968	-0.003
6	8.462	2.200	1.074
6	7.710	2.972	2.169
6	5.335	-0.847	3.203
6	3.011	-1.620	4.276
6	6.114	-0.104	4.300
6	6.147	1.434	4.315
6	5.345	-0.893	5.402
6	2.240	-0.832	5.389
6	5.391	2.209	5.404
6	3.066	1.470	6.471
6	4.602	1.441	6.477
6	4.587	-0.117	6.495
6	3.021	-0.087	6.489
6	4.551	-1.650	4.284
6	8.402	-0.875	1.042
6	6.084	-1.673	2.112
6	9.182	-0.132	2.140
6	9.214	1.406	2.156
6	8.422	-0.930	3.243
6	8.462	2.177	3.252
6	7.684	1.404	4.330
6	7.672	-0.159	4.349
6	7.626	-1.689	2.122
6	10.750	-0.125	-2.248
6	10.760	1.432	-2.193
6	11.489	-0.906	-1.146
6	9.153	-1.688	-0.056
6	12.281	-0.150	-0.031
6	12.337	1.387	-0.014
6	11.539	2.175	-1.097
6	11.484	-0.930	1.064
6	10.782	2.942	-0.000
6	11.534	2.151	1.082
6	10.751	1.384	2.159
6	10.740	-0.175	2.179
6	10.692	-1.686	-0.052
6	6.173	2.954	6.499
6	6.201	4.492	6.515
6	5.408	2.160	7.601
6	2.279	2.217	7.590
6	5.465	5.290	7.634

6	2.336	5.346	7.623
6	3.101	4.537	8.685
6	4.664	4.509	8.691
6	4.635	2.946	8.674
6	3.073	2.975	8.668
6	9.245	2.922	4.346
6	9.273	4.459	4.362
6	8.493	2.125	5.456
6	8.550	5.258	5.489
6	7.759	4.479	6.561
6	7.731	2.909	6.544
6	10.810	4.477	0.016
6	12.356	2.887	2.182
6	12.384	4.427	2.199
6	11.589	5.216	1.115
6	11.558	2.106	3.280
6	11.614	5.213	3.313
6	10.831	4.444	4.394
6	10.803	2.879	4.377
6	5.378	2.301	-5.484
6	4.642	3.098	-4.365
6	6.157	1.530	-4.376
6	5.396	2.302	-3.287
6	4.670	4.636	-4.349
6	6.208	4.644	-6.524
6	6.180	3.082	-6.540
6	5.452	5.381	-3.254
6	6.204	4.585	-2.173
6	6.176	3.047	-2.190
6	7.694	1.503	-4.370
6	7.742	3.053	-6.535
6	8.508	2.244	-5.473
6	6.241	6.149	-4.326
6	7.771	4.616	-6.518
6	8.473	2.249	-3.275
6	7.713	3.019	-2.182
6	7.741	4.557	-2.166
6	9.262	3.015	-4.348
6	9.290	4.552	-4.331
6	8.528	5.323	-3.242
6	7.777	6.120	-4.320
6	8.564	5.373	-5.439
6	5.435	5.430	-5.451
6	2.293	2.331	-3.339
6	1.570	3.131	-2.212
6	3.075	1.557	-2.229
6	2.325	2.334	-1.134
6	1.597	4.668	-2.196
6	3.112	4.681	-4.394
6	3.083	3.110	-4.411

6	2.381	5.412	-1.101
6	3.133	4.617	-0.019
6	3.105	3.079	-0.035
6	4.612	1.531	-2.215
6	3.158	6.186	-2.179
6	5.393	2.275	-1.116
6	4.641	3.051	-0.023
6	4.669	4.589	-0.006
6	5.449	5.361	-1.083
6	4.696	6.156	-2.165
6	2.349	5.464	-3.305
6	-0.771	2.376	-1.163
6	-1.541	3.162	-0.048
6	-1.514	4.702	-0.032
6	0.040	4.710	-2.227
6	0.012	3.144	-2.244
6	-0.691	5.438	1.068
6	0.061	4.648	2.151
6	0.092	6.205	-0.009
6	1.598	4.621	2.154
6	2.381	5.390	1.076
6	1.629	6.183	-0.006
6	-0.716	5.483	-1.130
6	8.521	5.300	-1.068
6	9.272	4.506	0.013
6	9.307	6.065	-2.146
6	11.594	5.239	-1.063
6	10.843	6.033	-2.144
6	5.449	5.333	1.092
6	6.201	4.538	2.173
6	6.229	6.104	0.016
6	7.737	4.510	2.185
6	8.517	5.279	1.107
6	7.765	6.074	0.025
6	2.377	5.364	3.251
6	3.129	4.570	4.332
6	3.161	6.133	2.175
6	4.667	4.543	4.340
6	5.450	5.314	3.266
6	4.697	6.109	2.184
6	4.729	7.694	-2.150
6	6.257	7.707	-4.344
6	5.508	8.437	-1.053
6	6.256	7.644	0.033
6	6.292	9.240	-2.133
6	7.822	7.678	-4.338
6	7.792	7.612	0.041
6	9.332	7.602	-2.131
6	8.575	8.374	-1.039
6	7.832	9.209	-2.125

6	8.603	8.422	-3.238
6	5.498	8.483	-3.251
6	1.661	7.721	0.010
6	3.170	7.748	-2.198
6	2.440	8.464	1.108
6	3.189	7.672	2.192
6	3.216	9.278	0.028
6	4.725	7.648	2.200
6	5.505	8.423	1.125
6	4.759	9.263	0.038
6	2.420	8.519	-1.093
6	-1.494	6.202	2.164
6	-0.696	5.415	3.247
6	-1.439	7.740	2.181
6	0.103	7.764	-0.028
6	-0.646	8.495	3.297
6	0.092	7.715	4.398
6	0.083	6.157	4.343
6	0.151	9.275	2.202
6	1.619	6.134	4.345
6	1.650	7.670	4.364
6	2.434	8.438	3.289
6	1.690	9.277	2.206
6	-0.641	8.520	1.087
6	8.569	8.353	1.142
6	9.321	7.559	2.223
6	9.297	6.021	2.205
6	9.349	9.162	0.061
6	10.890	7.590	-2.164
6	10.834	5.988	2.209
6	10.879	7.545	2.262
6	12.419	5.974	0.036
6	12.420	7.513	0.052
6	11.650	8.297	1.163
6	10.886	9.104	0.065
6	11.656	8.320	-1.047
6	5.502	8.390	3.302
6	6.253	7.596	4.383
6	6.231	6.058	4.365
6	6.280	9.212	2.229
6	8.518	5.255	3.285
6	7.768	6.032	4.379
6	7.812	7.593	4.432
6	8.590	8.361	3.343
6	7.822	9.172	2.239
6	2.370	5.341	5.425
6	2.407	8.440	5.488
6	3.160	7.645	6.571
6	3.149	6.087	6.520
6	3.206	9.223	4.392

6	5.446	5.287	5.437
6	4.686	6.060	6.526
6	4.726	7.618	6.577
6	5.512	8.389	5.501
6	4.746	9.198	4.400
1	-0.384	-0.281	-1.093
1	-0.414	-2.545	2.075
1	-1.513	-1.384	0.495
1	-2.620	-0.256	2.093
1	-2.604	1.984	2.116
1	-1.521	-1.416	3.674
1	-0.398	-0.347	5.289
1	1.824	-2.613	2.081
1	2.692	-0.328	-3.268
1	2.647	-2.610	-0.097
1	1.545	-1.445	-1.682
1	4.901	-2.660	-0.092
1	5.772	-0.353	-5.413
1	8.024	-0.392	-5.404
1	9.119	-1.549	-3.829
1	5.730	-2.638	-2.265
1	4.629	-1.475	-3.847
1	7.969	-2.675	-2.253
1	2.641	-2.648	4.265
1	6.021	-1.605	5.883
1	1.532	-1.515	5.865
1	4.920	-0.475	7.473
1	2.667	-0.433	7.464
1	4.879	-2.693	4.276
1	5.709	-2.699	2.104
1	9.105	-1.640	3.717
1	7.998	-0.515	5.328
1	7.963	-2.729	2.108
1	11.089	-0.463	-3.229
1	12.172	-1.606	-1.637
1	8.786	-2.716	-0.069
1	13.312	-0.520	-0.032
1	13.377	1.719	-0.008
1	12.165	-1.642	1.542
1	11.075	-0.534	3.154
1	11.025	-2.728	-0.063
1	6.085	1.450	8.081
1	1.573	1.533	8.064
1	6.167	5.964	8.129
1	1.654	6.045	8.112
1	2.761	4.876	9.669
1	5.008	4.835	9.677
1	4.968	2.588	9.653
1	2.720	2.628	9.645
1	9.176	1.416	5.928

1	9.258	5.932	5.976
1	8.100	4.805	7.548
1	8.059	2.551	7.524
1	13.385	2.519	2.179
1	13.426	4.758	2.203
1	12.237	1.395	3.758
1	12.319	5.889	3.806
1	11.181	4.770	5.377
1	11.140	2.519	5.353
1	4.677	1.626	-5.979
1	5.875	5.003	-7.503
1	5.835	2.756	-7.527
1	8.082	2.715	-7.519
1	9.189	1.546	-5.962
1	8.123	4.963	-7.495
1	9.270	6.058	-5.913
1	4.758	6.140	-5.930
1	1.585	1.658	-3.826
1	2.784	5.039	-5.373
1	2.743	2.785	-5.398
1	1.667	6.173	-3.777
1	-1.476	1.700	-1.655
1	-2.583	2.831	-0.052
1	-2.543	5.070	-0.028
1	-0.298	5.069	-3.203
1	-0.338	2.818	-3.227
1	-1.395	6.194	-1.607
1	5.924	8.066	-5.322
1	5.963	10.283	-2.126
1	8.176	8.023	-5.313
1	8.202	10.238	-2.113
1	9.311	9.105	-3.714
1	4.822	9.195	-3.733
1	2.844	8.105	-3.177
1	2.880	10.318	0.042
1	5.134	10.288	0.047
1	1.737	9.229	-1.567
1	-2.534	5.871	2.158
1	-2.469	8.109	2.183
1	-0.233	8.123	-1.004
1	-1.330	9.196	3.787
1	-0.247	8.052	5.379
1	-0.183	10.318	2.213
1	2.057	10.305	2.219
1	-1.322	9.231	0.609
1	9.019	10.203	0.070
1	11.241	7.936	-3.138
1	11.227	7.870	3.244
1	13.447	5.605	0.034
1	13.463	7.845	0.058

1	12.356	8.973	1.656
1	11.258	10.134	0.076
1	12.364	9.006	-1.522
1	5.942	10.250	2.243
1	8.150	7.917	5.418
1	9.298	9.035	3.832
1	8.196	10.199	2.247
1	1.724	9.138	5.979
1	2.819	7.982	7.553
1	2.873	10.264	4.402
1	5.071	7.943	7.563
1	6.213	9.065	5.997
1	5.112	10.228	4.414
6	-0.730	5.412	5.445
1	-1.411	6.113	5.936
6	0.024	4.617	6.528
1	-0.315	4.956	7.511
6	-1.523	4.655	4.328
1	-2.553	5.024	4.328
6	-0.005	3.051	6.512
1	-0.356	2.704	7.487
6	-0.785	2.307	5.412
1	-1.491	1.621	5.888
6	-1.551	3.115	4.311
1	-2.593	2.784	4.304
6	12.394	4.474	-2.161
1	13.436	4.805	-2.153
6	11.572	2.177	-3.295
1	12.253	1.477	-3.786
6	11.629	5.283	-3.261
1	12.335	5.969	-3.737
6	10.819	2.973	-4.378
1	11.158	2.634	-5.360
6	10.848	4.539	-4.361
1	11.199	4.886	-5.336
6	12.366	2.934	-2.177
1	13.395	2.566	-2.177

.mol2 file

@<TRIPOS>MOLECULE
Molecule Name
384 588
SMALL
NO_CHARGES

@<TRIPOS>ATOM
1 C1 -0.0364 0.0441 -0.1109 C
2 C2 1.5221 0.0304 -0.0721 C
3 C3 0.0091 1.6012 -0.0580 C

4	C4	1.5455	1.5680	-0.0551	C
5	C5	2.2739	-0.7638	1.0090	C
6	C6	-0.0434	-1.5151	2.0867	C
7	C7	-0.8075	-0.7080	0.9879	C
8	C8	3.0508	-0.0221	2.1096	C
9	C9	3.0777	1.5160	2.1254	C
10	C10	2.3254	2.3107	1.0435	C
11	C11	-0.7462	2.3728	1.0355	C
12	C12	-1.5769	0.0767	2.0992	C
13	C13	-1.5766	1.6153	2.1151	C
14	C14	2.2678	-0.7846	3.1900	C
15	C15	-0.8128	-0.7304	3.1980	C
16	C16	0.0330	3.1125	2.1345	C
17	C17	1.5704	3.0838	2.1373	C
18	C18	2.3221	2.2897	3.2184	C
19	C19	-0.7515	2.3503	3.2142	C
20	C20	-0.0003	1.5569	4.2944	C
21	C21	1.5362	1.5247	4.2962	C
22	C22	1.5115	-0.0120	4.2821	C
23	C23	-0.0466	-0.0008	4.3153	C
24	C24	1.4942	-1.5726	2.0899	C
25	C25	3.0306	-0.0039	-2.2815	C
26	C26	4.5896	-0.0066	-2.2325	C
27	C27	5.3411	-0.8006	-1.1518	C
28	C28	3.0206	-1.5823	-0.0882	C
29	C29	2.2527	-0.7710	-1.1925	C
30	C30	6.1172	-0.0588	-0.0502	C
31	C31	6.1451	1.4807	-0.0340	C
32	C32	5.3378	-0.8335	1.0255	C
33	C33	5.3936	2.2566	1.0586	C
34	C34	4.6142	1.4851	2.1346	C
35	C35	4.5866	-0.0544	2.1178	C
36	C36	4.5625	-1.6223	-0.0786	C
37	C37	6.1167	-0.0276	-4.4276	C
38	C38	7.6825	-0.0546	-4.4212	C
39	C39	8.4357	-0.8505	-3.3380	C
40	C40	6.0962	-1.6082	-2.2500	C
41	C41	5.3307	-0.7994	-3.3508	C
42	C42	9.1927	-0.0809	-2.2141	C
43	C43	9.2235	1.4556	-2.1952	C
44	C44	8.4086	-0.8491	-1.1386	C
45	C45	8.4652	2.2252	-1.1010	C
46	C46	7.6817	1.4559	-0.0247	C
47	C47	7.6531	-0.0821	-0.0418	C
48	C48	7.6364	-1.6333	-2.2423	C
49	C49	3.1016	3.0330	4.3158	C
50	C50	2.3147	2.2670	5.3922	C
51	C51	1.5811	4.5745	6.4982	C
52	C52	1.5534	3.0384	6.4818	C
53	C53	6.1734	3.0002	2.1566	C

54	C54	5.3941	2.2287	3.2327	C
55	C55	4.6389	3.0045	4.3234	C
56	C56	9.2446	2.9683	-0.0034	C
57	C57	8.4618	2.1997	1.0738	C
58	C58	7.7095	2.9722	2.1688	C
59	C59	5.3349	-0.8472	3.2029	C
60	C60	3.0108	-1.6196	4.2764	C
61	C61	6.1142	-0.1041	4.3001	C
62	C62	6.1472	1.4336	4.3152	C
63	C63	5.3447	-0.8935	5.4015	C
64	C64	2.2399	-0.8321	5.3890	C
65	C65	5.3909	2.2092	5.4044	C
66	C66	3.0659	1.4702	6.4705	C
67	C67	4.6023	1.4414	6.4769	C
68	C68	4.5866	-0.1171	6.4949	C
69	C69	3.0209	-0.0874	6.4886	C
70	C70	4.5509	-1.6504	4.2839	C
71	C71	8.4024	-0.8747	1.0424	C
72	C72	6.0838	-1.6732	2.1121	C
73	C73	9.1818	-0.1315	2.1400	C
74	C74	9.2139	1.4059	2.1559	C
75	C75	8.4224	-0.9299	3.2431	C
76	C76	8.4620	2.1768	3.2515	C
77	C77	7.6845	1.4039	4.3296	C
78	C78	7.6723	-0.1588	4.3486	C
79	C79	7.6262	-1.6891	2.1219	C
80	C80	10.7503	-0.1252	-2.2475	C
81	C81	10.7602	1.4324	-2.1932	C
82	C82	11.4892	-0.9056	-1.1463	C
83	C83	9.1530	-1.6877	-0.0559	C
84	C84	12.2814	-0.1503	-0.0308	C
85	C85	12.3366	1.3873	-0.0137	C
86	C86	11.5390	2.1749	-1.0965	C
87	C87	11.4839	-0.9303	1.0638	C
88	C88	10.7821	2.9415	-0.0004	C
89	C89	11.5336	2.1509	1.0822	C
90	C90	10.7505	1.3838	2.1592	C
91	C91	10.7398	-0.1747	2.1787	C
92	C92	10.6917	-1.6857	-0.0517	C
93	C93	6.1731	2.9542	6.4990	C
94	C94	6.2009	4.4916	6.5154	C
95	C95	5.4084	2.1598	7.6011	C
96	C96	2.2792	2.2170	7.5897	C
97	C97	5.4649	5.2897	7.6343	C
98	C98	2.3355	5.3456	7.6230	C
99	C99	3.1009	4.5372	8.6850	C
100	C100	4.6636	4.5090	8.6906	C
101	C101	4.6354	2.9465	8.6740	C
102	C102	3.0728	2.9747	8.6685	C
103	C103	9.2453	2.9218	4.3457	C

104	C104	9.2731	4.4587	4.3622	C
105	C105	8.4930	2.1252	5.4558	C
106	C106	8.5496	5.2581	5.4893	C
107	C107	7.7594	4.4794	6.5609	C
108	C108	7.7310	2.9090	6.5441	C
109	C109	10.8098	4.4768	0.0161	C
110	C110	12.3560	2.8870	2.1823	C
111	C111	12.3838	4.4274	2.1989	C
112	C112	11.5890	5.2163	1.1153	C
113	C113	11.5581	2.1056	3.2800	C
114	C114	11.6142	5.2134	3.3135	C
115	C115	10.8310	4.4444	4.3943	C
116	C116	10.8027	2.8789	4.3774	C
117	C117	5.3782	2.3006	-5.4844	C
118	C118	4.6420	3.0983	-4.3653	C
119	C119	6.1573	1.5296	-4.3761	C
120	C120	5.3964	2.3023	-3.2872	C
121	C121	4.6697	4.6357	-4.3488	C
122	C122	6.2079	4.6441	-6.5236	C
123	C123	6.1797	3.0816	-6.5404	C
124	C124	5.4519	5.3806	-3.2541	C
125	C125	6.2039	4.5852	-2.1732	C
126	C126	6.1762	3.0466	-2.1897	C
127	C127	7.6938	1.5029	-4.3696	C
128	C128	7.7423	3.0533	-6.5348	C
129	C129	8.5075	2.2445	-5.4728	C
130	C130	6.2407	6.1486	-4.3263	C
131	C131	7.7705	4.6158	-6.5179	C
132	C132	8.4728	2.2488	-3.2747	C
133	C133	7.7135	3.0194	-2.1820	C
134	C134	7.7412	4.5568	-2.1655	C
135	C135	9.2619	3.0154	-4.3478	C
136	C136	9.2897	4.5516	-4.3313	C
137	C137	8.5283	5.3229	-3.2416	C
138	C138	7.7772	6.1199	-4.3199	C
139	C139	8.5641	5.3733	-5.4391	C
140	C140	5.4346	5.4304	-5.4506	C
141	C141	2.2932	2.3314	-3.3392	C
142	C142	1.5696	3.1305	-2.2119	C
143	C143	3.0747	1.5574	-2.2290	C
144	C144	2.3251	2.3345	-1.1342	C
145	C145	1.5972	4.6675	-2.1955	C
146	C146	3.1117	4.6807	-4.3939	C
147	C147	3.0835	3.1103	-4.4108	C
148	C148	2.3805	5.4125	-1.1013	C
149	C149	3.1331	4.6171	-0.0186	C
150	C150	3.1054	3.0789	-0.0351	C
151	C151	4.6121	1.5313	-2.2146	C
152	C152	3.1581	6.1856	-2.1794	C
153	C153	5.3930	2.2754	-1.1156	C

154	C154	4.6415	3.0512	-0.0230	C
155	C155	4.6692	4.5893	-0.0065	C
156	C156	5.4486	5.3609	-1.0825	C
157	C157	4.6955	6.1560	-2.1649	C
158	C158	2.3495	5.4642	-3.3055	C
159	C159	-0.7715	2.3755	-1.1628	C
160	C160	-1.5412	3.1615	-0.0482	C
161	C161	-1.5135	4.7019	-0.0317	C
162	C162	0.0397	4.7100	-2.2270	C
163	C163	0.0116	3.1445	-2.2437	C
164	C164	-0.6911	5.4383	1.0682	C
165	C165	0.0606	4.6478	2.1509	C
166	C166	0.0920	6.2054	-0.0089	C
167	C167	1.5980	4.6211	2.1538	C
168	C168	2.3808	5.3896	1.0764	C
169	C169	1.6286	6.1833	-0.0057	C
170	C170	-0.7156	5.4833	-1.1296	C
171	C171	8.5207	5.2998	-1.0679	C
172	C172	9.2724	4.5056	0.0131	C
173	C173	9.3067	6.0650	-2.1455	C
174	C174	11.5943	5.2390	-1.0634	C
175	C175	10.8433	6.0327	-2.1435	C
176	C176	5.4490	5.3328	1.0916	C
177	C177	6.2012	4.5382	2.1731	C
178	C178	6.2285	6.1045	0.0157	C
179	C179	7.7373	4.5104	2.1853	C
180	C180	8.5173	5.2787	1.1069	C
181	C181	7.7650	6.0735	0.0250	C
182	C182	2.3775	5.3643	3.2512	C
183	C183	3.1293	4.5702	4.3323	C
184	C184	3.1609	6.1335	2.1748	C
185	C185	4.6666	4.5431	4.3399	C
186	C186	5.4497	5.3142	3.2657	C
187	C187	4.6975	6.1088	2.1841	C
188	C188	4.7285	7.6937	-2.1498	C
189	C189	6.2565	7.7071	-4.3443	C
190	C190	5.5077	8.4367	-1.0525	C
191	C191	6.2561	7.6439	0.0325	C
192	C192	6.2919	9.2401	-2.1332	C
193	C193	7.8222	7.6775	-4.3377	C
194	C194	7.7920	7.6116	0.0410	C
195	C195	9.3315	7.6017	-2.1312	C
196	C196	8.5751	8.3742	-1.0392	C
197	C197	7.8321	9.2094	-2.1254	C
198	C198	8.6032	8.4221	-3.2380	C
199	C199	5.4982	8.4833	-3.2509	C
200	C200	1.6606	7.7207	0.0102	C
201	C201	3.1703	7.7482	-2.1984	C
202	C202	2.4401	8.4640	1.1077	C
203	C203	3.1894	7.6715	2.1919	C

204	C204	3.2163	9.2784	0.0281	C
205	C205	4.7254	7.6483	2.2003	C
206	C206	5.5047	8.4230	1.1247	C
207	C207	4.7587	9.2627	0.0382	C
208	C208	2.4200	8.5192	-1.0930	C
209	C209	-1.4941	6.2021	2.1641	C
210	C210	-0.6964	5.4145	3.2469	C
211	C211	-1.4388	7.7397	2.1812	C
212	C212	0.1026	7.7639	-0.0283	C
213	C213	-0.6465	8.4951	3.2967	C
214	C214	0.0924	7.7146	4.3978	C
215	C215	0.0826	6.1571	4.3435	C
216	C216	0.1510	9.2750	2.2020	C
217	C217	1.6193	6.1340	4.3454	C
218	C218	1.6500	7.6705	4.3642	C
219	C219	2.4340	8.4385	3.2886	C
220	C220	1.6896	9.2771	2.2059	C
221	C221	-0.6413	8.5196	1.0866	C
222	C222	8.5689	8.3531	1.1417	C
223	C223	9.3206	7.5589	2.2227	C
224	C224	9.2972	6.0213	2.2055	C
225	C225	9.3488	9.1620	0.0610	C
226	C226	10.8897	7.5904	-2.1642	C
227	C227	10.8336	5.9878	2.2088	C
228	C228	10.8791	7.5450	2.2619	C
229	C229	12.4195	5.9739	0.0358	C
230	C230	12.4198	7.5125	0.0520	C
231	C231	11.6504	8.2971	1.1634	C
232	C232	10.8864	9.1044	0.0646	C
233	C233	11.6558	8.3198	-1.0468	C
234	C234	5.5015	8.3901	3.3018	C
235	C235	6.2531	7.5962	4.3825	C
236	C236	6.2306	6.0583	4.3646	C
237	C237	6.2801	9.2118	2.2288	C
238	C238	8.5175	5.2548	3.2845	C
239	C239	7.7680	6.0321	4.3792	C
240	C240	7.8121	7.5934	4.4318	C
241	C241	8.5900	8.3605	3.3429	C
242	C242	7.8221	9.1718	2.2386	C
243	C243	2.3701	5.3410	5.4249	C
244	C244	2.4069	8.4402	5.4880	C
245	C245	3.1603	7.6446	6.5712	C
246	C246	3.1491	6.0872	6.5197	C
247	C247	3.2062	9.2229	4.3922	C
248	C248	5.4464	5.2875	5.4373	C
249	C249	4.6856	6.0604	6.5261	C
250	C250	4.7261	7.6177	6.5775	C
251	C251	5.5121	8.3894	5.5006	C
252	C252	4.7464	9.1980	4.3998	C
253	H253	-0.3840	-0.2815	-1.0933	H

254	H254	-0.4144	-2.5448	2.0753	H
255	H255	-1.5134	-1.3841	0.4950	H
256	H256	-2.6198	-0.2555	2.0934	H
257	H257	-2.6043	1.9839	2.1165	H
258	H258	-1.5209	-1.4163	3.6738	H
259	H259	-0.3979	-0.3468	5.2894	H
260	H260	1.8238	-2.6134	2.0813	H
261	H261	2.6923	-0.3276	-3.2678	H
262	H262	2.6469	-2.6096	-0.0969	H
263	H263	1.5448	-1.4453	-1.6817	H
264	H264	4.9005	-2.6604	-0.0925	H
265	H265	5.7717	-0.3527	-5.4127	H
266	H266	8.0240	-0.3916	-5.4035	H
267	H267	9.1189	-1.5486	-3.8289	H
268	H268	5.7302	-2.6383	-2.2647	H
269	H269	4.6294	-1.4753	-3.8474	H
270	H270	7.9691	-2.6746	-2.2527	H
271	H271	2.6407	-2.6482	4.2646	H
272	H272	6.0208	-1.6047	5.8833	H
273	H273	1.5317	-1.5154	5.8652	H
274	H274	4.9195	-0.4755	7.4726	H
275	H275	2.6671	-0.4328	7.4636	H
276	H276	4.8794	-2.6930	4.2763	H
277	H277	5.7086	-2.6985	2.1038	H
278	H278	9.1053	-1.6396	3.7175	H
279	H279	7.9984	-0.5155	5.3276	H
280	H280	7.9626	-2.7292	2.1084	H
281	H281	11.0893	-0.4626	-3.2289	H
282	H282	12.1724	-1.6062	-1.6368	H
283	H283	8.7862	-2.7160	-0.0693	H
284	H284	13.3118	-0.5198	-0.0324	H
285	H285	13.3769	1.7186	-0.0076	H
286	H286	12.1647	-1.6418	1.5418	H
287	H287	11.0750	-0.5336	3.1538	H
288	H288	11.0254	-2.7282	-0.0625	H
289	H289	6.0849	1.4499	8.0809	H
290	H290	1.5731	1.5327	8.0643	H
291	H291	6.1665	5.9644	8.1288	H
292	H292	1.6543	6.0446	8.1121	H
293	H293	2.7611	4.8756	9.6690	H
294	H294	5.0084	4.8349	9.6770	H
295	H295	4.9681	2.5877	9.6532	H
296	H296	2.7205	2.6282	9.6452	H
297	H297	9.1759	1.4160	5.9277	H
298	H298	9.2576	5.9318	5.9759	H
299	H299	8.0997	4.8046	7.5476	H
300	H300	8.0590	2.5507	7.5235	H
301	H301	13.3853	2.5189	2.1788	H
302	H302	13.4258	4.7581	2.2030	H
303	H303	12.2374	1.3947	3.7578	H

304	H304	12.3186	5.8887	3.8062	H
305	H305	11.1807	4.7704	5.3774	H
306	H306	11.1400	2.5192	5.3532	H
307	H307	4.6767	1.6260	-5.9792	H
308	H308	5.8755	5.0032	-7.5028	H
309	H309	5.8349	2.7560	-7.5270	H
310	H310	8.0823	2.7152	-7.5188	H
311	H311	9.1887	1.5455	-5.9621	H
312	H312	8.1229	4.9625	-7.4946	H
313	H313	9.2702	6.0576	-5.9134	H
314	H314	4.7581	6.1404	-5.9304	H
315	H315	1.5853	1.6576	-3.8260	H
316	H316	2.7837	5.0390	-5.3733	H
317	H317	2.7432	2.7852	-5.3975	H
318	H318	1.6665	6.1733	-3.7774	H
319	H319	-1.4760	1.7001	-1.6553	H
320	H320	-2.5831	2.8307	-0.0521	H
321	H321	-2.5429	5.0699	-0.0283	H
322	H322	-0.2978	5.0694	-3.2027	H
323	H323	-0.3381	2.8183	-3.2268	H
324	H324	-1.3952	6.1940	-1.6074	H
325	H325	5.9238	8.0657	-5.3219	H
326	H326	5.9634	10.2828	-2.1255	H
327	H327	8.1761	8.0231	-5.3126	H
328	H328	8.2021	10.2380	-2.1133	H
329	H329	9.3113	9.1055	-3.7140	H
330	H330	4.8221	9.1946	-3.7327	H
331	H331	2.8443	8.1049	-3.1774	H
332	H332	2.8798	10.3184	0.0417	H
333	H333	5.1337	10.2880	0.0465	H
334	H334	1.7370	9.2289	-1.5673	H
335	H335	-2.5344	5.8708	2.1580	H
336	H336	-2.4692	8.1093	2.1830	H
337	H337	-0.2327	8.1227	-1.0035	H
338	H338	-1.3296	9.1957	3.7873	H
339	H339	-0.2465	8.0520	5.3793	H
340	H340	-0.1827	10.3175	2.2129	H
341	H341	2.0565	10.3053	2.2194	H
342	H342	-1.3221	9.2312	0.6086	H
343	H343	9.0194	10.2029	0.0697	H
344	H344	11.2410	7.9365	-3.1382	H
345	H345	11.2265	7.8704	3.2445	H
346	H346	13.4471	5.6052	0.0344	H
347	H347	13.4628	7.8446	0.0580	H
348	H348	12.3562	8.9730	1.6564	H
349	H349	11.2576	10.1341	0.0763	H
350	H350	12.3640	9.0058	-1.5223	H
351	H351	5.9422	10.2499	2.2425	H
352	H352	8.1503	7.9171	5.4181	H
353	H353	9.2979	9.0347	3.8321	H

354 H354 8.1958 10.1991 2.2473 H
355 H355 1.7236 9.1383 5.9788 H
356 H356 2.8189 7.9818 7.5534 H
357 H357 2.8734 10.2642 4.4024 H
358 H358 5.0712 7.9430 7.5625 H
359 H359 6.2132 9.0654 5.9972 H
360 H360 5.1123 10.2281 4.4142 H
361 C361 -0.7296 5.4123 5.4454 C
362 H362 -1.4106 6.1128 5.9360 H
363 C363 0.0236 4.6169 6.5283 C
364 H364 -0.3153 4.9561 7.5107 H
365 C365 -1.5234 4.6554 4.3277 C
366 H366 -2.5527 5.0238 4.3279 H
367 C367 -0.0046 3.0513 6.5116 C
368 H368 -0.3558 2.7037 7.4867 H
369 C369 -0.7855 2.3067 5.4123 C
370 H370 -1.4913 1.6209 5.8881 H
371 C371 -1.5512 3.1154 4.3113 C
372 H372 -2.5930 2.7843 4.3042 H
373 C373 12.3940 4.4741 -2.1606 C
374 H374 13.4359 4.8051 -2.1532 H
375 C375 11.5724 2.1772 -3.2950 C
376 H376 12.2533 1.4767 -3.7856 H
377 C377 11.6285 5.2829 -3.2615 C
378 H378 12.3345 5.9688 -3.7371 H
379 C379 10.8194 2.9729 -4.3779 C
380 H380 11.1583 2.6337 -5.3603 H
381 C381 10.8476 4.5385 -4.3610 C
382 H382 11.1990 4.8862 -5.3360 H
383 C383 12.3662 2.9340 -2.1772 C
384 H384 13.3954 2.5655 -2.1774 H
@<TRIPOS>BOND
1 1 2 1
2 1 3 1
3 1 7 1
4 1 253 1
5 2 4 1
6 2 5 1
7 2 29 1
8 3 4 1
9 3 11 1
10 3 159 1
11 4 10 1
12 4 144 1
13 5 8 1
14 5 24 1
15 5 28 1
16 6 7 1
17 6 15 1
18 6 24 1

19 6 254 1
20 7 12 1
21 7 255 1
22 8 9 1
23 8 14 1
24 8 35 1
25 9 10 1
26 9 18 1
27 9 34 1
28 10 17 1
29 10 150 1
30 11 13 1
31 11 16 1
32 11 160 1
33 12 13 1
34 12 15 1
35 12 256 1
36 13 19 1
37 13 257 1
38 14 22 1
39 14 24 1
40 14 60 1
41 15 23 1
42 15 258 1
43 16 17 1
44 16 19 1
45 16 165 1
46 17 18 1
47 17 167 1
48 18 21 1
49 18 49 1
50 19 20 1
51 19 371 1
52 20 21 1
53 20 23 1
54 20 369 1
55 21 22 1
56 21 50 1
57 22 23 1
58 22 64 1
59 23 259 1
60 24 260 1
61 25 26 1
62 25 29 1
63 25 143 1
64 25 261 1
65 26 27 1
66 26 41 1
67 26 151 1
68 27 30 1

69 27 36 1
70 27 40 1
71 28 29 1
72 28 36 1
73 28 262 1
74 29 263 1
75 30 31 1
76 30 32 1
77 30 47 1
78 31 33 1
79 31 46 1
80 31 153 1
81 32 35 1
82 32 36 1
83 32 72 1
84 33 34 1
85 33 53 1
86 33 154 1
87 34 35 1
88 34 54 1
89 35 59 1
90 36 264 1
91 37 38 1
92 37 41 1
93 37 119 1
94 37 265 1
95 38 39 1
96 38 127 1
97 38 266 1
98 39 42 1
99 39 48 1
100 39 267 1
101 40 41 1
102 40 48 1
103 40 268 1
104 41 269 1
105 42 43 1
106 42 44 1
107 42 80 1
108 43 45 1
109 43 81 1
110 43 132 1
111 44 47 1
112 44 48 1
113 44 83 1
114 45 46 1
115 45 56 1
116 45 133 1
117 46 47 1
118 46 57 1

119 47 71 1
120 48 270 1
121 49 50 1
122 49 55 1
123 49 183 1
124 50 52 1
125 50 66 1
126 51 52 1
127 51 98 1
128 51 243 1
129 51 363 1
130 52 96 1
131 52 367 1
132 53 54 1
133 53 58 1
134 53 177 1
135 54 55 1
136 54 62 1
137 55 65 1
138 55 185 1
139 56 57 1
140 56 88 1
141 56 172 1
142 57 58 1
143 57 74 1
144 58 76 1
145 58 179 1
146 59 61 1
147 59 70 1
148 59 72 1
149 60 64 1
150 60 70 1
151 60 271 1
152 61 62 1
153 61 63 1
154 61 78 1
155 62 65 1
156 62 77 1
157 63 68 1
158 63 70 1
159 63 272 1
160 64 69 1
161 64 273 1
162 65 67 1
163 65 93 1
164 66 67 1
165 66 69 1
166 66 96 1
167 67 68 1
168 67 95 1

169 68 69 1
170 68 274 1
171 69 275 1
172 70 276 1
173 71 73 1
174 71 79 1
175 71 83 1
176 72 79 1
177 72 277 1
178 73 74 1
179 73 75 1
180 73 91 1
181 74 76 1
182 74 90 1
183 75 78 1
184 75 79 1
185 75 278 1
186 76 77 1
187 76 103 1
188 77 78 1
189 77 105 1
190 78 279 1
191 79 280 1
192 80 81 1
193 80 82 1
194 80 281 1
195 81 86 1
196 81 375 1
197 82 84 1
198 82 92 1
199 82 282 1
200 83 92 1
201 83 283 1
202 84 85 1
203 84 87 1
204 84 284 1
205 85 86 1
206 85 89 1
207 85 285 1
208 86 88 1
209 86 383 1
210 87 91 1
211 87 92 1
212 87 286 1
213 88 89 1
214 88 109 1
215 89 90 1
216 89 110 1
217 90 91 1
218 90 113 1

219 91 287 1
220 92 288 1
221 93 94 1
222 93 95 1
223 93 108 1
224 94 97 1
225 94 107 1
226 94 248 1
227 95 101 1
228 95 289 1
229 96 102 1
230 96 290 1
231 97 100 1
232 97 249 1
233 97 291 1
234 98 99 1
235 98 246 1
236 98 292 1
237 99 100 1
238 99 102 1
239 99 293 1
240 100 101 1
241 100 294 1
242 101 102 1
243 101 295 1
244 102 296 1
245 103 104 1
246 103 105 1
247 103 116 1
248 104 106 1
249 104 115 1
250 104 238 1
251 105 108 1
252 105 297 1
253 106 107 1
254 106 239 1
255 106 298 1
256 107 108 1
257 107 299 1
258 108 300 1
259 109 112 1
260 109 172 1
261 109 174 1
262 110 111 1
263 110 113 1
264 110 301 1
265 111 112 1
266 111 114 1
267 111 302 1
268 112 227 1

269 112 229 1
270 113 116 1
271 113 303 1
272 114 115 1
273 114 227 1
274 114 304 1
275 115 116 1
276 115 305 1
277 116 306 1
278 117 118 1
279 117 119 1
280 117 123 1
281 117 307 1
282 118 120 1
283 118 121 1
284 118 147 1
285 119 120 1
286 119 127 1
287 120 126 1
288 120 151 1
289 121 124 1
290 121 140 1
291 121 146 1
292 122 123 1
293 122 131 1
294 122 140 1
295 122 308 1
296 123 128 1
297 123 309 1
298 124 125 1
299 124 130 1
300 124 157 1
301 125 126 1
302 125 134 1
303 125 156 1
304 126 133 1
305 126 153 1
306 127 129 1
307 127 132 1
308 128 129 1
309 128 131 1
310 128 310 1
311 129 135 1
312 129 311 1
313 130 138 1
314 130 140 1
315 130 189 1
316 131 139 1
317 131 312 1
318 132 133 1

319 132 135 1
320 133 134 1
321 134 137 1
322 134 171 1
323 135 136 1
324 135 379 1
325 136 137 1
326 136 139 1
327 136 381 1
328 137 138 1
329 137 173 1
330 138 139 1
331 138 193 1
332 139 313 1
333 140 314 1
334 141 142 1
335 141 143 1
336 141 147 1
337 141 315 1
338 142 144 1
339 142 145 1
340 142 163 1
341 143 144 1
342 143 151 1
343 144 150 1
344 145 148 1
345 145 158 1
346 145 162 1
347 146 147 1
348 146 158 1
349 146 316 1
350 147 317 1
351 148 149 1
352 148 152 1
353 148 169 1
354 149 150 1
355 149 155 1
356 149 168 1
357 150 154 1
358 151 153 1
359 152 157 1
360 152 158 1
361 152 201 1
362 153 154 1
363 154 155 1
364 155 156 1
365 155 176 1
366 156 157 1
367 156 178 1
368 157 188 1

369 158 318 1
370 159 160 1
371 159 163 1
372 159 319 1
373 160 161 1
374 160 320 1
375 161 164 1
376 161 170 1
377 161 321 1
378 162 163 1
379 162 170 1
380 162 322 1
381 163 323 1
382 164 165 1
383 164 166 1
384 164 209 1
385 165 167 1
386 165 210 1
387 166 169 1
388 166 170 1
389 166 212 1
390 167 168 1
391 167 182 1
392 168 169 1
393 168 184 1
394 169 200 1
395 170 324 1
396 171 172 1
397 171 173 1
398 171 181 1
399 172 180 1
400 173 175 1
401 173 195 1
402 174 175 1
403 174 229 1
404 174 373 1
405 175 226 1
406 175 377 1
407 176 177 1
408 176 178 1
409 176 187 1
410 177 179 1
411 177 186 1
412 178 181 1
413 178 191 1
414 179 180 1
415 179 238 1
416 180 181 1
417 180 224 1
418 181 194 1

419 182 183 1
420 182 184 1
421 182 217 1
422 183 185 1
423 183 243 1
424 184 187 1
425 184 203 1
426 185 186 1
427 185 248 1
428 186 187 1
429 186 236 1
430 187 205 1
431 188 190 1
432 188 199 1
433 188 201 1
434 189 193 1
435 189 199 1
436 189 325 1
437 190 191 1
438 190 192 1
439 190 207 1
440 191 194 1
441 191 206 1
442 192 197 1
443 192 199 1
444 192 326 1
445 193 198 1
446 193 327 1
447 194 196 1
448 194 222 1
449 195 196 1
450 195 198 1
451 195 226 1
452 196 197 1
453 196 225 1
454 197 198 1
455 197 328 1
456 198 329 1
457 199 330 1
458 200 202 1
459 200 208 1
460 200 212 1
461 201 208 1
462 201 331 1
463 202 203 1
464 202 204 1
465 202 220 1
466 203 205 1
467 203 219 1
468 204 207 1

469 204 208 1
470 204 332 1
471 205 206 1
472 205 234 1
473 206 207 1
474 206 237 1
475 207 333 1
476 208 334 1
477 209 210 1
478 209 211 1
479 209 335 1
480 210 215 1
481 210 365 1
482 211 213 1
483 211 221 1
484 211 336 1
485 212 221 1
486 212 337 1
487 213 214 1
488 213 216 1
489 213 338 1
490 214 215 1
491 214 218 1
492 214 339 1
493 215 217 1
494 215 361 1
495 216 220 1
496 216 221 1
497 216 340 1
498 217 218 1
499 217 243 1
500 218 219 1
501 218 244 1
502 219 220 1
503 219 247 1
504 220 341 1
505 221 342 1
506 222 223 1
507 222 225 1
508 222 242 1
509 223 224 1
510 223 228 1
511 223 241 1
512 224 227 1
513 224 238 1
514 225 232 1
515 225 343 1
516 226 233 1
517 226 344 1
518 227 228 1

519 228 231 1
520 228 345 1
521 229 230 1
522 229 346 1
523 230 231 1
524 230 233 1
525 230 347 1
526 231 232 1
527 231 348 1
528 232 233 1
529 232 349 1
530 233 350 1
531 234 235 1
532 234 237 1
533 234 252 1
534 235 236 1
535 235 240 1
536 235 251 1
537 236 239 1
538 236 248 1
539 237 242 1
540 237 351 1
541 238 239 1
542 239 240 1
543 240 241 1
544 240 352 1
545 241 242 1
546 241 353 1
547 242 354 1
548 243 246 1
549 244 245 1
550 244 247 1
551 244 355 1
552 245 246 1
553 245 250 1
554 245 356 1
555 246 249 1
556 247 252 1
557 247 357 1
558 248 249 1
559 249 250 1
560 250 251 1
561 250 358 1
562 251 252 1
563 251 359 1
564 252 360 1
565 361 362 1
566 361 363 1
567 361 365 1
568 363 364 1

569 363 367 1
570 365 366 1
571 365 371 1
572 367 368 1
573 367 369 1
574 369 370 1
575 369 371 1
576 371 372 1
577 373 374 1
578 373 377 1
579 373 383 1
580 375 376 1
581 375 379 1
582 375 383 1
583 377 378 1
584 377 381 1
585 379 380 1
586 379 381 1
587 381 382 1
588 383 384 1

Figure S23. A solid view of the 3D carbon allotrope formed by fusions of several space filling carbon voxel nugget$_{24a}$s containing 252 carbon atoms. Cartesian coordinates of its atoms; the first line contains the total charge and multiplicity; the following lines contain the atomic numbers, followed by the x, y, and z coordinates in Å for each one of the atoms. Next, atomic coordinates in Tripos Mol2 file format (.mol2) with the distances also in Å.

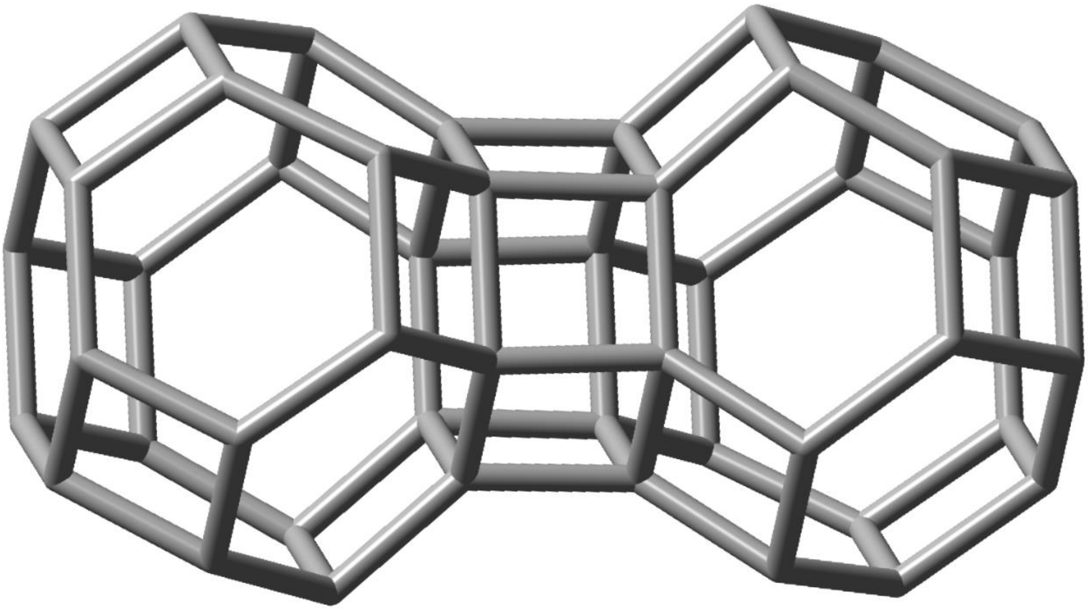

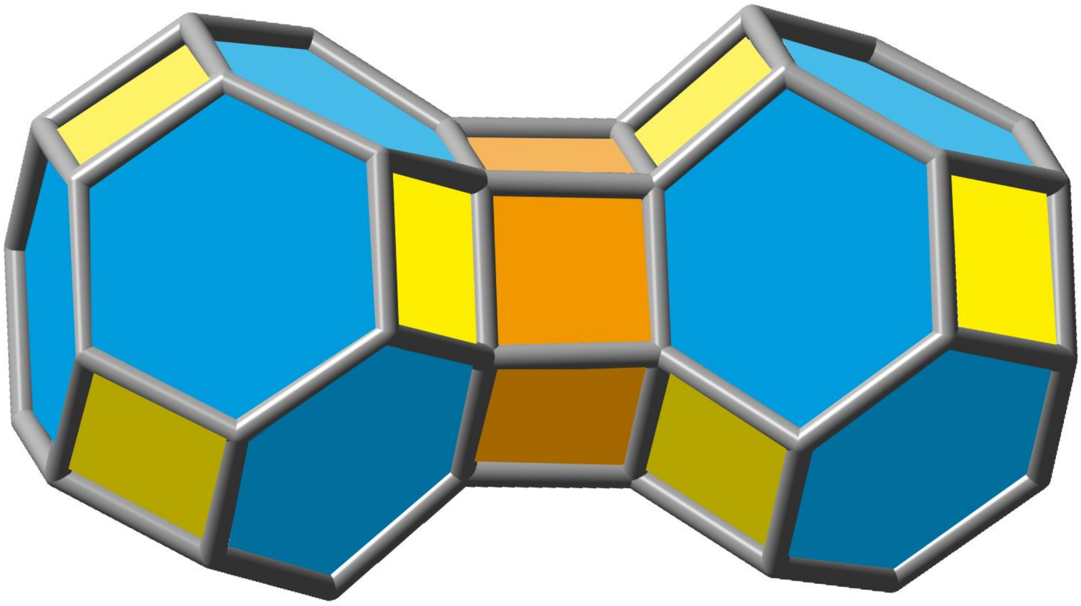

Cartesian Coordinates (Å)

0 1

6	8.922	-0.493	1.556
6	10.405	-0.794	1.734
6	8.653	-0.906	3.049
6	10.134	-1.209	3.237
1	10.588	-0.509	3.938
1	10.996	0.118	1.668
6	10.855	-1.862	0.701
1	11.781	-1.501	0.241

6	8.449	-1.932	-0.671
1	8.311	-1.445	-1.636
6	8.037	-1.008	0.468
6	11.087	-3.327	1.182
1	12.119	-3.604	0.941
6	10.824	-3.730	2.641
1	11.742	-4.181	3.032
6	10.319	-2.685	3.682
1	11.008	-2.687	4.533
6	7.513	-1.812	3.379
6	6.838	-1.962	0.816
6	6.582	-2.354	2.235
6	10.163	-3.864	0.046
1	10.792	-4.376	-0.690
6	7.241	-2.893	-0.321
1	6.486	-2.896	-1.106
6	7.614	-3.213	3.969
1	7.127	-3.261	4.942
6	9.101	-3.653	4.035
1	9.277	-4.064	5.035
6	9.634	-4.676	2.986
1	10.033	-5.540	3.527
6	6.676	-3.759	2.816
1	5.710	-4.085	3.201
6	7.242	-4.734	1.749
1	6.600	-5.621	1.743
6	8.723	-5.205	1.867
1	8.728	-6.299	1.924
6	8.990	-4.796	0.386
1	9.111	-5.712	-0.203
6	7.514	-4.316	0.234
1	6.988	-5.026	-0.411
6	9.911	-2.411	-0.460
1	10.438	-2.284	-1.412
6	5.861	3.594	2.140
6	7.342	3.123	2.258
6	5.594	3.185	3.621
6	7.069	2.704	3.773
1	7.596	3.415	4.418
1	5.855	4.687	2.083
1	5.472	4.100	4.210
1	7.984	4.010	2.264
6	7.908	2.147	1.191
1	8.873	2.474	0.807
6	5.483	2.041	-0.028
1	5.307	2.452	-1.028
6	4.950	3.064	1.021
1	4.551	3.929	0.480
6	8.002	0.742	1.773
6	7.746	0.350	3.191

6	7.343	1.281	4.328
1	8.098	1.284	5.113
6	4.420	2.252	3.961
1	3.791	2.764	4.697
6	3.760	2.118	1.366
1	2.842	2.569	0.975
6	3.497	1.715	2.825
1	2.465	1.992	3.066
6	7.071	0.200	0.628
6	4.265	1.073	0.325
1	3.575	1.075	-0.525
6	4.673	0.799	4.468
1	4.146	0.672	5.419
6	6.134	0.320	4.678
1	6.272	-0.167	5.643
6	6.546	-0.604	3.539
6	3.728	0.250	3.306
1	2.803	-0.110	3.766
6	4.179	-0.818	2.273
1	3.587	-1.730	2.340
6	5.662	-1.118	2.451
6	5.931	-0.706	0.959
6	4.450	-0.403	0.770
1	3.996	-1.103	0.070
6	6.970	1.602	0.038
1	7.457	1.650	-0.935

.mol2 file

```
@<TRIPOS>MOLECULE
Molecule Name
84 114
SMALL
NO_CHARGES


@<TRIPOS>ATOM
1 C1     8.9217    -0.4934     1.5559 C
2 C2    10.4050    -0.7940     1.7341 C
3 C3     8.6528    -0.9057     3.0486 C
4 C4    10.1342    -1.2092     3.2375 C
5 H5    10.5876    -0.5090     3.9375 H
6 H6    10.9965     0.1179     1.6675 H
7 C7    10.8554    -1.8616     0.7013 C
8 H8    11.7812    -1.5014     0.2409 H
9 C9     8.4494    -1.9320    -0.6712 C
10 H10     8.3114    -1.4450    -1.6355 H
11 C11     8.0374    -1.0081     0.4683 C
12 C12    11.0870    -3.3269     1.1817 C
13 H13    12.1188    -3.6036     0.9408 H
14 C14    10.8241    -3.7300     2.6413 C
```

15	H15	11.7421	-4.1812	3.0323	H
16	C16	10.3186	-2.6848	3.6822	C
17	H17	11.0083	-2.6866	4.5326	H
18	C18	7.5131	-1.8121	3.3793	C
19	C19	6.8377	-1.9622	0.8161	C
20	C20	6.5822	-2.3540	2.2345	C
21	C21	10.1633	-3.8640	0.0463	C
22	H22	10.7924	-4.3756	-0.6897	H
23	C23	7.2409	-2.8928	-0.3209	C
24	H24	6.4861	-2.8959	-1.1062	H
25	C25	7.6137	-3.2134	3.9689	C
26	H26	7.1268	-3.2614	4.9419	H
27	C27	9.1012	-3.6528	4.0355	C
28	H28	9.2768	-4.0639	5.0349	H
29	C29	9.6338	-4.6759	2.9864	C
30	H30	10.0328	-5.5403	3.5275	H
31	C31	6.6759	-3.7592	2.8160	C
32	H32	5.7105	-4.0853	3.2006	H
33	C33	7.2416	-4.7344	1.7492	C
34	H34	6.5998	-5.6213	1.7431	H
35	C35	8.7234	-5.2054	1.8672	C
36	H36	8.7284	-6.2989	1.9241	H
37	C37	8.9900	-4.7964	0.3864	C
38	H38	9.1114	-5.7116	-0.2025	H
39	C39	7.5144	-4.3161	0.2345	C
40	H40	6.9877	-5.0264	-0.4110	H
41	C41	9.9109	-2.4111	-0.4604	C
42	H42	10.4378	-2.2836	-1.4116	H
43	C43	5.8605	3.5936	2.1400	C
44	C44	7.3422	3.1226	2.2580	C
45	C45	5.5937	3.1847	3.6208	C
46	C46	7.0694	2.7043	3.7727	C
47	H47	7.5961	3.4146	4.4182	H
48	H48	5.8554	4.6871	2.0831	H
49	H49	5.4724	4.0998	4.2097	H
50	H50	7.9840	4.0095	2.2641	H
51	C51	7.9079	2.1474	1.1912	C
52	H52	8.8733	2.4736	0.8067	H
53	C53	5.4826	2.0410	-0.0283	C
54	H54	5.3070	2.4521	-1.0276	H
55	C55	4.9500	3.0641	1.0208	C
56	H56	4.5510	3.9285	0.4798	H
57	C57	8.0016	0.7422	1.7727	C
58	C58	7.7462	0.3504	3.1911	C
59	C59	7.3429	1.2810	4.3282	C
60	H60	8.0977	1.2841	5.1134	H
61	C61	4.4205	2.2522	3.9609	C
62	H62	3.7914	2.7638	4.6969	H
63	C63	3.7597	2.1182	1.3659	C
64	H64	2.8417	2.5694	0.9749	H

65 C65 3.4968 1.7151 2.8255 C
66 H66 2.4650 1.9918 3.0664 H
67 C67 7.0707 0.2003 0.6279 C
68 C68 4.2653 1.0731 0.3250 C
69 H69 3.5755 1.0749 -0.5253 H
70 C70 4.6729 0.7993 4.4676 C
71 H71 4.1460 0.6718 5.4188 H
72 C72 6.1344 0.3202 4.6784 C
73 H73 6.2724 -0.1669 5.6427 H
74 C74 6.5464 -0.6036 3.5389 C
75 C75 3.7284 0.2498 3.3059 C
76 H76 2.8026 -0.1104 3.7663 H
77 C77 4.1788 -0.8178 2.2731 C
78 H78 3.5873 -1.7297 2.3397 H
79 C79 5.6622 -1.1184 2.4513 C
80 C80 5.9310 -0.7061 0.9585 C
81 C81 4.4496 -0.4026 0.7697 C
82 H82 3.9962 -1.1028 0.0697 H
83 C83 6.9701 1.6017 0.0383 C
84 H84 7.4570 1.6497 -0.9347 H
@<TRIPOS>BOND
1 1 2 1
2 1 3 1
3 1 11 1
4 1 57 1
5 2 6 1
6 2 4 1
7 2 7 1
8 3 4 1
9 3 18 1
10 3 58 1
11 4 5 1
12 4 16 1
13 7 8 1
14 7 12 1
15 7 41 1
16 9 10 1
17 9 11 1
18 9 23 1
19 9 41 1
20 11 19 1
21 11 67 1
22 12 13 1
23 12 14 1
24 12 21 1
25 14 15 1
26 14 16 1
27 14 29 1
28 16 17 1
29 16 27 1

30 18 20 1
31 18 25 1
32 18 74 1
33 19 20 1
34 19 23 1
35 19 80 1
36 20 31 1
37 20 79 1
38 21 22 1
39 21 37 1
40 21 41 1
41 23 24 1
42 23 39 1
43 25 26 1
44 25 27 1
45 25 31 1
46 27 28 1
47 27 29 1
48 29 30 1
49 29 35 1
50 31 32 1
51 31 33 1
52 33 34 1
53 33 35 1
54 33 39 1
55 35 36 1
56 35 37 1
57 37 38 1
58 37 39 1
59 39 40 1
60 41 42 1
61 43 48 1
62 43 44 1
63 43 45 1
64 43 55 1
65 44 50 1
66 44 46 1
67 44 51 1
68 45 49 1
69 45 46 1
70 45 61 1
71 46 47 1
72 46 59 1
73 51 52 1
74 51 57 1
75 51 83 1
76 53 54 1
77 53 55 1
78 53 68 1
79 53 83 1

80 55 56 1
81 55 63 1
82 57 58 1
83 57 67 1
84 58 59 1
85 58 74 1
86 59 60 1
87 59 72 1
88 61 62 1
89 61 65 1
90 61 70 1
91 63 64 1
92 63 65 1
93 63 68 1
94 65 66 1
95 65 75 1
96 67 80 1
97 67 83 1
98 68 69 1
99 68 81 1
100 70 71 1
101 70 72 1
102 70 75 1
103 72 73 1
104 72 74 1
105 74 79 1
106 75 76 1
107 75 77 1
108 77 78 1
109 77 79 1
110 77 81 1
111 79 80 1
112 80 81 1
113 81 82 1
114 83 84 1

Figure S24. Compound $C_{48}H_{42}$ obtained by fusing together two nugget$_{24a}$ compounds via a hexagonal prism. Cartesian coordinates of its atoms; the first line contains the total charge and multiplicity; the following lines contain the atomic numbers, followed by the x, y, and z coordinates in Å for each one of the atoms. Next, atomic coordinates in Tripos Mol2 file format (.mol2) with the distances also in Å.

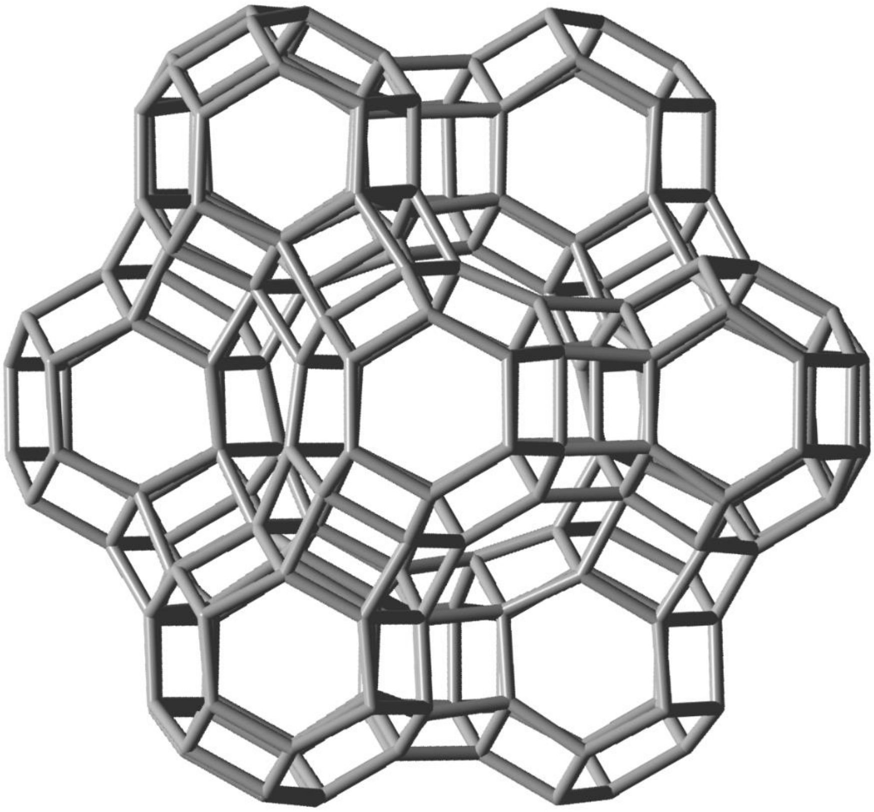

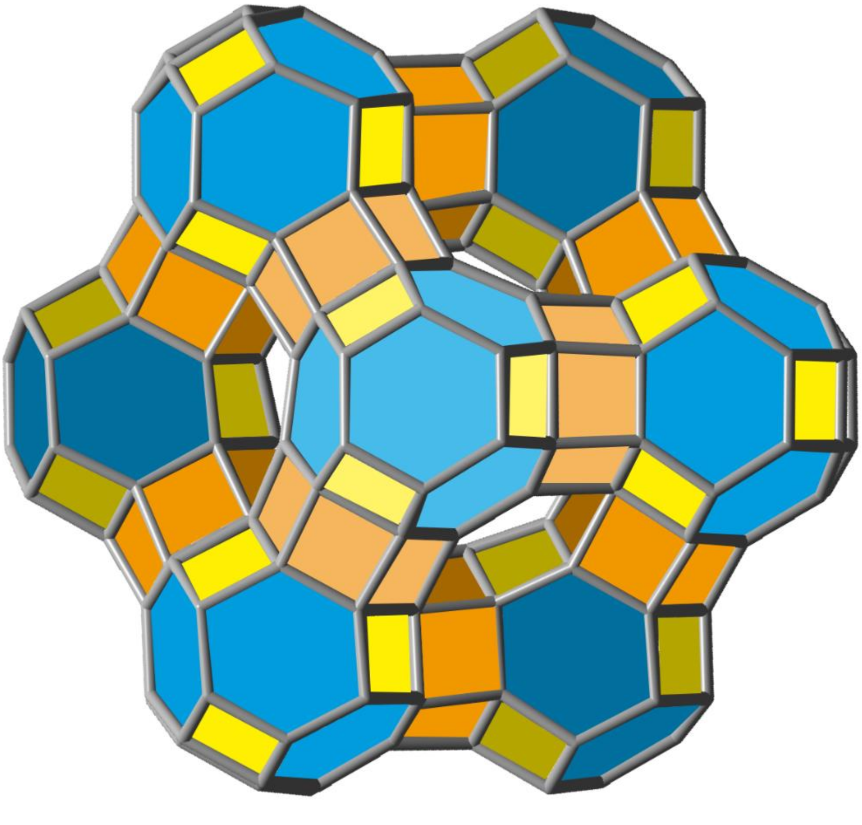

Cartesian Coordinates (Å)

0 1

6	24.522	-1.717	-0.278
6	25.615	-1.782	0.758
6	23.913	-0.780	0.857
6	25.036	-0.678	1.844
6	25.986	-3.215	1.081
6	23.954	-4.326	-0.189
6	23.633	-2.888	-0.576
6	25.868	-3.689	2.609
6	25.392	-2.672	3.625
6	24.923	-1.119	3.280
6	22.576	-0.953	1.557
6	22.164	-3.110	0.106
6	21.609	-2.155	1.112
6	25.087	-4.922	2.113
6	22.583	-4.546	0.427
6	22.461	-1.391	3.007
6	23.675	-1.711	3.862
6	24.151	-3.154	4.337
6	21.488	-2.594	2.551
6	21.927	-3.976	2.906
6	23.275	-4.244	3.791
6	23.718	-5.227	2.718
6	22.461	-5.000	1.896
6	25.203	-4.476	0.676
6	22.335	1.280	-3.912
6	23.604	1.029	-3.134
6	21.766	2.182	-2.846
6	23.148	1.922	-2.002
6	23.938	-0.384	-2.687
6	21.836	-1.296	-3.823
6	21.451	0.104	-4.209
6	23.752	-0.761	-1.222
6	23.170	0.110	-0.119
6	23.160	1.541	-0.552
6	20.412	1.976	-2.242
6	20.080	-0.110	-3.579
6	19.492	0.823	-2.581
6	22.892	-2.047	-1.685
6	20.464	-1.510	-3.194
6	20.352	1.460	-0.716
6	21.672	1.307	0.024
6	21.749	-0.059	0.621
6	19.402	0.351	-1.155
6	19.712	-1.081	-0.766
6	20.960	-1.210	0.073
6	21.548	-2.240	-1.057
6	20.253	-2.035	-1.804

6	23.110	-1.569	-3.106
6	27.076	-1.330	0.976
6	26.528	-0.391	1.992
6	26.409	-0.863	3.537
6	26.849	-2.233	3.911
6	27.366	-3.260	2.801
6	27.475	-2.821	1.372
6	29.837	1.728	4.570
6	30.790	2.882	4.939
6	29.944	2.197	3.037
6	30.892	3.320	3.497
6	30.370	3.878	6.023
6	28.206	2.358	6.351
6	28.455	1.494	5.137
6	29.976	5.292	5.632
6	30.078	5.736	4.153
6	30.577	4.761	3.101
6	28.656	2.374	2.259
6	27.046	1.747	4.318
6	27.142	2.185	2.881
6	28.570	5.113	6.213
6	26.926	2.702	5.462
6	28.523	3.772	1.707
6	29.322	4.920	2.249
6	28.768	5.952	3.389
6	27.122	3.618	2.448
6	26.563	4.513	3.538
6	27.411	5.798	3.995
6	27.319	5.382	5.444
6	26.492	4.080	4.990
6	29.011	3.589	6.653
6	29.838	-3.199	3.021
6	30.690	-2.039	3.441
6	29.949	-2.750	1.561
6	30.799	-1.590	1.980
6	30.284	-1.143	4.567
6	28.049	-2.559	4.783
6	28.547	-3.509	3.711
6	29.939	0.244	4.067
6	30.042	0.682	2.638
6	30.511	-0.201	1.503
6	28.778	-2.566	0.648
6	28.499	0.005	4.717
6	28.391	-1.160	0.238
6	29.267	0.036	0.669
6	28.713	0.904	1.782
6	27.369	0.754	2.402
6	27.256	0.281	3.947
6	28.931	-1.361	5.215
6	22.912	7.013	-0.413

6	21.689	7.232	-1.295
6	23.455	5.971	-1.545
6	22.197	6.279	-2.362
6	20.358	6.949	-0.606
6	21.587	6.196	1.711
6	22.849	6.688	1.050
6	19.514	5.772	-1.030
6	19.969	4.859	-2.157
6	21.327	5.079	-2.797
6	23.907	4.591	-1.191
6	23.272	5.183	1.361
6	23.745	4.156	0.350
6	19.481	5.221	0.406
6	22.119	4.683	2.180
6	23.055	3.420	-1.616
6	21.796	3.686	-2.373
6	20.327	3.451	-1.702
6	22.885	2.978	-0.078
6	21.489	2.743	0.477
6	20.116	2.934	-0.307
6	19.918	3.847	0.874
6	21.278	3.505	1.756
6	20.368	6.470	0.856
6	27.786	8.558	0.458
6	26.542	8.777	-0.354
6	28.296	7.590	-0.603
6	27.051	7.809	-1.415
6	25.194	8.666	0.265
6	26.370	8.067	2.555
6	27.731	8.242	1.924
6	24.408	7.478	-0.248
6	24.911	6.513	-1.276
6	26.248	6.666	-1.960
6	28.771	6.216	-0.286
6	28.147	6.823	2.229
6	28.664	5.882	1.187
6	24.353	6.970	1.284
6	26.672	6.633	2.920
6	27.900	5.066	-0.714
6	26.682	5.265	-1.599
6	25.375	4.987	-0.891
6	27.701	4.663	0.743
6	26.376	4.512	1.477
6	25.255	4.420	0.491
6	24.738	5.523	1.553
6	25.773	5.468	2.630
6	25.124	8.218	1.700
6	19.291	-2.656	4.434
6	18.860	-1.679	5.532
6	18.748	-2.111	6.972

6	19.224	-3.480	7.316
6	19.706	-4.429	6.255
6	19.715	-4.059	4.803
6	22.385	0.351	8.207
6	23.207	1.603	8.412
6	22.429	0.846	6.652
6	23.336	2.026	6.836
6	22.651	2.523	9.468
6	20.405	1.185	9.808
6	21.001	0.225	8.772
6	22.217	4.000	9.023
6	22.401	4.396	7.572
6	22.951	3.401	6.386
6	21.170	1.114	5.869
6	19.654	0.490	7.891
6	19.721	0.873	6.451
6	20.882	3.780	9.754
6	19.143	1.411	8.994
6	20.821	2.514	5.414
6	21.580	3.747	5.887
6	21.096	4.834	6.957
6	19.349	2.272	6.033
6	18.868	3.212	7.094
6	19.763	4.498	7.559
6	19.580	4.022	8.996
6	18.731	2.838	8.575
6	21.288	2.383	10.167
6	22.339	-4.577	6.510
6	23.176	-3.411	6.956
6	22.346	-4.148	5.059
6	23.211	-2.873	5.532
6	22.735	-2.495	8.074
6	20.483	-3.742	8.133
6	20.965	-4.690	7.073
6	22.420	-1.090	7.619
6	22.601	-0.602	6.217
6	22.755	-1.504	5.048
6	21.113	-3.879	4.256
6	20.924	-1.276	8.276
6	20.651	-2.355	3.837
6	21.467	-1.140	4.153
6	21.222	-0.339	5.399
6	19.826	-0.574	5.936
6	19.679	-1.029	7.479
6	21.365	-2.661	8.683
6	20.244	7.438	3.963
6	19.003	7.620	3.119
6	20.767	6.378	3.011
6	19.454	6.591	2.119
6	17.669	7.274	3.711

6	18.727	6.742	6.025
6	20.083	6.966	5.402
6	16.824	6.074	3.279
6	17.239	5.109	2.220
6	18.617	5.429	1.690
6	21.259	5.025	3.402
6	20.469	5.535	5.728
6	20.922	4.492	4.742
6	16.688	5.649	4.712
6	19.023	5.310	6.425
6	20.312	3.765	2.915
6	19.047	3.970	2.141
6	17.626	3.707	2.631
6	20.094	3.215	4.278
6	18.736	3.084	4.866
6	17.447	3.260	4.097
6	16.952	4.239	5.142
6	18.200	4.130	5.995
6	17.532	6.847	5.142
6	25.842	1.823	9.610
6	24.665	2.062	8.684
6	25.287	2.801	10.617
6	24.079	3.097	9.769
6	24.770	2.393	7.232
6	27.237	2.592	7.535
6	27.198	2.110	8.975
6	24.305	3.947	6.832
6	23.822	4.928	7.856
6	23.665	4.480	9.374
6	26.078	4.037	10.924
6	28.044	3.288	9.389
6	27.434	4.274	10.327
6	25.589	4.349	6.191
6	28.180	3.834	7.979
6	25.656	5.447	10.511
6	24.408	5.742	9.746
6	24.539	6.196	8.311
6	27.014	5.683	9.917
6	27.187	6.240	8.539
6	25.936	6.483	7.720
6	26.411	5.481	6.696
6	27.768	5.220	7.576
6	26.043	2.910	6.672
1	24.257	-5.043	-0.923
1	25.431	-5.903	2.369
1	22.006	-5.374	0.072
1	23.946	-6.245	2.955
1	21.873	-5.855	1.636
1	25.624	-5.157	-0.034
1	22.458	1.624	-4.918

1	24.521	1.201	-3.658
1	24.913	-0.270	-3.112
1	22.180	-1.995	-4.557
1	21.515	0.441	-5.223
1	19.207	0.079	-4.168
1	18.560	1.001	-3.075
1	19.877	-2.353	-3.493
1	18.433	0.245	-0.715
1	18.739	-1.307	-0.381
1	19.625	-2.864	-2.056
1	23.555	-2.217	-3.832
1	31.694	2.672	5.471
1	31.866	3.403	3.063
1	31.271	3.784	6.593
1	30.601	6.093	5.967
1	30.703	6.568	4.402
1	31.547	4.964	2.697
1	30.100	-4.206	3.271
1	31.589	-2.177	4.003
1	30.285	-3.458	0.832
1	31.772	-1.426	1.565
1	31.120	-1.313	5.213
1	27.943	-2.921	5.784
1	28.790	-4.490	4.061
1	31.433	-0.016	0.992
1	29.107	-3.192	-0.155
1	28.434	-0.894	-0.798
1	29.379	0.393	-0.333
1	28.892	-1.632	6.249
1	21.418	8.212	-1.631
1	22.277	6.646	-3.364
1	20.011	7.933	-0.843
1	18.598	5.968	-1.547
1	19.123	5.041	-2.786
1	21.353	5.372	-3.826
1	28.406	9.376	0.761
1	26.246	9.761	-0.653
1	29.265	7.752	-1.026
1	27.100	8.129	-2.435
1	24.856	9.663	0.068
1	26.092	8.796	3.287
1	28.310	9.071	2.273
1	26.309	6.927	-2.996
1	29.730	6.277	-0.758
1	28.287	4.375	-1.433
1	27.029	4.656	-2.407
1	24.728	8.897	2.426
1	18.391	-2.563	3.864
1	17.960	-1.466	4.993
1	17.752	-2.193	7.356

1	18.269	-3.613	7.780
1	19.106	-5.255	5.936
1	19.087	-4.848	4.445
1	20.266	0.868	10.821
1	20.573	4.461	10.519
1	18.203	1.231	9.473
1	19.155	4.665	9.738
1	17.762	2.732	9.017
1	21.243	2.132	11.206
1	22.694	-5.546	6.791
1	24.079	-3.612	7.494
1	23.564	-2.710	8.715
1	20.388	-4.047	9.155
1	21.223	-5.701	7.312
1	21.337	-2.964	9.709
1	20.815	8.312	4.198
1	18.777	8.627	2.839
1	17.382	8.252	3.386
1	18.437	7.461	6.762
1	20.659	7.801	5.742
1	15.962	6.243	2.667
1	16.368	5.178	1.602
1	15.729	5.501	5.163
1	17.055	2.879	2.265
1	16.805	2.440	3.848
1	15.989	4.018	5.553
1	17.137	7.508	5.885
1	26.092	0.844	9.963
1	25.198	2.437	11.619
1	27.525	1.130	9.253
1	25.960	3.739	11.945
1	28.918	3.051	9.959
1	28.326	4.153	10.906
1	25.254	6.106	11.252
1	23.986	6.462	10.416
1	24.182	7.166	8.034
1	27.618	6.513	10.219
1	27.743	7.119	8.791
1	25.700	7.497	7.474

.mol2 file

@<TRIPOS>MOLECULE
Molecule Name
336 528
SMALL
NO_CHARGES

@<TRIPOS>ATOM
1 C1 24.5220 -1.7169 -0.2780 C

2	C2	25.6150	-1.7819	0.7582	C
3	C3	23.9128	-0.7799	0.8569	C
4	C4	25.0356	-0.6782	1.8436	C
5	C5	25.9859	-3.2149	1.0814	C
6	C6	23.9544	-4.3259	-0.1890	C
7	C7	23.6326	-2.8876	-0.5764	C
8	C8	25.8684	-3.6891	2.6090	C
9	C9	25.3919	-2.6722	3.6250	C
10	C10	24.9231	-1.1194	3.2800	C
11	C11	22.5762	-0.9525	1.5569	C
12	C12	22.1641	-3.1098	0.1055	C
13	C13	21.6093	-2.1545	1.1124	C
14	C14	25.0875	-4.9222	2.1128	C
15	C15	22.5832	-4.5457	0.4266	C
16	C16	22.4611	-1.3914	3.0065	C
17	C17	23.6751	-1.7114	3.8621	C
18	C18	24.1514	-3.1539	4.3370	C
19	C19	21.4876	-2.5940	2.5513	C
20	C20	21.9274	-3.9760	2.9063	C
21	C21	23.2750	-4.2436	3.7908	C
22	C22	23.7181	-5.2271	2.7177	C
23	C23	22.4612	-5.0001	1.8963	C
24	C24	25.2025	-4.4763	0.6758	C
25	C25	22.3347	1.2795	-3.9122	C
26	C26	23.6039	1.0285	-3.1336	C
27	C27	21.7658	2.1821	-2.8458	C
28	C28	23.1480	1.9216	-2.0022	C
29	C29	23.9379	-0.3840	-2.6868	C
30	C30	21.8360	-1.2960	-3.8232	C
31	C31	21.4511	0.1039	-4.2092	C
32	C32	23.7524	-0.7608	-1.2217	C
33	C33	23.1699	0.1102	-0.1193	C
34	C34	23.1600	1.5406	-0.5524	C
35	C35	20.4124	1.9756	-2.2416	C
36	C36	20.0799	-0.1099	-3.5786	C
37	C37	19.4920	0.8232	-2.5808	C
38	C38	22.8923	-2.0474	-1.6854	C
39	C39	20.4642	-1.5100	-3.1936	C
40	C40	20.3515	1.4605	-0.7155	C
41	C41	21.6718	1.3071	0.0243	C
42	C42	21.7489	-0.0588	0.6209	C
43	C43	19.4024	0.3507	-1.1550	C
44	C44	19.7116	-1.0812	-0.7657	C
45	C45	20.9602	-1.2097	0.0726	C
46	C46	21.5481	-2.2405	-1.0565	C
47	C47	20.2532	-2.0348	-1.8040	C
48	C48	23.1101	-1.5693	-3.1058	C
49	C49	27.0762	-1.3298	0.9757	C
50	C50	26.5283	-0.3910	1.9915	C
51	C51	26.4086	-0.8629	3.5370	C

52	C52	26.8489	-2.2333	3.9113	C
53	C53	27.3656	-3.2596	2.8008	C
54	C54	27.4751	-2.8214	1.3724	C
55	C55	29.8374	1.7279	4.5697	C
56	C56	30.7899	2.8822	4.9386	C
57	C57	29.9439	2.1970	3.0369	C
58	C58	30.8920	3.3202	3.4969	C
59	C59	30.3699	3.8777	6.0232	C
60	C60	28.2064	2.3577	6.3506	C
61	C61	28.4552	1.4936	5.1371	C
62	C62	29.9763	5.2916	5.6316	C
63	C63	30.0781	5.7360	4.1531	C
64	C64	30.5770	4.7611	3.1008	C
65	C65	28.6558	2.3740	2.2589	C
66	C66	27.0455	1.7474	4.3180	C
67	C67	27.1421	2.1846	2.8814	C
68	C68	28.5699	5.1134	6.2134	C
69	C69	26.9260	2.7024	5.4616	C
70	C70	28.5230	3.7722	1.7070	C
71	C71	29.3215	4.9200	2.2494	C
72	C72	28.7678	5.9521	3.3888	C
73	C73	27.1219	3.6175	2.4476	C
74	C74	26.5628	4.5131	3.5383	C
75	C75	27.4107	5.7985	3.9950	C
76	C76	27.3191	5.3816	5.4437	C
77	C77	26.4923	4.0797	4.9900	C
78	C78	29.0108	3.5894	6.6532	C
79	C79	29.8384	-3.1987	3.0213	C
80	C80	30.6897	-2.0387	3.4407	C
81	C81	29.9486	-2.7503	1.5607	C
82	C82	30.7989	-1.5897	1.9802	C
83	C83	30.2842	-1.1425	4.5672	C
84	C84	28.0495	-2.5593	4.7827	C
85	C85	28.5470	-3.5088	3.7110	C
86	C86	29.9395	0.2437	4.0669	C
87	C87	30.0421	0.6822	2.6379	C
88	C88	30.5107	-0.2010	1.5027	C
89	C89	28.7780	-2.5664	0.6485	C
90	C90	28.4992	0.0054	4.7172	C
91	C91	28.3907	-1.1598	0.2380	C
92	C92	29.2670	0.0360	0.6694	C
93	C93	28.7129	0.9045	1.7820	C
94	C94	27.3691	0.7538	2.4025	C
95	C95	27.2560	0.2815	3.9474	C
96	C96	28.9308	-1.3606	5.2149	C
97	C97	22.9122	7.0127	-0.4132	C
98	C98	21.6889	7.2324	-1.2954	C
99	C99	23.4552	5.9709	-1.5453	C
100	C100	22.1969	6.2789	-2.3621	C
101	C101	20.3580	6.9487	-0.6060	C

102	C102	21.5868	6.1956	1.7106	C
103	C103	22.8494	6.6882	1.0498	C
104	C104	19.5137	5.7721	-1.0297	C
105	C105	19.9687	4.8589	-2.1567	C
106	C106	21.3269	5.0793	-2.7974	C
107	C107	23.9068	4.5915	-1.1907	C
108	C108	23.2718	5.1832	1.3608	C
109	C109	23.7453	4.1559	0.3502	C
110	C110	19.4806	5.2214	0.4060	C
111	C111	22.1186	4.6832	2.1802	C
112	C112	23.0553	3.4196	-1.6160	C
113	C113	21.7957	3.6855	-2.3732	C
114	C114	20.3271	3.4513	-1.7018	C
115	C115	22.8852	2.9779	-0.0780	C
116	C116	21.4893	2.7426	0.4770	C
117	C117	20.1164	2.9341	-0.3068	C
118	C118	19.9175	3.8467	0.8739	C
119	C119	21.2779	3.5049	1.7562	C
120	C120	20.3681	6.4697	0.8560	C
121	C121	27.7864	8.5584	0.4579	C
122	C122	26.5416	8.7775	-0.3540	C
123	C123	28.2958	7.5895	-0.6028	C
124	C124	27.0514	7.8094	-1.4151	C
125	C125	25.1936	8.6664	0.2649	C
126	C126	26.3703	8.0672	2.5545	C
127	C127	27.7314	8.2420	1.9235	C
128	C128	24.4084	7.4777	-0.2478	C
129	C129	24.9112	6.5132	-1.2763	C
130	C130	26.2477	6.6656	-1.9598	C
131	C131	28.7713	6.2160	-0.2865	C
132	C132	28.1470	6.8233	2.2295	C
133	C133	28.6642	5.8822	1.1870	C
134	C134	24.3532	6.9698	1.2839	C
135	C135	26.6719	6.6332	2.9200	C
136	C136	27.8997	5.0657	-0.7138	C
137	C137	26.6816	5.2650	-1.5990	C
138	C138	25.3751	4.9874	-0.8913	C
139	C139	27.7006	4.6626	0.7428	C
140	C140	26.3758	4.5123	1.4771	C
141	C141	25.2551	4.4205	0.4913	C
142	C142	24.7377	5.5225	1.5529	C
143	C143	25.7729	5.4685	2.6298	C
144	C144	25.1237	8.2184	1.7000	C
145	C145	19.2914	-2.6555	4.4337	C
146	C146	18.8601	-1.6789	5.5317	C
147	C147	18.7477	-2.1115	6.9722	C
148	C148	19.2238	-3.4796	7.3164	C
149	C149	19.7055	-4.4286	6.2555	C
150	C150	19.7154	-4.0586	4.8029	C
151	C151	22.3852	0.3511	8.2065	C

152	C152	23.2073	1.6035	8.4121	C
153	C153	22.4291	0.8464	6.6517	C
154	C154	23.3360	2.0257	6.8364	C
155	C155	22.6513	2.5229	9.4684	C
156	C156	20.4052	1.1846	9.8080	C
157	C157	21.0010	0.2251	8.7721	C
158	C158	22.2173	4.0003	9.0229	C
159	C159	22.4007	4.3962	7.5718	C
160	C160	22.9511	3.4006	6.3862	C
161	C161	21.1704	1.1144	5.8691	C
162	C162	19.6544	0.4899	7.8907	C
163	C163	19.7209	0.8733	6.4514	C
164	C164	20.8822	3.7800	9.7542	C
165	C165	19.1432	1.4107	8.9939	C
166	C166	20.8210	2.5140	5.4143	C
167	C167	21.5796	3.7470	5.8870	C
168	C168	21.0957	4.8344	6.9575	C
169	C169	19.3485	2.2720	6.0334	C
170	C170	18.8676	3.2117	7.0944	C
171	C171	19.7634	4.4976	7.5590	C
172	C172	19.5797	4.0219	8.9965	C
173	C173	18.7312	2.8378	8.5755	C
174	C174	21.2880	2.3830	10.1666	C
175	C175	22.3385	-4.5769	6.5104	C
176	C176	23.1761	-3.4108	6.9562	C
177	C177	22.3465	-4.1482	5.0592	C
178	C178	23.2111	-2.8728	5.5319	C
179	C179	22.7351	-2.4949	8.0741	C
180	C180	20.4835	-3.7419	8.1335	C
181	C181	20.9650	-4.6902	7.0729	C
182	C182	22.4197	-1.0899	7.6186	C
183	C183	22.6012	-0.6017	6.2169	C
184	C184	22.7552	-1.5039	5.0477	C
185	C185	21.1126	-3.8786	4.2559	C
186	C186	20.9235	-1.2757	8.2761	C
187	C187	20.6511	-2.3554	3.8371	C
188	C188	21.4667	-1.1397	4.1534	C
189	C189	21.2219	-0.3387	5.3986	C
190	C190	19.8262	-0.5736	5.9362	C
191	C191	19.6786	-1.0288	7.4789	C
192	C192	21.3650	-2.6610	8.6830	C
193	C193	20.2436	7.4383	3.9626	C
194	C194	19.0027	7.6197	3.1192	C
195	C195	20.7668	6.3777	3.0115	C
196	C196	19.4545	6.5908	2.1193	C
197	C197	17.6687	7.2738	3.7105	C
198	C198	18.7266	6.7417	6.0254	C
199	C199	20.0832	6.9661	5.4023	C
200	C200	16.8236	6.0741	3.2794	C
201	C201	17.2394	5.1095	2.2200	C

202	C202	18.6166	5.4292	1.6898	C
203	C203	21.2587	5.0249	3.4025	C
204	C204	20.4690	5.5348	5.7281	C
205	C205	20.9222	4.4923	4.7424	C
206	C206	16.6881	5.6491	4.7118	C
207	C207	19.0228	5.3098	6.4245	C
208	C208	20.3124	3.7649	2.9146	C
209	C209	19.0469	3.9696	2.1410	C
210	C210	17.6261	3.7065	2.6305	C
211	C211	20.0939	3.2153	4.2779	C
212	C212	18.7359	3.0838	4.8661	C
213	C213	17.4470	3.2595	4.0969	C
214	C214	16.9522	4.2392	5.1424	C
215	C215	18.2001	4.1302	5.9952	C
216	C216	17.5316	6.8474	5.1418	C
217	C217	25.8419	1.8225	9.6095	C
218	C218	24.6653	2.0617	8.6844	C
219	C219	25.2874	2.8015	10.6172	C
220	C220	24.0789	3.0973	9.7689	C
221	C221	24.7705	2.3931	7.2322	C
222	C222	27.2374	2.5916	7.5353	C
223	C223	27.1984	2.1099	8.9754	C
224	C224	24.3055	3.9469	6.8316	C
225	C225	23.8215	4.9283	7.8562	C
226	C226	23.6647	4.4800	9.3743	C
227	C227	26.0775	4.0373	10.9239	C
228	C228	28.0438	3.2884	9.3888	C
229	C229	27.4342	4.2739	10.3271	C
230	C230	25.5891	4.3490	6.1915	C
231	C231	28.1804	3.8345	7.9786	C
232	C232	25.6556	5.4466	10.5107	C
233	C233	24.4084	5.7424	9.7458	C
234	C234	24.5391	6.1961	8.3106	C
235	C235	27.0143	5.6827	9.9172	C
236	C236	27.1873	6.2402	8.5389	C
237	C237	25.9361	6.4826	7.7199	C
238	C238	26.4109	5.4805	6.6956	C
239	C239	27.7676	5.2199	7.5756	C
240	C240	26.0433	2.9101	6.6723	C
241	H241	24.2571	-5.0427	-0.9235	H
242	H242	25.4307	-5.9026	2.3694	H
243	H243	22.0059	-5.3741	0.0724	H
244	H244	23.9458	-6.2454	2.9546	H
245	H245	21.8730	-5.8553	1.6365	H
246	H246	25.6238	-5.1571	-0.0340	H
247	H247	22.4579	1.6243	-4.9176	H
248	H248	24.5208	1.2006	-3.6576	H
249	H249	24.9131	-0.2696	-3.1120	H
250	H250	22.1803	-1.9946	-4.5569	H
251	H251	21.5146	0.4406	-5.2228	H

252	H252	19.2072	0.0789	-4.1682	H
253	H253	18.5599	1.0009	-3.0753	H
254	H254	19.8771	-2.3529	-3.4932	H
255	H255	18.4331	0.2445	-0.7146	H
256	H256	18.7392	-1.3071	-0.3805	H
257	H257	19.6253	-2.8638	-2.0557	H
258	H258	23.5554	-2.2174	-3.8315	H
259	H259	31.6941	2.6724	5.4709	H
260	H260	31.8663	3.4032	3.0625	H
261	H261	31.2710	3.7835	6.5925	H
262	H262	30.6013	6.0927	5.9670	H
263	H263	30.7034	6.5678	4.4023	H
264	H264	31.5468	4.9636	2.6966	H
265	H265	30.0997	-4.2059	3.2706	H
266	H266	31.5894	-2.1774	4.0031	H
267	H267	30.2847	-3.4576	0.8315	H
268	H268	31.7716	-1.4264	1.5654	H
269	H269	31.1199	-1.3125	5.2134	H
270	H270	27.9429	-2.9211	5.7840	H
271	H271	28.7895	-4.4905	4.0608	H
272	H272	31.4325	-0.0164	0.9918	H
273	H273	29.1065	-3.1918	-0.1551	H
274	H274	28.4341	-0.8944	-0.7976	H
275	H275	29.3792	0.3934	-0.3329	H
276	H276	28.8916	-1.6315	6.2493	H
277	H277	21.4183	8.2118	-1.6308	H
278	H278	22.2770	6.6462	-3.3639	H
279	H279	20.0107	7.9327	-0.8425	H
280	H280	18.5978	5.9679	-1.5470	H
281	H281	19.1227	5.0410	-2.7860	H
282	H282	21.3534	5.3721	-3.8262	H
283	H283	28.4063	9.3762	0.7608	H
284	H284	26.2463	9.7614	-0.6534	H
285	H285	29.2651	7.7520	-1.0258	H
286	H286	27.1002	8.1288	-2.4352	H
287	H287	24.8564	9.6627	0.0684	H
288	H288	26.0923	8.7958	3.2872	H
289	H289	28.3099	9.0713	2.2734	H
290	H290	26.3093	6.9269	-2.9956	H
291	H291	29.7301	6.2766	-0.7576	H
292	H292	28.2869	4.3745	-1.4330	H
293	H293	27.0295	4.6556	-2.4067	H
294	H294	24.7278	8.8973	2.4261	H
295	H295	18.3907	-2.5626	3.8636	H
296	H296	17.9603	-1.4665	4.9931	H
297	H297	17.7524	-2.1930	7.3565	H
298	H298	18.2685	-3.6127	7.7796	H
299	H299	19.1061	-5.2553	5.9358	H
300	H300	19.0872	-4.8475	4.4453	H
301	H301	20.2660	0.8682	10.8206	H

```
302 H302    20.5726     4.4609    10.5194 H
303 H303    18.2035     1.2308     9.4730 H
304 H304    19.1549     4.6653     9.7384 H
305 H305    17.7622     2.7320     9.0168 H
306 H306    21.2432     2.1320    11.2058 H
307 H307    22.6939    -5.5463     6.7911 H
308 H308    24.0791    -3.6117     7.4939 H
309 H309    23.5644    -2.7100     8.7151 H
310 H310    20.3877    -4.0471     9.1546 H
311 H311    21.2228    -5.7008     7.3121 H
312 H312    21.3375    -2.9636     9.7090 H
313 H313    20.8151     8.3116     4.1983 H
314 H314    18.7769     8.6274     2.8391 H
315 H315    17.3820     8.2522     3.3857 H
316 H316    18.4368     7.4614     6.7623 H
317 H317    20.6593     7.8015     5.7416 H
318 H318    15.9624     6.2432     2.6674 H
319 H319    16.3683     5.1776     1.6023 H
320 H320    15.7294     5.5010     5.1633 H
321 H321    17.0548     2.8788     2.2652 H
322 H322    16.8049     2.4404     3.8484 H
323 H323    15.9889     4.0184     5.5525 H
324 H324    17.1371     7.5081     5.8853 H
325 H325    26.0916     0.8439     9.9628 H
326 H326    25.1977     2.4372    11.6193 H
327 H327    27.5252     1.1297     9.2533 H
328 H328    25.9599     3.7387    11.9447 H
329 H329    28.9177     3.0508     9.9587 H
330 H330    28.3258     4.1529    10.9062 H
331 H331    25.2538     6.1057    11.2517 H
332 H332    23.9864     6.4617    10.4162 H
333 H333    24.1818     7.1661     8.0342 H
334 H334    27.6177     6.5133    10.2187 H
335 H335    27.7431     7.1190     8.7915 H
336 H336    25.6996     7.4968     7.4740 H
@<TRIPOS>BOND
1 1 2 1
2 1 3 1
3 1 7 1
4 1 32 1
5 2 4 1
6 2 5 1
7 2 49 1
8 3 4 1
9 3 11 1
10 3 33 1
11 4 10 1
12 4 50 1
13 5 8 1
14 5 24 1
```

15 5 54 1
16 6 7 1
17 6 15 1
18 6 24 1
19 6 241 1
20 7 12 1
21 7 38 1
22 8 9 1
23 8 14 1
24 8 53 1
25 9 10 1
26 9 18 1
27 9 52 1
28 10 17 1
29 10 51 1
30 11 13 1
31 11 16 1
32 11 42 1
33 12 13 1
34 12 15 1
35 12 46 1
36 13 19 1
37 13 45 1
38 14 22 1
39 14 24 1
40 14 242 1
41 15 23 1
42 15 243 1
43 16 17 1
44 16 19 1
45 16 188 1
46 17 18 1
47 17 184 1
48 18 21 1
49 18 178 1
50 19 20 1
51 19 187 1
52 20 21 1
53 20 23 1
54 20 185 1
55 21 22 1
56 21 177 1
57 22 23 1
58 22 244 1
59 23 245 1
60 24 246 1
61 25 26 1
62 25 27 1
63 25 31 1
64 25 247 1

65 26 28 1
66 26 29 1
67 26 248 1
68 27 28 1
69 27 35 1
70 27 113 1
71 28 34 1
72 28 112 1
73 29 32 1
74 29 48 1
75 29 249 1
76 30 31 1
77 30 39 1
78 30 48 1
79 30 250 1
80 31 36 1
81 31 251 1
82 32 33 1
83 32 38 1
84 33 34 1
85 33 42 1
86 34 115 1
87 34 41 1
88 35 37 1
89 35 40 1
90 35 114 1
91 36 37 1
92 36 39 1
93 36 252 1
94 37 43 1
95 37 253 1
96 38 46 1
97 38 48 1
98 39 47 1
99 39 254 1
100 40 41 1
101 40 43 1
102 40 117 1
103 41 42 1
104 41 116 1
105 42 45 1
106 43 44 1
107 43 255 1
108 44 45 1
109 44 47 1
110 44 256 1
111 45 46 1
112 46 47 1
113 47 257 1
114 48 258 1

115 49 50 1
116 49 54 1
117 49 91 1
118 50 51 1
119 50 94 1
120 51 52 1
121 51 95 1
122 52 53 1
123 52 84 1
124 53 54 1
125 53 85 1
126 54 89 1
127 55 56 1
128 55 57 1
129 55 61 1
130 55 86 1
131 56 58 1
132 56 59 1
133 56 259 1
134 57 58 1
135 57 65 1
136 57 87 1
137 58 64 1
138 58 260 1
139 59 62 1
140 59 78 1
141 59 261 1
142 60 61 1
143 60 69 1
144 60 78 1
145 60 222 1
146 61 66 1
147 61 90 1
148 62 63 1
149 62 68 1
150 62 262 1
151 63 64 1
152 63 72 1
153 63 263 1
154 64 71 1
155 64 264 1
156 65 67 1
157 65 70 1
158 65 93 1
159 66 67 1
160 66 69 1
161 66 95 1
162 67 73 1
163 67 94 1
164 68 76 1

165 68 78 1
166 68 239 1
167 69 77 1
168 69 240 1
169 70 71 1
170 70 73 1
171 70 139 1
172 71 72 1
173 71 133 1
174 72 75 1
175 72 132 1
176 73 74 1
177 73 140 1
178 74 75 1
179 74 77 1
180 74 143 1
181 75 76 1
182 75 135 1
183 76 77 1
184 76 238 1
185 77 230 1
186 78 231 1
187 79 80 1
188 79 81 1
189 79 85 1
190 79 265 1
191 80 82 1
192 80 83 1
193 80 266 1
194 81 82 1
195 81 89 1
196 81 267 1
197 82 88 1
198 82 268 1
199 83 86 1
200 83 96 1
201 83 269 1
202 84 85 1
203 84 96 1
204 84 270 1
205 85 271 1
206 86 87 1
207 86 90 1
208 87 88 1
209 87 93 1
210 88 92 1
211 88 272 1
212 89 91 1
213 89 273 1
214 90 95 1

215 90 96 1
216 91 92 1
217 91 274 1
218 92 93 1
219 92 275 1
220 93 94 1
221 94 95 1
222 96 276 1
223 97 98 1
224 97 99 1
225 97 103 1
226 97 128 1
227 98 100 1
228 98 101 1
229 98 277 1
230 99 100 1
231 99 107 1
232 99 129 1
233 100 106 1
234 100 278 1
235 101 104 1
236 101 120 1
237 101 279 1
238 102 103 1
239 102 111 1
240 102 120 1
241 102 195 1
242 103 108 1
243 103 134 1
244 104 105 1
245 104 110 1
246 104 280 1
247 105 106 1
248 105 114 1
249 105 281 1
250 106 113 1
251 106 282 1
252 107 109 1
253 107 112 1
254 107 138 1
255 108 109 1
256 108 111 1
257 108 142 1
258 109 115 1
259 109 141 1
260 110 118 1
261 110 120 1
262 110 202 1
263 111 119 1
264 111 203 1

265 112 113 1
266 112 115 1
267 113 114 1
268 114 117 1
269 115 116 1
270 116 117 1
271 116 119 1
272 117 118 1
273 118 119 1
274 118 209 1
275 119 208 1
276 120 196 1
277 121 122 1
278 121 123 1
279 121 127 1
280 121 283 1
281 122 124 1
282 122 125 1
283 122 284 1
284 123 124 1
285 123 131 1
286 123 285 1
287 124 130 1
288 124 286 1
289 125 128 1
290 125 144 1
291 125 287 1
292 126 127 1
293 126 135 1
294 126 144 1
295 126 288 1
296 127 132 1
297 127 289 1
298 128 129 1
299 128 134 1
300 129 130 1
301 129 138 1
302 130 137 1
303 130 290 1
304 131 133 1
305 131 136 1
306 131 291 1
307 132 133 1
308 132 135 1
309 133 139 1
310 134 142 1
311 134 144 1
312 135 143 1
313 136 137 1
314 136 139 1

315 136 292 1
316 137 138 1
317 137 293 1
318 138 141 1
319 139 140 1
320 140 141 1
321 140 143 1
322 141 142 1
323 142 143 1
324 144 294 1
325 145 146 1
326 145 150 1
327 145 187 1
328 145 295 1
329 146 147 1
330 146 190 1
331 146 296 1
332 147 148 1
333 147 191 1
334 147 297 1
335 148 149 1
336 148 180 1
337 148 298 1
338 149 150 1
339 149 181 1
340 149 299 1
341 150 185 1
342 150 300 1
343 151 152 1
344 151 153 1
345 151 157 1
346 151 182 1
347 152 154 1
348 152 155 1
349 152 218 1
350 153 154 1
351 153 161 1
352 153 183 1
353 154 160 1
354 154 221 1
355 155 158 1
356 155 174 1
357 155 220 1
358 156 157 1
359 156 165 1
360 156 174 1
361 156 301 1
362 157 162 1
363 157 186 1
364 158 159 1

365 158 164 1
366 158 226 1
367 159 160 1
368 159 168 1
369 159 225 1
370 160 167 1
371 160 224 1
372 161 163 1
373 161 166 1
374 161 189 1
375 162 163 1
376 162 165 1
377 162 191 1
378 163 169 1
379 163 190 1
380 164 172 1
381 164 174 1
382 164 302 1
383 165 173 1
384 165 303 1
385 166 167 1
386 166 169 1
387 166 211 1
388 167 168 1
389 167 205 1
390 168 171 1
391 168 204 1
392 169 170 1
393 169 212 1
394 170 171 1
395 170 173 1
396 170 215 1
397 171 172 1
398 171 207 1
399 172 173 1
400 172 304 1
401 173 305 1
402 174 306 1
403 175 176 1
404 175 177 1
405 175 181 1
406 175 307 1
407 176 178 1
408 176 179 1
409 176 308 1
410 177 178 1
411 177 185 1
412 178 184 1
413 179 182 1
414 179 192 1

415 179 309 1
416 180 181 1
417 180 192 1
418 180 310 1
419 181 311 1
420 182 183 1
421 182 186 1
422 183 184 1
423 183 189 1
424 184 188 1
425 185 187 1
426 186 191 1
427 186 192 1
428 187 188 1
429 188 189 1
430 189 190 1
431 190 191 1
432 192 312 1
433 193 194 1
434 193 195 1
435 193 199 1
436 193 313 1
437 194 196 1
438 194 197 1
439 194 314 1
440 195 196 1
441 195 203 1
442 196 202 1
443 197 200 1
444 197 216 1
445 197 315 1
446 198 199 1
447 198 207 1
448 198 216 1
449 198 316 1
450 199 204 1
451 199 317 1
452 200 201 1
453 200 206 1
454 200 318 1
455 201 202 1
456 201 210 1
457 201 319 1
458 202 209 1
459 203 205 1
460 203 208 1
461 204 205 1
462 204 207 1
463 205 211 1
464 206 214 1

465 206 216 1
466 206 320 1
467 207 215 1
468 208 209 1
469 208 211 1
470 209 210 1
471 210 213 1
472 210 321 1
473 211 212 1
474 212 213 1
475 212 215 1
476 213 214 1
477 213 322 1
478 214 215 1
479 214 323 1
480 216 324 1
481 217 218 1
482 217 219 1
483 217 223 1
484 217 325 1
485 218 220 1
486 218 221 1
487 219 220 1
488 219 227 1
489 219 326 1
490 220 226 1
491 221 224 1
492 221 240 1
493 222 223 1
494 222 231 1
495 222 240 1
496 223 228 1
497 223 327 1
498 224 225 1
499 224 230 1
500 225 226 1
501 225 234 1
502 226 233 1
503 227 229 1
504 227 232 1
505 227 328 1
506 228 229 1
507 228 231 1
508 228 329 1
509 229 235 1
510 229 330 1
511 230 238 1
512 230 240 1
513 231 239 1
514 232 233 1

515 232 235 1
516 232 331 1
517 233 234 1
518 233 332 1
519 234 237 1
520 234 333 1
521 235 236 1
522 235 334 1
523 236 237 1
524 236 239 1
525 236 335 1
526 237 238 1
527 237 336 1
528 238 239 1

Figure S25. Solid view perspective of a section of the regular skew apeirohedron allotrope of carbon formed by fusions of nugget$_{24a}$s through their hexagonal faces via hexagonal prisms. In this figure, there are 10 fused nuggets$_{24a}$ with 240 carbon atoms. Cartesian coordinates of its atoms; the first line contains the total charge and multiplicity; the following lines contain the atomic numbers, followed by the x, y, and z coordinates in Å for each one of the atoms. Next, atomic coordinates in Tripos Mol2 file format (.mol2) with the distances also in Å.

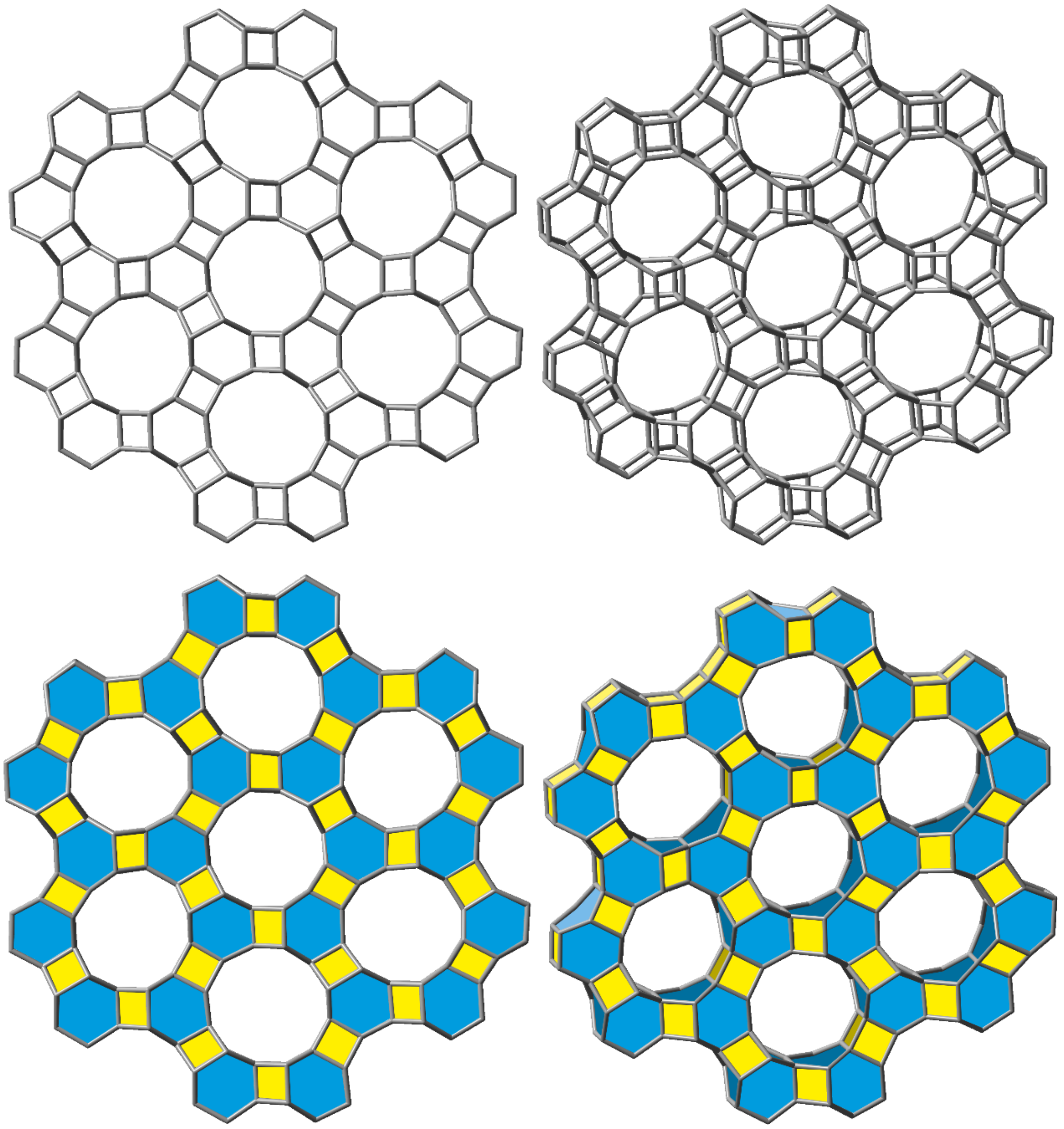

Cartesian Coordinates (Å)

0 1
6 8.563 -1.777 3.532
6 9.556 -1.191 2.364
6 9.515 -0.980 4.450
6 10.453 -0.434 3.319
1 10.536 0.641 3.272
1 9.094 -0.238 5.115
6 9.868 -2.610 1.823
6 8.934 -3.158 2.932
6 10.670 -1.896 4.995
1 11.003 -1.544 5.972
6 11.622 -1.316 3.872
1 12.392 -0.690 4.326

6	9.448	-4.145	3.951
1	8.663	-4.738	4.414
6	10.388	-3.443	5.046
1	10.110	-3.770	6.047
6	12.254	-2.300	2.841
1	13.169	-1.903	2.402
6	11.336	-2.953	1.716
1	11.741	-2.793	0.719
6	12.716	-5.040	5.125
6	11.536	-4.293	4.476
6	13.676	-4.445	3.987
6	12.460	-3.722	3.383
1	13.022	-4.766	6.126
1	14.492	-3.855	4.383
6	13.961	-5.895	3.485
1	14.996	-6.174	3.684
6	13.023	-6.480	4.603
1	13.616	-7.034	5.332
6	11.591	-4.390	2.282
6	10.650	-4.982	3.401
6	12.398	-5.329	1.383
1	12.714	-4.839	0.462
6	13.607	-6.140	1.996
1	14.479	-6.081	1.347
6	10.544	-6.497	3.599
1	9.637	-6.774	4.137
6	11.774	-7.296	4.185
1	11.450	-7.988	4.961
6	11.567	-6.618	1.096
6	10.635	-7.210	2.215
6	12.769	-7.388	1.711
6	11.849	-7.974	2.813
6	11.286	-6.938	-0.382
1	11.746	-6.208	-1.049
6	11.595	-8.402	-0.903
6	12.172	-9.432	0.103
6	13.068	-8.794	1.210
6	12.145	-9.384	2.315
6	11.238	-10.031	1.220
6	9.760	-9.576	1.309
1	9.114	-10.306	1.793
6	9.450	-8.112	1.830
1	8.701	-8.156	2.621
1	12.137	-8.374	-1.847
6	8.533	-0.909	1.232
6	7.575	-1.527	2.397
6	7.147	-0.348	1.023
6	6.187	-0.959	2.190
6	7.744	-3.026	1.907
6	8.684	-2.428	0.790

6	6.378	-1.078	-0.152
6	5.441	-1.650	0.975
6	8.381	-2.780	-0.669
1	9.226	-2.700	-1.345
6	7.102	-1.949	-1.199
1	7.348	-1.389	-2.099
6	5.220	-3.157	1.025
1	4.322	-3.434	1.573
6	6.517	-3.926	1.605
1	6.258	-4.609	2.410
6	7.511	-4.028	-0.898
6	6.587	-4.609	0.226
6	6.279	-3.263	-1.501
6	5.342	-3.866	-0.378
6	6.375	-5.098	-3.464
1	6.990	-5.042	-4.363
6	6.002	-3.627	-2.987
1	6.387	-2.886	-3.686
6	4.173	-4.803	-0.790
1	3.368	-4.826	-0.058
6	4.546	-6.274	-1.262
1	3.949	-6.995	-0.702
6	3.348	-3.278	-3.934
6	4.547	-4.004	-3.318
6	2.419	-3.865	-2.825
6	3.625	-4.590	-2.212
6	1.555	-4.590	-3.914
6	2.497	-3.993	-5.039
6	3.954	-6.036	-2.696
6	4.884	-5.442	-3.814
6	2.710	-6.859	-3.098
1	2.376	-7.496	-2.278
6	1.459	-6.123	-3.735
1	0.535	-6.445	-3.258
6	4.538	-5.696	-5.300
1	5.413	-5.563	-5.938
6	3.296	-4.953	-5.949
1	3.564	-4.517	-6.910
6	2.952	-7.694	-4.405
6	3.879	-7.103	-5.524
6	1.739	-6.927	-5.012
6	2.659	-6.340	-6.121
6	2.730	-9.219	-4.323
1	2.390	-9.519	-3.332
6	1.848	-9.944	-5.423
1	1.133	-10.623	-4.963
6	1.232	-9.063	-6.534
6	0.875	-7.607	-6.081
6	1.796	-7.017	-7.193
6	2.164	-8.464	-7.666

6	3.678	-8.770	-7.646
1	4.148	-8.691	-8.625
6	4.553	-8.051	-6.537
1	5.417	-7.579	-7.007
6	4.041	-10.091	-6.956
6	3.127	-10.680	-5.841
6	4.370	-11.507	-7.437
6	3.457	-12.098	-6.322
6	4.016	-10.007	-4.749
6	4.938	-9.417	-5.873
6	5.614	-12.230	-6.827
6	4.682	-12.831	-5.687
6	6.420	-9.832	-5.946
1	7.014	-9.100	-6.492
6	6.782	-11.288	-6.453
1	7.588	-11.269	-7.182
6	4.929	-12.467	-4.208
1	4.559	-13.210	-3.505
6	4.596	-11.003	-3.725
1	3.990	-11.048	-2.820
6	8.531	-12.143	-4.414
6	7.296	-11.560	-5.042
6	7.488	-12.851	-3.158
6	6.406	-12.160	-3.966
6	8.427	-12.076	-2.190
6	9.351	-11.462	-3.290
6	6.099	-10.715	-3.395
6	7.016	-10.103	-4.520
6	6.450	-10.459	-1.886
1	5.572	-10.592	-1.253
6	7.709	-11.184	-1.195
1	7.458	-11.646	-0.245
6	8.271	-9.263	-4.115
1	8.591	-8.617	-4.932
6	9.554	-9.971	-3.471
1	10.482	-9.679	-3.952
6	7.088	-9.033	-1.681
6	8.011	-8.433	-2.804
6	8.313	-9.782	-1.079
6	9.231	-9.187	-2.196
6	6.414	-8.075	-0.669
1	5.550	-8.544	-0.199
6	7.293	-7.353	0.445
1	6.824	-7.436	1.423
6	8.812	-7.659	0.468
6	9.165	-9.099	-0.019
6	10.084	-8.511	-1.130
6	9.743	-7.064	-0.656
6	9.122	-6.184	-1.770
1	9.838	-5.506	-2.230

6	8.236	-6.906	-2.876
1	8.578	-6.603	-3.866
6	6.951	-6.112	-2.453
6	6.026	-6.704	-1.331
6	6.920	-6.031	-0.246
6	7.837	-5.449	-1.359
6	1.990	2.996	-1.849
6	1.031	2.409	-0.712
1	0.578	3.173	-0.093
1	2.054	4.076	-1.842
6	1.117	2.290	-2.929
1	0.572	3.025	-3.523
6	0.177	1.706	-1.804
1	-0.812	2.161	-1.873
6	3.119	0.512	-3.237
1	3.992	0.630	-3.879
6	1.900	1.316	-3.847
1	2.159	1.757	-4.808
6	0.061	0.169	-1.623
1	-0.875	-0.123	-1.149
6	1.274	-0.642	-1.022
1	0.926	-1.275	-0.206
6	0.459	-0.757	-5.077
6	1.212	-0.035	-4.001
6	-0.632	-1.402	-3.816
6	0.297	-0.593	-2.926
6	-0.167	-2.767	-4.385
6	0.772	-2.207	-5.498
6	1.484	-1.469	-2.338
6	2.432	-0.880	-3.457
6	1.259	-3.028	-2.256
1	0.985	-3.363	-1.257
6	0.348	-3.753	-3.363
1	-0.436	-4.354	-2.908
6	3.123	-1.856	-4.474
1	4.038	-1.449	-4.901
6	2.238	-2.553	-5.604
1	2.648	-2.389	-6.598
6	4.566	-0.363	0.759
6	5.512	0.216	-0.323
6	5.452	0.359	1.806
6	6.456	0.963	0.655
6	3.084	-0.085	0.815
1	2.530	-0.828	1.383
6	2.732	1.395	1.270
1	1.915	1.388	1.990
6	3.874	2.370	1.689
1	3.478	3.187	2.294
6	5.097	1.700	2.405
1	5.355	1.944	3.425

6	6.053	2.301	1.317
1	6.834	2.954	1.680
6	4.831	2.948	0.569
1	4.871	4.036	0.651
6	4.604	2.530	-0.932
1	4.994	3.278	-1.622
6	4.969	1.058	-1.447
1	5.613	1.083	-2.323
6	3.468	0.735	-1.758
6	2.528	0.155	-0.631
6	2.219	1.608	-0.161
6	3.146	2.174	-1.256
6	-3.442	-3.618	-9.815
6	-4.392	-4.216	-8.674
1	-5.417	-3.877	-8.745
1	-3.958	-2.957	-10.499
6	-3.098	-5.070	-10.270
1	-3.530	-5.276	-11.250
6	-4.031	-5.657	-9.140
1	-4.897	-6.143	-9.592
6	-0.670	-4.753	-9.153
1	0.201	-4.298	-9.625
6	-1.585	-5.417	-10.259
1	-1.128	-5.345	-11.243
6	-3.418	-6.555	-8.032
1	-4.155	-7.218	-7.583
6	-2.502	-5.902	-6.927
1	-2.847	-6.201	-5.938
6	-0.845	-8.118	-10.002
6	-1.273	-6.745	-9.576
6	-1.923	-8.797	-8.748
6	-2.179	-7.317	-8.501
6	-0.610	-9.452	-8.248
6	0.332	-8.873	-9.351
6	-1.239	-6.726	-7.363
6	-0.304	-6.128	-8.488
6	-0.641	-7.746	-6.317
1	-1.186	-7.753	-5.375
6	-0.305	-9.247	-6.784
1	-0.775	-9.983	-6.138
6	1.204	-6.569	-8.542
1	1.844	-5.834	-9.027
6	1.556	-8.043	-9.049
1	2.278	-8.029	-9.862
6	-1.333	-2.695	-5.427
6	-0.413	-2.100	-6.524
6	-1.618	-1.244	-4.976
6	-0.658	-0.617	-6.128
6	-2.523	-3.575	-5.706
1	-2.816	-4.185	-4.855

6	-3.755	-2.797	-6.333
1	-4.685	-3.116	-5.864
6	-3.679	-1.242	-6.483
1	-4.680	-0.818	-6.574
6	-2.864	-0.489	-5.369
1	-3.333	0.214	-4.699
6	-1.950	0.151	-6.474
1	-1.927	1.231	-6.518
6	-2.737	-0.634	-7.601
1	-3.309	0.065	-8.214
6	-1.922	-1.620	-8.532
1	-1.689	-1.163	-9.493
6	-0.646	-2.409	-7.975
1	0.230	-2.258	-8.600
6	-1.337	-3.801	-8.150
6	-2.268	-4.387	-7.018
6	-3.469	-3.593	-7.616
6	-2.557	-3.011	-8.713
6	0.490	-14.399	-12.162
6	-0.447	-14.999	-11.010
1	-1.277	-15.580	-11.389
1	0.160	-14.660	-13.160
6	1.710	-15.148	-11.542
1	2.041	-15.952	-12.201
6	0.786	-15.736	-10.407
1	0.686	-16.815	-10.531
6	2.597	-12.774	-10.643
1	3.201	-12.059	-11.202
6	2.908	-14.239	-11.152
1	3.698	-14.235	-11.901
6	1.085	-15.384	-8.925
1	0.694	-16.130	-8.236
6	0.774	-13.925	-8.410
1	0.172	-13.976	-7.504
6	4.680	-15.113	-9.144
6	3.448	-14.504	-9.748
6	3.640	-15.827	-7.873
6	2.565	-15.090	-8.663
6	4.585	-15.043	-6.928
6	5.505	-14.432	-8.032
6	2.280	-13.638	-8.080
6	3.198	-13.034	-9.216
6	2.642	-13.382	-6.568
1	1.778	-13.434	-5.909
6	3.889	-14.159	-5.922
1	3.610	-14.680	-5.010
6	4.462	-12.191	-8.813
1	4.768	-11.486	-9.584
6	5.730	-12.951	-8.212
1	6.641	-12.716	-8.757

6	-0.880	-10.662	-9.205
6	0.020	-10.071	-10.322
6	-2.054	-9.872	-9.829
6	-1.114	-9.256	-11.005
6	-1.079	-12.137	-8.975
1	-1.413	-12.373	-7.967
6	-1.981	-12.833	-10.080
1	-2.695	-13.511	-9.614
6	-2.648	-11.951	-11.187
1	-3.493	-12.476	-11.633
6	-3.074	-10.504	-10.745
1	-4.106	-10.192	-10.740
6	-2.198	-9.896	-11.898
1	-2.712	-9.284	-12.626
6	-1.731	-11.346	-12.327
1	-2.152	-11.602	-13.301
6	-0.183	-11.666	-12.311
1	0.250	-11.602	-13.309
6	0.789	-10.972	-11.246
1	1.635	-10.486	-11.724
6	1.128	-12.335	-10.559
6	0.203	-12.918	-9.421
6	-0.702	-13.568	-10.511
6	0.196	-12.990	-11.622
6	13.833	-15.552	-1.867
6	12.886	-16.173	-0.730
1	12.852	-17.254	-0.747
1	14.302	-16.306	-2.485
6	14.675	-14.851	-0.750
1	15.669	-15.297	-0.686
6	13.746	-15.457	0.362
1	14.302	-16.187	0.951
6	13.557	-12.551	-1.514
1	13.910	-11.912	-2.323
6	14.779	-13.309	-0.870
1	15.711	-12.996	-1.337
6	12.963	-14.491	1.291
1	12.706	-14.956	2.242
6	11.714	-13.749	0.680
1	10.846	-13.903	1.321
6	15.375	-11.845	1.469
6	14.482	-12.501	0.403
6	14.422	-12.461	2.600
6	13.567	-13.092	1.489
6	14.035	-11.014	3.041
6	14.971	-10.410	1.929
6	12.345	-12.336	0.895
6	13.277	-11.733	-0.213
6	11.633	-11.400	1.885
1	10.769	-11.878	2.346

6	12.517	-10.703	2.997
1	12.047	-10.798	3.975
6	13.473	-10.214	-0.303
1	13.826	-9.908	-1.288
6	14.351	-9.526	0.817
1	15.080	-8.850	0.373
6	9.390	-13.329	-2.161
6	10.288	-12.735	-3.285
6	8.487	-14.006	-3.209
6	9.499	-13.325	-4.477
6	9.883	-14.284	-1.102
1	9.246	-14.317	-0.222
6	10.244	-15.749	-1.631
1	9.878	-16.507	-0.940
6	9.972	-16.109	-3.132
6	8.735	-15.374	-3.779
6	9.609	-14.787	-4.879
6	10.888	-15.495	-4.256
6	12.076	-14.539	-3.847
1	12.883	-14.532	-4.578
6	11.763	-13.042	-3.345
1	12.336	-12.301	-3.896
6	12.312	-13.347	-1.915
6	11.379	-13.960	-0.799
6	11.709	-15.383	-1.328
6	12.616	-14.785	-2.421
1	16.407	-12.160	1.559
1	14.942	-13.106	3.296
1	14.442	-10.794	4.029
1	15.818	-9.904	2.394
6	5.731	-17.809	-9.587
6	4.840	-18.375	-8.424
1	3.990	-18.960	-8.741
1	5.468	-18.106	-10.592
6	7.023	-18.439	-8.926
1	7.403	-19.251	-9.547
6	6.110	-19.039	-7.779
1	6.060	-20.124	-7.878
6	7.925	-15.970	-8.048
1	8.494	-15.246	-8.625
6	8.208	-17.470	-8.529
1	8.999	-17.478	-9.279
6	6.413	-18.638	-6.298
1	6.038	-19.378	-5.592
6	6.063	-17.172	-5.797
1	5.421	-17.182	-4.920
6	9.987	-18.489	-6.598
6	8.776	-17.694	-7.116
6	9.051	-19.099	-5.452
6	7.878	-18.277	-6.007

6	9.939	-18.407	-4.376
6	10.861	-17.811	-5.498
6	7.564	-16.842	-5.480
6	8.498	-16.244	-6.615
6	7.926	-16.648	-3.994
1	7.078	-16.708	-3.316
6	9.182	-17.436	-3.436
1	8.903	-17.949	-2.514
6	9.776	-15.472	-6.234
1	10.107	-14.771	-6.998
6	10.991	-16.271	-5.621
1	11.905	-16.012	-6.157
6	6.446	-15.693	-8.023
6	5.551	-16.277	-6.900
6	4.694	-16.931	-8.011
6	5.633	-16.322	-9.192
1	10.433	-19.240	-7.236
1	8.994	-20.179	-5.477
1	10.491	-19.149	-3.798
1	11.847	-18.275	-5.451

.mol2 file

@<TRIPOS>MOLECULE
Molecule Name
432 648
SMALL
NO_CHARGES

@<TRIPOS>ATOM

1	C1	8.5631	-1.7766	3.5317	C
2	C2	9.5565	-1.1906	2.3641	C
3	C3	9.5152	-0.9797	4.4500	C
4	C4	10.4530	-0.4337	3.3187	C
5	H5	10.5355	0.6413	3.2716	H
6	H6	9.0937	-0.2381	5.1150	H
7	C7	9.8678	-2.6096	1.8228	C
8	C8	8.9344	-3.1580	2.9321	C
9	C9	10.6696	-1.8962	4.9946	C
10	H10	11.0034	-1.5437	5.9720	H
11	C11	11.6224	-1.3160	3.8720	C
12	H12	12.3920	-0.6904	4.3264	H
13	C13	9.4478	-4.1452	3.9514	C
14	H14	8.6629	-4.7378	4.4142	H
15	C15	10.3881	-3.4430	5.0457	C
16	H16	10.1100	-3.7704	6.0468	H
17	C17	12.2541	-2.3000	2.8405	C
18	H18	13.1687	-1.9029	2.4021	H
19	C19	11.3363	-2.9530	1.7158	C
20	H20	11.7405	-2.7930	0.7193	H

21	C21	12.7161	-5.0401	5.1246	C
22	C22	11.5358	-4.2931	4.4759	C
23	C23	13.6760	-4.4449	3.9870	C
24	C24	12.4596	-3.7223	3.3826	C
25	H25	13.0223	-4.7661	6.1257	H
26	H26	14.4921	-3.8548	4.3833	H
27	C27	13.9607	-5.8946	3.4845	C
28	H28	14.9960	-6.1741	3.6845	H
29	C29	13.0225	-6.4802	4.6028	C
30	H30	13.6156	-7.0337	5.3322	H
31	C31	11.5908	-4.3904	2.2822	C
32	C32	10.6500	-4.9821	3.4013	C
33	C33	12.3978	-5.3288	1.3825	C
34	H34	12.7140	-4.8386	0.4620	H
35	C35	13.6071	-6.1395	1.9957	C
36	H36	14.4794	-6.0814	1.3467	H
37	C37	10.5435	-6.4970	3.5992	C
38	H38	9.6371	-6.7738	4.1374	H
39	C39	11.7737	-7.2960	4.1851	C
40	H40	11.4504	-7.9879	4.9611	H
41	C41	11.5667	-6.6181	1.0964	C
42	C42	10.6346	-7.2102	2.2151	C
43	C43	12.7687	-7.3884	1.7111	C
44	C44	11.8492	-7.9738	2.8133	C
45	C45	11.2860	-6.9380	-0.3822	C
46	H46	11.7461	-6.2083	-1.0485	H
47	C47	11.5954	-8.4023	-0.9028	C
48	C48	12.1717	-9.4323	0.1026	C
49	C49	13.0681	-8.7937	1.2105	C
50	C50	12.1446	-9.3845	2.3153	C
51	C51	11.2383	-10.0312	1.2199	C
52	C52	9.7600	-9.5760	1.3089	C
53	H53	9.1141	-10.3060	1.7931	H
54	C54	9.4503	-8.1121	1.8299	C
55	H55	8.7013	-8.1564	2.6206	H
56	H56	12.1366	-8.3743	-1.8465	H
57	C57	8.5331	-0.9086	1.2325	C
58	C58	7.5746	-1.5268	2.3972	C
59	C59	7.1467	-0.3485	1.0234	C
60	C60	6.1869	-0.9586	2.1897	C
61	C61	7.7436	-3.0259	1.9066	C
62	C62	8.6837	-2.4275	0.7903	C
63	C63	6.3781	-1.0783	-0.1524	C
64	C64	5.4411	-1.6499	0.9750	C
65	C65	8.3807	-2.7802	-0.6690	C
66	H66	9.2264	-2.7004	-1.3451	H
67	C67	7.1019	-1.9492	-1.1988	C
68	H68	7.3481	-1.3887	-2.0987	H
69	C69	5.2197	-3.1573	1.0249	C
70	H70	4.3222	-3.4344	1.5731	H

71	C71	6.5170	-3.9255	1.6052	C
72	H72	6.2585	-4.6086	2.4095	H
73	C73	7.5109	-4.0280	-0.8979	C
74	C74	6.5868	-4.6095	0.2264	C
75	C75	6.2793	-3.2629	-1.5014	C
76	C76	5.3421	-3.8661	-0.3782	C
77	C77	6.3747	-5.0985	-3.4642	C
78	H78	6.9896	-5.0418	-4.3627	H
79	C79	6.0021	-3.6265	-2.9872	C
80	H80	6.3871	-2.8857	-3.6855	H
81	C81	4.1734	-4.8028	-0.7898	C
82	H82	3.3684	-4.8264	-0.0575	H
83	C83	4.5456	-6.2737	-1.2617	C
84	H84	3.9492	-6.9945	-0.7022	H
85	C85	3.3477	-3.2780	-3.9338	C
86	C86	4.5469	-4.0039	-3.3178	C
87	C87	2.4193	-3.8655	-2.8248	C
88	C88	3.6252	-4.5905	-2.2119	C
89	C89	1.5553	-4.5902	-3.9145	C
90	C90	2.4968	-3.9927	-5.0389	C
91	C91	3.9544	-6.0357	-2.6960	C
92	C92	4.8838	-5.4422	-3.8145	C
93	C93	2.7102	-6.8595	-3.0978	C
94	H94	2.3761	-7.4964	-2.2783	H
95	C95	1.4589	-6.1226	-3.7348	C
96	H96	0.5353	-6.4451	-3.2578	H
97	C97	4.5382	-5.6956	-5.3002	C
98	H98	5.4127	-5.5632	-5.9376	H
99	C99	3.2963	-4.9527	-5.9489	C
100	H100	3.5636	-4.5167	-6.9096	H
101	C101	2.9520	-7.6945	-4.4049	C
102	C102	3.8786	-7.1033	-5.5239	C
103	C103	1.7392	-6.9271	-5.0117	C
104	C104	2.6590	-6.3398	-6.1213	C
105	C105	2.7304	-9.2190	-4.3232	C
106	H106	2.3904	-9.5186	-3.3318	H
107	C107	1.8482	-9.9436	-5.4234	C
108	H108	1.1332	-10.6227	-4.9631	H
109	C109	1.2323	-9.0635	-6.5344	C
110	C110	0.8752	-7.6073	-6.0807	C
111	C111	1.7961	-7.0173	-7.1929	C
112	C112	2.1644	-8.4637	-7.6662	C
113	C113	3.6780	-8.7699	-7.6465	C
114	H114	4.1482	-8.6915	-8.6248	H
115	C115	4.5534	-8.0509	-6.5372	C
116	H116	5.4165	-7.5794	-7.0072	H
117	C117	4.0411	-10.0906	-6.9564	C
118	C118	3.1271	-10.6797	-5.8413	C
119	C119	4.3699	-11.5069	-7.4366	C
120	C120	3.4567	-12.0980	-6.3215	C

121	C121	4.0158	-10.0074	-4.7492	C
122	C122	4.9375	-9.4168	-5.8731	C
123	C123	5.6135	-12.2304	-6.8268	C
124	C124	4.6817	-12.8313	-5.6867	C
125	C125	6.4196	-9.8324	-5.9455	C
126	H126	7.0138	-9.1001	-6.4918	H
127	C127	6.7817	-11.2879	-6.4531	C
128	H128	7.5882	-11.2691	-7.1824	H
129	C129	4.9292	-12.4670	-4.2081	C
130	H130	4.5585	-13.2104	-3.5050	H
131	C131	4.5955	-11.0031	-3.7249	C
132	H132	3.9895	-11.0477	-2.8202	H
133	C133	8.5307	-12.1426	-4.4137	C
134	C134	7.2959	-11.5603	-5.0420	C
135	C135	7.4881	-12.8511	-3.1584	C
136	C136	6.4057	-12.1596	-3.9663	C
137	C137	8.4265	-12.0764	-2.1902	C
138	C138	9.3510	-11.4616	-3.2904	C
139	C139	6.0994	-10.7150	-3.3948	C
140	C140	7.0157	-10.1026	-4.5201	C
141	C141	6.4502	-10.4594	-1.8864	C
142	H142	5.5724	-10.5922	-1.2532	H
143	C143	7.7094	-11.1844	-1.1948	C
144	H144	7.4577	-11.6456	-0.2454	H
145	C145	8.2711	-9.2634	-4.1147	C
146	H146	8.5915	-8.6175	-4.9323	H
147	C147	9.5537	-9.9712	-3.4715	C
148	H148	10.4818	-9.6793	-3.9520	H
149	C149	7.0877	-9.0325	-1.6813	C
150	C150	8.0113	-8.4329	-2.8041	C
151	C151	8.3134	-9.7816	-1.0792	C
152	C152	9.2307	-9.1869	-2.1963	C
153	C153	6.4139	-8.0748	-0.6691	C
154	H154	5.5497	-8.5443	-0.1986	H
155	C155	7.2933	-7.3531	0.4446	C
156	H156	6.8235	-7.4358	1.4228	H
157	C157	8.8122	-7.6593	0.4677	C
158	C158	9.1654	-9.0993	-0.0188	C
159	C159	10.0839	-8.5108	-1.1301	C
160	C160	9.7427	-7.0639	-0.6556	C
161	C161	9.1222	-6.1843	-1.7700	C
162	H162	9.8384	-5.5058	-2.2297	H
163	C163	8.2362	-6.9055	-2.8759	C
164	H164	8.5783	-6.6031	-3.8659	H
165	C165	6.9510	-6.1120	-2.4525	C
166	C166	6.0262	-6.7035	-1.3306	C
167	C167	6.9196	-6.0311	-0.2457	C
168	C168	7.8365	-5.4495	-1.3594	C
169	C169	1.9902	2.9959	-1.8490	C
170	C170	1.0312	2.4094	-0.7118	C

171	H171	0.5776	3.1727	-0.0934	H
172	H172	2.0541	4.0761	-1.8421	H
173	C173	1.1174	2.2905	-2.9285	C
174	H174	0.5722	3.0253	-3.5226	H
175	C175	0.1770	1.7057	-1.8035	C
176	H176	-0.8118	2.1612	-1.8726	H
177	C177	3.1190	0.5123	-3.2370	C
178	H178	3.9916	0.6302	-3.8794	H
179	C179	1.8998	1.3162	-3.8467	C
180	H180	2.1589	1.7565	-4.8076	H
181	C181	0.0610	0.1686	-1.6235	C
182	H182	-0.8748	-0.1226	-1.1490	H
183	C183	1.2744	-0.6417	-1.0216	C
184	H184	0.9259	-1.2752	-0.2061	H
185	C185	0.4589	-0.7566	-5.0768	C
186	C186	1.2120	-0.0350	-4.0013	C
187	C187	-0.6323	-1.4022	-3.8162	C
188	C188	0.2975	-0.5934	-2.9265	C
189	C189	-0.1666	-2.7671	-4.3845	C
190	C190	0.7725	-2.2072	-5.4977	C
191	C191	1.4842	-1.4693	-2.3380	C
192	C192	2.4320	-0.8805	-3.4574	C
193	C193	1.2591	-3.0278	-2.2555	C
194	H194	0.9848	-3.3634	-1.2571	H
195	C195	0.3484	-3.7534	-3.3628	C
196	H196	-0.4357	-4.3536	-2.9082	H
197	C197	3.1235	-1.8561	-4.4737	C
198	H198	4.0379	-1.4486	-4.9009	H
199	C199	2.2379	-2.5529	-5.6042	C
200	H200	2.6477	-2.3893	-6.5977	H
201	C201	4.5659	-0.3631	0.7588	C
202	C202	5.5117	0.2156	-0.3227	C
203	C203	5.4518	0.3588	1.8060	C
204	C204	6.4561	0.9628	0.6551	C
205	C205	3.0842	-0.0853	0.8150	C
206	H206	2.5298	-0.8281	1.3833	H
207	C207	2.7315	1.3949	1.2695	C
208	H208	1.9149	1.3875	1.9902	H
209	C209	3.8745	2.3705	1.6890	C
210	H210	3.4780	3.1873	2.2938	H
211	C211	5.0966	1.7003	2.4054	C
212	H212	5.3551	1.9439	3.4245	H
213	C213	6.0530	2.3005	1.3165	C
214	H214	6.8343	2.9542	1.6802	H
215	C215	4.8311	2.9483	0.5685	C
216	H216	4.8713	4.0358	0.6513	H
217	C217	4.6045	2.5301	-0.9319	C
218	H218	4.9938	3.2776	-1.6222	H
219	C219	4.9692	1.0582	-1.4473	C
220	H220	5.6129	1.0834	-2.3226	H

221	C221	3.4682	0.7346	-1.7577	C
222	C222	2.5282	0.1546	-0.6314	C
223	C223	2.2190	1.6079	-0.1605	C
224	C224	3.1461	2.1740	-1.2557	C
225	C225	-3.4422	-3.6176	-9.8150	C
226	C226	-4.3923	-4.2165	-8.6741	C
227	H227	-5.4173	-3.8774	-8.7448	H
228	H228	-3.9579	-2.9568	-10.4993	H
229	C229	-3.0982	-5.0697	-10.2696	C
230	H230	-3.5302	-5.2761	-11.2499	H
231	C231	-4.0312	-5.6571	-9.1404	C
232	H232	-4.8971	-6.1433	-9.5918	H
233	C233	-0.6703	-4.7528	-9.1534	C
234	H234	0.2009	-4.2981	-9.6251	H
235	C235	-1.5852	-5.4172	-10.2587	C
236	H236	-1.1277	-5.3447	-11.2435	H
237	C237	-3.4180	-6.5546	-8.0320	C
238	H238	-4.1550	-7.2183	-7.5828	H
239	C239	-2.5015	-5.9017	-6.9270	C
240	H240	-2.8471	-6.2012	-5.9379	H
241	C241	-0.8452	-8.1176	-10.0023	C
242	C242	-1.2731	-6.7454	-9.5759	C
243	C243	-1.9228	-8.7967	-8.7479	C
244	C244	-2.1785	-7.3175	-8.5006	C
245	C245	-0.6096	-9.4516	-8.2482	C
246	C246	0.3315	-8.8733	-9.3510	C
247	C247	-1.2394	-6.7257	-7.3632	C
248	C248	-0.3037	-6.1278	-8.4878	C
249	C249	-0.6411	-7.7463	-6.3172	C
250	H250	-1.1864	-7.7535	-5.3752	H
251	C251	-0.3049	-9.2466	-6.7839	C
252	H252	-0.7751	-9.9835	-6.1376	H
253	C253	1.2041	-6.5689	-8.5423	C
254	H254	1.8444	-5.8344	-9.0275	H
255	C255	1.5560	-8.0425	-9.0490	C
256	H256	2.2776	-8.0294	-9.8621	H
257	C257	-1.3331	-2.6954	-5.4271	C
258	C258	-0.4129	-2.1001	-6.5241	C
259	C259	-1.6183	-1.2444	-4.9759	C
260	C260	-0.6576	-0.6172	-6.1282	C
261	C261	-2.5229	-3.5746	-5.7058	C
262	H262	-2.8159	-4.1849	-4.8553	H
263	C263	-3.7550	-2.7967	-6.3333	C
264	H264	-4.6846	-3.1161	-5.8641	H
265	C265	-3.6788	-1.2417	-6.4830	C
266	H266	-4.6801	-0.8181	-6.5739	H
267	C267	-2.8641	-0.4895	-5.3688	C
268	H268	-3.3334	0.2135	-4.6986	H
269	C269	-1.9503	0.1509	-6.4740	C
270	H270	-1.9267	1.2307	-6.5183	H

271	C271	-2.7371	-0.6343	-7.6009	C
272	H272	-3.3091	0.0648	-8.2138	H
273	C273	-1.9220	-1.6203	-8.5316	C
274	H274	-1.6894	-1.1628	-9.4927	H
275	C275	-0.6465	-2.4087	-7.9749	C
276	H276	0.2297	-2.2582	-8.6000	H
277	C277	-1.3367	-3.8005	-8.1499	C
278	C278	-2.2681	-4.3866	-7.0182	C
279	C279	-3.4692	-3.5929	-7.6162	C
280	C280	-2.5569	-3.0115	-8.7134	C
281	C281	0.4897	-14.3991	-12.1624	C
282	C282	-0.4467	-14.9993	-11.0100	C
283	H283	-1.2774	-15.5798	-11.3890	H
284	H284	0.1597	-14.6599	-13.1595	H
285	C285	1.7104	-15.1478	-11.5424	C
286	H286	2.0408	-15.9519	-12.2015	H
287	C287	0.7858	-15.7359	-10.4068	C
288	H288	0.6858	-16.8151	-10.5313	H
289	C289	2.5970	-12.7739	-10.6431	C
290	H290	3.2009	-12.0589	-11.2019	H
291	C291	2.9082	-14.2385	-11.1517	C
292	H292	3.6978	-14.2353	-11.9009	H
293	C293	1.0849	-15.3835	-8.9252	C
294	H294	0.6936	-16.1302	-8.2357	H
295	C295	0.7743	-13.9250	-8.4104	C
296	H296	0.1716	-13.9764	-7.5036	H
297	C297	4.6804	-15.1127	-9.1441	C
298	C298	3.4476	-14.5044	-9.7480	C
299	C299	3.6401	-15.8270	-7.8734	C
300	C300	2.5646	-15.0900	-8.6626	C
301	C301	4.5852	-15.0426	-6.9285	C
302	C302	5.5054	-14.4315	-8.0320	C
303	C303	2.2799	-13.6380	-8.0804	C
304	C304	3.1977	-13.0337	-9.2163	C
305	C305	2.6422	-13.3820	-6.5683	C
306	H306	1.7776	-13.4336	-5.9089	H
307	C307	3.8886	-14.1593	-5.9222	C
308	H308	3.6100	-14.6802	-5.0098	H
309	C309	4.4617	-12.1906	-8.8132	C
310	H310	4.7683	-11.4858	-9.5837	H
311	C311	5.7296	-12.9509	-8.2120	C
312	H312	6.6406	-12.7162	-8.7570	H
313	C313	-0.8797	-10.6616	-9.2054	C
314	C314	0.0203	-10.0711	-10.3216	C
315	C315	-2.0539	-9.8723	-9.8295	C
316	C316	-1.1139	-9.2561	-11.0052	C
317	C317	-1.0791	-12.1372	-8.9745	C
318	H318	-1.4131	-12.3734	-7.9674	H
319	C319	-1.9811	-12.8327	-10.0796	C
320	H320	-2.6948	-13.5113	-9.6143	H

321	C321	-2.6478	-11.9511	-11.1869	C
322	H322	-3.4933	-12.4762	-11.6334	H
323	C323	-3.0735	-10.5039	-10.7445	C
324	H324	-4.1061	-10.1917	-10.7399	H
325	C325	-2.1979	-9.8960	-11.8981	C
326	H326	-2.7118	-9.2836	-12.6257	H
327	C327	-1.7312	-11.3464	-12.3268	C
328	H328	-2.1516	-11.6019	-13.3012	H
329	C329	-0.1826	-11.6660	-12.3110	C
330	H330	0.2496	-11.6024	-13.3092	H
331	C331	0.7886	-10.9721	-11.2459	C
332	H332	1.6350	-10.4865	-11.7239	H
333	C333	1.1283	-12.3352	-10.5590	C
334	C334	0.2025	-12.9180	-9.4212	C
335	C335	-0.7020	-13.5683	-10.5110	C
336	C336	0.1958	-12.9899	-11.6218	C
337	C337	13.8330	-15.5519	-1.8669	C
338	C338	12.8858	-16.1727	-0.7297	C
339	H339	12.8515	-17.2540	-0.7472	H
340	H340	14.3019	-16.3062	-2.4853	H
341	C341	14.6753	-14.8509	-0.7505	C
342	H342	15.6691	-15.2965	-0.6864	H
343	C343	13.7460	-15.4566	0.3617	C
344	H344	14.3021	-16.1873	0.9507	H
345	C345	13.5571	-12.5509	-1.5136	C
346	H346	13.9097	-11.9115	-2.3227	H
347	C347	14.7786	-13.3094	-0.8701	C
348	H348	15.7113	-12.9958	-1.3368	H
349	C349	12.9633	-14.4914	1.2906	C
350	H350	12.7057	-14.9557	2.2417	H
351	C351	11.7140	-13.7490	0.6804	C
352	H352	10.8459	-13.9030	1.3213	H
353	C353	15.3754	-11.8452	1.4694	C
354	C354	14.4821	-12.5006	0.4033	C
355	C355	14.4215	-12.4607	2.6003	C
356	C356	13.5672	-13.0920	1.4891	C
357	C357	14.0349	-11.0144	3.0410	C
358	C358	14.9711	-10.4103	1.9294	C
359	C359	12.3445	-12.3357	0.8946	C
360	C360	13.2773	-11.7335	-0.2130	C
361	C361	11.6330	-11.3997	1.8850	C
362	H362	10.7688	-11.8781	2.3460	H
363	C363	12.5166	-10.7033	2.9970	C
364	H364	12.0471	-10.7978	3.9750	H
365	C365	13.4731	-10.2139	-0.3035	C
366	H366	13.8265	-9.9079	-1.2881	H
367	C367	14.3509	-9.5261	0.8165	C
368	H368	15.0801	-8.8502	0.3728	H
369	C369	9.3895	-13.3293	-2.1613	C
370	C370	10.2881	-12.7345	-3.2848	C

371	C371	8.4872	-14.0063	-3.2091	C
372	C372	9.4987	-13.3251	-4.4768	C
373	C373	9.8829	-14.2842	-1.1024	C
374	H374	9.2461	-14.3174	-0.2219	H
375	C375	10.2438	-15.7490	-1.6311	C
376	H376	9.8783	-16.5067	-0.9399	H
377	C377	9.9716	-16.1094	-3.1322	C
378	C378	8.7347	-15.3744	-3.7790	C
379	C379	9.6086	-14.7868	-4.8789	C
380	C380	10.8882	-15.4946	-4.2560	C
381	C381	12.0759	-14.5388	-3.8467	C
382	H382	12.8825	-14.5318	-4.5783	H
383	C383	11.7631	-13.0420	-3.3446	C
384	H384	12.3359	-12.3010	-3.8960	H
385	C385	12.3124	-13.3470	-1.9146	C
386	C386	11.3790	-13.9597	-0.7986	C
387	C387	11.7090	-15.3828	-1.3282	C
388	C388	12.6163	-14.7853	-2.4212	C
389	H389	16.4067	-12.1599	1.5588	H
390	H390	14.9418	-13.1059	3.2959	H
391	H391	14.4420	-10.7945	4.0290	H
392	H392	15.8178	-9.9038	2.3944	H
393	C393	5.7310	-17.8088	-9.5872	C
394	C394	4.8396	-18.3748	-8.4244	C
395	H395	3.9904	-18.9599	-8.7414	H
396	H396	5.4675	-18.1063	-10.5924	H
397	C397	7.0226	-18.4386	-8.9256	C
398	H398	7.4032	-19.2513	-9.5470	H
399	C399	6.1098	-19.0387	-7.7795	C
400	H400	6.0596	-20.1240	-7.8776	H
401	C401	7.9250	-15.9696	-8.0483	C
402	H402	8.4943	-15.2461	-8.6254	H
403	C403	8.2082	-17.4696	-8.5292	C
404	H404	8.9992	-17.4781	-9.2791	H
405	C405	6.4126	-18.6381	-6.2975	C
406	H406	6.0380	-19.3785	-5.5916	H
407	C407	6.0632	-17.1716	-5.7973	C
408	H408	5.4213	-17.1823	-4.9200	H
409	C409	9.9868	-18.4887	-6.5983	C
410	C410	8.7759	-17.6943	-7.1158	C
411	C411	9.0512	-19.0989	-5.4519	C
412	C412	7.8777	-18.2766	-6.0066	C
413	C413	9.9390	-18.4071	-4.3762	C
414	C414	10.8610	-17.8114	-5.4980	C
415	C415	7.5636	-16.8424	-5.4804	C
416	C416	8.4985	-16.2444	-6.6153	C
417	C417	7.9256	-16.6479	-3.9941	C
418	H418	7.0778	-16.7082	-3.3160	H
419	C419	9.1816	-17.4363	-3.4356	C
420	H420	8.9027	-17.9489	-2.5137	H

421 C421 9.7759 -15.4717 -6.2340 C
422 H422 10.1069 -14.7708 -6.9983 H
423 C423 10.9911 -16.2714 -5.6210 C
424 H424 11.9049 -16.0125 -6.1572 H
425 C425 6.4457 -15.6926 -8.0233 C
426 C426 5.5513 -16.2771 -6.8997 C
427 C427 4.6937 -16.9308 -8.0115 C
428 C428 5.6334 -16.3224 -9.1916 C
429 H429 10.4327 -19.2401 -7.2362 H
430 H430 8.9942 -20.1790 -5.4768 H
431 H431 10.4912 -19.1491 -3.7983 H
432 H432 11.8471 -18.2754 -5.4510 H
@<TRIPOS>BOND
1 1 2 1
2 1 3 1
3 1 8 1
4 1 58 1
5 2 4 1
6 2 7 1
7 2 57 1
8 3 6 1
9 3 4 1
10 3 9 1
11 4 5 1
12 4 11 1
13 7 8 1
14 7 19 1
15 7 62 1
16 8 13 1
17 8 61 1
18 9 10 1
19 9 11 1
20 9 15 1
21 11 12 1
22 11 17 1
23 13 14 1
24 13 15 1
25 13 32 1
26 15 16 1
27 15 22 1
28 17 18 1
29 17 19 1
30 17 24 1
31 19 20 1
32 19 31 1
33 21 25 1
34 21 22 1
35 21 23 1
36 21 29 1
37 22 24 1

38 22 32 1
39 23 26 1
40 23 24 1
41 23 27 1
42 24 31 1
43 27 28 1
44 27 29 1
45 27 35 1
46 29 30 1
47 29 39 1
48 31 32 1
49 31 33 1
50 32 37 1
51 33 34 1
52 33 35 1
53 33 41 1
54 35 36 1
55 35 43 1
56 37 38 1
57 37 39 1
58 37 42 1
59 39 40 1
60 39 44 1
61 41 42 1
62 41 43 1
63 41 45 1
64 42 44 1
65 42 54 1
66 43 44 1
67 43 49 1
68 44 50 1
69 45 46 1
70 45 47 1
71 45 160 1
72 47 48 1
73 47 159 1
74 47 56 1
75 48 49 1
76 48 51 1
77 48 365 1
78 49 50 1
79 49 367 1
80 50 51 1
81 50 363 1
82 51 52 1
83 51 361 1
84 52 53 1
85 52 54 1
86 52 158 1
87 54 55 1

88 54 157 1
89 57 58 1
90 57 59 1
91 57 62 1
92 58 60 1
93 58 61 1
94 59 60 1
95 59 63 1
96 59 204 1
97 60 64 1
98 60 203 1
99 61 62 1
100 61 71 1
101 62 65 1
102 63 64 1
103 63 67 1
104 63 202 1
105 64 69 1
106 64 201 1
107 65 66 1
108 65 67 1
109 65 73 1
110 67 68 1
111 67 75 1
112 69 70 1
113 69 71 1
114 69 76 1
115 71 72 1
116 71 74 1
117 73 74 1
118 73 75 1
119 73 168 1
120 74 76 1
121 74 167 1
122 75 76 1
123 75 79 1
124 76 81 1
125 77 78 1
126 77 79 1
127 77 92 1
128 77 165 1
129 79 80 1
130 79 86 1
131 81 82 1
132 81 83 1
133 81 88 1
134 83 84 1
135 83 91 1
136 83 166 1
137 85 86 1

138 85 87 1
139 85 90 1
140 85 197 1
141 86 88 1
142 86 92 1
143 87 88 1
144 87 89 1
145 87 193 1
146 88 91 1
147 89 90 1
148 89 95 1
149 89 195 1
150 90 99 1
151 90 199 1
152 91 92 1
153 91 93 1
154 92 97 1
155 93 94 1
156 93 95 1
157 93 101 1
158 95 96 1
159 95 103 1
160 97 98 1
161 97 99 1
162 97 102 1
163 99 100 1
164 99 104 1
165 101 102 1
166 101 103 1
167 101 105 1
168 102 104 1
169 102 115 1
170 103 104 1
171 103 110 1
172 104 111 1
173 105 106 1
174 105 107 1
175 105 121 1
176 107 108 1
177 107 109 1
178 107 118 1
179 109 110 1
180 109 112 1
181 109 251 1
182 110 111 1
183 110 249 1
184 111 112 1
185 111 253 1
186 112 113 1
187 112 255 1

188 113 114 1
189 113 115 1
190 113 117 1
191 115 116 1
192 115 122 1
193 117 118 1
194 117 119 1
195 117 122 1
196 118 120 1
197 118 121 1
198 119 120 1
199 119 123 1
200 119 309 1
201 120 124 1
202 120 305 1
203 121 122 1
204 121 131 1
205 122 125 1
206 123 124 1
207 123 127 1
208 123 311 1
209 124 129 1
210 124 307 1
211 125 126 1
212 125 127 1
213 125 140 1
214 127 128 1
215 127 134 1
216 129 130 1
217 129 131 1
218 129 136 1
219 131 132 1
220 131 139 1
221 133 134 1
222 133 135 1
223 133 138 1
224 133 372 1
225 134 136 1
226 134 140 1
227 135 136 1
228 135 137 1
229 135 371 1
230 136 139 1
231 137 138 1
232 137 143 1
233 137 369 1
234 138 147 1
235 138 370 1
236 139 140 1
237 139 141 1

238 140 145 1
239 141 142 1
240 141 143 1
241 141 149 1
242 143 144 1
243 143 151 1
244 145 146 1
245 145 147 1
246 145 150 1
247 147 148 1
248 147 152 1
249 149 150 1
250 149 151 1
251 149 153 1
252 150 152 1
253 150 163 1
254 151 152 1
255 151 158 1
256 152 159 1
257 153 154 1
258 153 155 1
259 153 166 1
260 155 156 1
261 155 157 1
262 155 167 1
263 157 160 1
264 157 158 1
265 158 159 1
266 159 160 1
267 160 161 1
268 161 162 1
269 161 163 1
270 161 168 1
271 163 164 1
272 163 165 1
273 165 166 1
274 165 168 1
275 166 167 1
276 167 168 1
277 169 172 1
278 169 170 1
279 169 173 1
280 169 224 1
281 170 171 1
282 170 175 1
283 170 223 1
284 173 174 1
285 173 175 1
286 173 179 1
287 175 176 1

288 175 181 1
289 177 178 1
290 177 179 1
291 177 192 1
292 177 221 1
293 179 180 1
294 179 186 1
295 181 182 1
296 181 183 1
297 181 188 1
298 183 184 1
299 183 191 1
300 183 222 1
301 185 186 1
302 185 187 1
303 185 190 1
304 185 260 1
305 186 188 1
306 186 192 1
307 187 188 1
308 187 189 1
309 187 259 1
310 188 191 1
311 189 190 1
312 189 195 1
313 189 257 1
314 190 199 1
315 190 258 1
316 191 192 1
317 191 193 1
318 192 197 1
319 193 194 1
320 193 195 1
321 195 196 1
322 197 198 1
323 197 199 1
324 199 200 1
325 201 202 1
326 201 203 1
327 201 205 1
328 202 204 1
329 202 219 1
330 203 204 1
331 203 211 1
332 204 213 1
333 205 206 1
334 205 207 1
335 205 222 1
336 207 208 1
337 207 209 1

338 207 223 1
339 209 210 1
340 209 211 1
341 209 215 1
342 211 212 1
343 211 213 1
344 213 214 1
345 213 215 1
346 215 216 1
347 215 217 1
348 217 218 1
349 217 219 1
350 217 224 1
351 219 220 1
352 219 221 1
353 221 222 1
354 221 224 1
355 222 223 1
356 223 224 1
357 225 228 1
358 225 226 1
359 225 229 1
360 225 280 1
361 226 227 1
362 226 231 1
363 226 279 1
364 229 230 1
365 229 231 1
366 229 235 1
367 231 232 1
368 231 237 1
369 233 234 1
370 233 235 1
371 233 248 1
372 233 277 1
373 235 236 1
374 235 242 1
375 237 238 1
376 237 239 1
377 237 244 1
378 239 240 1
379 239 247 1
380 239 278 1
381 241 242 1
382 241 243 1
383 241 246 1
384 241 316 1
385 242 244 1
386 242 248 1
387 243 244 1

388 243 245 1
389 243 315 1
390 244 247 1
391 245 246 1
392 245 251 1
393 245 313 1
394 246 255 1
395 246 314 1
396 247 248 1
397 247 249 1
398 248 253 1
399 249 250 1
400 249 251 1
401 251 252 1
402 253 254 1
403 253 255 1
404 255 256 1
405 257 258 1
406 257 259 1
407 257 261 1
408 258 260 1
409 258 275 1
410 259 260 1
411 259 267 1
412 260 269 1
413 261 262 1
414 261 263 1
415 261 278 1
416 263 264 1
417 263 265 1
418 263 279 1
419 265 266 1
420 265 267 1
421 265 271 1
422 267 268 1
423 267 269 1
424 269 270 1
425 269 271 1
426 271 272 1
427 271 273 1
428 273 274 1
429 273 275 1
430 273 280 1
431 275 276 1
432 275 277 1
433 277 278 1
434 277 280 1
435 278 279 1
436 279 280 1
437 281 284 1

438 281 282 1
439 281 285 1
440 281 336 1
441 282 283 1
442 282 287 1
443 282 335 1
444 285 286 1
445 285 287 1
446 285 291 1
447 287 288 1
448 287 293 1
449 289 290 1
450 289 291 1
451 289 304 1
452 289 333 1
453 291 292 1
454 291 298 1
455 293 294 1
456 293 295 1
457 293 300 1
458 295 296 1
459 295 303 1
460 295 334 1
461 297 298 1
462 297 299 1
463 297 302 1
464 297 428 1
465 298 300 1
466 298 304 1
467 299 300 1
468 299 301 1
469 299 427 1
470 300 303 1
471 301 302 1
472 301 307 1
473 301 426 1
474 302 311 1
475 302 425 1
476 303 304 1
477 303 305 1
478 304 309 1
479 305 306 1
480 305 307 1
481 307 308 1
482 309 310 1
483 309 311 1
484 311 312 1
485 313 314 1
486 313 315 1
487 313 317 1

488 314 316 1
489 314 331 1
490 315 316 1
491 315 323 1
492 316 325 1
493 317 318 1
494 317 319 1
495 317 334 1
496 319 320 1
497 319 321 1
498 319 335 1
499 321 322 1
500 321 323 1
501 321 327 1
502 323 324 1
503 323 325 1
504 325 326 1
505 325 327 1
506 327 328 1
507 327 329 1
508 329 330 1
509 329 331 1
510 329 336 1
511 331 332 1
512 331 333 1
513 333 334 1
514 333 336 1
515 334 335 1
516 335 336 1
517 337 340 1
518 337 338 1
519 337 341 1
520 337 388 1
521 338 339 1
522 338 343 1
523 338 387 1
524 341 342 1
525 341 343 1
526 341 347 1
527 343 344 1
528 343 349 1
529 345 346 1
530 345 347 1
531 345 360 1
532 345 385 1
533 347 348 1
534 347 354 1
535 349 350 1
536 349 351 1
537 349 356 1

538 351 352 1
539 351 359 1
540 351 386 1
541 353 354 1
542 353 355 1
543 353 358 1
544 353 389 1
545 354 356 1
546 354 360 1
547 355 356 1
548 355 357 1
549 355 390 1
550 356 359 1
551 357 358 1
552 357 363 1
553 357 391 1
554 358 367 1
555 358 392 1
556 359 360 1
557 359 361 1
558 360 365 1
559 361 362 1
560 361 363 1
561 363 364 1
562 365 366 1
563 365 367 1
564 367 368 1
565 369 370 1
566 369 371 1
567 369 373 1
568 370 372 1
569 370 383 1
570 371 372 1
571 371 378 1
572 372 379 1
573 373 374 1
574 373 375 1
575 373 386 1
576 375 376 1
577 375 377 1
578 375 387 1
579 377 378 1
580 377 380 1
581 377 419 1
582 378 379 1
583 378 417 1
584 379 380 1
585 379 421 1
586 380 381 1
587 380 423 1

588 381 382 1
589 381 383 1
590 381 388 1
591 383 384 1
592 383 385 1
593 385 386 1
594 385 388 1
595 386 387 1
596 387 388 1
597 393 396 1
598 393 394 1
599 393 397 1
600 393 428 1
601 394 395 1
602 394 399 1
603 394 427 1
604 397 398 1
605 397 399 1
606 397 403 1
607 399 400 1
608 399 405 1
609 401 402 1
610 401 403 1
611 401 416 1
612 401 425 1
613 403 404 1
614 403 410 1
615 405 406 1
616 405 407 1
617 405 412 1
618 407 408 1
619 407 415 1
620 407 426 1
621 409 410 1
622 409 411 1
623 409 414 1
624 409 429 1
625 410 412 1
626 410 416 1
627 411 412 1
628 411 413 1
629 411 430 1
630 412 415 1
631 413 414 1
632 413 419 1
633 413 431 1
634 414 423 1
635 414 432 1
636 415 416 1
637 415 417 1

638 416 421 1
639 417 418 1
640 417 419 1
641 419 420 1
642 421 422 1
643 421 423 1
644 423 424 1
645 425 426 1
646 425 428 1
647 426 427 1
648 427 428 1

Figure S26. Two perspectives of compound $C_{288}H_{144}$ obtained by square face-fusions of 24 units of nugget$_{16}$. Cartesian coordinates of its atoms; the first line contains the total charge and multiplicity; the following lines contain the atomic numbers, followed by the x, y, and z coordinates in Å for each one of the atoms. Next, atomic coordinates in Tripos Mol2 file format (.mol2) with the distances also in Å.

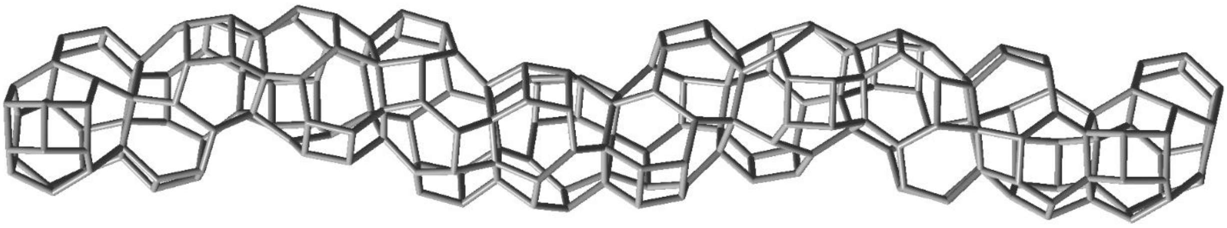

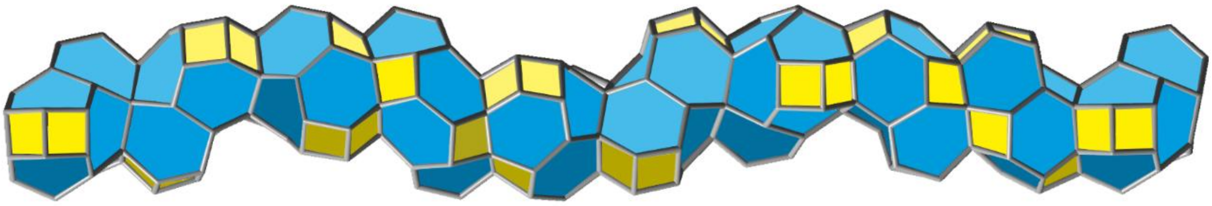

Cartesian Coordinates (Å)

0 1

6	3.438	-8.032	0.566
6	2.717	-7.433	-0.633
6	3.091	-7.962	-2.015
6	4.041	-9.068	-2.485
6	4.722	-9.925	-1.417
6	4.447	-9.349	0.094
6	5.970	-6.729	3.144
6	7.028	-7.625	2.416
1	7.706	-8.068	3.139
1	6.153	-6.659	4.210
6	5.932	-8.610	2.024
1	6.023	-9.523	2.618
6	4.872	-7.679	2.656
1	4.379	-8.173	3.494
6	3.877	-7.011	1.708
1	3.050	-6.941	2.426
6	3.647	-5.045	-0.003
1	3.103	-4.096	0.022
6	2.966	-5.971	-1.026
1	2.027	-5.509	-1.346
6	3.682	-6.532	-2.295
1	3.265	-6.113	-3.212
6	3.956	-5.456	1.473
1	3.349	-4.896	2.185
6	5.927	-8.929	0.535

1	6.459	-9.896	0.501
6	5.337	-8.728	-3.232
1	5.488	-9.121	-4.231
6	6.107	-9.551	-2.147
1	6.703	-10.323	-2.626
6	6.957	-8.325	-1.815
1	7.972	-8.460	-2.192
6	6.963	-7.989	-0.328
1	7.923	-8.419	0.002
6	5.251	-6.390	-2.229
1	5.290	-5.726	-3.103
6	6.132	-7.489	-2.817
1	6.762	-7.130	-3.632
6	7.494	-6.373	1.519
6	6.416	-5.580	2.234
6	5.396	-4.841	1.357
6	5.150	-4.772	-0.156
6	6.014	-5.659	-1.033
6	7.207	-6.409	-0.128
6	11.303	-6.017	1.141
6	11.009	-5.612	-0.342
1	11.765	-6.026	-1.002
1	12.233	-6.563	1.244
6	9.757	-6.485	-0.338
1	9.923	-7.380	-0.941
6	9.992	-6.807	1.154
1	10.173	-7.874	1.295
6	8.963	-6.253	2.136
1	9.075	-7.045	2.888
6	8.249	-4.068	3.385
1	8.304	-3.756	4.433
6	6.773	-4.313	3.023
1	6.177	-4.270	3.939
6	6.012	-3.512	1.919
1	5.237	-2.874	2.345
6	9.384	-5.112	3.137
1	9.785	-5.504	4.072
6	8.511	-5.738	-0.794
1	8.426	-6.057	-1.847
6	5.431	-3.482	-0.935
1	4.627	-3.018	-1.494
6	6.386	-4.321	-1.849
1	6.098	-4.215	-2.891
6	7.542	-3.386	-1.507
1	7.779	-2.756	-2.366
6	8.777	-4.127	-1.011
1	9.402	-4.162	-1.919
6	6.976	-2.726	0.952
1	6.577	-1.746	1.244
6	6.627	-2.617	-0.530

1	6.497	-1.583	-0.855
6	11.042	-4.054	0.061
6	11.272	-4.524	1.485
6	10.313	-3.988	2.557
6	9.062	-3.099	2.516
6	8.561	-2.682	1.147
6	9.631	-3.163	-0.045
6	12.985	-1.818	-2.386
6	11.850	-0.764	-2.154
1	11.622	-0.247	-3.081
1	13.379	-1.784	-3.396
6	10.851	-1.889	-1.900
1	10.167	-1.980	-2.746
6	11.988	-2.924	-2.032
1	11.811	-3.586	-2.882
6	12.360	-3.687	-0.763
1	12.704	-4.600	-1.265
6	13.691	-3.467	1.478
1	14.622	-3.915	1.839
6	12.517	-4.077	2.264
1	12.894	-4.911	2.864
6	11.552	-3.239	3.162
1	11.673	-3.479	4.219
6	13.755	-3.422	-0.081
1	14.540	-4.070	-0.473
6	10.088	-1.719	-0.592
1	9.135	-1.287	-0.938
6	9.094	-1.680	3.096
1	8.395	-1.416	3.882
6	8.630	-1.169	1.690
1	7.726	-0.576	1.785
6	9.861	-0.271	1.606
1	9.572	0.774	1.737
6	10.649	-0.465	0.317
1	10.297	0.381	-0.296
6	11.638	-1.692	2.882
1	11.989	-1.460	3.896
6	10.368	-0.846	2.946
1	10.423	-0.065	3.705
6	12.731	-0.045	-1.015
6	13.763	-1.131	-1.258
6	14.234	-1.929	-0.035
6	13.832	-1.939	1.447
6	12.674	-1.048	1.850
6	12.193	-0.087	0.568
6	12.761	3.793	-1.210
6	12.689	3.765	0.353
1	12.050	4.565	0.715
1	12.230	4.638	-1.633
6	11.926	2.444	0.299

1	10.879	2.606	0.563
6	12.081	2.421	-1.236
1	11.107	2.471	-1.724
6	12.969	1.319	-1.811
1	12.453	1.237	-2.775
6	15.470	0.653	-2.192
1	16.094	0.584	-3.088
6	15.196	-0.769	-1.672
1	15.556	-1.487	-2.415
6	15.641	-1.274	-0.263
1	16.418	-2.036	-0.333
6	14.346	1.701	-2.473
1	14.251	1.917	-3.537
6	12.552	1.362	1.171
1	11.921	1.403	2.074
6	14.795	-1.419	2.520
1	15.097	-2.081	3.323
6	13.665	-0.428	2.957
1	13.450	-0.545	4.015
6	14.601	0.753	2.721
1	14.916	1.178	3.677
6	13.995	1.820	1.818
1	13.643	2.566	2.550
6	16.032	-0.103	0.715
1	17.073	-0.440	0.809
6	15.684	-0.215	2.198
1	16.567	-0.191	2.838
6	14.291	3.895	0.444
6	14.286	3.863	-1.073
6	15.181	2.814	-1.748
6	16.072	1.680	-1.223
6	16.058	1.432	0.273
6	15.172	2.614	1.059
6	17.598	1.769	-1.339
6	17.660	1.574	0.213
1	18.297	0.730	0.461
1	18.132	0.992	-1.875
6	18.422	2.888	0.353
1	19.468	2.690	0.598
6	18.277	3.130	-1.165
1	19.255	3.150	-1.649
6	17.393	4.302	-1.583
1	17.915	4.521	-2.522
6	14.894	5.017	-1.882
1	14.275	5.214	-2.762
6	15.164	6.351	-1.163
1	14.808	7.168	-1.797
6	14.709	6.650	0.301
1	13.932	7.414	0.335
6	16.020	4.019	-2.301

1	16.122	3.957	-3.385
6	17.791	3.835	1.365
1	18.416	3.666	2.257
6	15.537	6.396	3.082
1	15.229	6.938	3.969
6	16.665	5.353	3.381
1	16.872	5.318	4.446
6	15.730	4.218	2.973
1	15.410	3.662	3.856
6	16.343	3.291	1.931
1	16.690	2.448	2.552
6	14.312	5.352	1.100
1	13.271	5.672	1.234
6	14.650	5.251	2.586
1	13.764	5.136	3.210
6	17.663	6.124	2.380
6	16.506	7.064	2.100
6	16.114	7.265	0.629
6	18.152	5.355	0.977
6	17.624	5.540	-0.599
6	16.594	6.649	-0.692
6	21.234	6.571	3.726
6	21.707	6.265	2.265
1	22.611	5.665	2.281
1	21.928	6.198	4.471
6	20.477	5.389	2.046
1	20.767	4.336	2.029
6	19.961	5.767	3.451
1	19.902	4.886	4.091
6	18.692	6.614	3.500
1	18.334	6.240	4.468
6	17.815	9.063	3.223
1	17.434	9.803	3.932
6	16.646	8.572	2.350
1	15.712	8.964	2.765
6	16.592	8.750	0.799
1	15.810	9.447	0.499
6	18.774	8.106	3.998
1	18.646	8.192	5.078
6	19.697	5.765	0.792
1	20.054	5.015	0.068
6	17.379	7.490	-1.706
1	16.991	7.600	-2.712
6	18.513	6.413	-1.618
1	18.747	6.033	-2.608
6	19.509	7.489	-1.200
1	20.199	7.700	-2.020
6	20.264	7.136	0.075
1	21.220	6.757	-0.322
6	17.992	9.108	0.172

1	17.651	10.084	-0.198
6	18.373	8.534	-1.191
1	18.555	9.310	-1.936
6	21.779	7.841	1.944
6	21.268	8.058	3.355
6	20.017	8.932	3.514
6	19.064	9.616	2.523
6	19.304	9.354	1.049
6	20.717	8.486	0.825
6	24.922	8.929	0.018
6	23.972	9.889	-0.774
1	24.267	9.931	-1.818
1	25.731	8.550	-0.595
6	22.815	8.915	-0.576
1	22.584	8.412	-1.518
6	23.725	8.015	0.288
1	23.858	7.038	-0.179
6	23.366	7.912	1.768
1	23.764	6.901	1.920
6	23.552	9.187	4.045
1	24.143	9.015	4.950
6	22.075	8.893	4.359
1	22.013	8.435	5.351
6	20.941	9.962	4.254
1	20.533	10.217	5.233
6	24.322	8.553	2.843
1	25.096	7.862	3.178
6	21.576	9.578	0.012
1	20.957	9.742	-0.886
6	19.035	11.143	2.396
1	18.103	11.669	2.568
6	19.339	10.954	0.871
1	18.587	11.458	0.273
6	20.590	11.816	1.009
1	20.428	12.788	0.539
6	21.840	11.141	0.458
1	21.931	11.606	-0.537
6	21.368	11.234	3.430
1	21.251	11.910	4.287
6	20.345	11.923	2.530
1	20.162	12.959	2.820
6	24.335	11.097	0.226
6	25.195	10.097	0.974
6	24.939	9.949	2.480
6	23.913	10.555	3.448
6	22.840	11.442	2.845
6	23.138	11.708	1.221
6	25.007	14.456	-1.516
6	24.231	15.107	-0.322
1	23.622	15.933	-0.676

1	24.855	14.990	-2.447
6	23.393	13.841	-0.171
1	22.376	14.021	-0.525
6	24.222	13.163	-1.283
1	23.593	12.921	-2.140
6	25.103	11.993	-0.852
1	25.074	11.459	-1.810
6	27.379	11.420	0.305
1	28.324	11.015	-0.069
6	26.695	10.350	1.175
1	27.244	9.410	1.067
6	26.375	10.545	2.691
1	26.979	9.891	3.319
6	26.670	12.152	-0.879
1	27.101	11.875	-1.842
6	23.391	13.298	1.253
1	22.432	13.680	1.642
6	24.348	11.563	4.518
1	24.153	11.342	5.561
6	23.299	12.556	3.913
1	22.615	12.895	4.684
6	24.402	13.584	3.676
1	24.307	14.405	4.390
6	24.433	14.101	2.244
1	23.900	15.061	2.336
6	26.445	12.051	3.146
1	27.269	11.869	3.848
6	25.451	12.570	4.182
1	25.940	12.938	5.085
6	25.617	15.386	0.447
6	26.296	14.696	-0.721
6	27.237	13.530	-0.388
6	27.618	12.810	0.913
6	26.891	13.238	2.173
6	25.927	14.572	1.875
6	25.880	18.873	2.039
6	26.587	18.154	3.237
1	26.282	18.603	4.178
1	25.285	19.721	2.360
6	25.785	16.887	2.955
1	25.023	16.749	3.725
6	25.160	17.562	1.715
1	24.080	17.663	1.833
6	25.550	16.980	0.359
1	24.620	17.246	-0.159
6	27.470	16.956	-1.418
1	27.564	17.385	-2.420
6	27.365	15.426	-1.547
1	27.249	15.172	-2.604
6	28.401	14.434	-0.927

1	28.973	13.914	-1.696
6	26.449	17.826	-0.619
1	25.860	18.466	-1.277
6	26.661	15.649	2.820
1	26.563	15.190	3.818
6	29.025	12.955	1.502
1	29.625	12.066	1.661
6	28.348	13.461	2.820
1	28.673	12.860	3.664
6	29.146	14.758	2.738
1	29.925	14.767	3.503
6	28.270	16.001	2.833
1	28.391	16.286	3.891
6	29.323	15.105	0.159
1	30.241	14.932	-0.417
6	29.741	14.303	1.389
1	30.823	14.185	1.464
6	28.029	18.488	2.604
6	27.273	19.120	1.451
6	27.625	18.631	0.039
6	28.557	17.539	-0.504
6	29.260	16.662	0.515
6	28.986	17.223	2.067
6	30.618	19.764	5.131
6	31.659	18.889	4.355
1	32.360	18.445	5.055
1	30.833	19.814	6.192
6	30.555	17.902	3.978
1	30.669	16.978	4.549
6	29.514	18.815	4.662
1	29.046	18.304	5.504
6	28.497	19.496	3.750
1	27.690	19.551	4.493
6	28.191	21.507	2.105
1	27.635	22.447	2.170
6	27.497	20.593	1.079
1	26.542	21.048	0.798
6	28.180	20.074	-0.227
1	27.728	20.507	-1.120
6	28.549	21.060	3.558
1	27.953	21.591	4.301
6	30.491	17.620	2.482
1	31.019	16.655	2.406
6	29.828	17.915	-1.273
1	29.958	17.551	-2.286
6	30.628	17.068	-0.228
1	31.225	16.316	-0.733
6	31.474	18.288	0.122
1	32.481	18.171	-0.285
6	31.520	18.568	1.618

1	32.489	18.127	1.909
6	29.747	20.235	-0.208
1	29.767	20.922	-1.064
6	30.621	19.150	-0.837
1	31.233	19.534	-1.655
6	32.091	20.157	3.462
6	31.024	20.938	4.232
6	29.976	21.706	3.421
6	29.686	21.801	1.925
6	30.524	20.938	0.992
6	31.783	20.130	1.851
1	2.716	-8.535	1.210
1	1.737	-7.670	-0.217
1	2.284	-8.537	-2.468
1	3.256	-9.517	-3.093
1	4.496	-10.970	-1.206
1	3.847	-10.147	0.534
1	33.145	20.427	3.392
1	31.319	21.847	4.756
1	30.421	22.621	3.812
1	29.946	22.712	1.386
1	31.099	21.579	0.324
1	32.616	20.708	1.452

.mol2 file

```
@<TRIPOS>MOLECULE
Molecule Name
398 538
SMALL
NO_CHARGES


@<TRIPOS>ATOM
1 C1    3.4376   -8.0318    0.5664 C
2 C2    2.7168   -7.4334   -0.6326 C
3 C3    3.0913   -7.9623   -2.0153 C
4 C4    4.0410   -9.0677   -2.4851 C
5 C5    4.7220   -9.9248   -1.4166 C
6 C6    4.4467   -9.3494    0.0944 C
7 C7    5.9702   -6.7295    3.1443 C
8 C8    7.0278   -7.6250    2.4158 C
9 H9    7.7057   -8.0677    3.1392 H
10 H10    6.1528   -6.6589    4.2104 H
11 C11    5.9319   -8.6104    2.0243 C
12 H12    6.0228   -9.5228    2.6181 H
13 C13    4.8725   -7.6790    2.6563 C
14 H14    4.3787   -8.1734    3.4942 H
15 C15    3.8774   -7.0113    1.7078 C
16 H16    3.0502   -6.9410    2.4261 H
```

17	C17	3.6474	-5.0446	-0.0028	C
18	H18	3.1034	-4.0956	0.0224	H
19	C19	2.9660	-5.9714	-1.0264	C
20	H20	2.0273	-5.5094	-1.3460	H
21	C21	3.6818	-6.5320	-2.2952	C
22	H22	3.2646	-6.1132	-3.2116	H
23	C23	3.9561	-5.4561	1.4727	C
24	H24	3.3492	-4.8962	2.1846	H
25	C25	5.9268	-8.9292	0.5350	C
26	H26	6.4590	-9.8956	0.5007	H
27	C27	5.3367	-8.7276	-3.2324	C
28	H28	5.4876	-9.1211	-4.2312	H
29	C29	6.1073	-9.5506	-2.1469	C
30	H30	6.7028	-10.3226	-2.6257	H
31	C31	6.9567	-8.3255	-1.8146	C
32	H32	7.9723	-8.4602	-2.1925	H
33	C33	6.9633	-7.9892	-0.3281	C
34	H34	7.9235	-8.4188	0.0017	H
35	C35	5.2506	-6.3897	-2.2295	C
36	H36	5.2903	-5.7261	-3.1027	H
37	C37	6.1318	-7.4888	-2.8169	C
38	H38	6.7622	-7.1301	-3.6316	H
39	C39	7.4944	-6.3735	1.5191	C
40	C40	6.4164	-5.5803	2.2338	C
41	C41	5.3958	-4.8412	1.3574	C
42	C42	5.1504	-4.7717	-0.1560	C
43	C43	6.0141	-5.6586	-1.0328	C
44	C44	7.2075	-6.4090	-0.1284	C
45	C45	11.3026	-6.0170	1.1414	C
46	C46	11.0085	-5.6120	-0.3423	C
47	H47	11.7654	-6.0260	-1.0017	H
48	H48	12.2335	-6.5626	1.2436	H
49	C49	9.7570	-6.4848	-0.3376	C
50	H50	9.9232	-7.3798	-0.9407	H
51	C51	9.9919	-6.8074	1.1539	C
52	H52	10.1731	-7.8738	1.2946	H
53	C53	8.9630	-6.2529	2.1365	C
54	H54	9.0749	-7.0446	2.8882	H
55	C55	8.2492	-4.0681	3.3854	C
56	H56	8.3040	-3.7563	4.4326	H
57	C57	6.7734	-4.3127	3.0227	C
58	H58	6.1773	-4.2704	3.9391	H
59	C59	6.0119	-3.5121	1.9191	C
60	H60	5.2371	-2.8737	2.3447	H
61	C61	9.3842	-5.1115	3.1368	C
62	H62	9.7851	-5.5037	4.0720	H
63	C63	8.5106	-5.7378	-0.7945	C
64	H64	8.4257	-6.0567	-1.8467	H
65	C65	5.4310	-3.4817	-0.9350	C
66	H66	4.6266	-3.0180	-1.4942	H

67	C67	6.3856	-4.3214	-1.8489	C
68	H68	6.0983	-4.2146	-2.8906	H
69	C69	7.5419	-3.3863	-1.5071	C
70	H70	7.7792	-2.7555	-2.3663	H
71	C71	8.7770	-4.1269	-1.0114	C
72	H72	9.4019	-4.1620	-1.9192	H
73	C73	6.9756	-2.7263	0.9523	C
74	H74	6.5765	-1.7464	1.2436	H
75	C75	6.6270	-2.6167	-0.5302	C
76	H76	6.4974	-1.5835	-0.8548	H
77	C77	11.0418	-4.0543	0.0614	C
78	C78	11.2720	-4.5235	1.4852	C
79	C79	10.3130	-3.9877	2.5574	C
80	C80	9.0622	-3.0987	2.5160	C
81	C81	8.5606	-2.6821	1.1467	C
82	C82	9.6308	-3.1627	-0.0455	C
83	C83	12.9847	-1.8177	-2.3864	C
84	C84	11.8501	-0.7639	-2.1540	C
85	H85	11.6219	-0.2470	-3.0812	H
86	H86	13.3790	-1.7840	-3.3955	H
87	C87	10.8509	-1.8886	-1.8997	C
88	H88	10.1669	-1.9796	-2.7460	H
89	C89	11.9881	-2.9243	-2.0323	C
90	H90	11.8113	-3.5857	-2.8815	H
91	C91	12.3601	-3.6868	-0.7628	C
92	H92	12.7043	-4.5997	-1.2654	H
93	C93	13.6914	-3.4673	1.4782	C
94	H94	14.6224	-3.9146	1.8391	H
95	C95	12.5169	-4.0768	2.2644	C
96	H96	12.8937	-4.9108	2.8640	H
97	C97	11.5520	-3.2393	3.1624	C
98	H98	11.6731	-3.4789	4.2193	H
99	C99	13.7553	-3.4222	-0.0814	C
100	H100	14.5401	-4.0701	-0.4730	H
101	C101	10.0881	-1.7193	-0.5921	C
102	H102	9.1346	-1.2869	-0.9382	H
103	C103	9.0935	-1.6800	3.0957	C
104	H104	8.3952	-1.4159	3.8815	H
105	C105	8.6305	-1.1687	1.6900	C
106	H106	7.7260	-0.5757	1.7854	H
107	C107	9.8613	-0.2710	1.6056	C
108	H108	9.5719	0.7738	1.7369	H
109	C109	10.6493	-0.4653	0.3165	C
110	H110	10.2972	0.3811	-0.2963	H
111	C111	11.6378	-1.6917	2.8821	C
112	H112	11.9894	-1.4604	3.8956	H
113	C113	10.3683	-0.8459	2.9458	C
114	H114	10.4230	-0.0649	3.7053	H
115	C115	12.7314	-0.0453	-1.0149	C
116	C116	13.7626	-1.1305	-1.2584	C

117	C117	14.2340	-1.9288	-0.0349	C
118	C118	13.8323	-1.9391	1.4467	C
119	C119	12.6737	-1.0475	1.8504	C
120	C120	12.1934	-0.0868	0.5682	C
121	C121	12.7609	3.7926	-1.2105	C
122	C122	12.6889	3.7646	0.3534	C
123	H123	12.0501	4.5649	0.7145	H
124	H124	12.2302	4.6380	-1.6330	H
125	C125	11.9262	2.4442	0.2994	C
126	H126	10.8790	2.6061	0.5632	H
127	C127	12.0813	2.4213	-1.2365	C
128	H128	11.1068	2.4706	-1.7241	H
129	C129	12.9690	1.3195	-1.8106	C
130	H130	12.4525	1.2371	-2.7753	H
131	C131	15.4698	0.6535	-2.1920	C
132	H132	16.0942	0.5835	-3.0877	H
133	C133	15.1960	-0.7691	-1.6720	C
134	H134	15.5562	-1.4874	-2.4146	H
135	C135	15.6412	-1.2738	-0.2629	C
136	H136	16.4181	-2.0357	-0.3329	H
137	C137	14.3463	1.7011	-2.4727	C
138	H138	14.2514	1.9170	-3.5373	H
139	C139	12.5516	1.3623	1.1705	C
140	H140	11.9206	1.4033	2.0739	H
141	C141	14.7952	-1.4188	2.5200	C
142	H142	15.0970	-2.0809	3.3233	H
143	C143	13.6645	-0.4277	2.9572	C
144	H144	13.4503	-0.5452	4.0151	H
145	C145	14.6014	0.7531	2.7212	C
146	H146	14.9157	1.1779	3.6768	H
147	C147	13.9952	1.8196	1.8182	C
148	H148	13.6433	2.5655	2.5501	H
149	C149	16.0325	-0.1028	0.7153	C
150	H150	17.0728	-0.4397	0.8087	H
151	C151	15.6842	-0.2151	2.1976	C
152	H152	16.5667	-0.1907	2.8382	H
153	C153	14.2906	3.8952	0.4437	C
154	C154	14.2860	3.8628	-1.0726	C
155	C155	15.1810	2.8145	-1.7483	C
156	C156	16.0720	1.6803	-1.2229	C
157	C157	16.0575	1.4323	0.2733	C
158	C158	15.1717	2.6136	1.0590	C
159	C159	17.5979	1.7687	-1.3394	C
160	C160	17.6597	1.5737	0.2130	C
161	H161	18.2965	0.7299	0.4606	H
162	H162	18.1318	0.9919	-1.8745	H
163	C163	18.4221	2.8880	0.3526	C
164	H164	19.4676	2.6897	0.5975	H
165	C165	18.2770	3.1295	-1.1654	C
166	H166	19.2547	3.1497	-1.6486	H

167	C167	17.3926	4.3022	-1.5825	C
168	H168	17.9154	4.5210	-2.5222	H
169	C169	14.8941	5.0169	-1.8816	C
170	H170	14.2755	5.2140	-2.7623	H
171	C171	15.1637	6.3508	-1.1625	C
172	H172	14.8080	7.1677	-1.7975	H
173	C173	14.7090	6.6498	0.3012	C
174	H174	13.9322	7.4142	0.3353	H
175	C175	16.0199	4.0194	-2.3013	C
176	H176	16.1219	3.9573	-3.3851	H
177	C177	17.7905	3.8351	1.3648	C
178	H178	18.4156	3.6656	2.2572	H
179	C179	15.5367	6.3965	3.0819	C
180	H180	15.2293	6.9377	3.9693	H
181	C181	16.6650	5.3529	3.3808	C
182	H182	16.8722	5.3184	4.4461	H
183	C183	15.7302	4.2181	2.9729	C
184	H184	15.4099	3.6616	3.8562	H
185	C185	16.3428	3.2908	1.9312	C
186	H186	16.6903	2.4481	2.5517	H
187	C187	14.3119	5.3517	1.1000	C
188	H188	13.2708	5.6722	1.2337	H
189	C189	14.6504	5.2514	2.5855	C
190	H190	13.7637	5.1365	3.2103	H
191	C191	17.6627	6.1236	2.3801	C
192	C192	16.5062	7.0640	2.0999	C
193	C193	16.1143	7.2651	0.6294	C
194	C194	18.1519	5.3551	0.9774	C
195	C195	17.6242	5.5398	-0.5990	C
196	C196	16.5941	6.6490	-0.6923	C
197	C197	21.2344	6.5711	3.7261	C
198	C198	21.7070	6.2653	2.2650	C
199	H199	22.6113	5.6646	2.2807	H
200	H200	21.9277	6.1976	4.4708	H
201	C201	20.4774	5.3890	2.0455	C
202	H202	20.7666	4.3362	2.0286	H
203	C203	19.9612	5.7672	3.4506	C
204	H204	19.9020	4.8860	4.0907	H
205	C205	18.6915	6.6138	3.4998	C
206	H206	18.3335	6.2405	4.4677	H
207	C207	17.8149	9.0629	3.2225	C
208	H208	17.4336	9.8029	3.9324	H
209	C209	16.6459	8.5721	2.3497	C
210	H210	15.7123	8.9637	2.7645	H
211	C211	16.5923	8.7497	0.7992	C
212	H212	15.8097	9.4470	0.4987	H
213	C213	18.7743	8.1056	3.9983	C
214	H214	18.6461	8.1921	5.0777	H
215	C215	19.6974	5.7651	0.7923	C
216	H216	20.0540	5.0145	0.0675	H

217	C217	17.3790	7.4896	-1.7057	C
218	H218	16.9914	7.6000	-2.7119	H
219	C219	18.5126	6.4129	-1.6183	C
220	H220	18.7473	6.0333	-2.6081	H
221	C221	19.5095	7.4895	-1.1998	C
222	H222	20.1990	7.7000	-2.0200	H
223	C223	20.2638	7.1356	0.0754	C
224	H224	21.2198	6.7565	-0.3224	H
225	C225	17.9918	9.1083	0.1716	C
226	H226	17.6505	10.0835	-0.1982	H
227	C227	18.3726	8.5341	-1.1910	C
228	H228	18.5546	9.3096	-1.9362	H
229	C229	21.7794	7.8408	1.9436	C
230	C230	21.2684	8.0580	3.3549	C
231	C231	20.0167	8.9322	3.5143	C
232	C232	19.0644	9.6157	2.5231	C
233	C233	19.3044	9.3542	1.0485	C
234	C234	20.7165	8.4862	0.8250	C
235	C235	24.9224	8.9293	0.0183	C
236	C236	23.9722	9.8890	-0.7742	C
237	H237	24.2668	9.9314	-1.8184	H
238	H238	25.7314	8.5503	-0.5952	H
239	C239	22.8149	8.9147	-0.5761	C
240	H240	22.5838	8.4121	-1.5176	H
241	C241	23.7248	8.0149	0.2878	C
242	H242	23.8582	7.0383	-0.1795	H
243	C243	23.3658	7.9123	1.7683	C
244	H244	23.7637	6.9009	1.9204	H
245	C245	23.5525	9.1873	4.0448	C
246	H246	24.1427	9.0145	4.9497	H
247	C247	22.0747	8.8933	4.3591	C
248	H248	22.0132	8.4355	5.3508	H
249	C249	20.9407	9.9618	4.2542	C
250	H250	20.5333	10.2166	5.2330	H
251	C251	24.3223	8.5532	2.8431	C
252	H252	25.0959	7.8616	3.1780	H
253	C253	21.5759	9.5777	0.0118	C
254	H254	20.9570	9.7419	-0.8859	H
255	C255	19.0350	11.1430	2.3956	C
256	H256	18.1030	11.6686	2.5683	H
257	C257	19.3393	10.9536	0.8714	C
258	H258	18.5866	11.4576	0.2727	H
259	C259	20.5902	11.8164	1.0090	C
260	H260	20.4277	12.7884	0.5387	H
261	C261	21.8395	11.1411	0.4581	C
262	H262	21.9306	11.6063	-0.5374	H
263	C263	21.3678	11.2342	3.4299	C
264	H264	21.2510	11.9102	4.2865	H
265	C265	20.3450	11.9233	2.5296	C
266	H266	20.1622	12.9588	2.8196	H

267	C267	24.3353	11.0971	0.2256 C
268	C268	25.1949	10.0965	0.9739 C
269	C269	24.9389	9.9494	2.4804 C
270	C270	23.9125	10.5550	3.4481 C
271	C271	22.8400	11.4419	2.8453 C
272	C272	23.1383	11.7084	1.2212 C
273	C273	25.0073	14.4564	-1.5158 C
274	C274	24.2308	15.1068	-0.3218 C
275	H275	23.6216	15.9331	-0.6756 H
276	H276	24.8549	14.9901	-2.4468 H
277	C277	23.3928	13.8408	-0.1706 C
278	H278	22.3758	14.0209	-0.5251 H
279	C279	24.2223	13.1632	-1.2826 C
280	H280	23.5935	12.9206	-2.1402 H
281	C281	25.1032	11.9928	-0.8517 C
282	H282	25.0743	11.4591	-1.8102 H
283	C283	27.3786	11.4204	0.3049 C
284	H284	28.3236	11.0153	-0.0690 H
285	C285	26.6955	10.3501	1.1748 C
286	H286	27.2445	9.4099	1.0666 H
287	C287	26.3748	10.5452	2.6906 C
288	H288	26.9792	9.8908	3.3194 H
289	C289	26.6700	12.1520	-0.8789 C
290	H290	27.1005	11.8747	-1.8416 H
291	C291	23.3908	13.2978	1.2526 C
292	H292	22.4324	13.6798	1.6415 H
293	C293	24.3482	11.5627	4.5179 C
294	H294	24.1532	11.3418	5.5609 H
295	C295	23.2995	12.5556	3.9126 C
296	H296	22.6145	12.8947	4.6838 H
297	C297	24.4021	13.5835	3.6762 C
298	H298	24.3070	14.4048	4.3896 H
299	C299	24.4333	14.1005	2.2437 C
300	H300	23.9004	15.0614	2.3359 H
301	C301	26.4455	12.0514	3.1461 C
302	H302	27.2690	11.8694	3.8483 H
303	C303	25.4509	12.5698	4.1822 C
304	H304	25.9402	12.9381	5.0848 H
305	C305	25.6173	15.3858	0.4466 C
306	C306	26.2961	14.6961	-0.7212 C
307	C307	27.2373	13.5300	-0.3884 C
308	C308	27.6179	12.8095	0.9127 C
309	C309	26.8908	13.2376	2.1729 C
310	C310	25.9275	14.5718	1.8745 C
311	C311	25.8796	18.8733	2.0392 C
312	C312	26.5869	18.1544	3.2370 C
313	H313	26.2815	18.6029	4.1775 H
314	H314	25.2850	19.7210	2.3596 H
315	C315	25.7849	16.8873	2.9553 C
316	H316	25.0225	16.7485	3.7248 H

317	C317	25.1597	17.5620	1.7154	C
318	H318	24.0800	17.6625	1.8333	H
319	C319	25.5497	16.9795	0.3589	C
320	H320	24.6197	17.2456	-0.1594	H
321	C321	27.4697	16.9559	-1.4178	C
322	H322	27.5636	17.3847	-2.4199	H
323	C323	27.3653	15.4256	-1.5465	C
324	H324	27.2492	15.1717	-2.6044	H
325	C325	28.4007	14.4340	-0.9274	C
326	H326	28.9729	13.9142	-1.6964	H
327	C327	26.4487	17.8263	-0.6186	C
328	H328	25.8604	18.4661	-1.2770	H
329	C329	26.6609	15.6487	2.8201	C
330	H330	26.5628	15.1896	3.8178	H
331	C331	29.0254	12.9546	1.5022	C
332	H332	29.6247	12.0656	1.6613	H
333	C333	28.3476	13.4605	2.8200	C
334	H334	28.6728	12.8599	3.6640	H
335	C335	29.1458	14.7582	2.7382	C
336	H336	29.9253	14.7665	3.5029	H
337	C337	28.2699	16.0009	2.8328	C
338	H338	28.3907	16.2858	3.8912	H
339	C339	29.3229	15.1054	0.1587	C
340	H340	30.2409	14.9318	-0.4171	H
341	C341	29.7413	14.3028	1.3885	C
342	H342	30.8231	14.1851	1.4636	H
343	C343	28.0292	18.4878	2.6040	C
344	C344	27.2733	19.1199	1.4506	C
345	C345	27.6245	18.6305	0.0390	C
346	C346	28.5568	17.5388	-0.5040	C
347	C347	29.2601	16.6616	0.5149	C
348	C348	28.9861	17.2226	2.0667	C
349	C349	30.6183	19.7635	5.1309	C
350	C350	31.6586	18.8891	4.3546	C
351	H351	32.3601	18.4449	5.0548	H
352	H352	30.8333	19.8136	6.1922	H
353	C353	30.5552	17.9024	3.9785	C
354	H354	30.6688	16.9782	4.5489	H
355	C355	29.5137	18.8151	4.6621	C
356	H356	29.0456	18.3039	5.5043	H
357	C357	28.4965	19.4962	3.7504	C
358	H358	27.6899	19.5513	4.4926	H
359	C359	28.1911	21.5068	2.1053	C
360	H360	27.6348	22.4465	2.1697	H
361	C361	27.4966	20.5926	1.0791	C
362	H362	26.5424	21.0484	0.7980	H
363	C363	28.1801	20.0743	-0.2269	C
364	H364	27.7283	20.5074	-1.1197	H
365	C365	28.5492	21.0601	3.5575	C
366	H366	27.9533	21.5907	4.3010	H

367 C367 30.4914 17.6203 2.4820 C
368 H368 31.0187 16.6552 2.4062 H
369 C369 29.8278 17.9151 -1.2734 C
370 H370 29.9581 17.5514 -2.2861 H
371 C371 30.6281 17.0683 -0.2276 C
372 H372 31.2253 16.3157 -0.7334 H
373 C373 31.4735 18.2884 0.1215 C
374 H374 32.4808 18.1713 -0.2847 H
375 C375 31.5198 18.5681 1.6179 C
376 H376 32.4889 18.1269 1.9087 H
377 C377 29.7467 20.2347 -0.2076 C
378 H378 29.7665 20.9216 -1.0637 H
379 C379 30.6206 19.1503 -0.8370 C
380 H380 31.2327 19.5339 -1.6546 H
381 C381 32.0906 20.1575 3.4617 C
382 C382 31.0241 20.9383 4.2319 C
383 C383 29.9764 21.7059 3.4209 C
384 C384 29.6863 21.8006 1.9247 C
385 C385 30.5238 20.9384 0.9917 C
386 C386 31.7826 20.1295 1.8510 C
387 H387 2.7159 -8.5354 1.2095 H
388 H388 1.7374 -7.6702 -0.2170 H
389 H389 2.2835 -8.5373 -2.4681 H
390 H390 3.2557 -9.5173 -3.0928 H
391 H391 4.4960 -10.9701 -1.2056 H
392 H392 3.8473 -10.1466 0.5340 H
393 H393 33.1446 20.4265 3.3924 H
394 H394 31.3192 21.8473 4.7561 H
395 H395 30.4213 22.6209 3.8120 H
396 H396 29.9464 22.7121 1.3864 H
397 H397 31.0993 21.5795 0.3238 H
398 H398 32.6159 20.7076 1.4517 H
@<TRIPOS>BOND
1 1 2 1
2 1 6 1
3 1 15 1
4 1 387 1
5 2 3 1
6 2 19 1
7 2 388 1
8 3 4 1
9 3 21 1
10 3 389 1
11 4 5 1
12 4 27 1
13 4 390 1
14 5 6 1
15 5 29 1
16 5 391 1
17 6 25 1

18 6 392 1
19 7 8 1
20 7 10 1
21 7 13 1
22 7 40 1
23 8 9 1
24 8 11 1
25 8 39 1
26 11 12 1
27 11 13 1
28 11 25 1
29 13 14 1
30 13 15 1
31 15 16 1
32 15 23 1
33 17 18 1
34 17 19 1
35 17 23 1
36 17 42 1
37 19 20 1
38 19 21 1
39 21 22 1
40 21 35 1
41 23 24 1
42 23 41 1
43 25 26 1
44 25 33 1
45 27 28 1
46 27 29 1
47 27 37 1
48 29 30 1
49 29 31 1
50 31 32 1
51 31 33 1
52 31 37 1
53 33 34 1
54 33 44 1
55 35 36 1
56 35 37 1
57 35 43 1
58 37 38 1
59 39 40 1
60 39 44 1
61 39 53 1
62 40 41 1
63 40 57 1
64 41 42 1
65 41 59 1
66 42 43 1
67 42 65 1

68 43 44 1
69 43 67 1
70 44 63 1
71 45 46 1
72 45 48 1
73 45 51 1
74 45 78 1
75 46 47 1
76 46 49 1
77 46 77 1
78 49 50 1
79 49 51 1
80 49 63 1
81 51 52 1
82 51 53 1
83 53 54 1
84 53 61 1
85 55 56 1
86 55 57 1
87 55 61 1
88 55 80 1
89 57 58 1
90 57 59 1
91 59 60 1
92 59 73 1
93 61 62 1
94 61 79 1
95 63 64 1
96 63 71 1
97 65 66 1
98 65 67 1
99 65 75 1
100 67 68 1
101 67 69 1
102 69 70 1
103 69 71 1
104 69 75 1
105 71 72 1
106 71 82 1
107 73 74 1
108 73 75 1
109 73 81 1
110 75 76 1
111 77 78 1
112 77 82 1
113 77 91 1
114 78 79 1
115 78 95 1
116 79 80 1
117 79 97 1

118 80 81 1
119 80 103 1
120 81 82 1
121 81 105 1
122 82 101 1
123 83 84 1
124 83 86 1
125 83 89 1
126 83 116 1
127 84 85 1
128 84 87 1
129 84 115 1
130 87 88 1
131 87 89 1
132 87 101 1
133 89 90 1
134 89 91 1
135 91 92 1
136 91 99 1
137 93 94 1
138 93 95 1
139 93 99 1
140 93 118 1
141 95 96 1
142 95 97 1
143 97 98 1
144 97 111 1
145 99 100 1
146 99 117 1
147 101 102 1
148 101 109 1
149 103 104 1
150 103 105 1
151 103 113 1
152 105 106 1
153 105 107 1
154 107 108 1
155 107 109 1
156 107 113 1
157 109 110 1
158 109 120 1
159 111 112 1
160 111 113 1
161 111 119 1
162 113 114 1
163 115 116 1
164 115 120 1
165 115 129 1
166 116 117 1
167 116 133 1

168 117 118 1
169 117 135 1
170 118 119 1
171 118 141 1
172 119 120 1
173 119 143 1
174 120 139 1
175 121 122 1
176 121 124 1
177 121 127 1
178 121 154 1
179 122 123 1
180 122 125 1
181 122 153 1
182 125 126 1
183 125 127 1
184 125 139 1
185 127 128 1
186 127 129 1
187 129 130 1
188 129 137 1
189 131 132 1
190 131 133 1
191 131 137 1
192 131 156 1
193 133 134 1
194 133 135 1
195 135 136 1
196 135 149 1
197 137 138 1
198 137 155 1
199 139 140 1
200 139 147 1
201 141 142 1
202 141 143 1
203 141 151 1
204 143 144 1
205 143 145 1
206 145 146 1
207 145 147 1
208 145 151 1
209 147 148 1
210 147 158 1
211 149 150 1
212 149 151 1
213 149 157 1
214 151 152 1
215 153 154 1
216 153 158 1
217 153 187 1

218 154 155 1
219 154 169 1
220 155 156 1
221 155 175 1
222 156 157 1
223 156 159 1
224 157 158 1
225 157 160 1
226 158 185 1
227 159 160 1
228 159 162 1
229 159 165 1
230 160 161 1
231 160 163 1
232 163 164 1
233 163 165 1
234 163 177 1
235 165 166 1
236 165 167 1
237 167 168 1
238 167 175 1
239 167 195 1
240 169 170 1
241 169 171 1
242 169 175 1
243 171 172 1
244 171 173 1
245 171 196 1
246 173 174 1
247 173 187 1
248 173 193 1
249 175 176 1
250 177 178 1
251 177 185 1
252 177 194 1
253 179 180 1
254 179 181 1
255 179 189 1
256 179 192 1
257 181 182 1
258 181 183 1
259 181 191 1
260 183 184 1
261 183 185 1
262 183 189 1
263 185 186 1
264 187 188 1
265 187 189 1
266 189 190 1
267 191 192 1

268 191 194 1
269 191 205 1
270 192 193 1
271 192 209 1
272 193 196 1
273 193 211 1
274 194 195 1
275 194 215 1
276 195 196 1
277 195 219 1
278 196 217 1
279 197 198 1
280 197 200 1
281 197 203 1
282 197 230 1
283 198 199 1
284 198 201 1
285 198 229 1
286 201 202 1
287 201 203 1
288 201 215 1
289 203 204 1
290 203 205 1
291 205 206 1
292 205 213 1
293 207 208 1
294 207 209 1
295 207 213 1
296 207 232 1
297 209 210 1
298 209 211 1
299 211 212 1
300 211 225 1
301 213 214 1
302 213 231 1
303 215 216 1
304 215 223 1
305 217 218 1
306 217 219 1
307 217 227 1
308 219 220 1
309 219 221 1
310 221 222 1
311 221 223 1
312 221 227 1
313 223 224 1
314 223 234 1
315 225 226 1
316 225 227 1
317 225 233 1

318 227 228 1
319 229 230 1
320 229 234 1
321 229 243 1
322 230 231 1
323 230 247 1
324 231 232 1
325 231 249 1
326 232 233 1
327 232 255 1
328 233 234 1
329 233 257 1
330 234 253 1
331 235 236 1
332 235 238 1
333 235 241 1
334 235 268 1
335 236 237 1
336 236 239 1
337 236 267 1
338 239 240 1
339 239 241 1
340 239 253 1
341 241 242 1
342 241 243 1
343 243 244 1
344 243 251 1
345 245 246 1
346 245 247 1
347 245 251 1
348 245 270 1
349 247 248 1
350 247 249 1
351 249 250 1
352 249 263 1
353 251 252 1
354 251 269 1
355 253 254 1
356 253 261 1
357 255 256 1
358 255 257 1
359 255 265 1
360 257 258 1
361 257 259 1
362 259 260 1
363 259 261 1
364 259 265 1
365 261 262 1
366 261 272 1
367 263 264 1

368 263 265 1
369 263 271 1
370 265 266 1
371 267 268 1
372 267 272 1
373 267 281 1
374 268 269 1
375 268 285 1
376 269 270 1
377 269 287 1
378 270 271 1
379 270 293 1
380 271 272 1
381 271 295 1
382 272 291 1
383 273 274 1
384 273 276 1
385 273 279 1
386 273 306 1
387 274 275 1
388 274 277 1
389 274 305 1
390 277 278 1
391 277 279 1
392 277 291 1
393 279 280 1
394 279 281 1
395 281 282 1
396 281 289 1
397 283 284 1
398 283 285 1
399 283 289 1
400 283 308 1
401 285 286 1
402 285 287 1
403 287 288 1
404 287 301 1
405 289 290 1
406 289 307 1
407 291 292 1
408 291 299 1
409 293 294 1
410 293 295 1
411 293 303 1
412 295 296 1
413 295 297 1
414 297 298 1
415 297 299 1
416 297 303 1
417 299 300 1

418 299 310 1
419 301 302 1
420 301 303 1
421 301 309 1
422 303 304 1
423 305 306 1
424 305 310 1
425 305 319 1
426 306 307 1
427 306 323 1
428 307 308 1
429 307 325 1
430 308 309 1
431 308 331 1
432 309 310 1
433 309 333 1
434 310 329 1
435 311 312 1
436 311 314 1
437 311 317 1
438 311 344 1
439 312 313 1
440 312 315 1
441 312 343 1
442 315 316 1
443 315 317 1
444 315 329 1
445 317 318 1
446 317 319 1
447 319 320 1
448 319 327 1
449 321 322 1
450 321 323 1
451 321 327 1
452 321 346 1
453 323 324 1
454 323 325 1
455 325 326 1
456 325 339 1
457 327 328 1
458 327 345 1
459 329 330 1
460 329 337 1
461 331 332 1
462 331 333 1
463 331 341 1
464 333 334 1
465 333 335 1
466 335 336 1
467 335 337 1

468 335 341 1
469 337 338 1
470 337 348 1
471 339 340 1
472 339 341 1
473 339 347 1
474 341 342 1
475 343 344 1
476 343 348 1
477 343 357 1
478 344 345 1
479 344 361 1
480 345 346 1
481 345 363 1
482 346 347 1
483 346 369 1
484 347 348 1
485 347 371 1
486 348 367 1
487 349 350 1
488 349 352 1
489 349 355 1
490 349 382 1
491 350 351 1
492 350 353 1
493 350 381 1
494 353 354 1
495 353 355 1
496 353 367 1
497 355 356 1
498 355 357 1
499 357 358 1
500 357 365 1
501 359 360 1
502 359 361 1
503 359 365 1
504 359 384 1
505 361 362 1
506 361 363 1
507 363 364 1
508 363 377 1
509 365 366 1
510 365 383 1
511 367 368 1
512 367 375 1
513 369 370 1
514 369 371 1
515 369 379 1
516 371 372 1
517 371 373 1

518 373 374 1
519 373 375 1
520 373 379 1
521 375 376 1
522 375 386 1
523 377 378 1
524 377 379 1
525 377 385 1
526 379 380 1
527 381 382 1
528 381 386 1
529 381 393 1
530 382 383 1
531 382 394 1
532 383 384 1
533 383 395 1
534 384 385 1
535 384 396 1
536 385 386 1
537 385 397 1
538 386 398 1

Figure S27. Perspective of a helix made by fusion of nuggets$_{28b}$ via its hexagonal face, of formula $C_{226}H_{172}$. Cartesian coordinates of its atoms; the first line contains the total charge and multiplicity; the following lines contain the atomic numbers, followed by the x, y, and z coordinates in Å for each one of the atoms. Next, atomic coordinates in Tripos Mol2 file format (.mol2) with the distances also in Å.
